# Supplementary material for: Spectral measure of color variation of black-orange-black (BOB) pattern in small parasitoid wasps (Hymenoptera: Scelionidae), a statistical approach
Source: PLoS One. 2019 Oct 24;14(10):e0218061. doi: 10.1371/journal.pone.0218061 (PMC6812806; doi:10.1371/journal.pone.0218061)
Supplement: S1 Appendix — (PDF) [file pone.0218061.s001.pdf]

S1 Appendix. Color coordinates calculation

The tristimulus values for color coordinates, according to the CIE (International Commission on Illumination, or Commission Internationale d'Eclairage) definition, were calculated as follows:

$$X = k \sum \phi_\lambda \bar{x}(\lambda)\, \Delta\lambda \tag{1}$$

$$Y = k \sum \phi_\lambda \bar{y}(\lambda)\, \Delta\lambda \tag{2}$$

$$Z = k \sum \phi_\lambda \bar{z}(\lambda)\, \Delta\lambda \tag{3}$$

where $\bar{x}(\lambda)$, $\bar{y}(\lambda)$ and $\bar{z}(\lambda)$ are the standardized color-matching functions (CMF) of the CIE 1931 standard observer. The constant k in equations (1), (2) and (3) is defined so that Y=100 for objects with perfect reflectivity. Finally, ϕ_λ is the relative color stimulus function, defined as:

$$\phi_\lambda = R(\lambda)\, S(\lambda) \tag{4}$$

with $R(\lambda)$being the spectral reflectance factor (the measure spectra) and $S(\lambda)$ is the relative spectral power distribution (SPD) of the illuminat.

These CIE-XYZ tristimulus values were calculated using the measured spectra (Fig 2). A D- 65 day light standard illuminant for the CIE observer was chosen. Following the CIE recommendations, a spacing of 1 nm for wavelength was used. Since the data measured with the microspectrophotometer have a different wavelength spacing, an interpolation was made using the splinefun function in R [33], to generate a list of spectral points with the desired spacing.

After the tristimulus values were obtained, they were used to calculate the CIE 1976 (L* a* b*) (CIELAB) color space coordinates. The coordinates in the CIELAB color space are:

$$L = 116 f(Y/Y_n) - 16 \tag{5}$$

$$a = 500 \left[f(X/X_n) - f(Y/Y_n) \right] \tag{6}$$

$$b = 200 \left[f(X/X_n) - f(Z/Z_n) \right] \tag{7}$$

where

$$f(r) = r^{1/3} \text{ if } r > (24/116)^3 \tag{8}$$

and

$$f(r) = (841/108)\, r + 16/116 \text{ if } r \leq (24/116)^3 \tag{9}$$

with X_n, Y_n and Z_n being the tristimulus values of a idealized white reflector.
