# Supplementary material for: Spectral measure of color variation of black-orange-black (BOB) pattern in small parasitoid wasps (Hymenoptera: Scelionidae), a statistical approach
Source: PLoS One. 2019 Oct 24;14(10):e0218061. doi: 10.1371/journal.pone.0218061 (PMC6812806; doi:10.1371/journal.pone.0218061)
Supplement: S2 Appendix — (PDF) [file pone.0218061.s002.pdf]

S2 Appendix. Univariate comparison for the inter-genera case

For the univariate case, Table 2 shows how *Baryconus*, *Opisthacantha*, and *Scelio* absolute differences are not significantly different from zero. This contradicts the functional data analysis result presented before, where all the difference curves are different between genera.

When describing the difference between genera, Table 2 shows how there are three groups of genera without univariate mean differences: group *a* consists of *Baryconus*, *Opisthacantha*, *Scelio*, *Acanthoscelio* and *Chromoteleia*, while group *b* consist of *Triteleia*, *Sceliomorpha*, *Macroteleia* and *Evaniella* as members. Lastly, *Acanthoscelio* and *Chromoteleia* (group *c*) seem not to differ in terms of univariate means. On the other hand, when examining the curves in Fig 3, it is clear that they differ, with *Triteleia*'s difference curve being very close to zero from $\lambda = 420$ nm to $\lambda = 590$ nm and then going to a negative difference of 20 around $\lambda = 780$ nm, while the curve for *Macroteleia* deviates from zero around $\lambda = 500$ nm and reaches -20 at $\lambda = 680$ nm. Given that other characteristics from the curve are compared in this case, it is reasonable to have a contradictory result. The pattern of contradictory results between univariate and functional results is repeated in all pairs of genera belonging to the same univariate group (a,b,c), which could indicate one of the limitations of the univariate analysis: it ignores the fact that the spectrometer measurements are curves and they should be compared as such.
