# Supplementary material for: Spectral measure of color variation of black-orange-black (BOB) pattern in small parasitoid wasps (Hymenoptera: Scelionidae), a statistical approach
Source: PLoS One. 2019 Oct 24;14(10):e0218061. doi: 10.1371/journal.pone.0218061 (PMC6812806; doi:10.1371/journal.pone.0218061)
Supplement: S3 Appendix — (PDF) [file pone.0218061.s003.pdf]

S3 Appendix. Eosin hematoxylin technique.

The specimens were fixed in 10% formalin, where they were preserved until they were processed. Subsequently, a post fixation in formaldehyde, acetic acid, ethanol solution was carried out for two hours. Dehydration was carried out in alcohols with increasing concentration (70%, 95%,100%). Clarification was implemented with xylol and later the sample was impregnated with paraffin. Once the specimens were processed, they were included in paraffin blocks. Continuous cuts were made at 4 microns in thickness and 3 or 4 cuts were placed per slide, using the entire mesoscutum. Hematoxylin and eosin stains, in a VENTANA HE 600 system, were performed on all sections. The final cuts were observed in an Olympus bx51 microscope and the photographs obtained with an Olympus Dp72 camera.
