# Supplementary material for: Spectral measure of color variation of black-orange-black (BOB) pattern in small parasitoid wasps (Hymenoptera: Scelionidae), a statistical approach
Source: PLoS One. 2019 Oct 24;14(10):e0218061. doi: 10.1371/journal.pone.0218061 (PMC6812806; doi:10.1371/journal.pone.0218061)

S1 Fig. Eosin hematoxylin technique. Cross section of the cuticle shows the pigment concentrated in the epicuticle, of a specimen of *Baryconus* with black-orange-black color (a) and black color (b). The variation in the distribution of the pigment together with the roughness of the cuticle surface can contribute to the reflection of light in different directions and therefore to the dispersion of the data

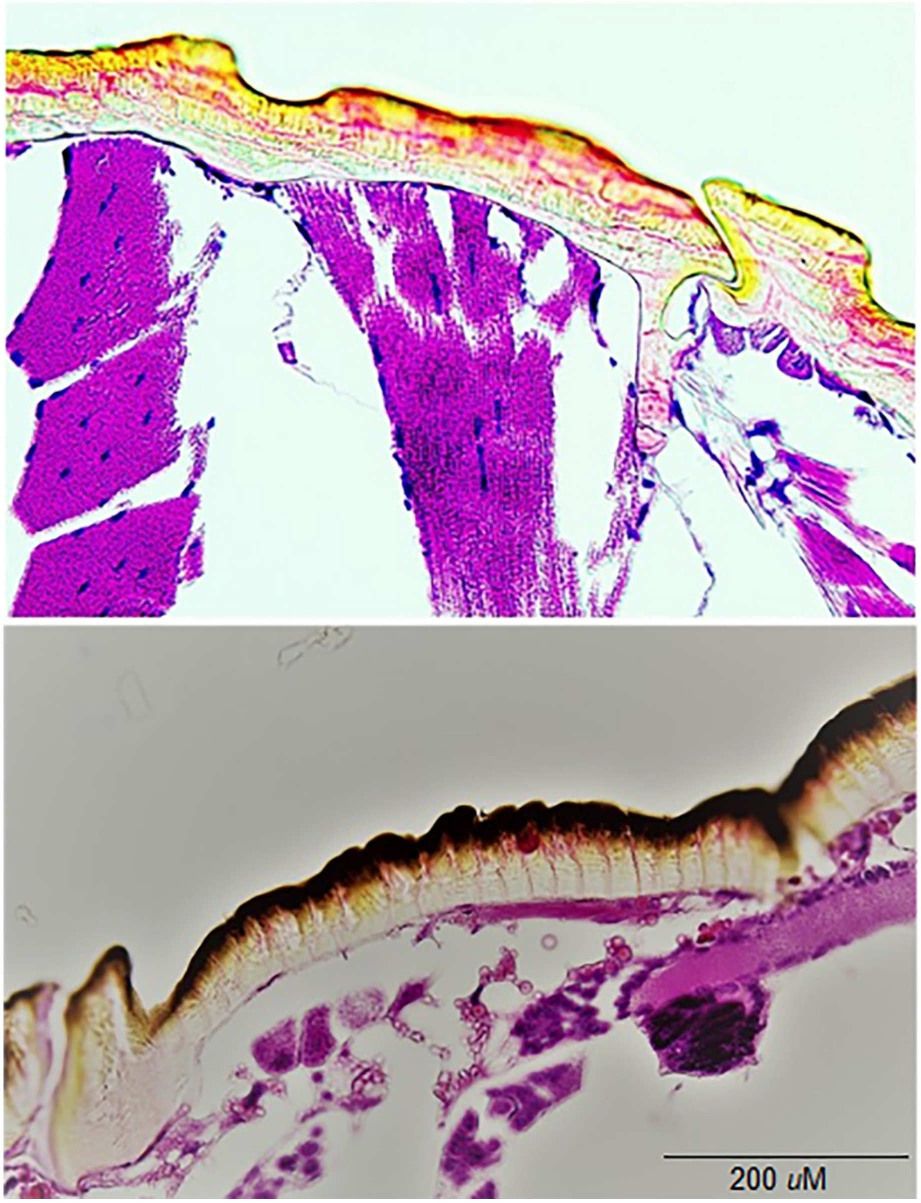

Supplement: S1 Fig — Cross section of the cuticle shows the pigment concentrated in the epicuticle, of a specimen of Baryconus with black-orange-black color (a) and black color (b). The variation in the distribution of the pigment together with the roughness of the cuticle surface can contribute to the reflection of light in different directions and therefore to the dispersion of the data. (PDF) [file pone.0218061.s004.pdf]
