# Supplementary material for: Spectral measure of color variation of black-orange-black (BOB) pattern in small parasitoid wasps (Hymenoptera: Scelionidae), a statistical approach
Source: PLoS One. 2019 Oct 24;14(10):e0218061. doi: 10.1371/journal.pone.0218061 (PMC6812806; doi:10.1371/journal.pone.0218061)

S2 Fig. Box plots for univariate mean differences per genus. Black dashed line represents the null hypothesis of no difference between colors per genus. *Acanthoscelio* (AC), *Baryconus* (BA), *Chromoteleia* (CR), *Macroteleia* (MA), *Opisthacantha* (OP), *Scelio* (SC), *Sceliomorpha* (SM), *Triteleia* (TR) and *Evaniella* (EV).

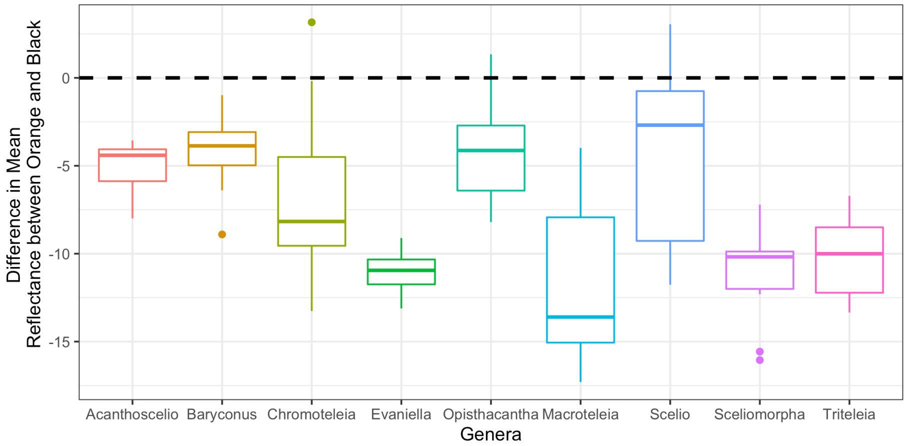

Supplement: S2 Fig — Black dashed line represents the null hypothesis of no difference between colors per genus. Acanthoscelio (AC), Baryconus (BA), Chromoteleia (CR), Macroteleia (MA), Opisthacantha (OP), Scelio (SC), Sceliomorpha (SM), Triteleia (TR) and Evaniella (EV). (PDF) [file pone.0218061.s005.pdf]
