# Supplementary material for: Spectral measure of color variation of black-orange-black (BOB) pattern in small parasitoid wasps (Hymenoptera: Scelionidae), a statistical approach
Source: PLoS One. 2019 Oct 24;14(10):e0218061. doi: 10.1371/journal.pone.0218061 (PMC6812806; doi:10.1371/journal.pone.0218061)
Supplement: S1 Table — The top 10 was extracted from a sample with overall minimum mean equal to 2.96 and maximum mean to 45.08 Acanthoscelio (AC), Baryconus (BA), Chromoteleia (CR), Macroteleia (MA), Opisthacantha (OP) and Sceliomorpha (SM). (PDF) [file pone.0218061.s006.pdf]

S1 Table. Top 10 $\overline{\Delta E}$ differences for comparisons of curves of different genera. The top 10 was extracted from a sample with overall minimum mean equal to 2.96 and maximum mean to 45.08 *Acanthoscelio* (AC), *Baryconus* (BA), *Chromoteleia* (CR), *Macroteleia* (MA), *Opisthacantha* (OP) and *Sceliomorpha* (SM).

	Genera 1	Genera 2	Color 1	Color 2	$\overline{\Delta E}$
1	CR	MA	BL	OR	45.08
2	MA	TR	OR	BL	44.79
3	MA	SM	OR	BL	43.59
4	LA	TR	OR	BL	41.51
5	CR	SM	BL	OR	41.25
6	SM	TR	OR	BL	41.22
7	MA	SC	OR	BL	41.02
8	CR	LA	BL	OR	40.84
9	BA	TR	OR	BL	39.69
10	AC	MA	BL	OR	39.58
