# Supplementary material for: Spectral measure of color variation of black-orange-black (BOB) pattern in small parasitoid wasps (Hymenoptera: Scelionidae), a statistical approach
Source: PLoS One. 2019 Oct 24;14(10):e0218061. doi: 10.1371/journal.pone.0218061 (PMC6812806; doi:10.1371/journal.pone.0218061)
Supplement: S2 Table — The top 10 was extracted from a sample with overall minimum mean equal to 2.96 and maximum mean to 45.08. Acanthoscelio (AC), Baryconus (BA), Chromoteleia (CR), Macroteleia (MA), Opisthacantha (OP), Scelio (SC), Sceliomorpha (SM) and Triteleia (TR). (PDF) [file pone.0218061.s007.pdf]

S2 Table. Top 10 $\overline{\Delta E}$ differences for comparisons of curves of the same genera. The top 10 was extracted from a sample with overall minimum mean equal to 2.96 and maximum mean to 45.08. *Acanthoscelio* (AC), *Baryconus* (BA), *Chromoteleia* (CR), *Macroteleia* (MA), *Opisthacantha* (OP), *Scelio* (SC), *Sceliomorpha* (SM) and *Triteleia* (TR).

	Genera 1	Genera 2	Color 1	Color 2	$\overline{\Delta E}$
1	SM	SM	BL	OR	39.71
2	MA	MA	BL	OR	34.59
3	SC	SC	BL	OR	34.36
4	BA	BA	BL	OR	34.11
5	CR	CR	BL	OR	32.02
6	LA	LA	BL	OR	30.26
7	TR	TR	BL	OR	29.57
8	AC	AC	BL	OR	20.37
9	LA	LA	OR	OR	10.40
10	LA	LA	BL	BL	10.28
