# Supplementary material for: Spectral measure of color variation of black-orange-black (BOB) pattern in small parasitoid wasps (Hymenoptera: Scelionidae), a statistical approach
Source: PLoS One. 2019 Oct 24;14(10):e0218061. doi: 10.1371/journal.pone.0218061 (PMC6812806; doi:10.1371/journal.pone.0218061)
Supplement: Attachment — Submitted filename: Austin1997.pdf [file pone.0218061.s008.pdf]

*Invertebrate Taxonomy*, 1997, **11**, 1–87

# The Ovipositor System of Scelionid and Platygastrid Wasps (Hymenoptera : Platygastroidea): Comparative Morphology and Phylogenetic Implications

*A. D. Austin and S. A. Field*

Department of Crop Protection, Waite Campus, The University of Adelaide, PO Glen Osmond, SA 5064, Australia.

*Abstract*

The morphology of the sclerotised components of the ovipositor system is comprehensively surveyed for scelionid and platygastrid wasps, with information being assessed for 120 genera and 220 species. A diagnosis for the ovipositor system is presented for most genera to complement existing generic descriptions. Two previously described and mechanically different forms of the ovipositor system are recognised: (1) the *Ceratobaeus*-type that is extended and retracted by antagonistic muscles and (2) the *Scelio*-type that is operated by changes in hydrostatic pressure, where the ovipositor is extended at the end of an elongate telescopic tube derived from expanded intersegmental membrane between metasomal segments 6 and 7. Comparison of these forms with the supposed ground plan for the Scelionidae strongly indicates that the *Scelio*-type is apomorphic, that it defines a monophyletic group associated with orthopteran host eggs, and that it comprises the tribes Scelionini, Calliscelionini, most Psilanteridini, Aradophagini, Neoscelionini, Platyscelionini, Doddiellini and four genera misplaced within the Sparasionini and Baryconini (*Archaeoteleia* Masner, *Bracalba* Dodd, *Chromoteleia* Ashmead and *Oxyscelio* Kieffer), as well as *Sceliacanthella* Dodd. Until a more robust classification of the superfamily is forthcoming, it is proposed that this group be informally referred to as the 'Scelionini *sensu lato*'. Further, seven genera (*Habroteleia* Kieffer, *Palpoteleia* Kieffer, *Anteris* Foerster, *Fusicornia* Risbec, *Leptoteleia* Kieffer, *Opisthacantha* Ashmead and *Styloteleia* Kieffer) are misplaced in the Calliscelionini and Psilanteridini because they possess the *Ceratobaeus*-type system. *Nixonia* Masner, *Sparasion* Latreille and *Sceliomorpha* Ashmead (Sparasionini) are considered to have the most primitive ovipositor system because they possess a *Ceratobaeus*-type system, and sub-basally fused lateral and latero-ventral apodemes, the latter being loosely attached to sternite 6. *Sparasion* and *Sceliomorpha* also have very short lateral apodemes and this, in conjunction with the form of the apodemes, can be considered to be the ground plan for the superfamily.

The Platygastridae all possess a modified ovipositor system but, nonetheless, one that in most cases is extended and retracted by musculature (i.e. *Ceratobaeus*-type). In particular, the system in most platygastrids is typified by having metasomal tergite 8 and associated cerci missing, the lateral apodemes short and forming a U-shape, and the ovipositor assembly generally robust. Only one of approximately 30 genera examined, *Acerotella* Masner, has very elongate apodemes, as in the Scelionidae. Many platygastrids also have a pair of latero-ventral apodemes, a presumed plesiomorphic character, rather than a single medial apodeme on sternite 6, which is the case for many Scelionidae. The most highly modified system is found in *Isostasius* Foerster and some *Synopeas* (*Sactogaster*) Foerster, where the ovipositor assembly is coiled vertically or partly so and the apodemes are greatly reduced. Generally, characters associated with the ovipositor system do not provide any independent support for the most recent higher-level classification of platygastrids, although they show substantial potential for more accurate definition of genera.

A preliminary cladistic analysis of 14 ovipositor characters supports the monophyly of five clades that correspond to the Scelionini *s. l.*, the Scelionidae (minus the Sparasionini *sensu stricto*), the Sparasionini *s. str.*, the Platygastridae, and the Sparasionini *s. str.*+Platygastridae. Overall, results from this study will provide baseline information on the ovipositor system as a prelude to a more complete phylogenetic analysis of the superfamily including external morphological characters. Although no new classification for the Scelionidae and Platygastridae is proposed, their higher-level taxonomy is reviewed and discussed and cases identified where, on the basis of ovipositor morphology, taxa (tribes and/or genera) apparently form monophyletic groups, and where taxa are misplaced. Finally, the status of the major higher-level groups within the superfamily is discussed, as is the available evidence to support their monophyly.

0818-0164/97/010001$30.00

**Introduction**

A common problem in attempting to develop a phylogenetic understanding for the various groups of parasitic Microhymenoptera is that many of the external morphological characters used in studies of larger wasps have either been greatly reduced or lost (i.e. reductional synapomorphies). This is particular so for many members of the Platygastroidea *sensu* Naumann (1991) and Masner (1993) (= Scelionoidea *sensu* Masner 1956; Richards and Davies 1977; Delvare and Aberlenc 1989; Masner and Huggert 1989), which are among the most simplified of all Hymenoptera. For example, they have very few tubular veins in the wings and no closed cells; the antennae are reduced to 10 or fewer flagellomeres; the sclerites comprising the mesosoma and metasoma are often fused, particularly in wingless forms; the surface is often smooth and sculptureless; and the genitalia, in both males and females, is completely internalised. Consequently, the current classification of the two included families, Scelionidae and Platygastridae, has relied heavily on reductional characters, and this has hampered the recognition of monophyletic groups. Undoubtedly, many reductional characters have evolved convergently: the fusion of mesosomal and metasomal sclerites, loss of wings, and fusiform appearance of genera and species that have invaded soil and litter habitats (e.g. scelionids – *Baeus* Haliday, *Dyscritobaeus* Perkins and *Trimorus* Foerster; platygastrids – *Parabaeus* Kieffer and *Platygastoides* Dodd; see Galloway and Austin 1984; Masner and Huggert 1989; Austin 1990), and loss of the fore wing submarginal vein in platygastrids (Masner and Huggert 1989).

However, the ovipositor system is less prone to reductions in complexity because it is vital to the process of host parasitisation for virtually all species of parasitic wasps (Gauld and Bolton 1988), and represents a functional unit comprising numerous components that phylogenetically can be divided into multiple independent characters. The potential of the ovipositor system as a character set of use in taxonomic and phylogenetic studies has been previously recognised in several groups of Microhymenoptera. For instance, anatomical studies have been undertaken for the Chalcidoidea (King 1962; King and Copland 1969; Copland and King 1971*a*, 1971*b*, 1972*a*, 1972*b*, 1972*c*; Copland *et al.* 1973; Copland 1976), Cynipoidea (Fergusson 1988), Ceraphronoidea (Masner and Dessart 1967), some proctotrupoid families (Naumann 1982; Mason 1983, 1984), and at a more general level for the whole order (Rasnitsyn 1988; Quicke *et al.* 1992, 1994). Ovipositor characters have been used to make preliminary phylogenetic inferences in some cases, although they are yet to be included in a parsimony-based phylogenetic analysis. Interestingly, at least some members of the above groups are of moderate size and have the ovipositor external or partly so, allowing for assessment of structural difference without requiring dissection of specimens. However, the reluctance of many workers to undertake time-consuming dissection of microwasps has meant that the potential of this character system has remained largely unrealised for groups that are very small in size and/or where the ovipositor is held internally.

This has been the case for all taxonomic studies on the Platygastroidea prior to Masner (1976). Earlier comprehensive studies (e.g. Ashmead 1893, 1901, 1903; Kieffer 1926; Dodd 1913, 1914, 1915, 1926; Szabó 1966; Kozlov 1970) exclusively refer to external morphological characters, and only mention the ovipositor system indirectly when describing the length of the metasomal horn, without recognising its function, that being a recess for a more elongate internally retracted ovipositor (Baumann 1938; Austin 1983; Galloway and Austin 1984). Masner (1976) was a landmark study in that it described for the first time some aspects of the ovipositor and its association with tergite 7, as one of several major character systems used to propose a substantially improved reclassification of the family. Subsequently, Austin (1983) and Field and Austin (1994) showed that scelionid wasps possess two unique and functionally different types of ovipositor system, one where the ovipositor is extended and retracted by antagonistic muscles and the other by changes in hydrostatic pressure (see below).

The phylogenetic importance of the ovipositor system in platygastroid wasps has been recently highlighted by Masner and Huggert (1989), Galloway *et al.* (1992), Field and Austin (1994) and Masner (1993) although its structure has been reported in detail for only 10 of the 150 or so recognised genera of Scelionidae, and for no members of the Platygastridae. Here, for the first time, the morphology of the ovipositor system is comprehensively surveyed for both

families, and in so doing information is presented on more than 80 genera of scelionids and 30 genera of platygastrids. These data serve to complement existing taxonomic diagnoses for genera, as well as provide baseline information on this character system as a prelude to a formal higher-level phylogenetic analysis of the superfamily. For this reason, detailed descriptions of the ovipositor system and associated structures are given for genera in each family. Although no new classification of the Scelionidae and Platygastridae is proposed here, we review their higher-level taxonomy and point out cases where, on the basis of ovipositor morphology, groups of tribes and/or genera apparently form monophyletic groups, and where particular genera are misplaced. Finally, the status of the major higher-level groups within the superfamily is discussed, as is the available evidence to support their monophyly.

## Materials and Methods

### *Dissections*

The metasoma of point-mounted (dried) or alcohol-preserved specimens were removed and cleared by placing them in warm 10% KOH for 1–6 h, which dissolved soft tissues and left only cuticular parts and sclerotised membranes. If the integument of the metasoma was particularly thick and/or hardened, some sclerites were removed to allow the KOH to enter the haemocoel and contact the tissues. Cleared specimens were rinsed in distilled water, covered in a drop of glycerine on a glass slide or excavated glass block and dissected under a stereomicroscope at high magnification with fine entomological pins. Visualisation of semi-transparent structures was facilitated by refracting light from a fibre-optics light source through the side of a glass block. By manipulating the light source, this created a dark-field effect and revealed fine structures that were otherwise difficult to see. Dissected specimens were then stored in glycerine-filled genitalia capsules for later examination.

### *Scanning Electron Microscopy*

Point-mounted (dried) or alcohol-preserved specimens were selected because they had the ovipositor system in various stages of extension. They were washed in concentrated soap solution (50%) to remove extraneous material, rinsed twice in distilled water (10–30 min each), critical-point dried in a CPD 750 critical-point drier before being mounted on cardboard points and coated. Clean specimens were coated directly with 30 nm of gold or platinum in an EMScope SC-250 sputter coater and examined in a Cambridge Stereoscan 250 (Mk 3B) scanning electron microscope at an accelerating voltage of 20 kV.

### *Phylogenetic Analysis*

Preliminary analysis of the character matrix presented in Table 2 was undertaken by PAUP 3.1.1 (Swofford 1993) and a Power MacIntosh 7500/100 computer, after the input of data into MacClade 3.02 (Maddison and Maddison 1992). The ancestral states discussed under Character Analysis (below) were used to code for a hypothetical ancestor and this was used as the reference taxon. The data were analysed as unordered and polarised, with the heuristic search option, and the simple addition sequence, TBR (tree bisection reconnection) branch swapping, and with one tree being saved at each step.

### *Material Examined*

As part of this project 90 genera and 169 species of Scelionidae and 34 genera and 51 species of Platygastridae have been examined (see the Appendix). Virtually all of this material was obtained by field collection by the senior author, from the collection in the Department of Crop Protection, University of Adelaide, or from Dr Lubomir Masner, Canadian National Collection. Where possible, more than one species of a genus was studied to determine whether any intrageneric variation in the ovipositor system existed. In all cases, except some generic clusters of Platygastridae, material of the 'type' genus for higher categories (subfamilies, tribes and generic clusters) was examined. Usually at least one and sometimes several specimens of each species were dissected. Some species were only examined externally (not dissected) when the ovipositor was fully or partly extended or where closely allied species had been studied in detail. Because of the similarity between many taxa not all genera are illustrated; for easy reference those represented by line drawing, electron micrograph or both are indicated in the Appendix.

## Terminology, Abbreviations and Classification

### *Terminology*

Four different nomenclatures are used in reference to insect ovipositor morphology (for summaries, see Smith 1970; Scudder 1971; Fergusson 1988). In this paper we have adopted that used by Scudder (1961*a*,

1961*b*, 1971) and followed by Austin (1983) and Field and Austin (1994), which is, in our opinion, functionally the most correct.

To assist in understanding the complexity of the component structures, the following terms used to describe the ovipositor system of scelionid and platygastrid genera are defined in some detail.

*Abdominal segments.* See under Metasomal segments.

*Abdominal segment 9 (A9).* Because the ovipositor system in the Platygastroidea is invaginated into the metasoma and at rest is held internally, profound changes have occurred to some elements, in particular abdominal segment 9 (A9). This segment is mostly represented by the tergal component, which consists of three parts, while the sternum is substantially reduced (Field and Austin 1994). The three parts of A9 are metasomal tergite 8 (T8), the visible sclerite at the posterior end of the metasoma, which in scelionids has a pair of cerci and T7 attached internally to its anterior margin (these tergites are often fused – T7+8); the dorsal component of the membranous tube surrounding the ovipositor (tubular T8), and the pair of arms attached to the proximal head of the ovipositor, called the *proximal arms of T8* (Fig. 4). The ventral component of the membranous tube is derived from S8, and the term *tubular A9* is used to refer to the membranous tube as a whole (Figs 6, 8, 11), as this accounts for both ventral (sternal) and dorsal (tergal) components.

*Apodemes.* These are internal sclerotised rod-like structures to which muscles are normally attached, and were first described for platygastroids in *Ceratobaeus* Ashmead and related genera (tribe Baeini) by Austin (1983). In this group the apodemes of tergal origin (T7+8) are paired and lateral to the ovipositor (Figs 7, 9) and hence are called *lateral apodemes*. Similarly, the apodeme of sternal origin (S6+7+8) runs along the midline, ventral to the ovipositor, and is termed the *medial apodeme* (Figs 7, 10). Examination of additional scelionid genera in the present study has shown this to be the most common arrangement in the family, but a second arrangement was also revealed. In some genera, the lateral apodemes of tergal origin are fused at some point with lateral apodemes of sternal origin (e.g. *Sparasion* Latreille, Fig. 14). To distinguish the latter, we have termed them *latero-ventral apodemes*. They are usually loosely attached to S6+7+8 by a membranous plate. In some genera (e.g. *Baryconus* Foerster, Fig. 29; *Leptoteleia* Kieffer, Fig. 58) this membranous plate extends all the way to the proximal head of the ovipositor, and is apparently the same structure as the ventral membranous plate listed below as a separate character. This arrangement of paired apodemes is also found in some platygastrids (e.g. *Platygastoides*, Fig. 140), but in some genera the lateral and latero-ventral apodemes are not connected, leaving two distinct pairs, one dorsal (of tergal origin) and one ventral (of sternal origin) (e.g. *Aphanomerus* Perkins, Figs 144, 145). Latero-ventral apodemes and the medial apodeme are never found together and they have probably evolved convergently (see below under Phylogenetic Implications).

Ceratobaeus-*type ovipositor system.* See under Overview.

*Gonoplacs.* Sheaths attached to the distal ends of the 2nd gonocoxae, which surround and protect the ovipositor and are extended from the metasoma at oviposition (Fig. 4).

*Latero-ventral apodemes.* See under Apodemes.

*Membranous plate.* See under Ventral membranous plate.

*Metasomal segments.* There exists the potential for confusion in the terminology used to refer to the abdominal segments in apocritan Hymenoptera. In this suborder, the 1st abdominal segment (A1), or propodeum, is fused to the thorax and thus the constriction, or 'waist', divides not the thorax and abdomen, but the mesosoma (thorax plus propodeum or A1) and metasoma (A2–A9). In order to be consistent with the taxonomic and anatomical literature on parasitic Hymenoptera, which has mostly standardised the use of the terms mesosoma and metasoma (the latter defined here as that part of the abdomen posterior to the propodeum), we refer to post-petiolar segments as metasomal segments and refer to abdominal segmentation (in parentheses) only where confusion may arise because of fusion of segments or homology. Thus, metasomal terga T1–T8 and sterna S1–S8 correspond to terga and sterna of abdominal segments 2–9 (A2–A9).

*Ovipositor.* Refers only to the shaft of the ovipositor, that is, the 1st and 2nd gonapophyses.

*Ovipositor assembly.* Mechanism consisting of the five primary elements of the ovipositor (see Fergusson 1988): 1st and 2nd gonapophyses, gonoplacs, 2nd gonocoxae and gonangulae, plus the proximal arms of T8, which comprise two sclerotised plates derived from tergum 8 and fused to the proximal part of the ovipositor assembly (Fig. 4; see also Field and Austin 1994). The length of the ovipositor assembly is measured from the most proximal point of the rami of the 1st gonapophyses to the distal tip of the ovipositor shaft.

*Ovipositor system.* All functional elements associated with oviposition, including the ovipositor assembly, tubular A9, telescopic tube, apodemes and associated tergites and sternites, but excluding the rest of the metasoma (i.e. T1–T6 and S1–S5). The type of ovipositor system is classified as either muscular (*Ceratobaeus*-type) or hydrostatic (*Scelio*-type) (see Overview below).

*Proximal arms.* Proximal arms of T8 (see under Abdominal segment 9).

Scelio-*type ovipositor system.* See under Overview.

*S6+7+8.* The most posterior metasomal sternite, comprising the fused 6th, 7th and 8th sterna (A7, A8 and A9), bearing either a medial apodeme (Figs 7, 10, 11) or latero-ventral apodemes (Fig. 14) internally. In some genera a suture line indicates the division between two of the three sternites involved (e.g. *Baryconus* Foerster, Fig. 28).

*Telescopic tube.* One or more sections of telescopic tube surrounding the ovipositor assembly and tubular A9 in genera with the *Scelio*-type ovipositor system (Figs 5, 6, 32-35; see Field and Austin 1994).

*Tubular A9.* The membranous tube surrounding the ovipositor (see above).

*T7+8.* The most posterior metasomal terga, comprising the mostly fused or partly fused 7th and 8th terga (i.e. A8 and A9), in scelionids usually bearing a pair of cerci externally on T8 and lateral apodemes internally on T7. In several genera a suture line indicates the division between these two tergites (e.g. *Anteris* Foerster, Fig. 49), while in some *Trimorus* they are distinct and separated (Figs 119, 120). Several genera that possess a *Scelio*-type ovipositor system have T7 partly separated from and overlying T8 (Field and Austin 1994). Platygastrids have T8 and cerci absent, and T7 forms a U-shaped connection between the lateral apodemes (Figs 148, 150). In a few genera (e.g. *Zelandonota* Masner & Huggert, Fig. 141) a small postero-medial membranous plate attached to the U-shaped connection may represent a remnant of T8.

*Ventral membranous plate.* Membranous plate ventral to the ovipositor assembly which is often visible proximally beyond the rami of the 1st gonapophyses (pva in Fig. 4). In some genera with a *Ceratobaeus*-type ovipositor system (e.g. *Baryconus*, Fig. 29; *Leptoteleia*, Fig. 58) it is large and clearly connected postero-medially to the lateral apodemes (see above). In others, it often appears as though the membranous plate is completely desclerotised except for its distal and proximal parts (Figs 4, 9). The distal part curves upwards and connects with the lateral apodemes and the lateral corners of T7 (dvp in Fig. 9; also see Figs 49, 96). Previously, the distal part of the ventral membranous plate has been referred to as part of T7 (Field and Austin 1994). In the *Scelio*-type ovipositor system much of the ventral membranous plate is absent or incorporated into the telescopic tube (as for the lateral apodemes), and the only portion retained is the proximal part visible above the rami of the 1st gonapophyses.

*2nd gonapophysis assembly.* Assembly at the proximal end of the 2nd gonapophysis (see Fig. 4). This may be complex, with two ovoid processes extending proximally (Fig. 4), or simple, lacking ovoid processes (e.g. *Trissolcus* Ashmead, Fig. 126), or absent (e.g. *Baeus*, Fig. 103).

*Abbreviations*

The following abbreviations are used in the text, apart from those that are given above under Terminology.

CNCI Collection of Insects, Arachnids and Nematodes, Research Branch, Agriculture Canada, Ottawa

WARI Insect collection in the Department of Crop Protection, University of Adelaide, Adelaide

*Classification*

The tribal and generic-level classification of the Scelionidae used here follows Masner (1976), but with the recent modifications of Masner (1980*b*), Galloway and Austin (1984), Austin (1988) and Galloway *et al.* (1992). The higher-level classification of the Platygastridae used follows Masner and Huggert (1989), but with the addition of two extra generic groupings, the *Platygaster*-group and *Synopeas*-group (*sensu* Muesebeck 1979), that contain those genera not included in Masner and Huggert (1989). In recent years, the higher-level classification of Scelionidae has achieved reasonable stability, as reflected in the work of Kozlov (1970), Masner (1976, 1979, 1980*b*) and Galloway and Austin (1984). However, for the Platygastridae the arrangement of subfamilies and tribes has been in a state of flux. This has culminated recently with the recognition by Masner and Huggert (1989) that the Inostemmatinae (*sensu* Kozlov 1970 and others) is a polyphyletic group and defined only by the plesiomorphic presence of a tubular submarginal vein. On the basis of a comprehensive revision of the genera previously accommodated in this subfamily, these authors have made substantial changes to the classification of platygastrids. Importantly, they have recognised only two subfamilies with extended limits (the Platygastrinae and Sceliotrachelinae), and have forsaken the use of tribes in favour of informal generic clusters. The latter change recognises that the relationships among platygastrid genera are often blurred by the small size and extreme reductional nature of many characters.

The treatment of tribes of Scelionidae are arranged here in the order in which they appear in Masner (1976) and Galloway and Austin (1984) so that results can be easily compared with these references. For the Platygastridae generic clusters (= tribes) are treated in the order in which they appear in the table given by Masner and Huggert (1989: 14). Within each tribe (Scelionidae) or generic cluster (Platygastridae) genera are arranged alphabetically but with the nominal genus first. In Table 1 and the Appendix genera are simply arranged alphabetically.

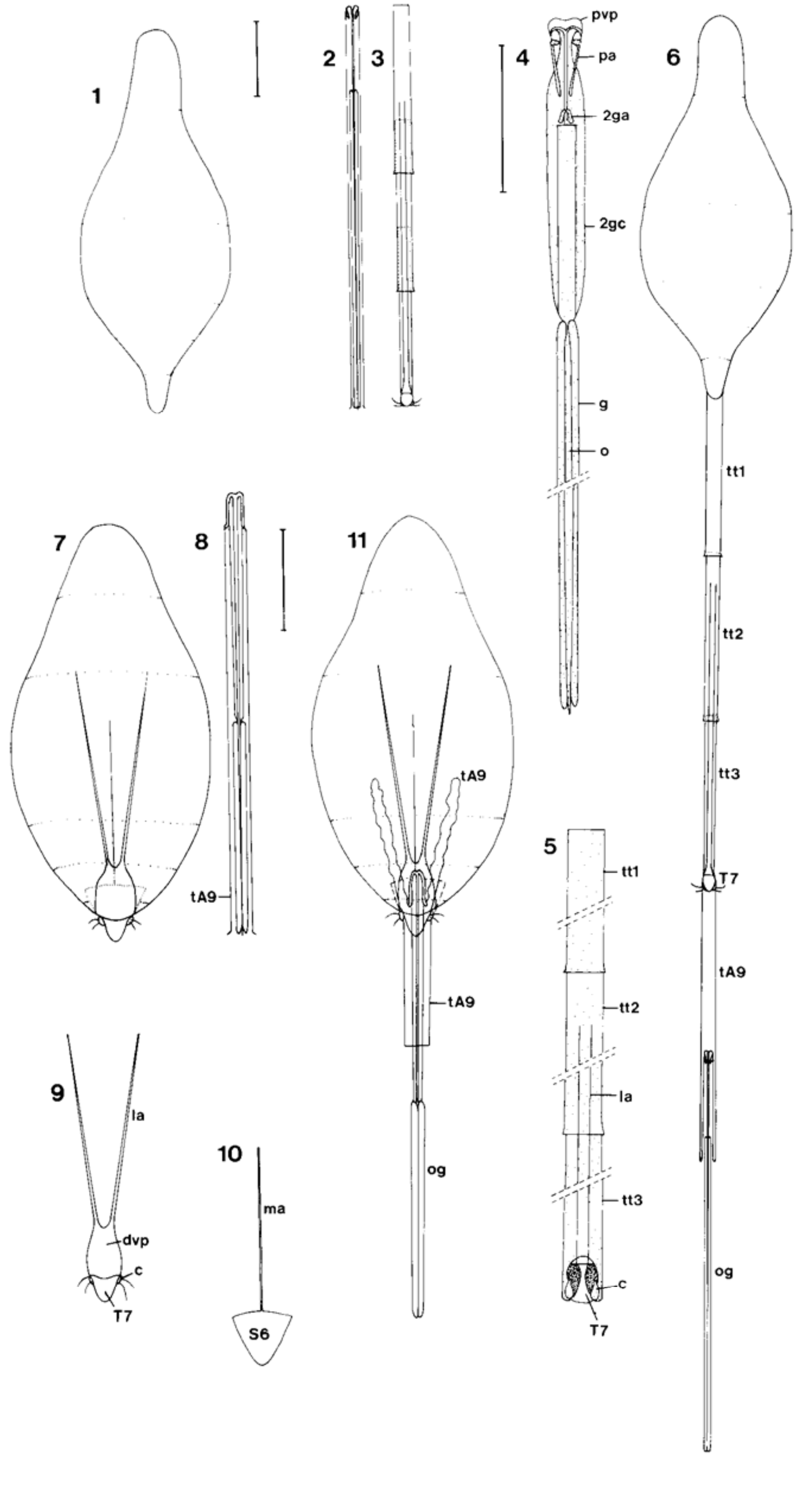
1
2
3
4
pvp
pa
2ga
2gc
g
o
6
tt1
tt2
tt3
T7
tA9
og
7
8
11
tA9
5
tt1
tt2
la
tt3
c
T7
9
la
dvp
c
T7
10
ma
S6

**Overview of the Platygastroid Ovipositor System**

The earliest report of the structure of the ovipositor in the Platygastroidea was for a species of *Probaryconus* Kieffer (Baumann 1938). Major taxonomic works (e.g. Ashmead 1893; Dodd 1926; Kieffer 1926; Kozlov 1970) never considered ovipositor morphology, until Masner (1976), in his reclassification of scelionid genera, listed in the diagnoses for tribes and some genera the relative position of T7 (T7+8 in this study), viz. 'T7 external and not extruded with ovipositor' or 'T7 internal and exserted with ovipositor'. This difference in the position of T7+8 has only become obvious since specimens have been collected in yellow pan traps containing concentrated salt solution (Dessart 1969). The salt acts as a short-term preservative and, as well, causes the ovipositor system to exsert itself from the metasoma, revealing an otherwise internal set of structures. In recognising that scelionids have two different arrangements for the position of T7+8, Masner (1976) laid the ground work for two recent studies (Austin 1983; Field and Austin 1994) that examined the mechanics of ovipositor extension and retraction in representative scelionid genera.

Austin (1983) and Field and Austin (1994) have shown that two functionally different types of ovipositor system exist within the Scelionidae. In both systems, the ovipositor assembly is generally similar in structure and is housed within an eversible membranous tube derived from abdominal segment 9 (i.e. tubular A9; Figs 6, 8, 11). In the first system, the *Ceratobaeus*-type, as it was first described for this genus (Austin 1983), the ovipositor assembly is extended and retracted by a set of antagonistic muscles attached to elongate apodemes protruding internally from T7+8 and S6+7+8 (Figs 7, 9, 10). It may also be assisted by changes in hydrostatic pressure (Austin 1983). In this system the proximal end of the ovipositor assembly stays within the distal end of the metasoma and tubular A9 collapses, also usually remaining inside the metasoma although it is sometimes partially extruded (Fig 11). The *Ceratobaeus*-type system has been described for several other genera in the tribes Baeini (Austin 1983) and Gryonini (Galloway *et al.* 1992).

In the second system, referred to as the *Scelio*-type (Field and Austin 1994), the mechanics of ovipositor extension is fundamentally different in that a telescopic tube with multiple sections (Figs 5, 6) extends the ovipositor and tubular A9 (Fig. 6) away from the metasoma by changes in hydrostatic pressure. The telescopic tube, which is derived from intersegmental membrane between metasomal segments 6 and 7, thus carries T7+8 and the ovipositor away from the distal end of the metasoma (Fig. 6), and effectively increases the distance to which the ovipositor can reach by two to three times that of the *Ceratobaeus*-type system. In the *Scelio*-type system there is no associated musculature, the medial (sternal) apodeme is absent, and the lateral apodemes are incorporated into the wall of the telescopic tube and probably serve to strengthen it (Field and Austin 1994). Tubular A9 is everted in a fashion similar to that of the *Ceratobaeus*-type system. Masner (1993), following comments in Austin (1983), has postulated that the changes in hydrostatic pressure responsible for ovipositor extension and contraction are

---

**Figs 1–6.** Components of the ovipositor system and their arrangement in *Calliscelio* sp. (Calliscelionini), an example of the *Scelio*-type ovipositor system: *1*, dorsal view of metasoma showing tergites 1–6; *2*, ovipositor assembly and tubular A9 removed from inside the telescopic tube (Fig. 3); *3*, unextended telescopic tube and tergites 7+8 removed from metasoma (Fig. 1); *4*, detailed view of ovipositor assembly (from Fig. 2); *5*, detailed view of unextended telescopic tube and tergites 7+8 (from Fig. 3); 6, fully extended ovipositor system. **Figs 7–11.** Components of the ovipositor and their arrangement in *Ceratobaeus masneri* Austin (Baeini), an example of the *Ceratobaeus*-type ovipositor system: *7*, metasoma showing position of lateral and medial apodemes; *8*, ovipositor assembly and tubular A9 removed from metasoma (Fig. 7); *9*, tergites 7+8 and lateral apodemes removed from metasoma (Fig. 7); *10*, sternites 6+7+8 and medial apodeme removed from metasoma (Fig. 7); *11*, fully extended ovipositor system. Scale lines: *1–3*, *6*, 0·5 mm; *4*, *5*, 250 μm; *7–11*, 250 μm. c, cercus; dvp, distal part of ventral membranous plate; g, gonoplac; la, lateral apodeme; ma, medial apodeme; o, ovipositor; og, ovipositor+gonoplacs; pa, proximal arms; pvp, proximal part of ventral membranous plate; S6, metasomal sternites 6+7+8; tA9, tubular part of abdominal segment 9; tt, telescopic tube (sections 1–3); T7, metasomal tergites 7+8; 2ga, 2nd gonapophyses assembly; 2gc, 2nd gonocoxae.

'created by pumping movements of the head and propleuron into the metasoma and by the bellows-like action of the metasomal tergites and sternites'. For *Scelio* Latreille, muscles have been identified connecting tergites and sternites that could produce such movement of the metasoma, but behavioural observations have shown that head and propleuron movements do not occur in this genus (Field and Austin 1994), whereas they do in *Ceratobaeus* (Austin 1983).

The *Scelio*-system has been reported for two other genera in the Scelionini and three genera in each of the Calliscelionini and Psilanteridini. However, Masner's (1976) description of T7+8 as being internal for various taxa indicates that this system could be universal for several tribes, although his report of 'T7 fused to T6' for the Scelionini was made prior to the realisation that these structures must be dissected to reveal their true arrangement.

The ovipositor system of the Platygastridae has virtually not been examined (see Masner and Huggert 1989). Our preliminary observations indicate that members of this family possess an ovipositor system that is mostly the same in structure (and presumably in function) as the *Ceratobaeus*-type. The main distinguishing features of the platygastrid system are the loss or reduction and subsequent internalisation of T7+8, and differences in the structure and arrangement of the dorsal and ventral apodemes.

## Ovipositor System of the Scelionidae

Relatively complete diagnoses of the ovipositor systems of each genus are presented here to supplement previously published descriptions based on external morphology. Discussion of the differences between genera, apparent relationships, and other notes are provided in the Comments sections at the end of each tribe (for scelionids) and generic cluster (for platygastrids). Character descriptions prefaced by a question-mark in the diagnoses indicate that the character was not properly revealed during dissections and that there is some uncertainty regarding its state. The numerical ranges for lengths of various characters are given below under Character Discussion. Qualitative descriptors for the length of characters (e.g. 'short', 'moderately elongate' and so on) are only given without accompanying numerical valves when accurate measurements could not be made because of the state of a dissection.

### Subfamily **Scelioninae**

### Tribe **Nixoniini** Masner

### Genus ***Nixonia*** Masner

(Figs 12, 13, 18, 19)

*Diagnosis*

Ovipositor assembly extended and retracted by muscles (i.e. *Ceratobaeus*-type system); ovipositor moderately elongate, about 0·7× length of metasoma; proximal part of ventral membranous plate present; 2nd gonapophyses assembly complex; gonoplacs elongate, about 0·65× length of ovipositor, slightly sinuate; proximal arms continuous with tubular A9, short, about 0·1× length of ovipositor or less; T7+8 completely fused, moderately short, rounded posteriorly, only lightly sclerotised, attached to T6 but partly contracted underneath it at rest, exposed during ovipositor extension; cercal hairs present but cerci indistinct; lateral apodemes fused with short latero-ventral apodemes, moderately elongate, length including T7+8 about 0·7× length of ovipositor; distal part of ventral membranous plate attached to lateral apodemes; latero-ventral apodemes only loosely attached to S6; S6 without medial apodeme.

**Figs 12, 13.** *Nixonia* sp. (Nixoniini): *12*, ovipositor assembly; *13*, tergites 7+8 with lateral and latero-ventral apodemes connected. **Figs 14, 15.** *Sparasion* sp. (Sparasionini): *14*, tergites 7+8 with lateral and latero-ventral apodemes connected; *15*, ovipositor assembly. **Figs 16, 17.** *Archaeoteleia mellea* Masner (Sparasionini): *16*, ovipositor assembly; *17*, unextended telescopic tube with tergites 7+8. Scale lines: *12–15*, 250 µm; *16, 17*, 250 µm. la, lateral apodeme; lva, latero-ventral apodeme; T7, metasomal tergites 7+8.

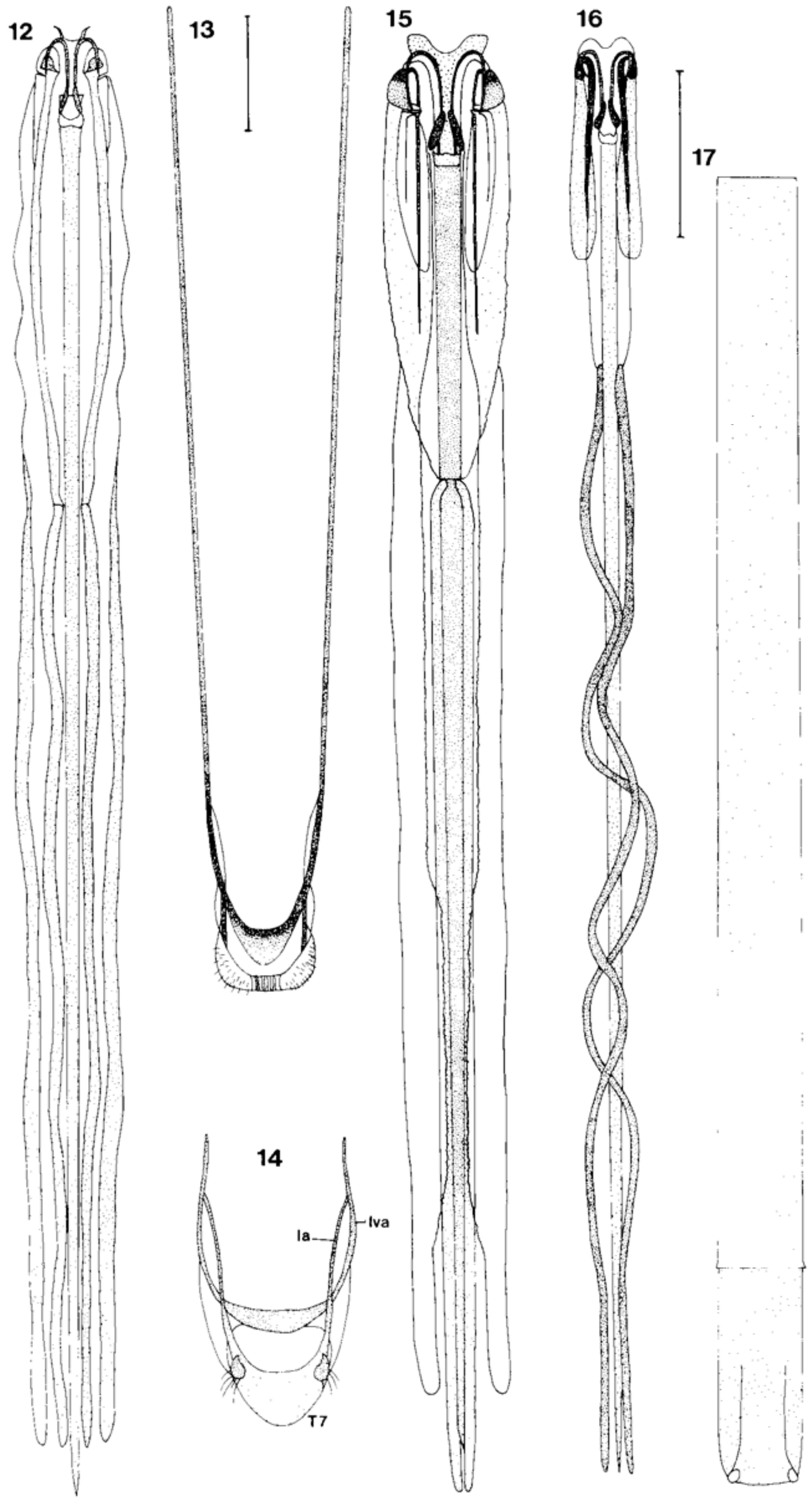
12
13
15
16
17
14
Ia
Iva
T7

*Comments on Nixoniini*

*Nixonia* is considered to be the most primitive living scelionid (Masner 1976) largely because of its larger number of antennal and palpal segments, 1-2-2 tibial spur formula, and more complete wing venation. Along with *Sparasion* and *Sceliomorpha* Ashmead (Sparasionini), it is the only genus to have the proximal arms joined to tubular A9. These genera also have the lateral apodemes fused sub-basally with latero-ventral apodemes, T7+8 at least partially desclerotised and hidden under T6 at rest and S6 without a medial apodeme, character states that are rare but not unique to these genera (see below under Phylogenetic Implications). *Nixonia* differs from *Sparasion* and *Sceliomorpha* primarily in having moderately elongate lateral apodemes, and T7+8 very short and rounded posteriorly (not triangular). The ovipositor system of *Nixonia* requires further study to determine the morphology of the cercal area and details of the juxtaposition of the distal terga and sterna; however, the genus is moderately rare and is known from few museum collections.

Tribe **Sparasionini** Masner

Genus ***Sparasion*** Latreille

(Figs 14, 15)

*Diagnosis*

Ovipositor assembly extended and retracted by muscles (i.e. *Ceratobaeus*-type system); ovipositor elongate, about 0·9× length of metasoma; proximal part of ventral membranous plate present; gonoplacs elongate, about 0·7× length of ovipositor; 2nd gonapophyses assembly complex; proximal arms slender, short, about 0·1× length of ovipositor, continuous with membranous extensions that reach almost to distal end of ovipositor; T7+8 fused but often distinct from each other, lightly to moderately sclerotised, attached to T6 but partly contracted underneath it at rest, exposed during ovipositor extension; cerci present and leaf-shaped; lateral apodemes very short, fused with latero-ventral apodemes, length including T7+8 about 0·2× length of ovipositor, distal part of ventral membranous plate attached to lateral apodemes; latero-ventral apodemes only loosely attached to S6; S6 without medial apodeme.

Genus ***Archaeoteleia*** Masner

(Figs 16, 17, 20–23)

*Diagnosis*

Ovipositor assembly and telescopic tube extended and retracted by hydrostatic pressure (i.e. *Scelio*-type system); telescopic tube with 2 sections, moderately robust, proximal section with fine longitudinal striations; ovipositor elongate to extremely elongate, sometimes extending into horn of T1 and thus longer than metasoma, 0·9–1·1× length of metasoma; proximal part of ventral membranous plate present; gonoplacs elongate, about 0·75× length of ovipositor, strongly sinuate, without proximal spines and serrated margin; proximal arms slender, moderately elongate; 2nd gonapophyses assembly complex; proximal arms slender, short, 0·12× length of ovipositor; T7+8 indistinct, no more sclerotised than distal part of telescopic tube, cerci present and leaf-shaped; lateral apodemes incorporated into wall of telescopic tube and distinct only in about distal one-fifth; S6 without medial apodeme.

*Comments on Sparasionini*

Like *Nixonia*, *Sparasion* and *Sceliomorpha* have a primitive number of palpal segments and tibial spur formula, but in other ways are similar to the Scelionini (Masner 1976). These three genera also have the proximal arms joined to tubular A9, the lateral apodemes fused sub-basally to latero-ventral apodemes (the latter of sternal origin), T7+8 at least partially desclerotised and hidden under T6 at rest, and S6 without a medial apodeme. *Sparasion* and *Sceliomorpha*

(Fig. 30) are identical in most respects, in particular in having very short lateral apodemes (about 0·15× length of elongate ovipositor). This character state is unique among the Scelionidae (although it occurs in a different form in *Neuroscelio* Dodd, Fig. 93), and is thought to be plesiomorphic. Very short lateral apodemes, in conjunction with their sub-basal attachment to the latero-ventral apodemes, are considered here to represents the ground plan for the superfamily (see below under Phylogenetic Implications). These genera differ primarily in *Sceliomorpha* having T8 membranous and indistinct and the cerci more tubular in shape (not leaf-like).

*Archaeoteleia*, as do the other two genera in this tribe, retains many primitive characters (e.g. palpal segment number, tibial spur formula, traces of more complete wing venation). It was described as an archaic member of the Scelionidae with a restricted Gondwanian distribution (Masner 1968*a*). However, this genus possesses a *Scelio*-type ovipositor system and clearly, because of this, it is misplaced in the tribe Sparasionini. It is better interpreted as a primitive member of a group including the Scelionini, Calliscelionini and Psilanteridini (Field and Austin 1994), which all have the more derived hydrostatically extended ovipositor system. However, because of its otherwise primitive suite of characters (see Masner 1968*a*, 1976) *Archaeoteleia* probably warrants tribal status in its own right.

### Tribe **Scelionini** Foerster

### Genus ***Scelio*** Latreille

(Figs 24, 25; Field and Austin 1994, figs 10, 13–16, 19–28)

*Diagnosis*

Ovipositor assembly and telescopic tube extended and retracted by hydrostatic pressure (i.e. *Scelio*-type system); telescopic tube with 2 or 3 sections; ovipositor elongate, 0·8–0·9× length of metasoma; proximal part of ventral membranous plate absent; gonoplacs moderately elongate, 0·55–0·60× length of ovipositor, curved ventrally at proximal end, with small spines at proximal margin and serrated margins along remaining proximal two-thirds; 2nd gonapophyses assembly complex; proximal arms slender, short, 0·1× length of ovipositor or shorter; T7+8 no more sclerotised than distal part of telescopic tube, posterior margin with a few setae; cerci present and lobe- to leaf-shaped; lateral apodemes incorporated into wall of telescopic tube and distinct throughout, about 0·75× length of ovipositor; S6 without medial apodeme.

*Comments on Scelionini*

Field and Austin (1994) have described the telescopic ovipositor system of *Scelio* in detail (after which this system is named), and have shown from dissections, histological examination, behavioural observations and manipulative experiments that this type of ovipositor system is extended and retracted by changes in hydrostatic pressure, not direct muscular contraction. Further, in this system T7+8 is invariably hidden beneath T6 at rest and is only seen when the telescopic tube is extended.

Members of the Scelionini represent a tightly knit group of genera that, among other characters, are defined by an incomplete submarginal vein in the hind wing and a restricted host range, acridid grasshoppers (Table 1). The ovipositor system of the tribe shows a similar trend in that it varies at most only slightly among genera. *Acanthoscelio* Ashmead, *Oreiscelio* Kieffer and *Heptascelio* Kieffer do not have spines on the proximal apex of the gonoplacs; *Acanthoscelio* and *Oreiscelio* have moderately elongate proximal arms (about 0·2–0·25× length of ovipositor); *Heptascelio* (Fig. 26), *Pseudoheptascelio* Szabó and *Synoditella* Muesebeck, like *Scelio*, have moderately elongate lateral apodemes, although in the latter two genera they are sometimes faint. *Heptascelio* differs from all other genera in that the lateral apodemes are apparently discontinuous at about one-fifth their length from T7+8, where they are truncated and connected by a U-shaped process (Fig. 26). Although T7+8 is slightly more sclerotised in most other genera than in *Scelio*, in *Dicroscelio* Kieffer it is particularly heavily sclerotised, sharply triangular and clearly differentiated from the surrounding telescopic tube. Apparent differences among genera in the size, shape and position of the cerci require confirmation from

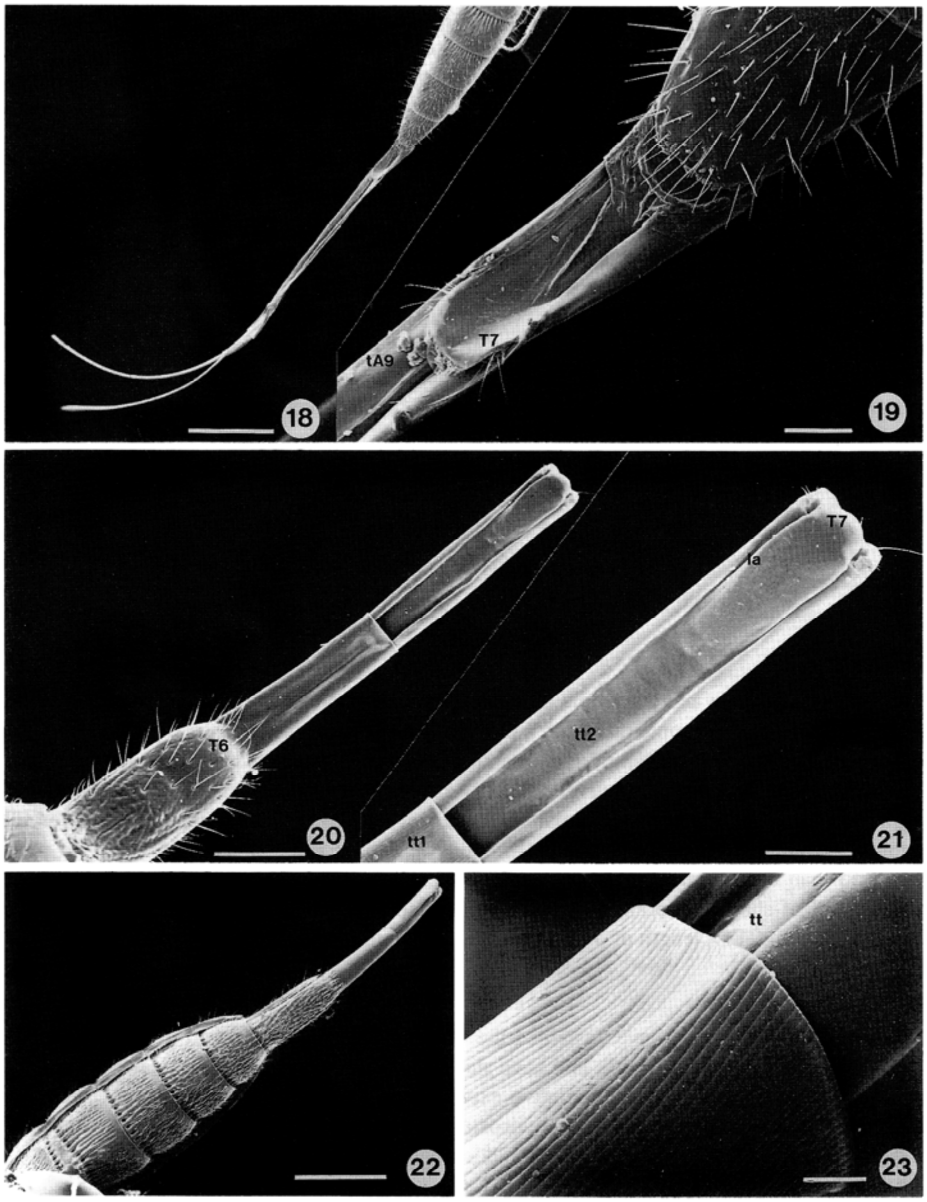


**Figs 18, 19.** *Nixonia* sp. (Nixoniini): *18*, fully extended ovipositor system; *19*, tergites 7+8 with cerci present postero-laterally. **Figs 20–23.** *Archaeoteleia mellea* Masner (Sparasionini): *20*, T6 and partially extended telescopic tube; *21*, last section of telescopic tube with dorsal ridges and tergites 7+8; *22*, ventral view of metasoma and partially extended ovipositor system (i.e. about 25% extended); *23*, microsculpure at distal end of first section of telescopic tube. Scale lines: *18*, 1·0 mm; *19, 21*, 100 µm; *20*, 200 µm; *22*, 0·5 mm; *23*, 20 µm. la, lateral apodeme; tA9, tubular part of abdominal segment 9; T6, metasomal tergite 6; T7, metasomal tergite 7+8; tt, telescopic tube (sections 1 and 2).

more detailed studies and consequently they are not described here. All genera in the Scelionini have an elongate ovipositor (0·8–0·9× length of metasoma), 2 or 3 sections in the telescopic tube, the proximal part of the ventral membranous plate absent, and short to moderately elongate gonoplacs (0·5–0·6× length of ovipositor).

**Table 1. List of scelionid and platygastrid genera with main host groups and the source of information**

Only host data from rearing records or behavioural observations (e.g. phoresy) are included, not that assumed from phylogenetic association. The higher classification follows Masner (1976), Galloway and Austin (1984) and Masner and Huggert (1989). Genera of unknown taxonomic status are not included nor are synonyms unless host data are published under that name (see Discussion for further details)

| Genus | Host group | Source |
|---|---|---|
| **SCELIONIDAE** | | |
| **Scelioninae** | | |
| **Nixoniini** | | |
| *Nixonia* Masner | unknown | Masner (1976) |
| **Sparasionini** | | |
| *Archaeoteleia* Masner | Rhaphidophoridae | L. Masner, personal communication |
| *Sceliomorpha* Ashmead | unknown | – |
| *Sparasion* Latreille | Tettigoniidae: Decticinae | Muesebeck (1979) |
| **Scelionini** | | |
| *Acanthoscelio* Ashmead | unknown | – |
| *Dicroscelio* Kieffer | unknown | – |
| *Freniger* Szabó | unknown | – |
| *Heptascelio* Kieffer | unknown | Masner (1976) |
| *Lepidoscelio* Kieffer | unknown | – |
| *Oreiscelio* Kieffer | unknown | – |
| *Pseudoheptascelio* Szabó (=*Tanaoscelio* Masner) | Acrididae | Masner (1972*b*) |
| *Scelio* Latreille | Acrididae | Muesebeck (1972) |
| *Sceliocerdo* Muesebeck | Acrididae | Muesebeck (1972) |
| *Synoditella* Muesebeck | Acrididae | Muesebeck (1972) |
| **Baryconini** | | |
| *Anthonyon* Huggert & Masner | unknown | Huggert and Masner (1983) |
| *Apegus* Foerster | unknown | – |
| *Baryconus* Foerster | Tettigoniidae: Phaneroptinae | Muesebeck (1979); Bin (1976) |
| *Bracalba* Dodd | unknown | – |
| *Chromoteleia* Ashmead | unknown | – |
| *Oxyscelio* Kieffer | unknown | – |
| **Calliscelionini** | | |
| *Alloteleia* Kieffer | unknown | – |
| *Amblyscelio* Kieffer | unknown | – |
| *Anteromorpha* Dodd | Gryllidae | L. Masner, personal communication |
| *Calliscelio* Ashmead | Gryllidae | Severin (1935) |
| *Crama* Galloway | unknown | – |
| *Dichoteleas* Kieffer | unknown | – |
| *Habroteleia* Kieffer | unknown | – |
| *Holoteleia* Kieffer | unknown | – |
| *Jarabambius* Galloway | unknown | – |
| *Lispoteleia* Galloway | unknown | – |
| *Macroteleia* Westwood | Tettigoniidae | Muesebeck (1977) |
| *Marshalliella* Kieffer | unknown | – |
| *Monoteleia* Kieffer | unknown | – |
| *Neoparidris* Galloway | unknown | – |
| *Oethecoctonus* Ashmead | Gryllidae: Oecanthinae | Masner (1983*a*) |
| *Palpoteleia* Kieffer | unknown | – |
| *Paridris* Kieffer | unknown | – |
| *Phaedrotelia* Kieffer | unknown | – |
| *Phoenoteleia* Kieffer | unknown | – |
| *Probaryconus* Kieffer | Gryllidae | Galloway and Austin (1984) |
| *Triteleia* Kieffer | unknown | – |
| *Yunkara* Galloway | unknown | – |

**Table 1.** continued

| Genus | Host group | Source |
|---|---|---|
| **Psilanteridini** | | |
| *Anteris* Foerster | unknown | – |
| *Calotelea* Westwood | unknown | Masner (1980*a*) |
| *Duarina* Dodd | unknown | – |
| *Duta* Nixon | Gryllidae | Masner (1991) |
| *Fusicornia* Risbec | unknown | – |
| *Genatropis* Galloway | unknown | – |
| *Harringtonia* Masner | unknown | Masner (1980*b*) |
| *Lapithoides* Nixon | unknown | – |
| *Leptoteleia* Kieffer | Gryllidae | Masner (1978) |
| *Lidgbirdius* Galloway | unknown | – |
| *Mallateleia* Dodd | unknown | – |
| *Mecix* Masner | unknown | Masner (1980*b*) |
| *Merriwa* Dodd | unknown | – |
| *Nyleta* Dodd | unknown | – |
| *Opisthacantha* Ashmead | unknown | – |
| *Oxyteleia* Kieffer | unknown | – |
| *Paraduta* Szabó | unknown | – |
| *Psilanteris* Kieffer | unknown | – |
| *Shreemana* Nixon | unknown | – |
| *Spiniteleia* Masner | unknown | Masner (1980*b*) |
| *Stenotelea* Huggert & Masner | unknown | Huggert and Masner (1983) |
| *Styloteleia* Kieffer | unknown | – |
| *Thoronella* Masner | ?Odonata | Carlow (1992) |
| *Trichoteleia* Kieffer | unknown | – |
| **Aradophagini** | | |
| *Aradophagus* Ashmead | unknown | García and Masner (1994) |
| **Neoscelionini** | | |
| *Neoscelio* Dodd | unknown | Galloway and Austin (1984) |
| **Cremastobaeini** | | |
| *Cremastobaeus* Ashmead | ?Gryllidae | I. D. Gauld, personal communication |
| **Mantibariini** | | |
| *Mantibaria* Kirby | Mantidae | Chopard (1923) |
| **Platyscelionini** | | |
| *Platyscelio* Kieffer | Orthoptera (? Tettigoniidae: Phaneropterinae) | Masner (1976) |
| **Doddiellini** | | |
| *Doddiella* Kieffer | unknown | Masner (1976) |
| **Gryonini** | | |
| *Breviscelio* Sundholm | unknown | Caleca (1992) |
| *Dyscritobaeus* Perkins (= Mirotelenomus Dodd) | unknown | Galloway and Austin (1984) |
| *Encrytoscelio* Dodd | Cydnidae | Bin (1979) |
| *Epigryon* Masner | Phymatidae | Masner (1980*b*) |
| *Eremioscelio* Priesner | Mantidae | Masner (1976) |
| *Gryon* Haliday | Alydidae, Coreidae, Largidae, Lygaeidae, Pentatomidae, Reduviidae | Masner (1983*b*) |
| *Hungarogryon* Szabó | unknown | – |
| *Maruzza* Mineo | Coreidae, Pentatomidae | Mineo (1982) |
| *Neuroscelio* Galloway, Austin & Masner | unknown | Galloway *et al.* (1992) |
| *Platyscelidris* Szabó | unknown | – |
| *Plesiobaeus* Kieffer | unknown | – |
| *Sundholmia* Szabó | unknown | – |

**Table 1.** continued

| Genus | Host group | Source |
|---|---|---|
| **Embidobiini** | | |
| *Echthrodesis* Masner | Araneae | Masner (1968*b*) |
| *Embidobia* Ashmead | Embioptera | Dodd (1939) |
| *Embioctonus* Masner | Embioptera | Masner (1980*b*) |
| *Endecascelio* Masner & Dessart | unknown | Masner and Dessart (1972) |
| *Palaeogryon* Masner | Embioptera | Masner (1976) |
| **Thoronini** | | |
| *Microthoron* Masner | unknown | Masner (1972*a*) |
| *Neothoron* Masner | unknown | Masner (1972*a*) |
| *Pseudanteris* Fouts | ?Gerridae | Masner (1972*a*) |
| *Tanaodytes* Masner | unknown | Masner (1972*a*) |
| *Thoron* Haliday | Nepidae | Masner (1972*a*) |
| *Thoronidea* Masner & Huggert | unknown | Masner and Huggert (1979*a*) |
| *Tiphodytes* Bradley | Gerridae | Masner (1972*a*) |
| **Baeini** | | |
| *Aneurobaeus* Kieffer | unknown | – |
| *Angolobaeus* Kozlov | unknown | Kozlov (1970) |
| *Apobaeus* Masner | unknown | – |
| *Baeus* Haliday | Araneae | Austin (1985) |
| *Ceratobaeus* Ashmead | Araneae | Austin (1985) |
| *Cyphacolus* Priesner | Araneae | A. D. Austin, unpublished data |
| *Hickmanella* Austin | Araneae | Austin (1985) |
| *Idris* Foerster | Araneae | Austin (1985) |
| *Mirobaeoides* Dodd | Araneae | Austin (1985) |
| *Mirobaeus* Dodd | Araneae | Austin (1985) |
| *Neobaeus* Austin | unknown | Austin (1988) |
| *Odontacolus* Kieffer | Araneae | Austin (1985) |
| **Unplaced Scelioninae** | | |
| *Parascelio* Dodd | unknown | – |
| *Sceliacanthella* Dodd | unknown | – |
| **Teleasinae** | | |
| **Teleasini** | | |
| *Ceratoteleas* Kozlov | unknown | – |
| *Echinoteleas* Risbec | unknown | – |
| *Gryonella* Dodd | unknown | – |
| *Odontoscelio* Kieffer | unknown | – |
| *Proteleas* Kozlov | unknown | – |
| *Scutelliteleas* Szabó | unknown | – |
| *Teleas* Latreille | unknown | – |
| *Trisacantha* Ashmead | unknown | Muesebeck (1979) |
| *Trimorus* Foerster | Carabidae | Muesebeck (1979) |
| **Xenomerini** | | |
| *Gryonoides* Dodd | unknown | – |
| *Xenomerus* Walker | Carabidae | A. D. Austin, unpublished data |
| **Telenominae** | | |
| **Telenomini** | | |
| *Aradoctonus* Masner | Aradidae | Masner (1980*b*) |
| *Eumicrosoma* Gahan | Lygaeidae | Dicke (1937) |
| *Latonius* Kononova | unknown | – |
| *Mudigere* Johnson | unknown | – |
| *Nirupana* Nixon | unknown | – |
| *Paratelenomus* Dodd (= Archiphanurus Szabó) | Plataspidae | Johnson (1985) |
| *Phanuromyia* Dodd | unknown | – |

**Table 1.** continued

| Genus | Host group | Source |
| --- | --- | --- |
| *Phanuropsis* Girault | Pentatomidae | Eberhard (1975); Johnson (1987) |
| *Phlebiaporus* Kozlov | unknown | – |
| *Protelenomus* Kieffer | unknown | – |
| *Psix* Kozlov & Le | Coreidae, Pentatomidae, Scutelleridae | Johnson and Masner (1985) |
| *Telenomus* Haliday | various Heteroptera and Lepidoptera (rarely Neuroptera and Diptera) | Johnson (1984*a*); Johnson and Bin (1982) |
| *Trissolcus* Ashmead | Pentatomoidea (mostly Pentatomidae) | Johnson (1984*b*) |
| **PLATYGASTRIDAE** | | |
| **Sceliotrachelinae** | | |
| ***Fidiobia*-cluster** | | |
| *Fidiobia* Ashmead | Chrysomelidae and Curculionidae | Masner and Huggert (1989) |
| *Neobia* Masner & Huggert | unknown | Masner and Huggert (1989) |
| *Oligomerella* Masner & Huggert | unknown | Masner and Huggert (1989) |
| *Platygastoides* Dodd | unknown | Masner and Huggert (1989) |
| *Platystasius* Nixon | Cerambycidae | Nixon (1937) |
| *Plutomerus* Masner & Huggert | unknown | Masner and Huggert (1989) |
| *Zelamerus* Masner & Huggert | unknown | Masner and Huggert (1989) |
| *Zelandonota* Masner & Huggert | unknown | Masner and Huggert (1989) |
| ***Aphanomerus*-cluster** | | |
| *Aphanomerella* Dodd | Chrysomelidae | Masner and Huggert (1989) |
| *Aphanomerus* Perkins | Flatidae, Ricaniidae | Masner and Huggert (1989) |
| *Austromerus* Masner & Huggert | unknown | Masner and Huggert (1989) |
| *Calomerella* Masner & Huggert | unknown | Masner and Huggert (1989) |
| *Helava* Masner & Huggert | ?Aphididae | Masner and Huggert (1989) |
| *Parabaeus* Kieffer | unknown | Austin (1990) |
| *Pseudaphanomerus* Szelényi | unknown | Masner and Huggert (1989) |
| *Tetrabaeus* Kieffer | Sphecidae: Crabroninae | Krombein (1964) |
| ***Isolia*-cluster** | | |
| *Afrisolia* Masner & Huggert | unknown | Masner and Huggert (1989) |
| *Isolia* Foerster | unknown | Masner and Huggert (1989) |
| *Pulchrisolia* Szabó | unknown | Masner and Huggert (1989) |
| *Sceliotrachelus* Brues | unknown | Masner and Huggert (1989) |
| ***Amitus*-cluster** | | |
| *Aleyroctonus* Masner & Huggert | Aleyrodidae | Masner and Huggert (1989) |
| *Alfredella* Masner & Huggert | unknown | Masner and Huggert (1989) |
| *Amitus* Haldeman | Aleyrodidae | Masner and Huggert (1989) |
| *Nanomerus* Masner & Huggert | unknown | Masner and Huggert (1989) |
| ***Allotropa*-cluster** | | |
| *Allotropa* Foerster | Pseudococcidae | Masner and Huggert (1989) |
| *Errolium* Masner & Huggert | Coccidae | Masner and Huggert (1989) |
| **Platygastrinae** | | |
| ***Proplatygaster*-cluster** | | |
| *Almargella* Masner & Huggert | unknown | Masner and Huggert (1989) |
| *Magellanium* Masner & Huggert | unknown | Masner and Huggert (1989) |
| *Orseta* Masner & Huggert | unknown | Masner and Huggert (1989 |
| *Metaclisis* Foerster | Cecidomyiidae | Masner (1981) |

**Table 1.** continued

| Genus | Host group | Source |
| --- | --- | --- |
| *Proplatygaster* Kieffer | Cecidomyiidae | Masner and Huggert (1989) |
| *Zelostemma* Masner & Huggert | Cecidomyiidae | Masner and Huggert (1989) |
| ***Isostasius*-cluster** | | |
| *Aceroteta* Kozlov & Masner | unknown | Masner and Huggert (1989) |
| *Annettella* Masner & Huggert | unknown | Masner and Huggert (1989) |
| *Isostasius* Foerster | Cecidomyiidae | Masner and Huggert (1989) |
| *Rao* Masner & Huggert | unknown | Masner and Huggert (1989) |
| ***Inostemma*-cluster** | | |
| *Acerotella* Masner | Cecidomyiidae | Masner (1980*c*) |
| *Inostemma* Haliday | Cecidomyiidae | Muesebeck (1979) |
| *Iphitrachelus* Walker | unknown | Masner and Huggert (1989) |
| *Sacespalus* Kieffer | unknown | Masner and Huggert (1989) |
| ***Allostemma*-cluster** | | |
| *Allostemma* Masner & Huggert | unknown | Masner and Huggert (1989) |
| ***Platygaster*-cluster** | | |
| *Amblyaspis* Foerster | unknown | – |
| *Anopedias* Foerster | unknown | – |
| *Eritrissomerus* Ashmead | Cecidomyiidae | Muesebeck (1979) |
| *Euxestonotus* Fouts | Cecidomyiidae | Muesebeck (1979) |
| *Gastrotrypes* Brues | unknown | – |
| *Isocybus* Foerster | unknown | – |
| *Metanopedias* Brues | Cecidomyiidae | Muesebeck (1979) |
| *Platygaster* Latreille | Cecidomyiidae | Muesebeck (1979) |
| *Trichacis* Foerster | Cecidomyiidae | Masner (1983*c*) |
| *Trichacoides* Dodd | Cecidomyiidae | Austin (1984) |
| ***Synopeas*-cluster** | | |
| *Isorhombus* Foerster | unknown | – |
| *Leptacis* Foerster | Cecidomyiidae | Muesebeck (1979) |
| *Piestopleura* Foerster | unknown | – |
| *Synopeas* Foerster | Cecidomyiidae | Austin (1984) |

## Tribe **Baryconini** Kozlov
## Genus ***Baryconus*** Foerster
(Figs 27–29)

*Diagnosis*

Ovipositor assembly extended and retracted by muscles (i.e. *Ceratobaeus*-type system); ovipositor elongate to extremely elongate, 0·9–1·5× length of metasoma; proximal part of ventral membranous plate not visible above rami of 1st gonapophyses; gonoplacs elongate, about 0·7× length of ovipositor, with concave folds at join with 2nd gonocoxae; 2nd gonapophyses assembly simple, with tear-shaped cavity; proximal arms moderately elongate, broad, 0·15× length of ovipositor; T7+8 fused but narrow cuticular bridge near proximal margin indicating division between plates, parts of T8 sometimes indistinct, curled ventrally; cerci large and leaf-shaped; lateral apodemes elongate, length including T7+8 about 0·9× length of ovipositor; ventral membranous plate large, distinct, longer than lateral apodemes; S6 large, with faint suture near proximal margin indicating division between S6 and S7+8, medial apodeme moderately elongate, robust, length of S6+7 and medial apodeme about 0·6× length of ovipositor.

### Genus *Bracalba* Dodd
(Figs 36, 37)

*Diagnosis*

Ovipositor assembly and telescopic tube extended and retracted by hydrostatic pressure (i.e. *Scelio*-type system); telescopic tube with 2 sections; ovipositor elongate, >0·9× length of metasoma; proximal part of ventral membranous plate present; gonoplacs elongate, 0·75× length of ovipositor, with small spines evenly spaced along margins; 2nd gonapophyses assembly complex; proximal arms slender, very short, <0·1× length of ovipositor; T7+8 fused, no sign of division between tergites, mostly membranous, square, with sclerotised posterior and lateral margins, anterior margin indistinct; cerci present (? shape not clear in dissection); lateral apodemes incorporated into wall of telescopic tube and distinct throughout, elongate, >0·9× length of ovipositor; S6 without medial apodeme.

### Genus *Chromoteleia* Ashmead
(Figs 31, 33)

*Diagnosis*

Ovipositor assembly and telescopic tube extended and retracted by hydrostatic pressure (i.e. *Scelio*-type system); telescopic tube robust, relatively well sclerotised but flexible, with (?) 3 sections; tubular A9 robust, relatively well sclerotised particularly along dorsal ridge, but flexible; ovipositor elongate, about 0·9× length of metasoma; proximal part of ventral membranous plate present; gonoplacs elongate, about 0·7× length of ovipositor; 2nd gonapophyses assembly complex; proximal arms slender, very short, <0·1× length of ovipositor; T7+8 fused, but with division between tergites or clearly separated, moderately well sclerotised, convex, broadly crescent-shaped, with few setae along posterior margin; cerci reduced but leaf-shaped; lateral apodemes incorporated into wall of telescopic tube and distinct throughout, about 0·7× length of ovipositor; S6 without medial apodeme.

### Genus *Oxyscelio* Kieffer
(Figs 38, 39)

*Diagnosis*

Ovipositor assembly and telescopic tube extended and retracted by hydrostatic pressure (i.e. *Scelio*-type system); telescopic tube with (?) 2 sections; ovipositor elongate, 0·95× length of metasoma; proximal part of ventral membranous plate present; gonoplacs moderately elongate, 0·55–0·65× length of ovipositor, with long spines spaced evenly along length; 2nd gonapophyses assembly complex; proximal arms slender, moderately elongate, about 0·15× length of ovipositor; T7+8 fused, no sign of division between tergites, lightly sclerotised, almost semi-circular in shape; cerci small and leaf-shaped; lateral apodemes incorporated into wall of telescopic tube, indistinct or absent for most of length of distal telescopic tube, visible only in proximal one-fifth; S6 without medial apodeme.

---

**Figs 24, 25.** *Scelio fulgidus* Crawford (Scelionini): *24*, ovipositor assembly; *25*, unextended telescopic tube with tergites 7+8. **Fig. 26.** *Heptascelio* sp. (Scelionini): unextended telescopic tube with tergites 7+8. **Figs 27–29.** *Baryconus* sp. (Baryconini): *27*, ovipositor assembly; *28*, sternites 6+7+8 and medial apodeme; *29*, tergites 7+8 and lateral apodemes. Scale lines: *24, 25*, 250 µm; *26*, 125 µm; *27–29*, 250 µm. S6, metasomal sternite 6; S7, metasomal sternite 7+8; vp, ventral membranous plate.

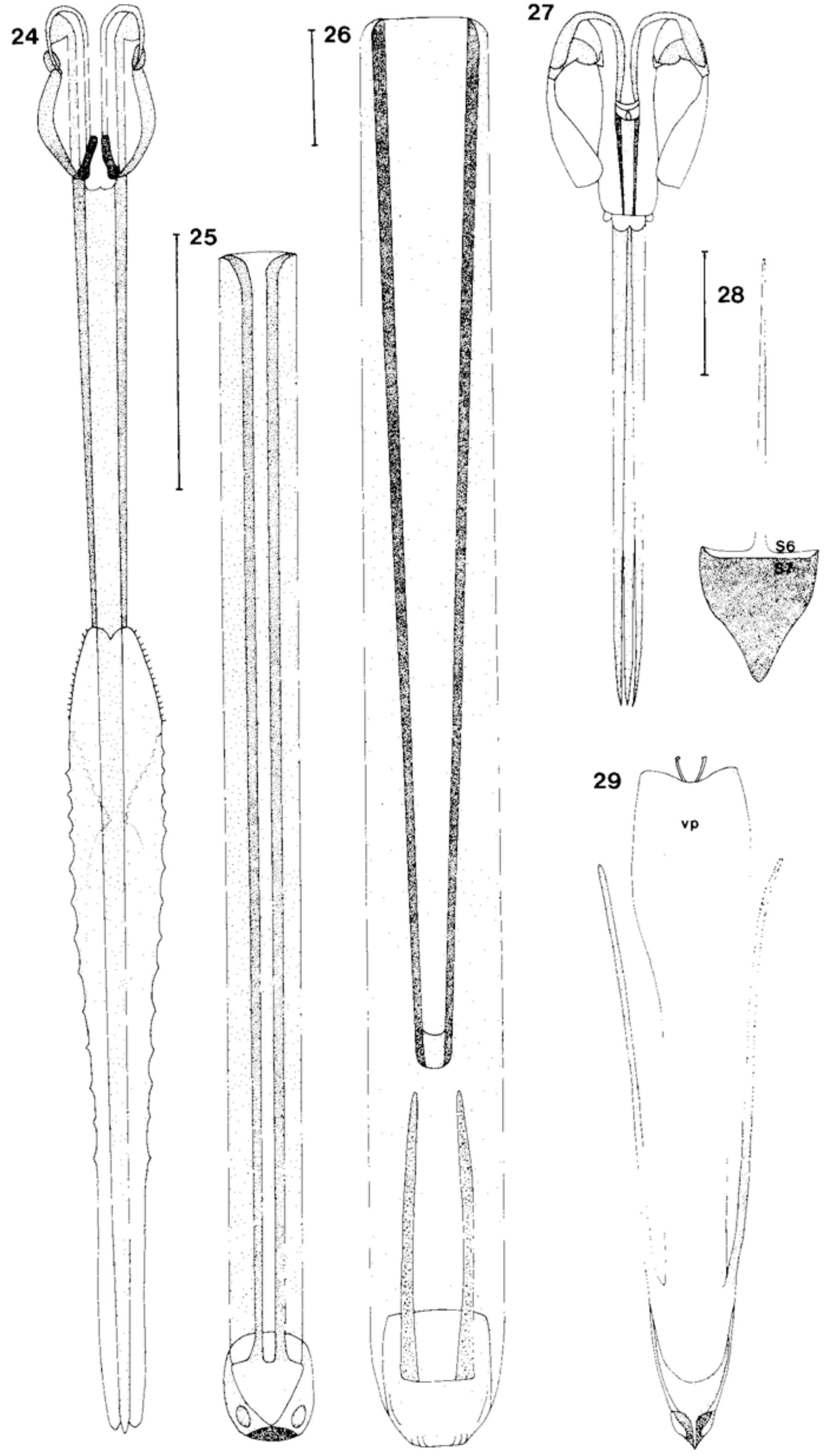
24
25
26
27
28
S6
S7
29
vp

*Comments on Baryconini*

Significantly, the nominal genus *Baryconus* has a *Ceratobaeus*-type ovipositor system while *Bracalba*, *Chromoteleia* and *Oxyscelio* all have a hydrostatically extended telescopic tube (i.e the *Scelio*-type system). On the basis of ovipositor system morphology, the latter three genera are misplaced in the Baryconini and, given that there are no major differences between them in ovipositor characters, they should either be placed in a new tribe or included in an existing one. The most distinguishing feature of the ovipositor system in two of these genera, *Bracalba* and *Oxyscelio*, is the presence of spines along the gonoplacs. Similar structures are known only in species of *Scelio*; however, the latter genus differs in many important characters and is clearly unrelated to *Bracalba* and *Oxyscelio* (Masner 1976; Galloway and Austin 1984). We have not had the opportunity to dissect specimens of *Apegus* Foerster but, given the comments of Masner (1976) that it is very similar to *Baryconus*, this genus is assumed also to have a *Ceratobaeus*-type system.

### Tribe **Calliscelionini** Masner

### Genus ***Calliscelio*** Ashmead

(Figs 1–6; Field and Austin 1994, figs 12, 35, 36, 40–48)

*Diagnosis*

Ovipositor assembly and telescopic tube extended and retracted by hydrostatic pressure (i.e. *Scelio*-type system); telescopic tube finely membranous, greatly elongated, with 3 sections; ovipositor elongate to extremely elongate, usually extending into horn of T1 and thus longer than metasoma, 0·9–1·2× length of metasoma; tubular A9 with minute pointed scale-like sculpture at distal end; proximal part of ventral membranous plate present; gonoplacs moderately elongate to elongate, 0·55–0·75× length of ovipositor; 2nd gonapophyses assembly complex; proximal arms slender, very short, about 0·05× length of ovipositor; T7 separated from and overlying T8, membranous, very slightly more sclerotised than telescopic tube, curled ventrally; cerci present, leaf-like; lateral apodemes incorporated into wall of telescopic tube, distinct or sometimes faint, moderately elongate, about 0·75× length of ovipositor; S6 without medial apodeme although often small medial spine (<0·1× length of S6) and small spine at each lateral corner.

### Genus ***Anteromorpha*** Dodd

(Fig. 32; Field and Austin 1994, figs 11, 34)

*Diagnosis*

Ovipositor assembly and telescopic tube extended and retracted by hydrostatic pressure (i.e. *Scelio*-type system); telescopic tube finely membranous although slightly thickened behind T7+8, elongate, with 4 relatively short sections; ovipositor elongate, >0·9× length of metasoma; proximal part of ventral membranous plate present; gonoplacs elongate, 0·65× length of ovipositor; 2nd gonapophyses assembly complex; proximal arms slender, short, about 0·1× length of ovipositor; T7 separated from and overlying T8, membranous, slightly more sclerotised than telescopic tube, curled ventrally; cerci present, leaf-like; lateral apodemes incorporated into wall of telescopic tube, distinct or sometimes faint, elongate; S6 without medial apodeme.

### Genus ***Palpoteleia*** Kieffer

(Figs 44–46)

*Diagnosis*

Ovipositor assembly extended and retracted by muscles (i.e. *Ceratobaeus*-type system); ovipositor moderately elongate; proximal part of ventral membranous plate present; gonoplacs moderately elongate, about 0·6× length of ovipositor; 2nd gonapophyses assembly simple;

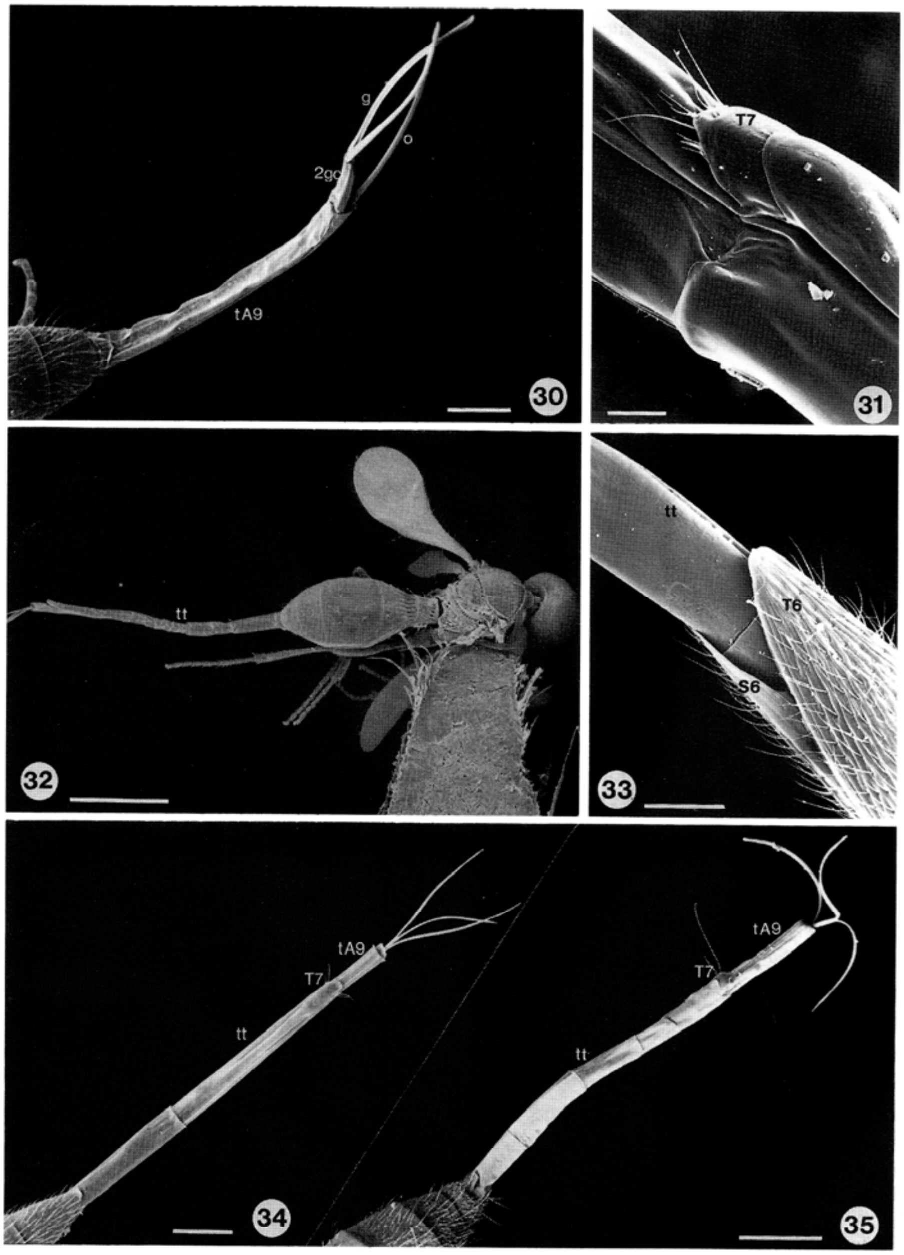


**Fig. 30.** *Sceliomorpha* sp. (Sparasionini), extended ovipositor system. **Figs 31, 33.** *Chromoteleia* sp. (Baryconini): *31*, tergites 7+8 on extended telescopic tube; *33*, distal metasoma and proximal end of extended telescopic tube. **Fig. 32.** *Anteromorpha* sp. (Calliscelionini), whole insect showing partly extended telescopic tube and ovipositor system. **Fig. 34.** Distal mesosoma of *Calotelea* sp. (Psilanteridini) showing partly extended telescopic tube and ovipositor system. **Fig. 35.** Distal mesosoma of *Paridris* sp. (Calliscelionini) showing partly extended telescopic tube and ovipositor system. Scale lines: *30*, *33*, *35*, 200 µm; 31, 50 µm; *32*, 0·5 mm; *34*, 100 µm. g, gonoplac; o, ovipositor; tA9, tubular part of abdominal segment 9; S6, metasomal sternites 6+7+8; T6, metasomal tergite 6; T7, metasomal tergites 7+8; tt, telescopic tube; 2gc, 2nd gonocoxae.

proximal arms slender, moderately elongate, about 0·15× length of ovipositor; T7+8 fused but T7 indicated by narrow cuticular bridge near proximal margin of T8; cerci present and leaf-shaped; lateral apodemes elongate, length including T7+8 about 0·9× length of ovipositor, with two distinct sections curving in opposite directions; distal part of ventral membranous plate indistinct; S6 with elongate medial apodeme, length of S6 and medial apodeme about 0·75× length of ovipositor.

Genus ***Paridris*** Ashmead

(Figs 35, 40, 41)

*Diagnosis*

Ovipositor assembly and telescopic tube extended and retracted by hydrostatic pressure (i.e. *Scelio*-type system); telescopic tube finely membranous, elongate, with 4 relatively short sections; ovipositor elongate, 0·95× length of metasoma; proximal part of ventral membranous plate present; gonoplacs extremely elongate, 0·80–0·85× length of ovipositor; 2nd gonapophyses assembly complex; proximal arms slender, very short, 0·05× length of ovipositor; T7 separated from and overlying T8, slightly more sclerotised than telescopic tube; cerci present, leaf-like; lateral apodemes incorporated into wall of telescopic tube, narrow but distinct, elongate, about 0·8× length of ovipositor; S6 without medial apodeme.

Genus ***Triteleia*** Kieffer

(Figs 42, 43)

*Diagnosis*

Ovipositor assembly and telescopic tube extended and retracted by hydrostatic pressure (i.e. *Scelio*-type system); telescopic tube membranous but robust, elongate, with 2–3 sections; ovipositor elongate, 0·9× length of metasoma; proximal part of ventral membranous plate present; gonoplacs moderately elongate, 0·55× length of ovipositor; 2nd gonapophyses assembly complex; proximal arms slender, moderately elongate, 0·19× length of ovipositor; T7 separated from and overlying T8, distinct, more sclerotised than telescopic tube; cerci present, leaf-like; lateral apodemes incorporated into wall of telescopic tube, indistinct in proximal half or virtually absent; S6 without medial apodeme.

*Comments on Calliscelionini*

Other genera dissected but not figured here are *Holoteleia* Kieffer, *Lispoteleia* Galloway, *Macroteleia* Westwood, *Oethecoctonus* Ashmead, *Phoenoteleia* Kieffer and *Probaryconus*. All have a *Scelio*-type ovipositor system, but some differ in detail compared with *Calliscelio*. *Holoteleia*, *Lispoteleia*, *Oethecoctonus* and *Phoenoteleia* all have T7 slightly more sclerotised, while *Macroteleia* has a much more robust telescopic tube that is flexible but heavily sclerotised compared with that of the other genera in the tribe. All of the above genera have an elongate ovipositor (about 0·9× length of metasoma or longer if T1 has a horn), a telescopic tube with either 2 or 3 sections, proximal arms that are slender and relatively short, T7 separated from and overlying T8, cerci that are leaf-like in shape; lateral apodemes incorporated into the wall of the telescopic tube that are elongate and distinct to faint, and S6 without medial apodeme.

**Figs 36, 37.** *Bracalba* sp. (Baryconini): *36*, ovipositor assembly; *37*, unextended telescopic tube with tergites 7+8. **Figs 38, 39.** *Oxyscelio* sp. (Baryconini): *38*, ovipositor assembly; *39*, unextended telescopic tube with tergites 7+8. **Figs 40, 41.** *Paridris* sp. (Calliscelionini): *40*, ovipositor assembly; *41*, unextended telescopic tube with tergites 7+8. Scale lines: *36*, *37*, 250 µm; *38*, *39*, 250 µm; *40*, *41*, 250 µm.

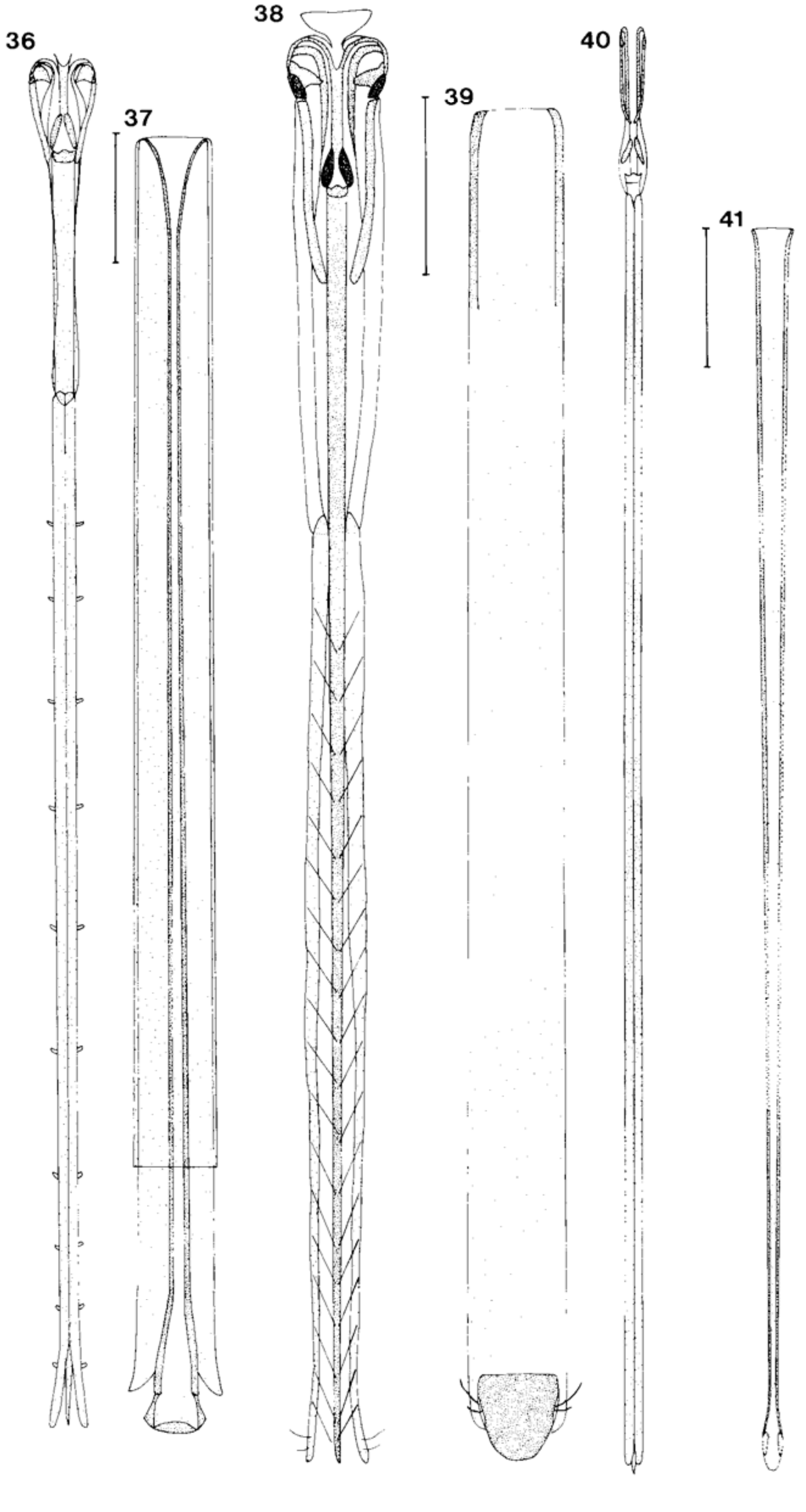
36
37
38
39
40
41

Apart from the taxa discussed above, external examination of two other genera in this tribe, *Crama* Galloway and *Dichoteleas* Kieffer, show them to have a *Scelio*-type ovipositor system, (i.e. dried specimens have the telescopic tube extruded from the metasoma).

Significantly, *Habroteleia* Kieffer and *Palpoteleia* do not fit the above pattern in that they have a *Ceratobaeus*-type ovipositor system and so, based on these characters, they are clearly misplaced in the tribe Calliscelionini. *Habroteleia* is further distinguished by lacking a medial apodeme on S6 (see Comments under *Mantibaria* Kirby), and *Palpoteleia* by having a 2nd gonapophyses assembly that is simple and lateral apodemes that have two distinct sections curving in opposite directions (Figs 44–46). The latter character is apparently unique within the Platygastroidea. These two genera are not closely related based on external characters (Masner 1976), nor on the details of the ovipositor system, which for *Habroteleia* (not figured here) are more similar to *Fusicornia* Risbec which also lacks a medial apodeme (Figs 52, 53).

Tribe **Psilanteridini** Kozlov

Genus ***Psilanteris*** Kieffer

(Figs 47, 48; Field and Austin 1994, figs 37–39)

*Diagnosis*

Ovipositor assembly and telescopic tube extended and retracted by hydrostatic pressure (i.e. *Scelio*-type system); ovipositor assembly somewhat delicate in form; telescopic tube membranous and elongate, with 3 sections; ovipositor elongate, about 0·8× length of metasoma; proximal part of ventral membranous plate absent; gonoplacs short, 0·45× length of ovipositor; 2nd gonapophyses assembly complex; proximal arms slender, short, 0·11× length of ovipositor; T7 separated from and overlying T8, moderately well sclerotised, curled ventrally; cerci present, leaf-like; lateral apodemes incorporated into wall of telescopic tube, distinct, moderately elongate, about 0·7× length of ovipositor; S6 without medial apodeme.

*Genera Similar to Psilanteris*

Genus ***Duta*** Nixon

(Field and Austin 1994, fig. 33)

*Diagnosis*

Ovipositor assembly and telescopic tube extended and retracted by hydrostatic pressure (i.e. *Scelio*-type system); telescopic tube membranous, elongate, with 4 relatively short sections; tubular A9 with 2 narrow sclerotised dorsal ridges; ovipositor elongate, about 0·9× length of metasoma; (?) proximal part of ventral membranous plate present; gonoplacs elongate; 2nd gonapophyses assembly complex; proximal arms slender, (?) moderately elongate; T7 separated from and overlying T8, slightly more sclerotised than telescopic tube; cerci present, leaf-like; lateral apodemes incorporated into wall of telescopic tube, distinct, elongate, about 0·8× length of ovipositor; S6 without medial apodeme.

**Figs 42, 43.** *Triteleia* sp. (Calliscelionini): *42*, ovipositor assembly; *43*, unextended telescopic tube with tergites 7+8. **Figs 44–46.** *Palpoteleia* sp. (Calliscelionini): *44*, ovipositor assembly; *45*, sternites 6+7+8 and medial apodeme; *46*, tergites 7+8 and lateral apodemes. **Figs 47, 48.** *Psilanteris* sp. (Psilanteridini): *47*, ovipositor assembly; *48*, unextended telescopic tube with tergites 7+8. **Figs 49–51.** *Anteris bilineatus* Thomson (Psilanteridini): *49*, tergites 7+8 and lateral apodemes; *50*, ovipositor assembly; *51*, sternites 6+7+8 and medial apodeme. Scale lines: *42*, *43*, 250 µm; *44–46*, 125 µm; *47–49*, 250 µm; *50*, *51*, 125 µm.

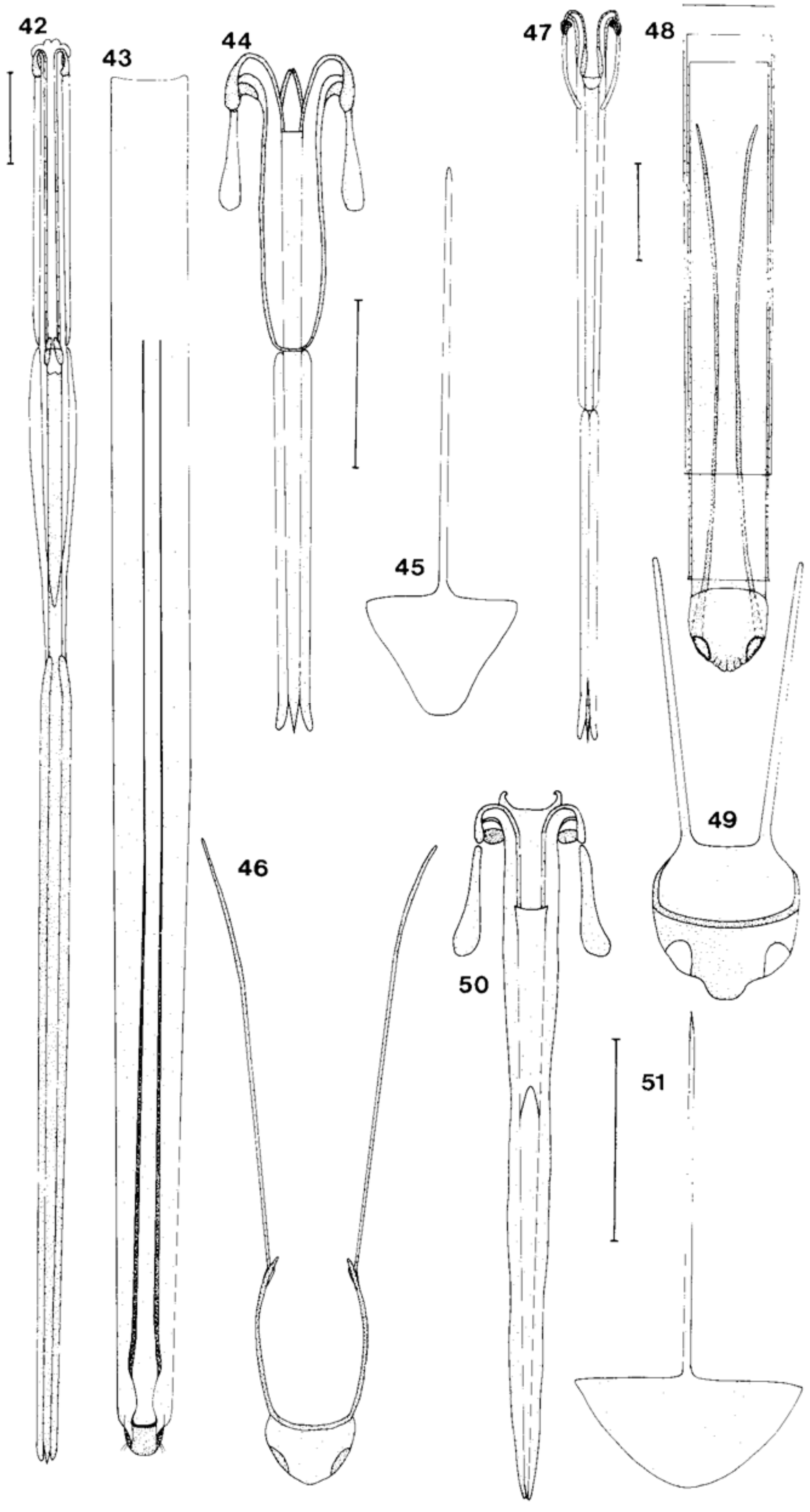
42
43
44
45
46
47
48
49
50
51

Genus ***Harringtonia*** Masner

(Field and Austin 1994, figs 29–32)

*Diagnosis*

Ovipositor assembly and telescopic tube extended and retracted by hydrostatic pressure (i.e. *Scelio*-type system); telescopic tube membranous, elongate, with 4 relatively short sections; ovipositor elongate, about 0·9× length of metasoma; (?) proximal part of ventral membranous plate present; gonoplacs elongate; 2nd gonapophyses assembly complex; proximal arms slender, (?) moderately elongate; T7 separated from and overlying T8, slightly more sclerotised than telescopic tube; cerci present, leaf-like; lateral apodemes incorporated into wall of telescopic tube, distinct, elongate, about 0·8× length of ovipositor; S6 without medial apodeme.

Genus ***Merriwa*** Dodd

(Figs 54, 55)

*Diagnosis*

Ovipositor assembly and telescopic tube extended and retracted by hydrostatic pressure (i.e. *Scelio*-type system); ovipositor assembly rather delicate in form; telescopic tube finely membranous, elongate, (?) with 2 sections; ovipositor extremely elongate, very slender, same length of metasoma; proximal part of ventral membranous plate present; gonoplacs elongate, 0·7× length of ovipositor; 2nd gonapophyses assembly complex; proximal arms slender, very short, 0·05× length of ovipositor; T7 separated from and overlying T8, slightly more sclerotised than telescopic tube; T8 curled ventrally, anterior margin indistinct; cerci present, leaf-like; lateral apodemes incorporated into wall of telescopic tube, distinct, elongate, about 0·8× length of ovipositor; S6 without medial apodeme.

Genus ***Spiniteleia*** Masner

(Fig. 82)

*Diagnosis*

Ovipositor assembly and telescopic tube extended and retracted by hydrostatic pressure (i.e. *Scelio*-type system); telescopic tube membranous and elongate, with 3 sections; ovipositor and gonoplacs elongate; (?) proximal part of ventral membranous plate present; 2nd gonapophyses assembly complex; proximal arms slender, very short, about 0·05× length of ovipositor; T7 separated from and overlying T8, moderately well sclerotised, curled ventrally; cerci present, leaf-like; lateral apodemes incorporated into wall of telescopic tube, distinct, moderately elongate, about 0·7× length of ovipositor, bowed in distal one-third; S6 without medial apodeme.

*Genera Differing from Psilanteris*

Genus ***Anteris*** Foerster

(Figs 49–51)

*Diagnosis (based on the type species,* A. bilineatus *Thomson)*

Ovipositor assembly extended and retracted by muscles (i.e. *Ceratobaeus*-type system); ovipositor moderately elongate; proximal part of ventral membranous plate present; gonoplacs elongate, about 0·65× length of ovipositor; 2nd gonapophyses assembly complex; proximal arms moderately broad, moderately elongate, 0·18× length of ovipositor; T7 fused to T8 but distinct, seen as a sclerotised ring on proximal margin of T8; cerci present, leaf-like; lateral apodemes moderately elongate, length including T7+8 about 0·7× length of ovipositor; distal

part of ventral membranous plate attached to lateral apodemes; S6 with moderately elongate medial apodeme, length of S6 and medial apodeme about 0·7× length of ovipositor.

## Genus *Fusicornia* Risbec
(Figs 52, 53)

*Diagnosis*

Ovipositor assembly extended and retracted by muscles (i.e. *Ceratobaeus*-type system); ovipositor elongate, about 0·9× length of metasoma; proximal part of ventral membranous plate present; gonoplacs slender, moderately elongate, about 0·6× length of ovipositor; 2nd gonapophyses assembly complex, located at proximal extremity of ovipositor assembly; proximal arms moderately broad, moderately elongate, about 0·15× length of ovipositor; T7+8 fused, no sign of division between tergites, almost membranous distally, heavily sclerotised proximally and strongly curved so that leaf-like cerci face laterally and are hidden in dorsal view; lateral apodemes moderately elongate, length including T7+8 0·65× length of ovipositor, ventral membranous plate extending half way along apodemes; S6 with medial apodeme absent.

## Genus *Leptoteleia* Kieffer
(Figs 56–58)

*Diagnosis*

Ovipositor assembly extended and retracted by muscles (i.e. *Ceratobaeus*-type system); ovipositor elongate, about 0·95× length of metasoma; proximal part of ventral membranous above rami of 1st gonapophyses apparently absent; gonoplacs short, about half length of ovipositor; 2nd gonapophyses assembly simple, with tear-shaped attachment; proximal arms slender, elongate, curled, sclerotised medially, 0·26× length of ovipositor; T7 fused to T8 but distinct, seen as a sclerotised ring on proximal margin of T8, T8 curled ventrally, cerci small, indistinct; lateral and latero-ventral apodemes attached, latero-ventral apodemes shorter and attached to large ventral membranous plate, lateral apodemes including T7+8 about 0·8× length of ovipositor, ventral membranous plate longer than lateral apodemes; S6 strongly curved dorsally, with suture near proximal margin indicating division between S6 and S7, medial apodeme moderately elongate, length of S6 and medial apodeme about 0·65× length of ovipositor.

## Genus *Opisthacantha* Ashmead
(Figs 59–61, 68, 70)

*Diagnosis (based on the type species,* O. mellipes *Ashmead)*

Ovipositor assembly extended and retracted by muscles (i.e. *Ceratobaeus*-type system); ovipositor elongate, 0·9–1·0× length of metasoma; proximal part of ventral membranous plate present; gonoplacs moderately elongate, 0·65× length of metasoma; 2nd gonapophyses assembly complex; proximal arms slender, short, about 0·1× length of ovipositor; T7 and T8 fused but T7 often indicated by less sclerotised anterior margin, T7+8 loosely attached to T6 and often protruding well away from it at the end of a short membranous flap; cerci leaf-like; lateral and latero-ventral apodemes fused, very elongate, distal parts with ventral membranous plate attached, proximal end of latero-ventral apodemes connected to lateral pair by hooks; lateral apodemes with T7+8 0·8× length of ovipositor; S6 with elongate medial apodeme, 0·7× length of ovipositor; in dead specimens tubular A9 often protruding from metasoma with ovipositor and gonoplacs, sometimes complete ovipositor assembly outside of metasoma but all contained within tubular A9 except for ovipositor shaft.

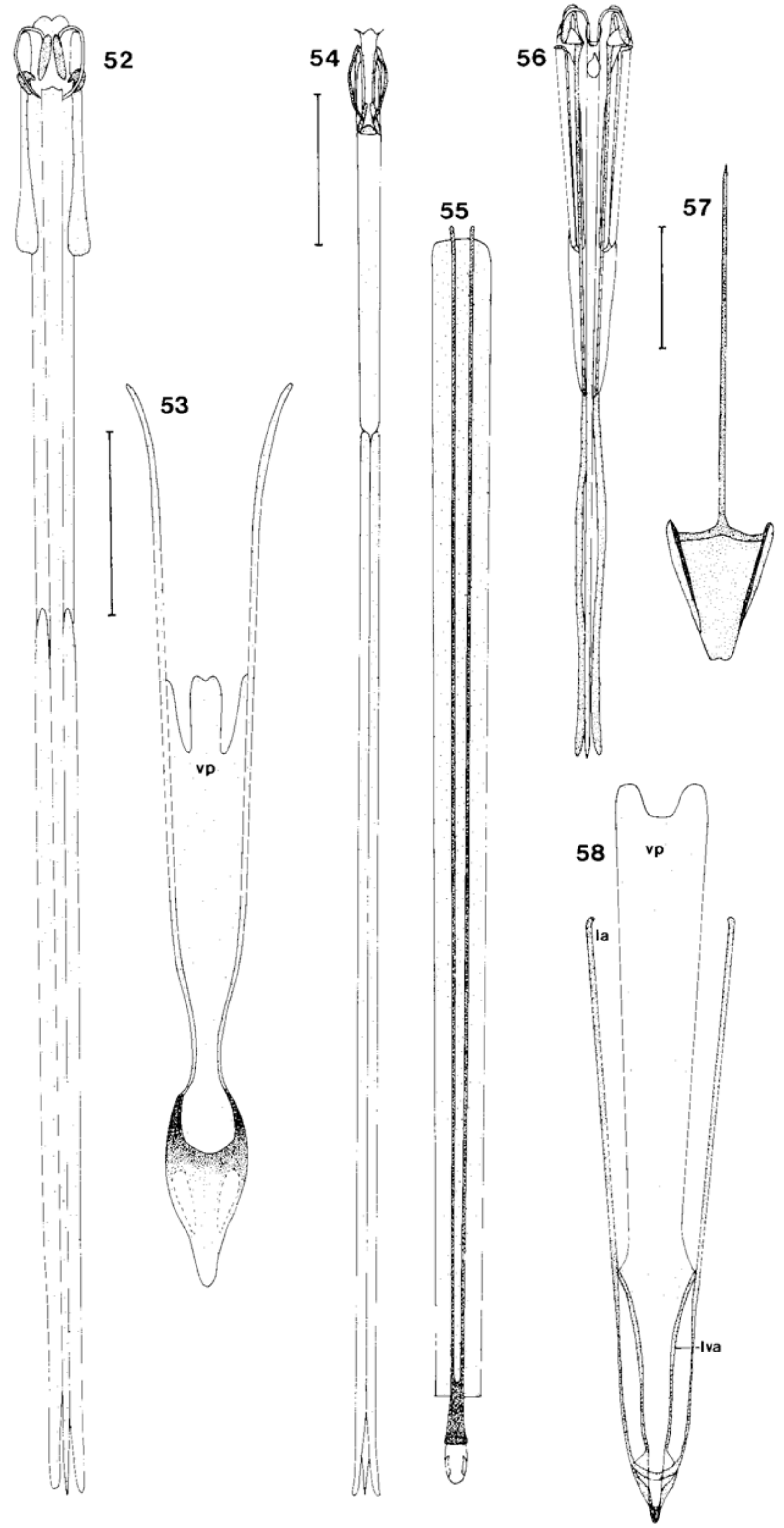
52
53
vp
54
55
56
57
58
vp
la
lva

## Genus ***Styloteleia*** Kieffer
(Figs 64–67)

*Diagnosis*

Ovipositor assembly extended and retracted by muscles (i.e. *Ceratobaeus*-type system); ovipositor extremely elongate, longer than metasoma because of horn on T1, about 1·1× length of metasoma; proximal part of ventral membranous plate absent; gonoplacs elongate, 0·7× length of ovipositor; 2nd gonapophyses assembly absent; proximal arms slender, moderately elongate, 0·15× length of ovipositor, with membranous extensions reaching about half along length of ovipositor; T7+8 fused but distinct from each other, extremely elongate; cerci leaf-like; elongate lateral and latero-ventral apodemes separated in proximal half, joining near apex, latero-ventral pair becoming indistinct where they join distal part of ventral membranous plate, membranous plate divided medially and attached to apodemes by bridge approximately two-thirds along apodemes, apodemes plus T7+8 about 0·7× length of ovipositor; S6 elongate, with medial apodeme 0·6× length of ovipositor.

*Comments on Psilanteridini*

The Calliscelionini and Psilanteridini are the largest tribes within the Platygastroidea, containing about 25 genera each. However, while the former tribe uniformly possesses a *Scelio*-type ovipositor system (with the exception of two genera, *Habroteleia* and *Palpoteleia*), the Psilanteridini is a mixed assemblage of genera containing both types of ovipositor systems. Apart from the genera described above in detail, external examination of 10 other genera, *Calotelea* Westwood, *Genatropis* Galloway, *Lidgbirdius* Galloway, *Mallateleia* Dodd, *Mecix* Masner, *Nyleta* Dodd, *Oxyteleia* Kieffer, *Paraduta* Szabó, *Shreemana* Nixon and *Thoronella* Masner (Figs 84, 85), show them to have a *Scelio*-type system, (i.e. dried specimens have the telescopic tube extruded from the metasoma). Examination of the ovipositor system for all genera in the Calliscelionini and Psilanteridini that possess a *Scelio*-type system show that there is no reason, based on this suite of characters, why they should be accommodated in separate tribes.

At least five genera currently placed in the Psilanteridini (Masner 1976; Galloway and Austin 1984), *Anteris s. str., Fusicornia, Leptoteleia, Opisthacantha s. str.* (Masner, in press) and *Styloteleia,* possess a *Ceratobaeus*-type ovipositor system. It therefore seems highly likely that this tribe is not monophyletic and these genera are misplaced. Further, it is unclear where these genera belong (Fig. 189). They cannot be accommodated easily into any existing tribes and so eventually, when the relationships among these genera are resolved, they may need to be placed into new higher-level categories. Of these genera, several show a number of interesting or unique characteristics. *Fusicornia* and *Leptoteleia* have the ventral membranous plate greatly elongated (Figs 53, 58), a character found in only one other genus examined (*Baryconus*). *Leptoteleia* also has an unusual 2nd gonapophyses assembly, similar to the simple form, but also with a tear-shaped structure of unknown homology, which appears to be loosely attached by a membrane near the 2nd gonapophyses assembly (Fig. 58). *Fusicornia* is one of only seven genera (along with *Echthrodesis* Masner, *Habroteleia, Mantibaria, Neuroscelio, Nixonia* and *Sparasion*) that has a *Ceratobaeus*-type system but lacks a medial apodeme on S6. *Styloteleia* is distinguished by several unusual characters: the 2nd gonapophyses assembly is absent, membranous extensions from the proximal arms reach about half way along the length of the ovipositor, and a ventral membranous plate is divided medially and spanned by a cross-bridge (Figs 64, 65), a character paralleled only in *Opisthacantha s. str.* (Fig. 60).

---

**Figs 52, 53.** *Fusicornia* sp. (Psilanteridini): *52*, ovipositor assembly; *53*, tergites 7+8 and lateral apodemes. **Figs 54, 55.** *Merriwa* sp. (Psilanteridini): *54*, ovipositor assembly; *55*, unextended telescopic tube with tergites 7+8. **Figs 56–58.** *Leptoteleia* sp. (Psilanteridini): *56*, ovipositor assembly; *57*, sternites 6+7+8 and medial apodeme; *58*, tergites 7+8 and lateral apodemes. Scale lines: *52, 53*, 250 µm; *54, 55*, 250 µm; *56–58*, 250 µm. la, lateral apodeme; lva, latero-ventral apodeme; vp, ventral membranous plate.

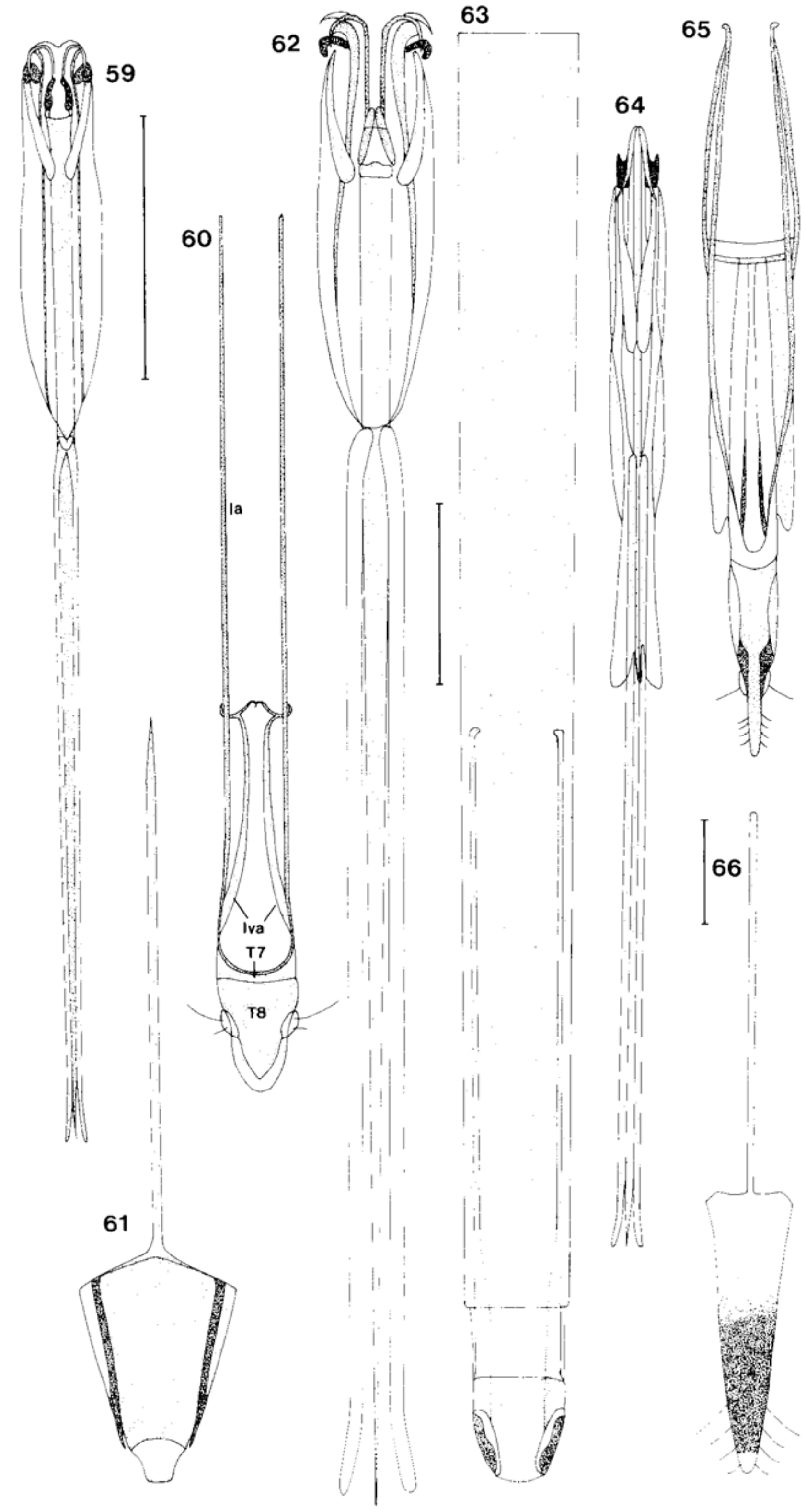
59
60
Ia
Iva
T7
T8
61
62
63
64
65
66

Interestingly, *Anteris* poses a number of difficulties. On the basis of the type species, *Anteris bilineatus*, this genus (*s. str.*) has a *Ceratobaeus*-type ovipositor system and therefore is misplaced within the Psilanteridini. However, several undescribed Neotropical species (in CNCI), which externally appear to be congeneric with *A. bilineatus*, have a *Scelio*-type ovipositor, and undoubtedly belong to a new, as yet undescribed, genus. This example highlights the need for the ovipositor assembly to be critically examined (i.e. dissected), when describing or reassessing higher-level taxa of scelionids.

Of all genera examined from this tribe, *Opisthacantha s. str.* presents the most unique situation. The type species, *Opisthacantha mellipes* (Figs 68, 70), and two undescribed species, *Opisthacantha* sp. 1 and 2 (Figs 59–61), have a *Ceratobaeus*-type system but with a number of significant modifications. In particular, tubular A9 often protrudes from the metasoma with the ovipositor and gonoplacs, and T7+8 is loosely attached to T6 and extends away from it at the end of a short membranous flap (Fig. 70). Sometimes the complete ovipositor assembly is extended from the metasoma (Fig. 68) and all components, except for ovipositor shaft, are contained within tubular A9. Also, in these species the lateral and latero-ventral apodemes are connected (apparently differently to that in *Styloteleia*) by hooks and the latero-ventral pair are joined by part of the ventral membranous plate. The phylogenetic position of *Opisthacantha* is problematic. Clearly, on the basis of the ovipositor system, it is not related to *Psilanteris* or other genera in this tribe that have a *Scelio*-type ovipositor system. The structure and connection between the apodemes of *Opisthacantha* may align it with *Leptoteleia* or *Styloteleia*, but this cannot be confirmed until the polarity of this character system is determined and until a more complete phylogenetic analysis has been undertaken.

## Tribe **Aradophagini** Kozlov

### Genus ***Aradophagus*** Ashmead

(Figs 71–73, 83)

*Diagnosis*

Ovipositor assembly and telescopic tube extended and retracted by hydrostatic pressure (i.e. *Scelio*-type system); telescopic tube finely membranous although slightly thickened dorsally, with at least 2 relatively short sections; ovipositor elongate, about 0·8× length of metasoma; proximal part of ventral membranous plate absent; gonoplacs elongate, 0·67× length of ovipositor; 2nd gonapophyses assembly complex; proximal arms short and broad, 0·13× length of ovipositor; T7+8 fused, no sign of division between tergites, moderately well sclerotised, sometimes with small medial plate on posterior margin; cerci present, broadly leaf-like; lateral apodemes incorporated into wall of telescopic tube, protruding from proximal end of tube, distinct, short, 0·55× length of ovipositor; S6 broad, with elongate medial apodeme.

*Comments on Aradophagini*

The position of this tribe has been problematic. Kieffer (1926) placed *Aradophagus* in the Telenominae, while Kozlov (1970) raised it to monotypic tribal status within this subfamily

---

**Figs 59–61.** *Opisthacantha* sp. (Psilanteridini): *59*, ovipositor assembly; *60*, tergites 7+8 and lateral apodemes; *61*, sternites 6+7+8 and medial apodeme. **Figs 62, 63.** *Sceliacanthella* sp. (unassigned): *62*, ovipositor assembly; *63*, unextended telescopic tube with tergites 7+8. **Figs 64–66.** *Styloteleia* sp. (Psilanteridini): *64*, ovipositor assembly; *65*, tergites 7+8 and lateral apodemes; *66*, sternites 6+7+8 and medial apodeme. Scale lines: *59–61*, 250 µm; *62*, *63*, 125 µm; *64–66*, 250 µm. la, lateral apodeme; lva, latero-ventral apodemes; T7, metasomal tergite 7; T8, metasomal tergite 8.

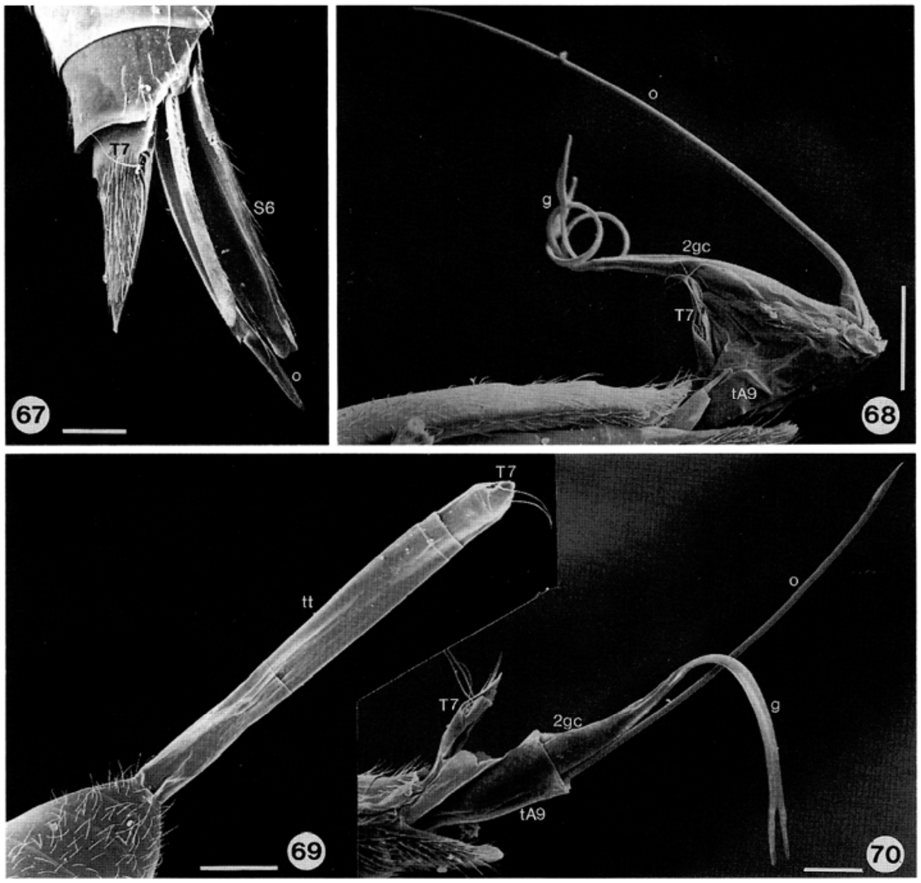


**Fig. 67.** *Styloteleia* sp. (Psilanteridini), sternites 6+7+8 deflected from tergites 7+8 to show fully retracted ovipositor. **Figs 68, 70.** *Opisthacantha mellipes* Ashmead (Psilanteridini): *68*, ovipositor system fully extended from body (under artificial conditions); *70*, ovipositor system extended from body (normal condition). **Fig. 69.** *Sceliacanthella* sp. (unassigned), partly extended telescopic tube. Scale lines: 100 µm. g, gonoplac; o, ovipositor; S6, metasomal sternites 6+7+8; tA9, tubular part of abdominal segment 9; T6, metasomal tergite 6; T7, metasomal tergites 7+8; tt, telescopic tube; 2gc, 2nd gonocoxae.

along with *Tiphodytes* Bradley (see under Thoronini). On the basis of characters associated with the antennae, ocelli and metasomal segments, Masner (1976) transferred the tribe to the Scelioninae, believing it to have relationships with the Psilanteridini. Two new genera were later added, *Abuko* Masner & Huggert and *Ladora* Masner & Huggert (1979*b*), but these recently have been synonymised with *Aradophagus* (García and Masner 1994). Clearly from the morphology of the ovipositor system, the Aradophagini does not belong to the Telenominae, but to the group of tribes including the Scelionini, Calliscelionini and Psilanteridini that possess a *Scelio*-type ovipositor (Fig. 189).

*Aradophagus* is the only genus with a *Scelio*-type ovipositor system that has S6 with a medial apodeme, a structure normally associated with the *Ceratobaeus*-type system. Also unusual are the lateral apodemes which protrude proximally past the telescopic tube when in the folded state (i.e. at rest), a character known only in two other genera, *Platyscelio* Kieffer (Platyscelionini) and *Neoscelio* Dodd (Neoscelionini).

Tribe **Neoscelionini** Kozlov

Genus ***Neoscelio*** Dodd

(Figs 109, 110)

*Diagnosis*

Ovipositor assembly and telescopic tube extended and retracted by hydrostatic pressure (i.e. *Scelio*-type system); telescopic tube moderately robust, with 2 or 3 sections; ovipositor moderately elongate, about 0·75× length of metasoma or longer; proximal part of ventral membranous plate present; gonoplacs elongate, 0·7× length of ovipositor; 2nd gonapophyses assembly complex; proximal arms slender, very short, 0·075× length of ovipositor; T7+8 fused, no sign of division between tergites, moderately well sclerotised, hexagonal in shape, cerci elongate, set in lateral recesses; lateral apodemes sinuate, incorporated into wall of telescopic tube, moderately elongate, 0·65× length of ovipositor, protruding proximally from telescopic tube, connection to T7+8 membranous; S6 without medial apodeme.

*Comments on Neoscelionini*

This monotypic tribe was recently proposed for *Neoscelio*, an endemic Australian genus of very large scelionid wasps (up to 5 mm) of unknown host relationships (Table 1). Masner (1976) could not place the genus, but Galloway and Austin (1984) proposed that it is closely allied to the Calliscelionini. The structure of the ovipositor system of *Neoscelio* supports such a relationship, in that both groups have the derived *Scelio*-type system, but whether the genus is more closely related to the Calliscelionini than any other tribe that possesses this type of ovipositor is yet to be determined by a more complete cladistic analysis. The most unusual features of the *Neoscelio* system are the sinuate lateral apodemes and the fact that they protrude proximally past the telescopic tube when at rest, the latter character being known only in two other genera, *Platyscelio* and *Aradophagus*.

Tribe **Cremastobaeini** Masner

Genus ***Cremastobaeus*** Ashmead

(Figs 74–76)

*Diagnosis*

Ovipositor assembly extended and retracted by muscles (i.e. *Ceratobaeus*-type system); ovipositor elongate, >0·9× length of metasoma; proximal part of ventral membranous plate present; gonoplacs short, 0·45× length of ovipositor; 2nd gonapophyses assembly simple; proximal arms moderately elongate and broad, 0·2× length of ovipositor; T7+8 fused, no sign of division between tergites, well sclerotised; cerci present, leaf-shaped; lateral apodemes short, length including T7+8 about half length of ovipositor, sometimes with small distal part of ventral membranous plate present; S6 with anterior suture indicating division between S6 and S7+8, medial apodeme elongate, as long as lateral apodeme, length including S6 about 0·65× length of ovipositor.

*Comments on Cremastobaeini*

This monotypic tribe is highly distinctive because of the structure of the antennae and metasoma (Masner 1976: Galloway and Austin 1984). Its ovipositor system is the typical *Ceratobaeus*-type and, apart from the gonoplacs being short, it has no distinctive features. The relationships of *Cremastobaeus* are not known, a fact that is explicit in the genus having been given separate tribal status.

### Tribe **Mantibariini** Kozlov

### Genus ***Mantibaria*** Kirby

(Figs 77–79)

*Diagnosis*

Ovipositor assembly extended and retracted by muscles (i.e. *Ceratobaeus*-type system); ovipositor moderately elongate, about 0·7× length of metasoma; proximal part of ventral membranous plate present; details of gonoplacs unknown; 2nd gonapophyses assembly simple; proximal arms very elongate and broad, 0·3× length of ovipositor; T7+8 fused, no sign of division between tergites, partly membranous, distal margin and sometimes medial region sclerotised; cerci replaced by circular sensory plates; lateral and latero-ventral apodemes present, moderately robust, with T7+8 about 0·7× length of ovipositor, sometimes apparently connected basally but not connected apically; distal part of ventral membranous plate apparently absent; latero-ventral apodemes apparently only loosely attached to S6; S6 without medial apodeme.

*Comments on Mantibariini*

*Mantibaria* is an unusual genus reflected in its monotypic tribal placement. It has several special morphological features, particularly short tarsal segments and blade-like femora, that are proposed to be adaptations for phoresy on its mantid hosts (Masner 1976; Galloway and Austin 1984; Table 1). The ovipositor system, although clearly being of the *Ceratobaeus*-type, also possesses a number of peculiar features: the presence of elongate lateral and latero-ventral apodemes, cerci that are apparently replaced by circular sensory plates, and the absence of a medial apodeme on S6. Only six other genera of scelionids lack a medial apodeme (*Echthrodesis*, *Fusicornia*, *Habroteleia*, *Nixonia*, *Neuroscelio* and *Sparasion*). The sensory plates on T7+8 are unique to this genus.

### Tribe **Platyscelionini** Kozlov

### Genus ***Platyscelio*** Kieffer

(Figs 80, 81)

*Diagnosis*

Ovipositor assembly and telescopic tube extended and retracted by hydrostatic pressure (i.e. *Scelio*-type system); telescopic tube with 2 sections; ovipositor moderately elongate; proximal part of ventral membranous plate present; gonoplacs elongate, about 0·7× length of ovipositor; 2nd gonapophyses assembly complex; 2nd gonocoxae developed as broad membranous plates; proximal arms slender, moderately elongate, 0·17× length of ovipositor; T7+8 membranous, no sign of division between tergites; cerci large and prominent, approximately leaf-shaped; lateral apodemes incorporated into wall of telescopic tube, protruding from proximal end of tube, indistinct proximally (i.e. not obviously reaching to T7+8), length including T7+8 0·63× length of ovipositor; S6 without medial apodeme.

---

**Figs 71–73.** *Aradophagus fasciatus* Ashmead (Aradophagini): *71*, ovipositor assembly; *72*, tergites 7+8 and lateral apodemes; *73*, sternites 6+7+8 and medial apodeme. **Figs 74–76.** *Cremastobaeus* sp. (Cremastobaeini): *74*, ovipositor assembly; *75*, tergites 7+8 and lateral apodemes; *76*, sternites 6+7+8 and medial apodeme. **Figs 77–79.** *Mantibaria* sp. (Mantibariini): *77*, ovipositor assembly (detail of gonoplacs unknown); *78*, tergites 7+8 and lateral apodemes; *79*, sternites 6+7+8. **Figs 80, 81.** *Platyscelio* sp. (Platyscelionini): *80*, ovipositor assembly; *81*, unextended telescopic tube with tergites 7+8. Scale lines: *71–73*, 125 μm; *74–76*, 250 μm; *77–79*, 250 μm; *80, 81*, 250 μm. la, lateral apodeme; lva, latero-ventral apodeme.

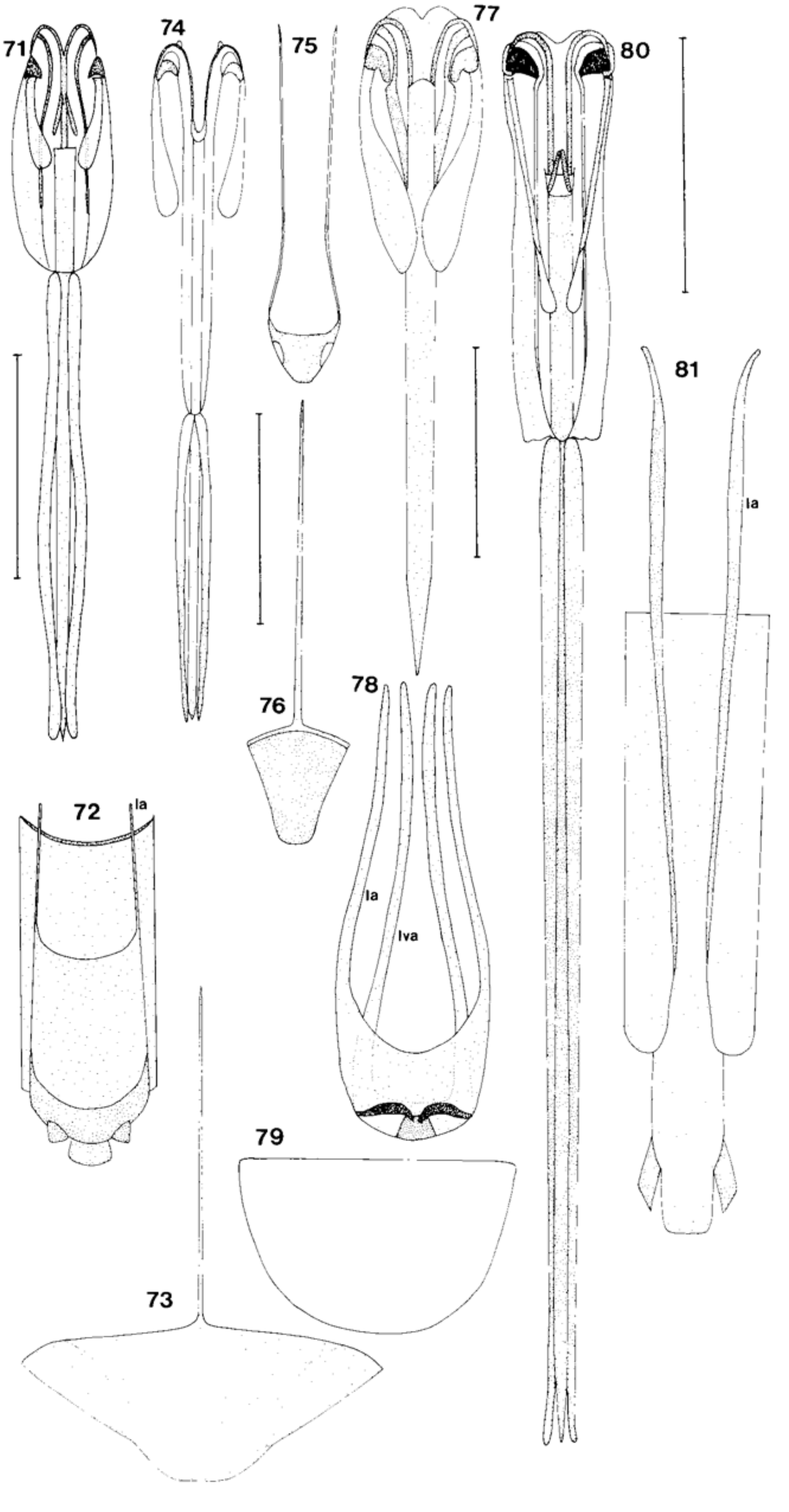
71
72
la
73
74
75
76
77
78
la
Iva
79
80
81
la

*Comments on Platyscelionini*

Although this monotypic tribe possesses many unique characters (see Masner 1976; Galloway and Austin 1984), the ovipositor system is telescopic, which clearly allies *Platyscelio* with the group of tribes that possesses the derived *Scelio*-type system, e.g. Scelionini, Calliscelionini and most Psilanteridini. The *Platyscelio* system has two unusual features: the 2nd gonocoxae are developed as a broad membranous plates and the lateral apodemes protrude proximally past the telescopic tube when at rest, as in *Aradophagus* and *Neoscelio*.

## Tribe **Doddiellini** Masner
### Genus ***Doddiella*** Kieffer
(Figs 88, 89)

*Diagnosis*

Ovipositor assembly and telescopic tube extended and retracted by hydrostatic pressure (i.e. *Scelio*-type system); telescopic tube somewhat delicate, (?) with 2 sections; ovipositor moderately elongate; proximal part of ventral membranous plate apparently absent; gonoplacs moderately elongate, 0·65× length of ovipositor; 2nd gonapophyses assembly simple; proximal arms short and slender, about 0·1× length of ovipositor; T7+8 fused, no sign of division between tergites, moderately well sclerotised compared with telescopic tube; cerci present, leaf-shaped, with long sensory hairs; lateral apodemes incorporated into wall of telescopic tube, extending for full length of tube, including T7+8 0·58× length of ovipositor; S6 without medial apodeme.

*Comments on Doddiellini*

Although externally *Doddiella* is one of the most unusual and distinct genera of scelionids, its ovipositor system is the standard *Scelio*-type and does not have any remarkable features. Masner (1976) and Galloway and Austin (1984) imply that the relationships of this genus are unknown, given its peculiar morphology and monotypic tribal status; however, the derived telescopic ovipositor system clearly aligns *Doddiella* with the Scelionini, Calliscelionini, most Psilanteridini, Aradophagini, Platyscelionini and Neoscelionini (Fig. 189).

## Tribe **Gryonini** Szabó
### Genus ***Gryon*** Haliday
(Figs 90–92)

*Diagnosis*

Ovipositor assembly extended and retracted by muscles (i.e. *Ceratobaeus*-type system), robust in form; ovipositor moderately elongate to elongate, 0·75–0·93× length of metasoma; proximal part of ventral membranous plate present; gonoplacs short, 0·4–0·5× length of ovipositor, with dense brush of sensillae on distal tips; 2nd gonapophyses assembly simple;

**Fig. 82.** Distal mesosoma of *Spiniteleia campbelli* Masner (Psilanteridini) showing telescopic tube partly extended. **Fig. 83.** Distal mesosoma of *Aradophagus fasciatus* Ashmead (Aradophagini) showing telescopic tube partly extended. **Figs 84, 85.** *Thoronella* sp. (Thoronini): *84*, dorso-lateral view of telescopic tube and ovipositor system partly extended; *85*, dorsal view of tergites 7+8 and and partly extended ovipositor system. **Figs 86, 87.** *Tiphodytes* sp. (Thoronini): *86*, lateral view of distal mesosoma and extended ovipositor assembly and tubular A9; *87*, dorsal view of tubular A9. Scale lines: *82, 84, 86*, 100 µm; *83, 85*, 50 µm; *87*, 50 µm. g, gonoplac; o, ovipositor; S6, metasomal sternites 6+7+8; tA9, tubular part of abdominal segment 9; T6, metasomal tergite 6; T7, metasomal tergites 7+8; tt, telescopic tube; 2gc, 2nd conocoxae.

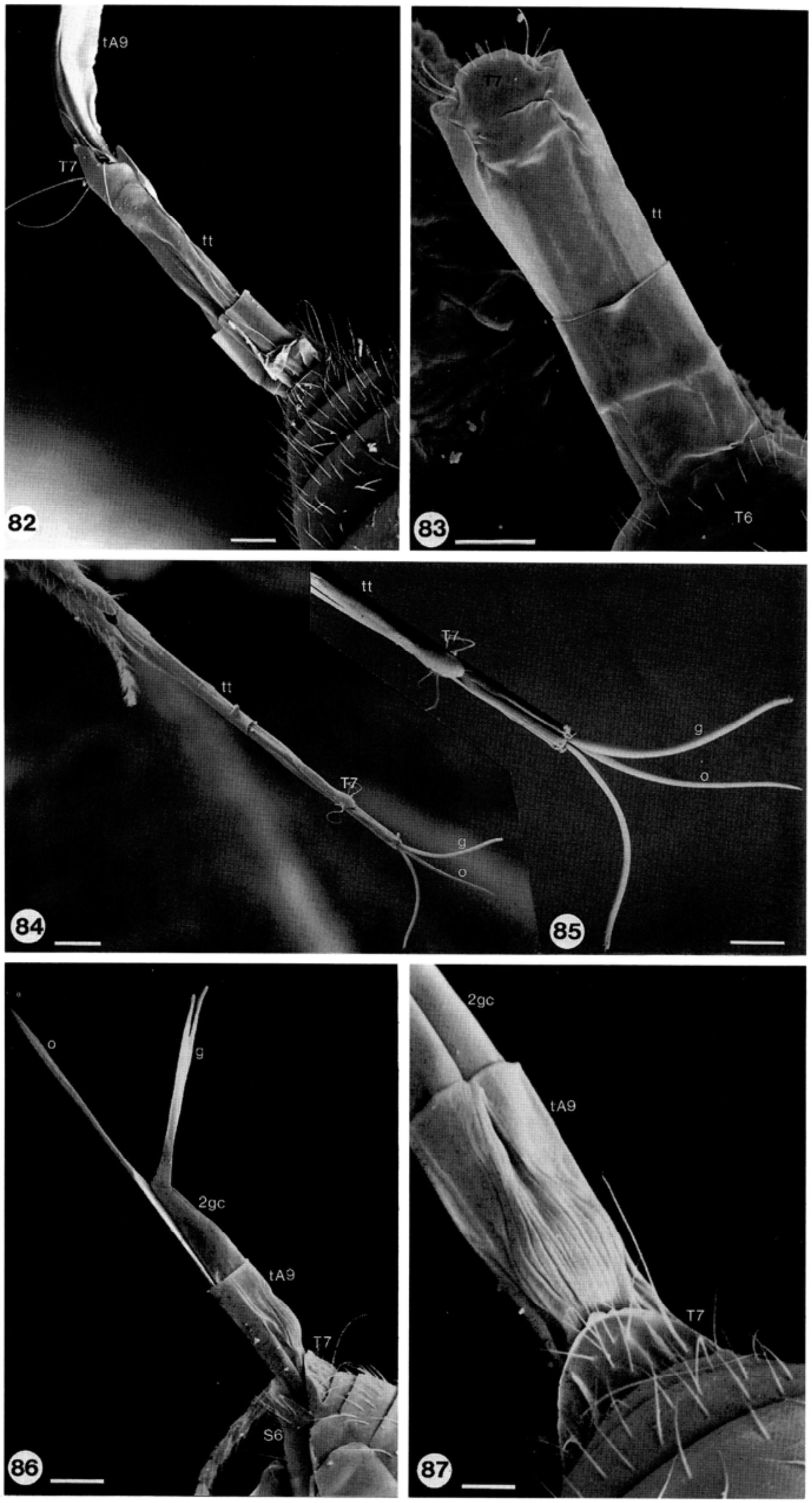
tA9
T7
tt
82
T7
tt
83
T6
tt
T7
tt
T7
g
o
g
o
84
85
o
g
2gc
tA9
T7
S6
86
2gc
tA9
T7
87

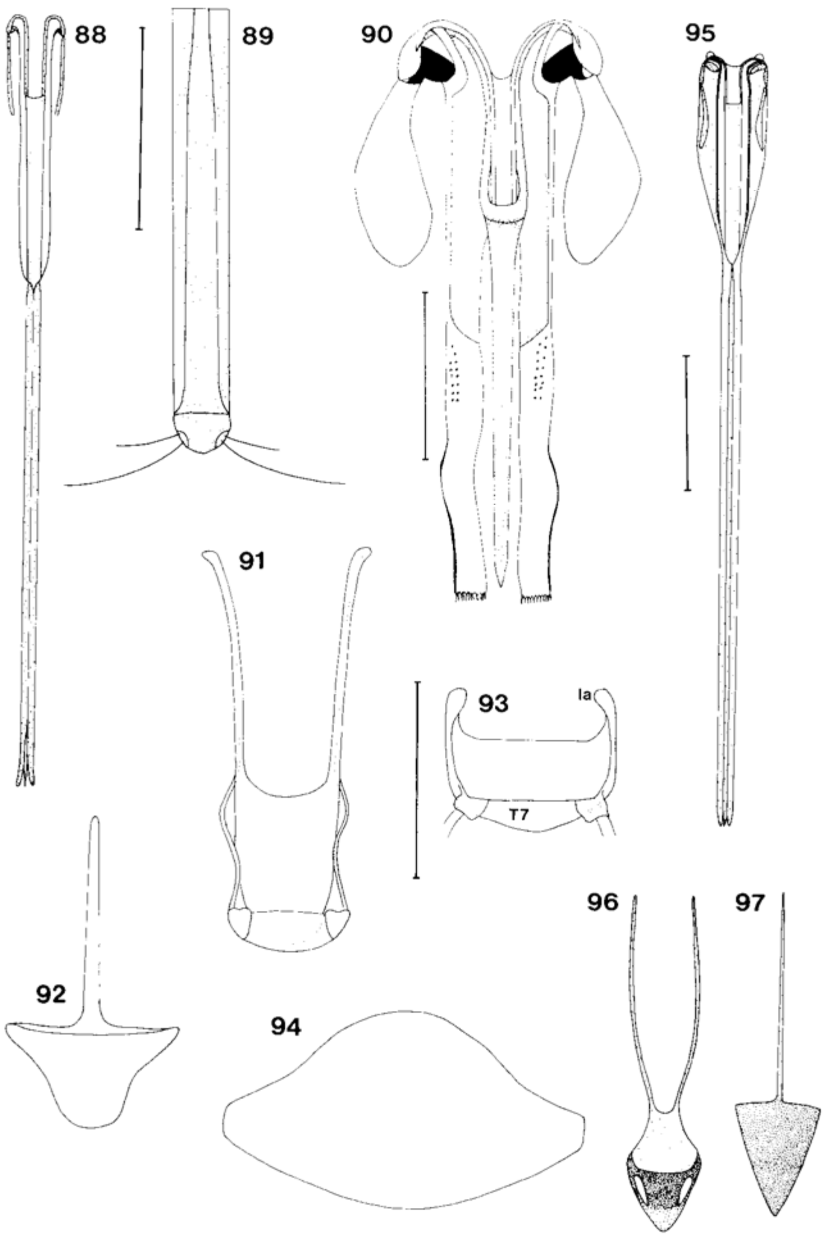


**Figs 88, 89.** *Doddiella* sp. (Doddiellini): *88*, ovipositor assembly; *89*, unextended telescopic tube with tergites 7+8. **Figs 90–92.** *Gryon pentatomus* (Dodd) (Gryonini): *90*, ovipositor assembly; *91*, tergites 7+8 and lateral apodemes; *92*, sternites 6+7+8 and medial apodeme. **Figs 93, 94.** *Neuroscelio doddi* Galloway, Austin & Masner (Gryonini): *93*, tergites 7+8 and lateral apodemes; *94*, sternites 6+7+8. **Figs 95–97.** *Thoron metallicus* (Haliday) (Thoronini): *95*, ovipositor assembly (detail of gonoplacs unknown); *96*, tergites 7+8 and lateral apodemes; *97*, sternites 6+7+8 and medial apodeme. Scale lines: *88*, *89*, 250 µm; *90–92*, 250 µm; *93*, *94*, 250 µm; *95–97*, 250 µm. la, lateral apodeme; T7, metasomal tergites 7+8.

proximal arms elongate and broad, 0·25–0·4× length of ovipositor; T7+8 fused, no sign of division between tergites, short and broad; cerci present, oval to leaf-shaped; lateral apodemes narrow and short, fused to more robust moderately elongate latero-ventral apodemes, including T7+8 apodemes 0·7–0·75× length of ovipositor, latero-ventral pair with distal part of

membranous plate attached; S6 with suture indicating division between S6 and S7+8, medial apodeme short to moderately elongate, including S6 0·55–0·65× length of ovipositor.

*Comments on Gryonini*

The ovipositor system of the Gryonini has been treated in some detail by Galloway *et al.* (1992) who have shown, without exception, that the group has a *Ceratobaeus*-type ovipositor system. It differs from most other tribes by the following characters: the ovipositor assembly is very robust; the gonoplacs are relatively short; the proximal arms are elongate to extremely elongate and very broad; and the lateral apodemes are narrow, and fused to the more robust elongate latero-ventral apodemes (Figs 90, 91). A notable feature of *Gryon* and *Dyscritobaeus* (= *Mirotelenomus* Dodd; Caleca 1992; Caleca and Mineo 1993) is that the lateral apodemes are markedly shorter and thinner than the latero-ventral apodemes to which they are fused. In other genera, except for *Neuroscelio*, both the lateral and latero-ventral apodemes are thin and rod-like.

*Neuroscelio* is the most unusual genus in this tribe; it has a semi-complex 2nd gonapophyses assembly, very short ovipositor (0·54× length of metasoma), very short flap-like apodemes, lacks a medial apodeme on S6 (none of which is seen in any other gryonine genera), and has a pronounced distal folding of the 2nd gonocoxae (Figs 93, 94) It is unclear from the arrangement of the apodemes whether they are homologous to the lateral or latero-ventral pair. The ovipositor system of *Breviscelio* Sundholm, *Encyrtoscelio* Dodd and *Dyscritobaeus* is less robust than that of *Gryon* and *Neuroscelio* but is similar in most other respects to *Gryon* (see figures in Galloway *et al.* 1992). *Gryon* and *Neuroscelio* are apparently unique among scelionid genera in having a dense brush of sensory hairs at the distal end of the gonoplacs (Fig. 90).

Tribe **Embidobiini** Kozlov

Genus ***Embidobia*** Ashmead

(Figs 98–100)

*Diagnosis*

Ovipositor assembly extended and retracted by muscles (i.e. *Ceratobaeus*-type system); ovipositor moderately elongate; proximal part of ventral membranous plate present; gonoplacs short, half length of ovipositor; 2nd gonapophyses assembly simple; proximal arms slender, short; 0·14× length of ovipositor T7+8 fused, no sign of division between tergites, well sclerotised; cerci present, leaf-shaped; lateral apodemes moderately elongate, including T7+8 0·65× length of ovipositor, distal part of ventral membranous plate attached; S6 large, with short medial apodeme, together about 0·45× length of ovipositor.

*Comments on Embidobiini*

Apart from having short gonoplacs and a short medial apodeme, *Embidobia* has a standard *Ceratobaeus*-type ovipositor system. Other genera examined but not illustrated here, namely *Embioctonus* Masner, *Endecascelio* Masner & Dessart and *Palaeogryon* Masner, have ovipositor systems almost identical to *Embidobia*, but with slight variation in the lengths of the gonoplacs, proximal arms and lateral apodemes. *Palaeogryon* Masner differs from the above genera in that the ovipositor assembly is more robust and superficially similar to *Trissolcus*. *Echthrodesis* generally has a shorter ovipositor (about 0·65× length of metasoma) and lacks a medial apodeme on S6, one of only six genera to have this character (see under *Fusicornia* and *Mantibaria*). However, on the basis of the host group (spider eggs) and the tridentate, convex form of the mandibles, *Echthrodesis* should be accommodated in the Baeini, not the Embidobiini. The body of this genus is highly modified, apterous and clothed in dense pilosity, all of which can be logically interpreted as adaptations for accessing its host, an intertidal spider that lay its eggs into a submerged silken nest (Masner 1968*b*).

## Tribe **Thoronini** Kozlov

### Genus ***Thoron*** Haliday

(Figs 95–97)

*Diagnosis*

Ovipositor assembly extended and retracted by muscles (i.e. *Ceratobaeus*-type system); ovipositor elongate, about 0·9× length of metasoma; proximal part of ventral membranous plate present; (?) gonoplacs elongate, about 0·7× length of ovipositor; 2nd gonapophyses assembly simple; proximal arms slender, very short, 0·08× length of ovipositor; T7+8 fused, no sign of division between tergites, well sclerotised; cerci large and leaf-shaped; lateral apodemes short, including T7+8 0·5–0·6× length of ovipositor, with moderately robust distal part of membranous plate attached; S6 with moderately short medial apodeme, including S6 half length of ovipositor.

### Genus ***Tiphodytes*** Bradley

(Figs 86, 87, 101, 102)

*Diagnosis*

Ovipositor assembly extended and retracted by muscles (i.e. *Ceratobaeus*-type system); ovipositor moderately elongate, 0·73× length of metasoma; proximal part of ventral membranous plate absent; 2nd gonapophyses assembly simple; gonoplacs short, 0·44× length of ovipositor; proximal arms slender, short, 0·11× length of ovipositor; tubular A9 with fine longitudinal striations; T7+8 fused, no sign of division between tergites, short, well sclerotised; cerci leaf-shaped; lateral apodemes elongate, 0·83× length of ovipositor including T7+8, distal part of ventral membranous plate present; S6 with moderately elongate medial apodeme.

*Comments on Thoronini*

Members of this tribe have probably been treated in more varied ways by previous authors than any other group of scelionid genera. Kieffer (1926) accommodated *Thoron* within the Baeinae, for which his concept was particularly amorphous. Later, Kozlov (1970) placed this genus into a separate tribe in the Baeinae, *Pseudanteris* Fouts as a tribe in the Scelioninae, and *Tiphodytes* as a tribe in the Telenominae. Hellén (1971) classified *Thoron* in the Anteridini along with *Anteris* and *Psilanteris*. However, Masner (1972*a*) extensively reviewed these genera, rejected the above taxonomic arrangements, which were based only on superficial resemblance (i.e. convergence), and placed them together on numerous antennal, mesosomal and metasomal characters (see Masner 1972*a*, 1976; Masner and Huggert 1979*a*). Also, several of these genera are associated with an unusual habitat, aquatic or semi-aquatic environs (see Table 1).

Members of this tribe all have a *Ceratobaeus*-type ovipositor system and, although they have no unique features, they are clearly different from the Telenominae. Apart from the two genera treated above, partial dissection of specimens of *Microthoron* Masner and *Pseudanteris* show their systems to be very similar to that in *Thoron*. They both have the same well-sclerotised and fused T7+8 and large robust ventral membranous plate connecting the lateral apodemes to this tergite. In *Microthoron* the ventral membranous plate is shorter and broader. The genus

---

**Figs 98–100.** *Embidobia* sp. (Embidobiini): *98*, ovipositor assembly; *99*, tergites 7+8 and lateral apodemes; *100*, sternites 6+7+8 and medial apodeme. **Figs 101, 102.** *Tiphodytes* sp. (Thoronini): *101*, ovipositor assembly; *102*, tergites 7+8 and lateral apodemes. **Figs 103–105.** *Baeus* sp. (Baeini): *103*, ovipositor assembly; *104*, tergites 7+8 and lateral apodemes; *105*, sternites 6+7+8 and medial apodeme. **Figs 106–108.** *Mirobaeoides setosus* Austin (Baeini): *106*, ovipositor assembly; *107*, sternites 6+7+8 and medial apodeme; *108*, tergites 7+8 and lateral apodemes. Scale lines: *98–100*, 250 μm; *101*, *102*, 125 μm; *103–105*, 125 μm; *104–108*, 125 μm. la, lateral apodeme; lva, latero-ventral apodeme.

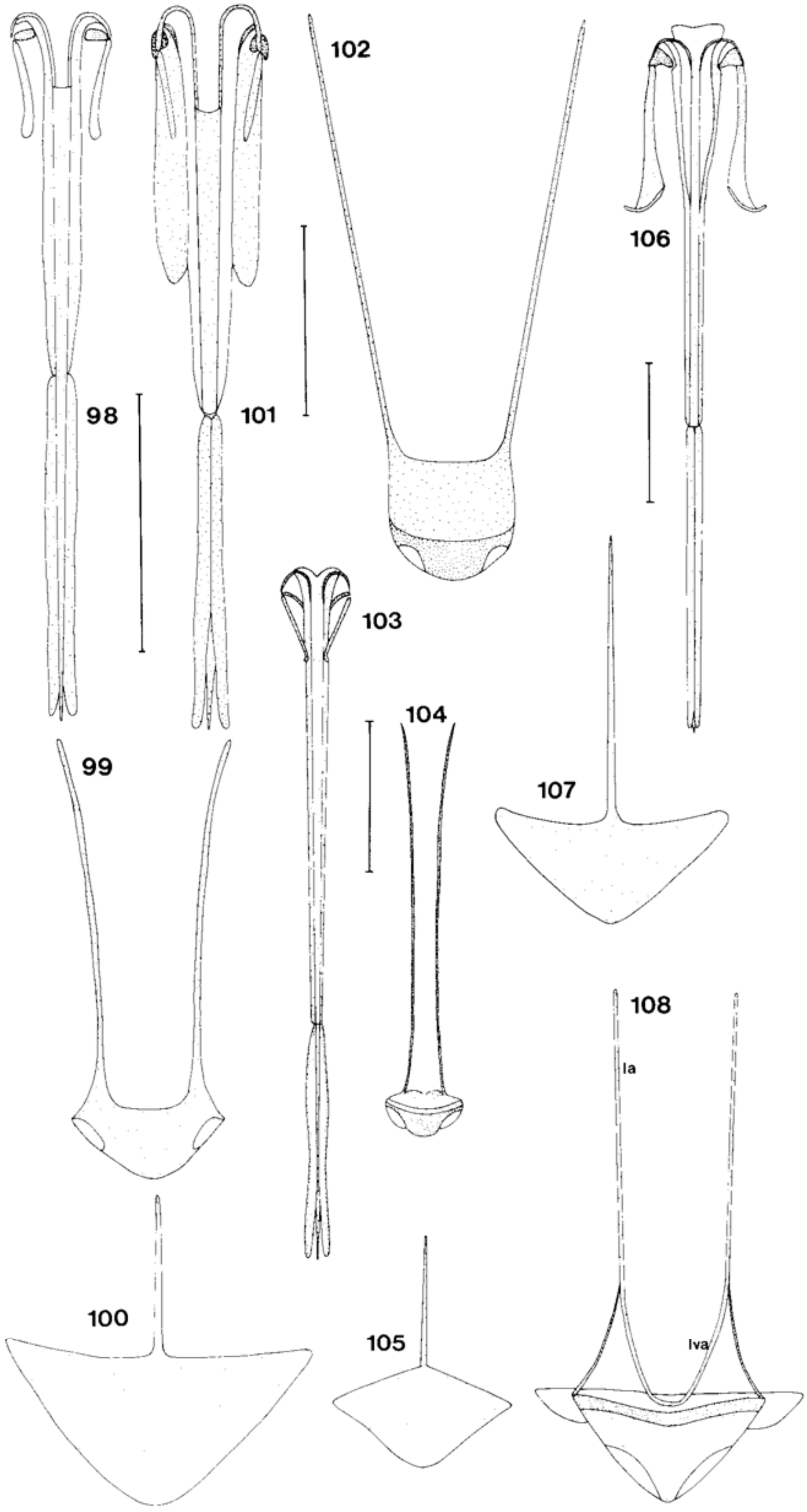
98
99
100
101
102
103
104
105
106
107
108
la
lva

*Thoronella*, originally placed in this tribe by Masner (1972*a*), was subsequently transferred to the Psilanteridini (Masner 1980*b*) where its telescopic and hydrostatically extended ovipositor system indicates that is correctly placed there (see Figs 84, 85). A possible relationship between the Psilanteridini and Thoronini, postulated by Masner (1972*a*, 1976), is unlikely given that these tribes differ completely in the form of the ovipositor system.

Tribe **Baeini** Ashmead

Genus ***Baeus*** Haliday

(Figs 103–105)

*Diagnosis*

Ovipositor assembly extended and retracted by muscles (i.e. *Ceratobaeus*-type system); ovipositor elongate, about 0·9× length of metasoma; proximal part of ventral membranous plate present; gonoplacs short, about 0·35× length of ovipositor; 2nd gonapophyses assembly absent; proximal arms slender, short, 0·1× length of metasoma; T7 distinct, represented as a sclerotised ring on anterior margin of T8; T8 short, well sclerotised; cerci leaf-shaped; lateral apodemes short, including T7+8 0·6× length of ovipositor, with distal part of ventral membranous plate connecting lateral apodemes, anterior margin of plate slightly more sclerotised; S6 broad, diamond-shaped, with short medial apodeme, 0·35× length of ovipositor including S6.

Genus ***Mirobaeoides*** Dodd

(Figs 106–108)

*Diagnosis*

Ovipositor assembly extended and retracted by muscles (i.e. *Ceratobaeus*-type system); ovipositor elongate, 0·95× length of metasoma; proximal part of ventral membranous plate present; gonoplacs short, 0·42× length of ovipositor; 2nd gonapophyses assembly absent; proximal arms moderately elongate, moderately broad, about 0·2× length of ovipositor; T7 distinct, represented as a sclerotised band on anterior margin of T8, attached to lateral apodemes; T8 well sclerotised; cerci leaf-shaped; lateral apodemes short, fused to latero-ventral apodemes, together including T7+8 apodemes 0·73× length of ovipositor, latero-ventral pair with distal part of ventral membranous plate attached; S6 broad, with fine anterior suture indicating division between S6 and S7+8, medial apodeme moderately short, including S6 about 0·55× length of ovipositor.

*Comments on Baeini*

The ovipositor system of the Baeini was examined by Austin (1983), but we have described here the nominal genus, *Baeus*, as well as *Mirobaeoides* which have not been treated

**Figs 109, 110.** *Neoscelio agilis* Dodd (Neoscelionini): *109*, ovipositor assembly; *110*, unextended telescopic tube with tergites 7+8. **Figs 111–113.** *Parascelio* sp. (unassigned): *111*, sternites 6+7+8 and medial apodeme; *112*, ovipositor assembly; *113*, tergites 7+8 and lateral apodemes. **Figs 114, 115.** *Trimorus caraborum* (Riley) (Teleasini): *114*, tergites 7 + 8 and lateral apodemes; *115*, ovipositor assembly. **Figs 116–117.** *Xenomerus* sp. (Xenomerini): *116*, ovipositor assembly; *117*, tergites 7+8 and lateral apodemes. **Figs 118–120.** *Trimorus canadensis* (Ashmead) (Teleasini): *118*, sternites 6+7+8 and medial apodeme; *119*, dorsal view of ovipositor system extended showing positions of tergites 7+8; *120*, as for Fig. 119, lateral view. Scale lines: *109*, *110*, 250 µm; *111–113*, 250 µm; *114*, *115*, 125 µm; *116–118*, 125 µm; *119*, *120*, 250 µm. mt, membranous tube; T6, metasomal tergite 6; T7, metasomal tergite 7; T8, metasomal tergite 8.

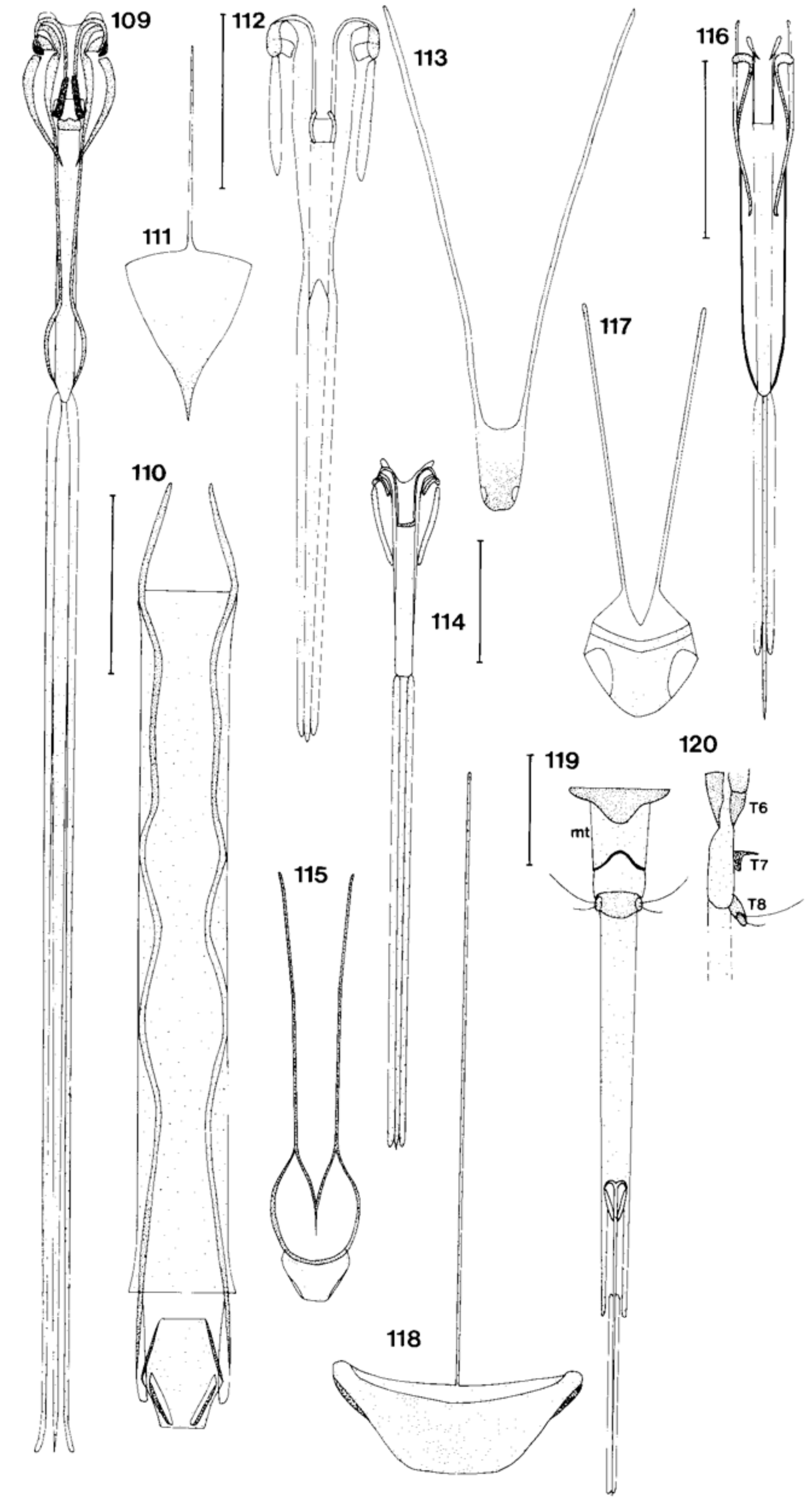
109
112
113
116
111
117
110
114
119
120
T6
mt
T7
T8
115
118

previously. Austin (1983) described the ovipositor system of *Ceratobaeus* (after which this system is named) in detail, and showed from dissections and behavioural observations that it is extended and retracted by antagonistic muscles attached to elongate apodemes, and not by hydrostatic pressure alone. Further, in this system T7+8 is exposed externally at rest, firmly attached to T6, and there is no expanded telescopic intersegmental membrane between these segments. The recognition and homology of some structures, particularly the proximal arms, 2nd gonocoxae and tubular A9 were given incorrectly in Austin (1983), but have been discussed and corrected in Field and Austin (1994).

Members of the Baeini represent a tightly knit group of genera that are well defined by several morphological characters (see Masner 1976; Galloway and Austin 1984; Austin 1986, 1988) and their biology: all are parasitoids of the eggs of spiders (Austin 1985; Table 1). *Baeus* and several other genera have an elongate ovipositor, often almost as long as the metasoma. However, *Ceratobaeus*, *Odontacolus* Kieffer and *Cyphacolus* Priesner have a horn developed on T1, which acts as a recess for an ovipositor that is much longer than the ventral length of the metasoma (see Austin 1983). In *Ceratobaeus* the ovipositor is virtually straight, but in *Odontacolus* it is looped back on itself inside the laterally compressed horn and is almost twice the ventral length of the metasoma. The mechanics of oviposition in the latter genus is unclear and requires further study. *Idris* Foerster, *Hickmanella* Austin and *Mirobaeus* Dodd have only a short to moderately elongate ovipositor (0·55–0·75× metasomal length) and lack a horn on T1. *Mirobaeoides* is exceptional in having lateral apodemes that are moderately short and fused to elongate latero-ventral apodemes (as in several gryonine genera; see Galloway *et al.* 1992). This genus also appears to have the most robust ovipositor system in that T7+8, S6 and the proximal arms are broad, and the proximal arms are more elongate. Substantial variation exists both within and between genera in the length of the proximal arms and the lateral and medial apodemes, although all members of the tribe appear to have relatively short gonoplacs (about half length of ovipositor or less). Some *Ceratobaeus* spp. with a large metasomal horn and elongate ovipositor (e.g. *C. cuspicornutus* Austin) are unique within the Scelionidae in that the lateral apodemes are fused in the distal part and divided (forked) proximally (Austin 1983).

ovipositor elongate; proximal part of ventral membranous plate absent; gonoplacs moderately elongate, 0·63× length of ovipositor; 2nd gonapophyses assembly complex; proximal arms moderately broad, moderately elongate, 0·16× length of ovipositor; T7+8 fused, no sign of division between tergites, well sclerotised, rounded posteriorly, at rest hidden under T6; cerci small, oval in shape; lateral apodemes moderately elongate, including T7+8 0·72× length of ovipositor, sharply diverging; distal part of ventral membranous plate greatly reduced or fused laterally with T7 and/or lateral apodemes; S6 sharply pointed distally, with moderately short medial apodeme, including S6 about half length of ovipositor.

*Comments on* Parascelio

This Neotropical genus has never been formally placed within the tribal classification developed for the family by Kozlov (1970) and Masner (1976). It has a number of very distinct morphological characters and was listed by Masner under 'genera incertae sedis'. The ovipositor system is of the normal *Ceratobaeus*-type but is modified in several ways. In particular, the lateral apodemes are completely fused to T7+8 and appear as a continuous elongate extension of this tergal plate. Also, the ovipositor assembly is robust and S6 is large and streamlined.

### Genus *Sceliacanthella* Dodd

(Figs 62, 63, 69)

*Diagnosis*

Ovipositor assembly and telescopic tube extended and retracted by hydrostatic pressure (i.e. *Scelio*-type system), moderately robust; telescopic tube membranous, elongate, with 3 sections; ovipositor elongate, about 0·8× length of metasoma; proximal part of ventral membranous plate absent; gonoplacs elongate, 0·72× length of ovipositor; 2nd gonapophyses assembly complex; proximal arms moderately slender, short, 0·1× length of ovipositor; (?) T7 separated from and overlying T8, slightly more sclerotised than telescopic tube; cerci present, elongate leaf-shaped; lateral apodemes incorporated into wall of telescopic tube, distinct, short, about half length of ovipositor; S6 without medial apodeme.

*Comments on* Sceliacanthella

This genus was assumed, on the basis of the structure of the antennae, metanotum, propodeum and wing venation, to be closely related to *Opisthacantha* (Galloway and Austin 1984), and it has previously been misidentified as this genus. However, because of the problems surrounding the limits of *Opisthacantha*, it was treated by Galloway and Austin as one of the three genera they listed as 'genera incertae sedis'. Irrespective of any external similarity to *Opisthacantha* (based partly on synplesiomorphic character states), *Sceliacanthella* clearly possesses a *Scelio*-type ovipositor system (Figs 63, 69) and, therefore, these genera are clearly not closely related. More so, the possession of a telescopic ovipositor system aligns *Sceliacanthella* with the Scelionini, Calliscelionini, most Psilanteridini, Aradophagini, Platyscelionini and Neoscelionini (Fig. 189). The ovipositor system of *Sceliacanthella* has no distinguishing characters, with the exception that the lateral apodemes are noticeably short, only about half the length of the ovipositor.

## Subfamily **Teleasinae**

### Tribe **Teleasini** Ashmead

### Genus ***Teleas*** Latreille

(Figs 124, 125)

*Diagnosis*

Ovipositor assembly extended and retracted by muscles (i.e. *Ceratobaeus*-type system), moderately delicate in form; ovipositor elongate, about 0·9× length of metasoma; proximal part of ventral membranous plate present, with fine rod-like structures extending proximally; gonoplacs elongate, 0·67× length of ovipositor; 2nd gonapophyses assembly simple; proximal arms slender, short, 0·1× length of ovipositor; T7+8 somewhat membranous, with several sclerotised teeth on posterior margin, T7+8 fused but T7 identifiable as a broad thickened margin proximal to T8; cerci large, leaf-like, with extremely long sensory hairs; lateral apodemes short, including T7+8 about half length of ovipositor, sharply diverging, attached to T7 by distal part of ventral membranous plate which is divided medially; S6 without medial apodeme.

### Genus ***Trimorus*** Foerster

(Figs 114, 115, 118–123)

*Diagnosis*

Ovipositor assembly extended and retracted by muscles (i.e. *Ceratobaeus*-type system); ovipositor elongate, about 0·8× length of metasoma; proximal part of ventral membranous plate present; gonoplacs elongate, about 0·7× length of metasoma; 2nd gonapophyses assembly simple; proximal arms slender, short, 0·11× length of ovipositor; T7+8 fused but T7 easily seen

as a sclerotised ring on proximal margin of T8, sometimes T8 extended from T6 by short exserted membranous tube with nail-like T7 attached to dorsal surface of tube and well separated from T8 (Figs 119, 120); T8 sometimes strongly curved so that cerci face ventro-laterally; cerci leaf-like to bud-like in shape, often with extremely long sensory hairs; lateral apodemes moderately elongate, with T7+8 about 0·65× length of ovipositor, sometimes curved outwards in distal part and connected by fork-like extensions that may be continuous with distal part of ventral membranous plate; S6 large, somewhat transverse, with elongate medial apodeme, including S6 equal to length of ovipositor.

*Comments*

See below.

Tribe **Xenomerini** Kozlov

Genus ***Xenomerus*** Walker

(Figs 116, 117)

*Diagnosis*

Ovipositor assembly extended and retracted by muscles (i.e. *Ceratobaeus*-type system); ovipositor elongate, about 0·8× length of metasoma; proximal part of ventral membranous plate indicated by elongate fine rod-like structures extending proximally; gonoplacs short, not reaching tip of ovipositor, about 0·45× length of metasoma; 2nd gonapophyses assembly simple; proximal arms moderately elongate, slender, 0·23× length of ovipositor; T7+8 fused but T7 identifiable as a broad thickened margin proximal to T8; cerci large, leaf-like; lateral apodemes diverging, connected by short lightly sclerotised distal part of ventral membranous plate; lateral apodemes short, including T7+8 about half length of ovipositor; S6 with suture indicating division between S6 and S7+8, medial apodeme very elongate, including S6 equal to length of ovipositor (sometimes S6 with medial apodeme absent).

Genus ***Gryonoides*** Dodd

(Figs 132, 133)

*Diagnosis*

Ovipositor assembly extended and retracted by muscles (i.e. *Ceratobaeus*-type system); ovipositor elongate, about 0·8× length of metasoma; proximal part of ventral membranous plate absent; gonoplacs moderately elongate, about 0·6× length of metasoma; 2nd gonapophyses assembly simple; proximal arms moderately elongate and broad, 0·15× length of ovipositor; T7+8 fused but T7 identifiable as a broad thickened margin proximal to T8; cerci large, leaf-like, with very long sensory hairs; lateral apodemes robust, broadly sinuate in shape, connected medially by transverse rod and posteriorly by lightly sclerotised part of ventral membranous plate; apodemes moderately elongate, including T7+8 0·66× length of ovipositor; S6 with moderately robust medial apodeme, including S6 0·7× length of ovipositor.

*Comments on Teleasinae*

The subfamily Teleasinae represent a closely knit group of genera that limited host records indicates has specialised in parasitising the eggs of Coleoptera, specifically members of the Carabidae (Table 1). With few exceptions, genera in this subfamily are morphologically similar to each other, often difficult to distinguish, and several appear to grade together. Masner (1976) recognises two generic groups within the Teleasini, the *Trimorus*-group and the more robust *Teleas*-group, the latter having long mandibles, a broad clypeus, and robust often specialised legs. However, Masner (1976) indicates that some elements of the largest and most diffuse genus, *Trimorus*, show transitions to *Teleas*, *Trisacantha* Ashmead, *Gryonoides* and

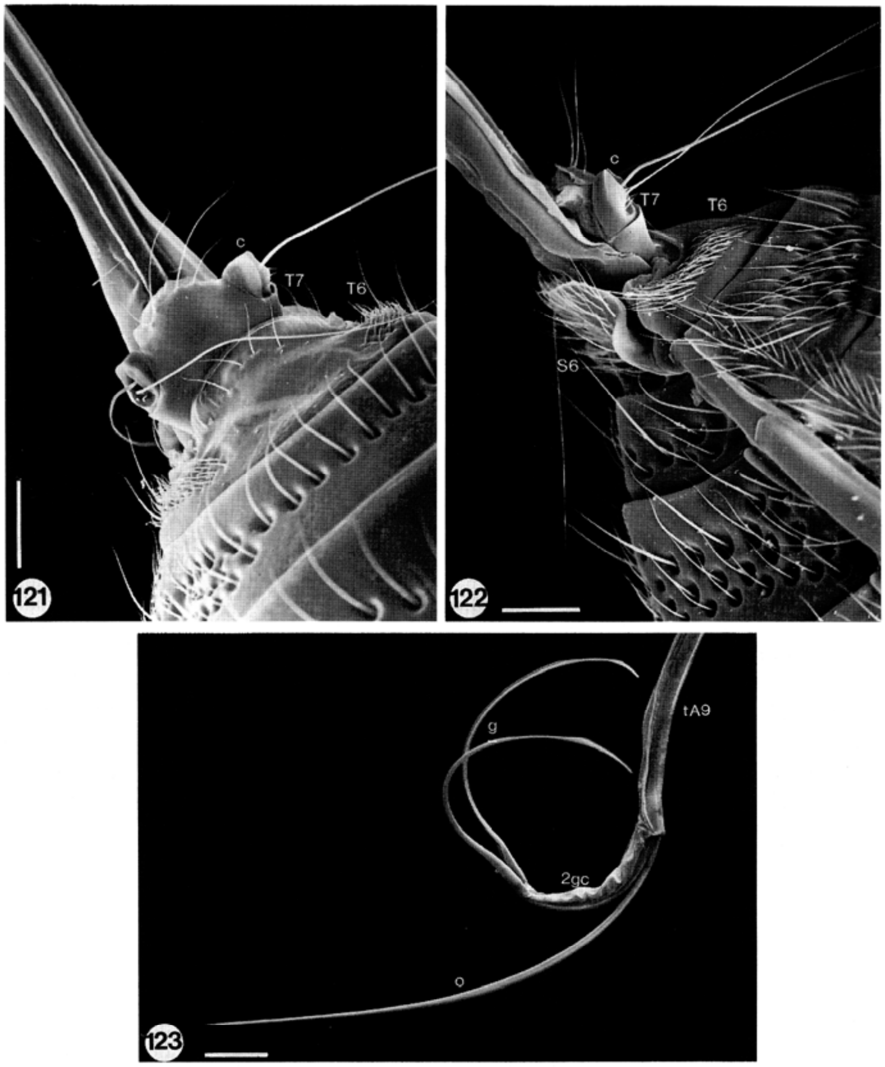


**Figs 121–123.** *Trimorus* sp. (Teleasini): *121*, dorsal view of distal metasoma showing tergites 7+8 with cerci and extended tubular A9; *122*, lateral view of distal metasoma showing tergites 7+8 with cerci and extend tubular A9; *123*, extended ovipositor assembly. Scale lines: *121*, *122*, 50 µm; *123*, 100 µm. g, gonoplac; o, ovipositor; S6, metasomal sternites 6+7+8; tA9, tubular part of abdominal segment 9; T6, metasomal tergite 6; T7, metasomal tergites 7 + 8; 2gc, 2nd gonocoxae.

*Xenomerus*, indicating that the two tribes may not be well supported. Indeed, Masner (1976) maintains the subfamily status of the Teleasinae because of its unusual host relationships, but also speculates that it may be better treated as a tribe within the Scelioninae. Given that the latter subfamily is probably polyphyletic and the most unusual hosts of scelionids are those of the Baeini (viz. spider eggs, Table 1), which are currently placed within the Scelioninae, there is obviously much that needs to be done to resolve the phylogenetic position of the Teleasinae.

Unfortunately, the structure of the ovipositor system sheds little light on the above problems, although at the generic level it has revealed some important differences. In particular, the ovipositor system of *Trimorus* spp. examined shows substantial variation, as much or more than

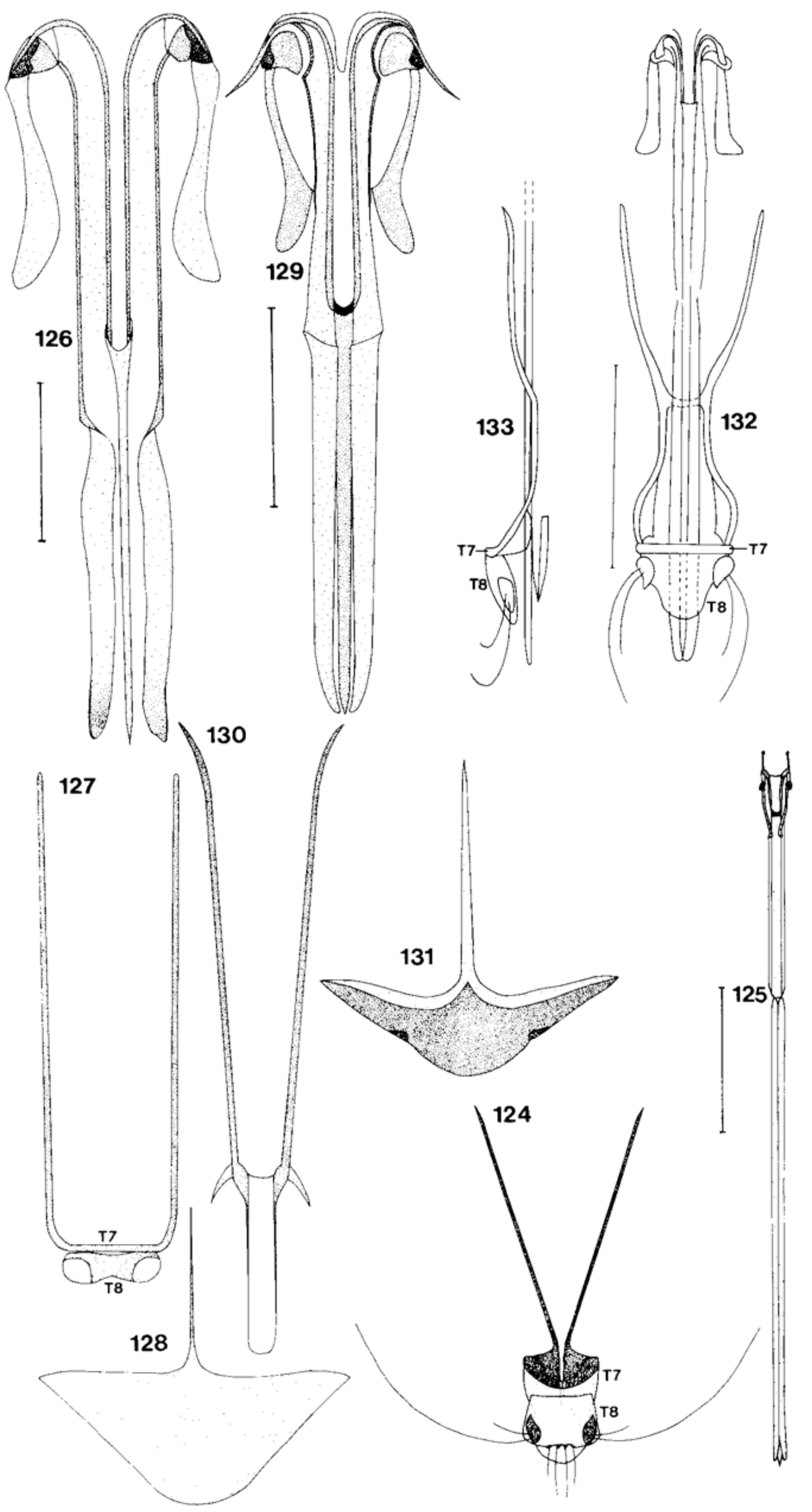
126
129
133
132
T7
T8
130
127
131
125
124
128

that between many scelionine tribes. The extension of T8 at the end of a short membranous tube and clear separation between T7 and T8 in *T. canadensis* (Ashmead) (Figs 119, 120), compared with the strong attachment between these tergites and the unusual arrangement of the lateral apodemes in other *Trimorus* spp. (Fig. 115), indicate that the genus may not be monophyletic. This supports in part Masner's (1976) statement that this is 'a very large genus with vague limits'. The short but extendible tube of *T. canadensis* can be interpreted as an early transitional stage between the *Ceratobaeus*- and *Scelio*-type ovipositor systems, except in *Trimorus* it may very well have evolved independently.

The other genera examined are equally diverse although all have a *Ceratobaeus*-type system. In *Gryonoides* the ovipositor system is among the most robust of any genus in the family (cf. *Gryon*), while *Xenomerus* is peculiar because the gonoplacs do not reach the distal end of the ovipositor and the proximal arms are elongate and slender. Interestingly, *Xenomerus* and *Teleas* share a unique synapomorphy within the family: the proximal part of the ventral membranous plate has fine rod-like structures extending anteriorly (Figs 116, 125). Also, of potential phylogenetic significance is that all teleasine genera examined (except *T. canadensis*) have T7 clearly delineated from T8 and fused to each other.

Subfamily **Telenominae**

Tribe **Telenomini** Thomson

Genus ***Telenomus*** Haliday

(Figs 134–136)

*Diagnosis*

Ovipositor assembly extended and retracted by muscles (i.e. *Ceratobaeus*-type system); ovipositor elongate, about 0·8× length of metasoma; proximal part of ventral membranous plate absent; gonoplacs short to moderately elongate, sometimes broad, 0·50–0·6× length of ovipositor; 2nd gonapophyses assembly simple; proximal arms slender, moderately elongate, 0·2× length of ovipositor; T7+8 moderately well sclerotised, sometimes fused with no sign of division between tergites and lateral apodemes attached to T7 by distal part of ventral membranous plate (Fig. 135); alternatively, T7 distinct, represented as a continuous sclerotised U-shaped rod-like connection between lateral apodemes anterior to T8, distal part of ventral membranous plate absent (as in *Trissolcus*, Fig. 127); cerci present, variable in shape, often plate- or leaf-shaped; lateral apodemes elongate, including T7+8 about 0·8× length of ovipositor; S6 often large, medial apodeme present but variable in length, including S6 often as short as half length of ovipositor.

Genus ***Trissolcus*** Ashmead

(Figs 126–128)

*Diagnosis*

Ovipositor assembly extended and retracted by muscles (i.e. *Ceratobaeus*-type system), moderately robust in form; ovipositor moderately elongate to elongate, 0·7–0·9× length of

**Figs 124, 125.** *Teleas neptuni* Sharkey (Teleasini): *124*, tergites 7+8 and lateral apodemes; *125*, ovipositor assembly. **Figs 126–128.** *Trissolcus oenone* (Dodd) (Telenomini): *126*, ovipositor assembly; *127*, tergites 7+8 and lateral apodemes; *128*, sternites 6+7+8 and medial apodeme. **Figs 129–131.** *Psix lacunatus* Johnson & Masner (Telenomini): *129*, ovipositor assembly; *130*, tergites 7+8 and lateral apodemes; *131*, sternites 6+7+8 and medial apodeme. **Figs 132, 133.** *Gryonoides* sp. (Xenomerini): *132*, dorsal view of ovipositor assembly, tergites 7+8 and lateral apodemes; *133*, lateral view of same specimen as in Fig. 132. Scale lines: *124*, *125*, 250 µm; *126–128*, 125 µm; *129–131*, 125 µm; *132*, *133*, 0·5 mm. T7, metasomal tergite 7; T8, metasomal tergite 8.

metasoma; ventral membranous plate absent; gonoplacs short to elongate, sometimes broad, 0·45–0·65× length of ovipositor, folded proximally near attachment with 2nd gonocoxae, often more heavily sclerotised distally; 2nd gonapophyses assembly simple; proximal arms elongate and broad, 0·3× length of ovipositor; T7 distinct, represented as a continuous sclerotised U-shaped rod-like connection between lateral apodemes on anterior margin of T8; T8 well sclerotised, short and broad; cerci modified as oval sensory plates; lateral apodemes moderately elongate to elongate, including T7+8 apodemes 0·65–0·8× length of ovipositor; distal part of ventral membranous plate apparently absent; S6 large, with short fine medial apodeme, including S6 about 0·45–0·5× length of ovipositor.

### Genus ***Psix*** Kozlov & Le

(Figs 129–131)

*Diagnosis*

Ovipositor assembly extended and retracted by muscles (i.e. *Ceratobaeus*-type system), moderately robust in form; ovipositor elongate, about 0·8× length of metasoma; proximal part of ventral membranous plate present but faint; gonoplacs short and broad, about half length of ovipositor; 2nd gonapophyses assembly simple; proximal arms elongate, broad at base, about 0·25× length of ovipositor; T7+8 narrow, elongate, somewhat membranous, without distinct division between tergites; cerci indistinct; lateral apodemes elongate, not obviously connected by distal part of membranous plate, including T7+8 apodemes about 0·9× length of ovipositor, with membranous spine-like processes projecting posteriorly from base; S6 short and broad, with fine anterior suture indicating division between S6 and S7+8, medial apodeme short, including S6 about 0·45× length of ovipositor.

*Comments on Telenominae*

The Telenominae represents one of the well-recognised monophyletic groups within the Platygastroidea, and is supported by numerous synapomorphies, particularly associated with the metasoma (see Masner 1976; Johnson 1984*a*; Johnson and Masner 1985). Although the overall structure of the ovipositor provides no obvious additional synapomorphy for the subfamily, as all genera possess the more plesiomorphic *Ceratobaeus*-type system, it does shows a number of interesting characteristics that may be important in resolving generic-level relationships. Generally, the subfamily can be divided into two groups based on their host relationships: robust species (e.g. *Trissolcus, Psix, Paratelenomus* Dodd, *Telenomus podisi*-group) that parasitise the eggs of Heteroptera, and more delicate species that attack lepidopteran, and more rarely dipteran, neuropteran and heteropteran, eggs (e.g. other *Telenomus* groups, *Nirupana* Nixon, *Eumicrosoma* Gahan) (Johnson 1984*a*; Table 1). Our examination of several genera indicates that the former robust group generally has an ovipositor system (Figs 126, 129) that is stouter than that of the latter more delicate group, thus indicating a correlation between the morphology of the ovipositor system and broad host relationships, as in the Baeini (see Austin 1985). Significantly, *Phanuropsis* Girault (not illustrated) tends to be intermediate in structure between these groups; that is, the ovipositor, proximal arms and lateral apodemes are similar to those of some *Telenomus* (species that are more delicate than that shown in Figs 134–136), while the 2nd gonapophyses assembly extends to the middle of the ovipositor, and the gonoplacs are folded proximally and sclerotised distally, as in *Trissolcus* (Fig. 126). Other genera examined but not diagnosed above are *Eumicrosoma* and *Paratelenomus*. Both have an ovipositor system that is very similar to *Telenomus*, but that differs very slightly in the relative lengths of the lateral apodemes.

All genera dissected have a relatively short medial apodeme on S6, elongate robust proximal arms, and moderately short gonoplacs. The only departure from this is known in some *Phanuromyia* Dodd from the Neotropics (CNCI, not dissected), where the robust gonoplacs are extremely elongate (about 0·9× length of ovipositor) and the ovipositor is apparently permanently exserted from the metasoma.

Telenomines differ most noticeably in the structure of T7+8. In some *Telenomus* the arrangement of these tergites, the lateral apodemes and distal part of the ventral membranous

plate are similar to most other scelionine genera that have a *Ceratobaeus*-type system (Fig. 135). However, they differ substantially in *Trissolcus*, *Psix* and other species of *Telenomus* (not figured here). In *Trissolcus* and some *Telenomus* the lateral apodemes are linked, apparently through T7, which is U-shaped and rod-like, in a similar way to most platygastrids (see below), and T8 is greatly shortened and separated from T7 (Fig. 127). In *Psix* T7+8 are more membranous, greatly elongated and there are membranous spine-shaped processes projecting posteriorly from the base of the lateral apodemes (Fig. 130).

This arrangement of T7 being rod-like and linking the lateral apodemes was first described for *Trissolcus flavipes* (Thomson) by Oeser (1961) and later was assumed to be universal for the Telenominae by Masner and Huggert (1979*a*). However, results here indicate that it is not common to all genera or, indeed, to species within a single genus (e.g. *Telenomus*), although it is clearly widespread across the subfamily. To assess the full importance of this and other characters in developing a phylogeny for the Telenominae and scelionids in general, further comparative study on a larger range of species than has been undertaken here will be required.

## Ovipositor System of the Platygastridae

### Subfamily **Sceliotrachelinae**

***Fidiobia*-cluster**

Genus ***Fidiobia*** Ashmead

(Fig. 137)

*Diagnosis*

Ovipositor assembly extended and retracted by muscles (i.e. *Ceratobaeus*-type system), delicate in form; ovipositor elongate, as long as metasoma; proximal part of ventral membranous plate absent; gonoplacs very short, 0·22× length of metasoma, narrow proximally, broadening distally; 2nd gonapophyses assembly complex; proximal arms very slender, moderately elongate, 0·16× length of ovipositor; lateral apodemes joined by U-shaped rod-like T7, apodemes very short, about 0·1× length of ovipositor, (?) with distal part of ventral membranous plate attached; latero-ventral apodemes independent of S6, forming a second U-shaped structure, attached to lateral apodemes at proximal ends; T8 absent; S6 simple.

Genus ***Platygastoides*** Dodd

(Figs 138–140)

*Diagnosis*

Ovipositor assembly extended and retracted by muscles (i.e. *Ceratobaeus*-type system), moderately delicate in form; ovipositor moderately elongate, about 0·75× length of metasoma; (?) proximal part of ventral membranous plate present; gonoplacs short, 0·45× length of ovipositor; 2nd gonapophyses assembly complex; proximal arms slender, short, 0·1× length of ovipositor; lateral apodemes joined by U-shaped rod-like T7, apodemes very short, about 0·15× length of ovipositor, with distal part of ventral membranous plate attached; latero-ventral apodemes independent of S6, forming a second U-shaped structure, partly desclerotised medially, attached to lateral apodemes at proximal ends; T8 absent; S6 simple.

Genus ***Zelandonota*** Masner & Huggert

(Figs 141, 142)

*Diagnosis*

Ovipositor assembly extended and retracted by muscles (i.e. *Ceratobaeus*-type system); ovipositor elongate, 0·8× length of metasoma; proximal part of ventral membranous plate

present, divided into two strips distally; gonoplacs elongate, about 0·7× length of ovipositor; 2nd gonapophyses assembly complex; proximal arms apparently continuous with membranous tube surrounding ovipositor, reaching almost to ovipositor tip; lateral apodemes joined by U-shaped rod-like T7, apodemes very short, diverging proximally, about 0·3× length of ovipositor, with small membranous plate attached postero-medially (possibly a remnant of T8, cerci absent); latero-ventral apodemes independent of S6, forming a second U-shaped structure, attached to lateral apodemes at proximal ends; S6 simple.

*Comments on* Fidiobia-*cluster*

This group of eight genera was united on the basis of females having an abrupt but segmented antennal clava, lateral ocelli moderately close to the eye margins, and the fore tibial spur without a comb (Masner and Huggert 1989). Of these, three genera (*Neobia* Masner & Huggert, *Oligomerella* Masner & Huggert and *Plutomerus* Masner & Huggert) are monotypic or contain very few species, and five are known from numerous species, although the two endemic New Zealand genera (*Zelamerus* Masner & Huggert and *Zelandonota*) have only a single species so far described. Where known (two genera), members of this tribe are parasitoids of beetle eggs (Table 1). The ovipositor systems of the three most speciose genera (*Fidiobia*, *Platygastoides* and *Zelandonota*) were examined here and show the most homogenous system of any platygastrid group, in that the lateral and latero-ventral apodemes comprise two U-shaped structures that are attached at the proximal ends (Fig. 140). Further, the latero-ventral apodemes are separate from S6, which is a simple sternite, and the ovipositor assembly is generally somewhat delicate in form rather than robust. These genera differ in the lengths of the proximal arms, gonoplacs and lateral apodemes. In *Zelandonota* T7 (represented as the transverse element of the U-shaped structure) has a membranous plate attached postero-medially and this may be homologous to a greatly reduced T8 (see Comments below).

## ***Aphanomerus*-cluster**

### Genus ***Aphanomerus*** Perkins

(Figs 143–145)

*Diagnosis*

Ovipositor assembly extended and retracted by muscles (i.e. *Ceratobaeus*-type system); ovipositor elongate, about 0·75× length of metasoma; proximal part of ventral membranous plate present; gonoplacs short, folded and not reaching to tip of ovipositor, 0·4× length of ovipositor; 2nd gonapophyses assembly complex, with lateral processes elongate anteriorly; proximal arms moderately elongate, 0·15× length of ovipositor, broad, continuous with membranous tube surrounding ovipositor, extending over half length along ovipositor; lateral apodemes joined by U-shaped rod-like T7, very short, about 0·2× length of ovipositor, with small membranous plate attached postero-medially (possibly a remnant of T8 with cerci absent), proximal ends with small membranous flange-like structures; S6 triangular, with 2 short latero-ventral apodemes, including S6 about 0·25× length of ovipositor.

### Genus ***Austromerus*** Masner & Huggert

(Figs 146–148)

*Diagnosis*

Ovipositor assembly extended and retracted by muscles (i.e. *Ceratobaeus*-type system), moderately robust in form; ovipositor elongate, (?) 0·8× length of metasoma; proximal part of ventral membranous plate present; gonoplacs elongate but connection between gonoplacs and 2nd gonocoxae indistinct; 2nd gonapophyses assembly complex; proximal arms moderately elongate, 0·19× length of ovipositor, broad, continuous with membranous tube surrounding ovipositor; lateral apodemes joined by U-shaped rod-like T7, very short, about 0·3× length of

ovipositor, slightly diverging at proximal ends, without small membranous plate attached postero-medially; T8 absent; S6 broadly triangular, with 2 short latero-ventral apodemes, including S6 about 0·3× length of ovipositor.

### Genus ***Parabaeus*** Kieffer

(Figs 149, 150)

*Diagnosis*

Ovipositor assembly extended and retracted by muscles (i.e. *Ceratobaeus*-type system), moderately robust in form; ovipositor elongate to extremely elongate, 0·85× length of metasoma or slightly longer than ventral metasoma when horn on T1 present; proximal part of ventral membranous plate attached to proximal head, divided into two strips distally; gonoplacs very short, about 0·3× length of ovipositor; 2nd gonapophyses assembly complex; proximal arms moderately elongate and robust, 0·18× length of ovipositor; lateral apodemes joined by broad U-shaped rod-like T7, very short, about 0·3× length of ovipositor, diverging proximally, without small membranous plate attached postero-medially; T8 absent; S6 simple, independent latero-ventral apodemes or those attached to S6 absent.

*Comments on* Aphanomerus*-cluster*

The genera in this group contain few described species (six genera are monotypic), but numerous undescribed taxa are known in collections (Masner and Huggert 1989). The group is characterised by a trend in the form of the antenna, from a clava that is clearly segmented to one that is abrupt and unsegmented. The ovipositor assembly is also reasonably uniform for the group in that it is moderately delicate in most genera, although for *Parabaeus* it is more robust. However, the significant feature of this generic cluster is the presence of two latero-ventral apodemes that are fused to the proximal margin of S6+7+8 and do not connect to the lateral apodemes (Figs 145, 147). This character is present in several genera examined, including *Aphanomerella* Dodd and *Tetrabaeus* Kieffer (not figured or diagnosed here), but it is absent in *Parabaeus* and probably *Helava* Masner & Huggert (not clear in dissection), indicating that these genera are possibly misplaced in the *Aphanomerus*-cluster. Outside of this generic cluster, S6 with fused latero-ventral apodemes is known for only one other genus, *Metaclisis* Foerster (Figs 159, 160) in the *Proplatygaster*-cluster (Platygastrinae).

Similar to most other generic groups within the Platygastroidea, several ovipositor characters are variable among genera of this cluster: the lengths of the ovipositor assembly, gonoplacs and proximal arms. *Aphanomerus* stands alone among platygastrid genera in that the ovipositor extends well past the gonoplacs by the full length of the latter. *Aphanomerella* and *Helava* were very similar to *Aphanomerus* but the gonoplacs are relatively longer. *Parabaeus* is one of only very few platygastrid genera (and then only in some species) to have T1 developed into a hump or horn to accommodate an ovipositor that is as long or longer than the metasoma (see Comments below on *Inostemma* Haliday). Although our dissection of *Tetrabaeus* revealed that this genus has latero-ventral apodemes on S6+7+8, the species examined was very small and other features of the ovipositor system were not clear enough to be described.

## ***Isolia***-cluster

### Genus ***Isolia*** Foerster

(Fig. 151)

*Diagnosis*

Ovipositor assembly extended and retracted by muscles (i.e. *Ceratobaeus*-type system), moderately delicate in form; ovipositor moderately elongate, (?) about 0·7× length of metasoma; proximal part of ventral membranous plate apparently absent; gonoplacs short to elongate, 0·45–0·7× length of ovipositor, not clearly delineated from 2nd gonocoxae, not reaching to tip of

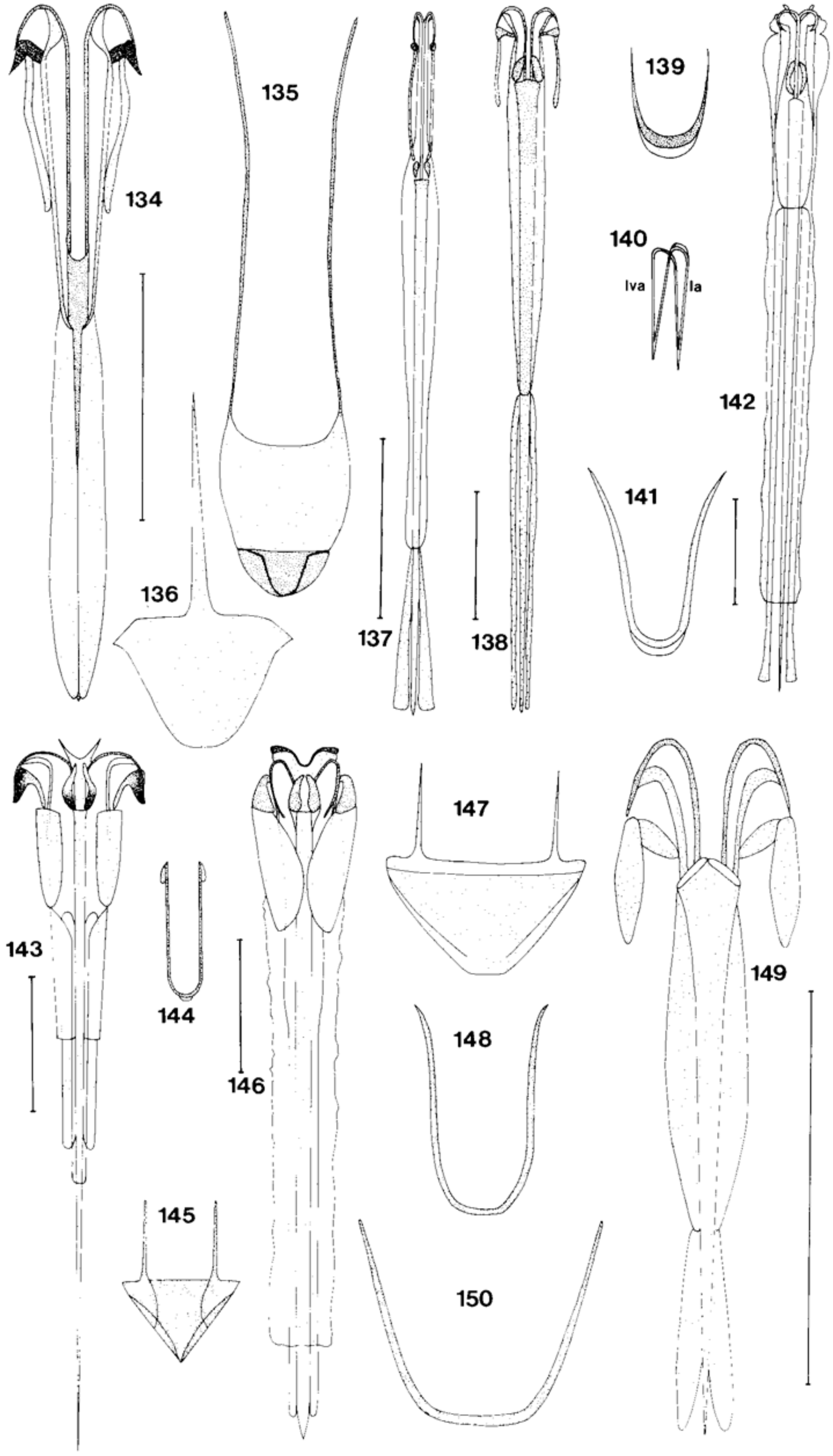
134
135
136
137
138
139
140
Iva
Ia
141
142
143
144
145
146
147
148
149
150

ovipositor; 2nd gonapophyses assembly simple; proximal arms curved and broadened distally, short to moderately elongate, 0·11–0·2× length of ovipositor, without small membranous plate attached postero-medially; lateral apodemes possibly present, but if so very short (not evident in dissection); T8 absent; 6 simple, independent latero-ventral apodemes or those attached to S6 absent.

*Comments on* Isolia-*cluster*

This group of four relatively small genera is moderately well characterised on the basis of external morphological characters: specialised mandibles, fore tibial spurs with combs, and the ocelli in a compact group distant from the eye margin (Masner and Huggert 1989). Unfortunately, the genera in this cluster, with the exception of *Isolia*, are moderately rare in collections and we did not have access to material for dissection. In particular, the ovipositor system of *Sceliotrachelus* Brues will be important to examine, as this genus has been previously treated as a chalcidoid, a scelionid, and a playgastrid with varying relationships (see Masner 1964; Kozlov 1970; Masner and Huggert 1989). The two specimens of *Isolia* dissected revealed a somewhat aberrant ovipositor system, in that the gonoplacs are more-or-less continuous and fused with the 2nd gonocoxae (Fig. 151), although this needs to be confirmed by further dissections, as does the form of the lateral apodemes, if present. Nevertheless, even if this is so, such a character may be of importance only at the generic level.

## ***Amitus*-cluster**

### Genus ***Amitus*** Haldeman

(Figs 152, 153)

*Diagnosis*

Ovipositor assembly extended and retracted by muscles (i.e. *Ceratobaeus*-type system), very broad and robust in form; ovipositor moderately elongate, (?) about 0·7× length of metasoma; proximal part of ventral membranous plate absent; gonoplacs thin, short, half length of ovipositor; 2nd gonapophyses assembly simple; 2nd gonapophyses divided and extending into rami very low on ovipositor shaft, about one-third distance from distal end of ovipositor tip; proximal arms elongate, 0·32× length of ovipositor, broad proximally, tapering distally; lateral apodemes joined by strongly diverging U-shaped rod-like T7, very short, 0·25× length of ovipositor, broadened postero-medially into short partly sclerotised plate (possibly including remnant of T8 with cerci absent); S6 simple, independent latero-ventral apodemes or those attached to S6 absent.

*Comments on* Amitus-*cluster*

This moderately small cluster of four genera is dominated by *Amitus* which is virtually cosmopolitan and known from numerous species. The other three genera, *Nanomerus* Masner & Huggert which is known only from Chile, *Alfredella* Masner & Huggert which is endemic to Australia, and *Aleyroctonus* Masner & Huggert, are monotypic, but the latter two are also known from several undescribed species. Host data are available for *Amitus* and *Aleyroctonus*, which are the only platygastrids known to parasitise Aleyrodidae (Table 1). Members of this group possibly

**Figs 134–136.** *Telenomus* sp. (Telenomini): *134*, ovipositor assembly; *135*, tergites 7+8 and lateral apodemes; *136*, sternites 6+7+8 and medial apodeme. **Fig. 137.** Ovipositor assembly of *Fidiobia* sp. (*Fidiobia*-cluster). **Figs 138–140.** *Platygastoides* sp. (*Fidiobia*-cluster): *138*, ovipositor assembly; *139*, lateral apodemes; *140*, lateral view showing lateral and latero-ventral apodemes attached proximally. **Figs 141, 142.** *Zelandonota* sp. (*Fidiobia*-cluster): *141*, lateral apodemes; *142*, ovipositor assembly. **Figs 143–145.** *Aphanomerus* sp. (*Aphanomerus*-cluster): *143*, ovipositor assembly; *144*, lateral apodemes; *145*, sternites 6+7+8 and latero-ventral apodeme. **Figs 146–148.** *Austromerus* sp. (*Aphanomerus*-cluster): *146*, ovipositor assembly; *147*, sternites 6+7+8 and latero-ventral apodeme; *148*, lateral apodemes. **Figs 149, 150.** *Parabaeus peckorus* Austin (*Aphanomerus*-cluster): *149*, ovipositor assembly; *150*, lateral apodemes. Scale lines: *134–136*, 125 µm; *137*, 125 µm; *138–140*, 125 µm; *141, 142*, 125 µm; *143–145*, 125 µm; *146–148*, 125 µm; *149, 150*, 125 µm. la, lateral apodeme; lva, latero-ventral apodeme.

represent more apomorphic members of the *Aphanomerus*-cluster: they are typified by having a subcompact to compact antennal clava and mouthparts that are opisthognathous. However, the ovipositor system of *Amitus*, the only genus examined in this group, is very different from any in the *Aphanomerus*-cluster. *Amitus* has a very broad robust ovipositor assembly, with huge proximal arms (Fig. 152) and, importantly, lacks latero-ventral apodemes on S6, a character almost exclusively associated with genera in the *Aphanomerus*-group. Until ovipositor characters can be examined for the other genera in this cluster, the uniqueness of the *Amitus* system is impossible to assess.

### ***Allotropa*-cluster**

### Genus ***Allotropa*** Foerster

(Figs 154, 155)

*Diagnosis*

Ovipositor assembly extended and retracted by muscles (i.e. *Ceratobaeus*-type system), rather delicate in form; ovipositor moderately elongate, (?) about 0·7× length of metasoma; proximal part of ventral membranous plate present; gonoplacs extremely elongate, about 0·75× length of ovipositor; 2nd gonapophyses assembly simple; proximal arms slender, 0·13× length of ovipositor; lateral apodemes joined by fine U-shaped rod-like T7, very short, 0·25× length of ovipositor, diverging at proximal ends, without small membranous plate attached postero-medially; T8 absent; S6 simple, independent latero-ventral apodemes or those attached to S6 absent.

*Comments on* Allotropa-*cluster*

This cluster contains only two genera, *Allotropa* and the endemic New Zealand genus *Errolium* Masner & Huggert Although there are few if any morphological characters that relate these genera, and they are only doubtfully placed together (Masner and Huggert 1989), they are the only platygastrids to have Coccoidea as a host group (Table 1). The ovipositor system of *Allotropa* has no special features; it is slender in form with rather fine lateral apodemes (Fig. 154, 155), and has a simple S6. Although we dissected a specimen of *Errolium*, few details were clear. The ovipositor assembly is about 0·7× the length of the metasoma and the lateral apodemes are somewhat longer than in *Allotropa*, being 0·37× the length of the ovipositor.

## Subfamily **Platygastrinae**

### ***Proplatygaster*-cluster**

### Genus ***Proplatygaster*** Kieffer

(Fig. 156)

*Diagnosis*

Ovipositor assembly extended and retracted by muscles (i.e. *Ceratobaeus*-type system); ovipositor short, about 0·6× length of metasomal; (?) proximal part of ventral membranous plate absent; gonoplacs elongate, 0·7× length of ovipositor; 2nd gonapophyses assembly simple; proximal arms broad and elongate, 0·28× length of ovipositor; lateral apodemes joined by U-shaped rod-like T7, short, about 0·4× length of ovipositor, diverging proximally; T8 absent; S6 simple, independent latero-ventral apodemes or those attached to S6 absent.

### Genus ***Almargella*** Masner & Huggert

(Figs 157, 158)

*Diagnosis*

Ovipositor assembly extended and retracted by muscles (i.e. *Ceratobaeus*-type system); ovipositor moderately elongate, (?) about 0·7× length of metasoma; proximal part of ventral

membranous plate present; gonoplacs moderately elongate, 0·63× length of ovipositor; 2nd gonapophyses assembly complex; 2nd gonocoxae very broad, almost twice as wide as gonoplacs, about 0·3× length of ovipositor; proximal arms moderately elongate and broad, but boundaries indistinct, about 0·15× length of ovipositor; lateral apodemes joined by U-shaped rod-like T7, very short, about 0·3× length of ovipositor, diverging proximally, with small membranous plate attached postero-medially (possibly remnant of T8 with cerci absent); S6 simple, independent latero-ventral apodemes or those attached to S6 absent.

### Genus ***Metaclisis*** Foerster

(Figs 159, 160)

*Diagnosis*

Ovipositor assembly extended and retracted by muscles (i.e. *Ceratobaeus*-type system); ovipositor moderately elongate, (?) about 0·7× length of metasoma; (?) proximal part of ventral membranous plate present; gonoplacs short, about 0·4× length of ovipositor or shorter; 2nd gonapophyses assembly complex; proximal arms moderately elongate, about 0·2× length of ovipositor; lateral apodemes joined by U-shaped rod-like T7, very short, about 0·2× length of ovipositor, without small membranous plates attached postero-medially; T8 absent; S6 subtriangular, with 2 short latero-ventral apodemes that extend proximally for same distance as lateral apodemes but do not connect with them, including S6 latero-ventral apodemes about 0·25× length of ovipositor.

*Comments on* Proplatygaster-*cluster*

This group of genera is characterised by spindle-like forms with large body size (Masner and Huggert 1989). Also, several genera (*Almargella*, *Magellanium* Masner & Huggert, *Proplatygaster* and *Zelostemma* Masner & Huggert) are restricted to southern hemisphere continents and, where records are available, the Cecidomyiidae is the only host group known (Table 1). However, generally the members of this cluster are morphologically heterogeneous and this is also reflected in the structure of the ovipositor system. *Metaclisis* has S6 with attached latero-ventral apodemes (Fig. 160), a character found elsewhere only in genera of the *Aphanomerus*-cluster. *Almargella* has a moderately robust ovipositor assembly, the 2nd gonocoxae unusually broad (Fig. 157), T7 (the transverse rod-like part of the lateral apodemes) with a posterior membranous plate that may be a remnant of T8 (Fig. 158), and S6 simple. *Proplatygaster*, postulated by Masner and Huggert (1989) to be the most plesiomorphic genus in this cluster, has a relatively short ovipositor assembly, but otherwise one that is unremarkable compared with many other platygastrids. Clearly, there is a need to examine the ovipositor systems of the other rarer genera and to assess their structure in the light of this group possibly being polyphyletic.

## ***Isostasius***-cluster

### Genus ***Isostasius*** Foerster

(Fig. 161)

*Diagnosis*

Mechanism for ovipositor extension uncertain, possibly extended and retracted by muscles (i.e. *Ceratobaeus*-type system); ovipositor assembly generally delicate; ovipositor extremely elongate, coiled vertically 2–3 times in clockwise direction (viewed from right side) so that it is several times as long as metasoma (gonoplacs not seen in dissection); (?) proximal part of ventral membranous plate absent; 2nd gonapophyses assembly simple; proximal arms broad, very short relative to ovipositor length but 0·33× length of metasoma; (?) 2nd gonocoxae apparently attached to proximal arms and disassociated from proximal gonoplacs; (?) lateral apodemes apparently absent; T8 absent; S6 simple, independent latero-ventral apodemes or those attached to S6 absent.

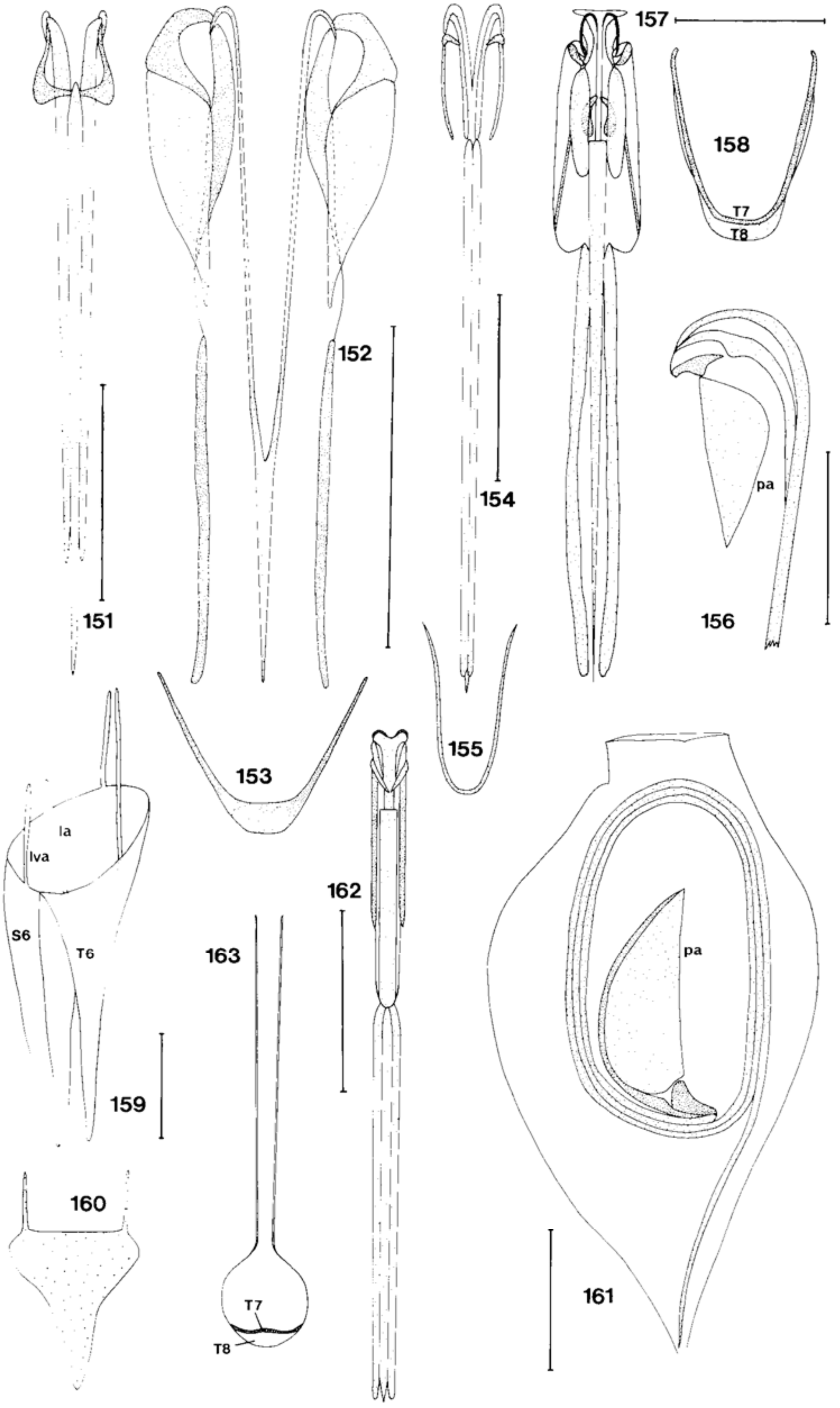
151
152
153
154
155
156
pa
157
158
T7
T8
159
la
lva
S6
T6
160
161
pa
162
163
T7
T8

*Comments on* Isostasius-*cluster*

This cluster contains only four genera of which *Isostasius* and *Aceroteta* Kozlov and Masner are cosmopolitan and *Annettella* Masner & Huggert and *Rao* Masner & Huggert are restricted to the Australasian region. Also, the latter three genera are known from single described species, but with undescribed species known in collections. The group is defined on the basis of characters associated with the propodeum, ocelli and structure of the metasoma, but no character is shared by all four genera (Masner and Huggert 1989). *Isostasius* and *Rao* are distinctive because of their highly modified metasoma (dorsally and ventrally expanded, T3–T5 fused and T6 internal). This external shape of the metasoma complements the internal structure of the ovipositor system for *Isostasius*. The ovipositor is coiled vertically so that it is up to several times the length of the metasoma, the proximal arms are greatly enlarged (Fig. 161), and T7 and S6 apparently lack apodemes. This may indicate that the ovipositor system is operated hydrostatically and not by antagonistic muscles. Given the similar external structure of the metasoma in *Rao*, we would predict that this genus also has an ovipositor similar in structure to that of *Isostasius*. However, *Aceroteta* and *Annettella* differ substantially in the shape of the metasoma and may very well have a different ovipositor system. A specimen of *Annettella* was dissected and, other than it having a moderately elongate ovipositor (0·7× length of metasoma) that is not coiled and elongate gonoplacs (0·7× length of ovipositor), we were unable to determine the overall structure of the system.

## ***Inostemma***-cluster

### Genus ***Inostemma*** Haliday

(Figs 164, 165)

*Diagnosis*

Ovipositor assembly extended and retracted by muscles (i.e. *Ceratobaeus*-type system), moderately robust in form; ovipositor elongate to extremely elongate, sometimes extending into horn of T1 and thus longer than metasoma, 0·85–1·7× length of metasoma; rami of 1st gonapophyses broad, truncate; proximal part of ventral membranous plate present; 2nd gonapophyses assembly complex; 2nd gonocoxae broadening distally, about 0·4× length of ovipositor; gonoplacs short, about half length of ovipositor, not quite reaching to tip of ovipositor; proximal arms slender, moderately elongate, about 0·2× length of ovipositor, attached distally to tubular membranous extension (tubular A9) that expands into broad distal part of ventral membranous plate; lateral apodemes very short, fine, extending from membranous plate (T7) which is lightly sclerotised along lateral margins (indistinct distally), apodemes about 0·4× length of ovipositor, including membranous plate; T8 absent; S6 simple, independent latero-ventral apodemes or those attached to S6 absent.

---

**Fig. 151.** Ovipositor assembly of *Isolia* sp., partly stylised (*Isolia*-cluster). **Figs 152, 153.** *Amitus* sp. (*Amitus*-cluster): *152*, ovipositor assembly; *153*, lateral apodemes. **Figs 154, 155.** *Allotropa* sp. (*Allotropa*-cluster): *154*, ovipositor assembly; *155*, lateral apodemes. **Fig. 156.** Lateral view of the proximal ovipositor assembly of *Proplatygaster* sp. (*Proplatygaster*-cluster). **Figs 157, 158.** *Almargella* sp. (*Proplatygaster*-cluster): *157*, ovipositor assembly; *158*, lateral apodemes. **Figs 159, 160.** *Metaclisis* sp. (*Proplatygaster*-cluster): *159*, lateral view of tergite 6, lateral apodemes and sternites 6+7+8 with latero-ventral apodemes; *160*, dorsal view of sternites 6+7+8 with latero-ventral apodemes. **Fig. 161.** Lateral view of the metasoma of *Isostasius* sp. (*Isostasius*-cluster) showing coiled ovipositor assembly. **Figs 162, 163.** *Acerotella aceris* Masner (*Inostemma*-cluster): *162*, ovipositor assembly; *163*, membranous tergites 7+8 and lateral apodemes. Scale lines: *151*, 125 µm; *152, 153*, 125 µm; *154, 155*, 125 µm; *156*, 125 µm; *157, 158*, 125 µm; *159, 160*, 125 µm; *161*, 125 µm; *162, 163*, 125 µm. la, lateral apodeme; lva, latero-ventral apodeme; S6, metasomal sternites 6+7+8; pa, proximal arm; T6, metasomal tergite 6; T7, metasomal tergite 7; T8, metasomal tergite 8.

Genus ***Acerotella*** Masner
(Figs 162, 163)

*Diagnosis*

Ovipositor assembly extended and retracted by muscles (i.e. *Ceratobaeus*-type system), somewhat delicate in form; ovipositor elongate, about 0·8× length of metasoma; proximal part of ventral membranous plate present; gonoplacs moderately elongate, 0·6× length of ovipositor; 2nd gonapophyses assembly simple; proximal arms slender, moderately elongate, about 0·2× length of ovipositor; T7 distinct, represented as a sclerotised ring fused to anterior margin of T8; lateral apodemes delicate, moderately elongate, including T7+8 0·65× length of ovipositor, with distal part of ventral membranous plate connecting lateral apodemes to T7, margins of plate slightly more sclerotised; T8 short, crescent-shaped and membranous, cerci absent; S6 simple, independent latero-ventral apodemes or those attached to S6 absent.

Genus ***Iphitrachelus*** Walker
(Figs 166, 167)

*Diagnosis*

Ovipositor assembly extended and retracted by muscles (i.e. *Ceratobaeus*-type system), moderately robust in form; ovipositor short, about 0·6× length of metasoma; (?) proximal part of ventral membranous plate present; gonoplacs elongate, apparently continuous and fused with 2nd gonocoxae; 2nd gonapophyses assembly enlarged and complex; 2nd gonocoxae simple; proximal arms elongate, 0·29× length of ovipositor; rami of 1st gonapophyses with very elongate distal rod-like extensions, 0·66× length of ovipositor; lateral apodemes very short, joined proximally by U-shaped rod-like connection, with T7+8 about 0·35× length of ovipositor, without distal part of ventral membranous plate attached; T7+8 present and fused, remnants of cerci indicated by lateral leaf-shaped areas; S6 apparently simple, independent latero-ventral apodemes or those attached to S6 absent.

*Comments on* Inostemma-*cluster*

This cluster of mostly well-speciated cosmopolitan genera, associated with cecidomyiid hosts (Table 1), is defined by the structure of metasomal segment 2. Of the four genera included, *Sacespalus* Kieffer is probably a synonym of *Inostemma* (Masner and Huggert 1989), the latter genus being well known for the development of an elongate horn on T1 in many species. Previously, species of *Inostemma* that lack a horn have been confused with *Acerotella*, but the distinction between and stability of these genera have been determined by Masner (1980*c*) and later by Masner and Huggert (1989).

The ovipositor system of the genera in this cluster provides no evidence of its possible monophyly. Indeed, the three genera examined have systems that are very different from each other and all other platygastrids. *Acerotella* is unique among platygastrids because it possesses a fully developed and sclerotised T8 (Fig. 163), whereas in all other genera this tergite is absent (except for *Iphitrachelus*) or exists only as a membranous plate attached to the posterior of a rod-like T7. The lateral apodemes of *Acerotella* are also unique in that they are extremely elongate compared with those in other platygastrid genera, as well as being fine and connected posteriorly by the distal part of the ventral membranous plate. As such, the ovipositor system of this genus bears a striking resemblance to that of *Ceratobaeus* (Baeini) (Austin 1983), as opposed to those of most other platygastrids in which the apodemes are more similar to the primitive scelionid *Sparasion* (Fig. 14).

The ovipositor system of *Iphitrachelus* is also very unusual. It is generally robust in form, the gonoplacs are apparently continuous with the 2nd gonocoxae, and the rami of the 1st gonapophyses have very elongate distal rod-like extensions (Fig. 166). However, the most aberrant features of this genus are that the lateral apodemes are joined proximally by a U-shaped rod-like connection, and T7+8 is present, fused and well-sclerotised, and has leaf-

shaped areas laterally where the cerci are normally situated in scelionids (Fig. 167). The above characters are unique to *Iphitrachelus* and therefore can be interpreted as apomorphic. However, the presence of a fused T7+8 with cerci-like structures is probably plesiomorphic for platygastrids and indicates a link in the transition of these characters from their presence in scelionids to their loss in the platygastrids.

Although the ovipositor system of *Inostemma* is the least unusual of the genera in this cluster, it still possesses a number of distinctive characters; in particular, the ovipositor is often extremely elongate and extends into a horn on T1, the 2nd gonocoxae are broadened distally, the ventral membranous plate is expanded and spade-shaped (Fig. 164), the lateral apodemes are delicate, and T7 is membranous (Fig. 165). However, given the size of this genus and recognition of many aberrant forms (Masner and Huggert 1989), the ovipositor system needs to be surveyed across a wider representation of species to determine the level of intrageneric variability for some of these characters.

## ***Allostemma*-cluster**

### Genus ***Allostemma*** Masner & Huggert

(Figs 168, 169)

*Diagnosis*

Ovipositor assembly extended and retracted by muscles (i.e. *Ceratobaeus*-type system), somewhat delicate in form; ovipositor moderately elongate, (?) about 0·7× length of metasoma; proximal part of ventral membranous plate present; gonoplacs elongate, 0·7× length of ovipositor; 2nd gonapophyses assembly complex; 2nd gonocoxae broadening posteriorly, at widest more than twice as wide as gonoplacs, about 0·3× length of ovipositor; proximal arms very short and slender, 0·08× length of ovipositor; lateral apodemes joined by moderately broad U-shaped rod-like T7, very short, about 0·15× length of ovipositor, diverging, broadened U-shaped part of T7 with small membranous plate attached postero-medially (possibly remnant of T8 with cerci absent); S6 simple, independent latero-ventral apodemes or those attached to S6 absent.

*Comments on* Allostemma-*cluster*

*Allostemma* is the only genus to be placed in its own cluster. It is postulated to be the sister-group to the *Inostemma*-cluster and the most plesiomorphic extant platygastrid genus by Masner and Huggert (1989), based on its 5- or 6-segmented antennal clava, primitive antennal sensillae formula, 1-2-2 tibial spur formula, and more complete wing venation. The ovipositor system of *Allostemma* has no remarkable features, with the exception that the 2nd gonocoxae are somewhat broadened (Fig. 168), similar to those of *Almargella*, and T7 has a membranous plate attached (Fig. 169), possibly homologous with a reduced T8. The latter of these two characters is undoubtedly plesiomorphic but, whether or not the ovipositor system supports a relationship with the *Inostemma*-group, or a more basal position, will require a detailed assessment of character polarity across the family (see below under Phylogenetic Implications).

## ***Platygaster*-cluster**

### Genus ***Platygaster*** Latreille

(Figs 180–183)

*Diagnosis*

Ovipositor assembly extended and retracted by muscles (i.e. *Ceratobaeus*-type system), somewhat delicate in form; ovipositor elongate, about 0·8× length of metasoma; (?) proximal part of ventral membranous plate present; gonoplacs elongate to extremely elongate, 0·7–0·9× length of ovipositor; 2nd gonapophyses assembly simple; proximal arms slender, very short to

moderately elongate, 0·08–0·15× length of ovipositor; lateral apodemes joined by U-shaped rod-like T7, very short, 0·15–0·28× length of ovipositor, sometimes with small membranous plate attached postero-medially (possibly remnant of T8, otherwise absent; cerci absent); latero-ventral apodemes independent of S6, forming a second U-shaped structure, attached to lateral apodemes at proximal ends, sometimes latero-ventral apodemes much shorter than lateral apodemes; S6 simple.

### Genus *Amblyaspis* Foerster

(Figs 170, 171)

*Diagnosis*

Ovipositor assembly extended and retracted by muscles (i.e. *Ceratobaeus*-type system), moderately robust in form; ovipositor very short, less than half length of metasoma; proximal part of ventral membranous plate absent; gonoplacs short, 0·46× length of ovipositor; 2nd gonapophyses assembly simple; 2nd gonocoxae not clearly visible; proximal arms elongate, 0·28× length of ovipositor, broad proximally, tapering distally, rami extending anteriorly well past proximal ends of gonocoxae; lateral apodemes joined by U-shaped rod-like T7, very short, about 0·25× length of ovipositor diverging, without membranous plate attached postero-medially; T8 absent; S6 simple, independent latero-ventral apodemes or those attached to S6 absent.

### Genus *Eritrissomerus* Ashmead

(Fig. 172)

*Diagnosis*

Ovipositor assembly extended and retracted by muscles (i.e. *Ceratobaeus*-type system), moderately robust in form; ovipositor moderately elongate, (?) about 0·7× length of metasoma; proximal part of ventral membranous plate absent; gonoplacs elongate, 0·7× length of ovipositor; 2nd gonapophyses assembly moderately simple; 2nd gonocoxae simple; proximal arms elongate, reaching past proximal ends of gonoplacs, 0·38× length of ovipositor, moderately broad proximally, tapering and elongate distally, rami extending anteriorly well past proximal ends of gonocoxae; lateral apodemes unknown; T8 absent; S6 apparently simple, independent latero-ventral apodemes or those attached to S6 absent.

---

**Figs 164, 165.** *Inostemma oculare* Austin (*Inostemma*-cluster): *164*, ovipositor assembly; *165*, sternites 6+7+8 with latero-ventral apodemes. **Figs 166, 167.** *Iphitrachelus lar* Walker (*Inostemma*-cluster): *166*, ovipositor assembly; *167*, lateral apodemes. **Figs 168, 169.** *Allostemma* sp. (*Alostemma*-cluster): *168*, ovipositor assembly; *169*, lateral apodemes. **Figs 170, 171.** *Amblyaspis* sp. (*Platygaster*-cluster): *170*, ovipositor assembly; *171*, lateral apodemes. **Fig. 172.** Ovipositor assembly of *Eritrissomerus* sp. (*Platygaster*-cluster). **Figs 173, 174.** *Euxestonotus* sp. (*Platygaster*-cluster): *173*, ovipositor assembly; *174*, lateral apodemes. **Figs 175, 176.** *Metanopedias* sp. (*Platygaster*-cluster): *175*, ovipositor assembly; *176*, lateral lateral apodemes and membranous T7. Scale lines: *164, 165*, 125 μm; *166, 167*, 60 μm; *168, 169*, 125 μm; *170, 171*, 60 μm; *172*, 125 μm; *173, 174*, 125 μm; *175, 176*, 125 μm. c, remnants of cerci; la, lateral apodeme; T7, metasomal tergite 7; T7+8, fused metasomal tergites 7 and 8; T8, metasomal tergite 8; vmp, ventral membranous plate.

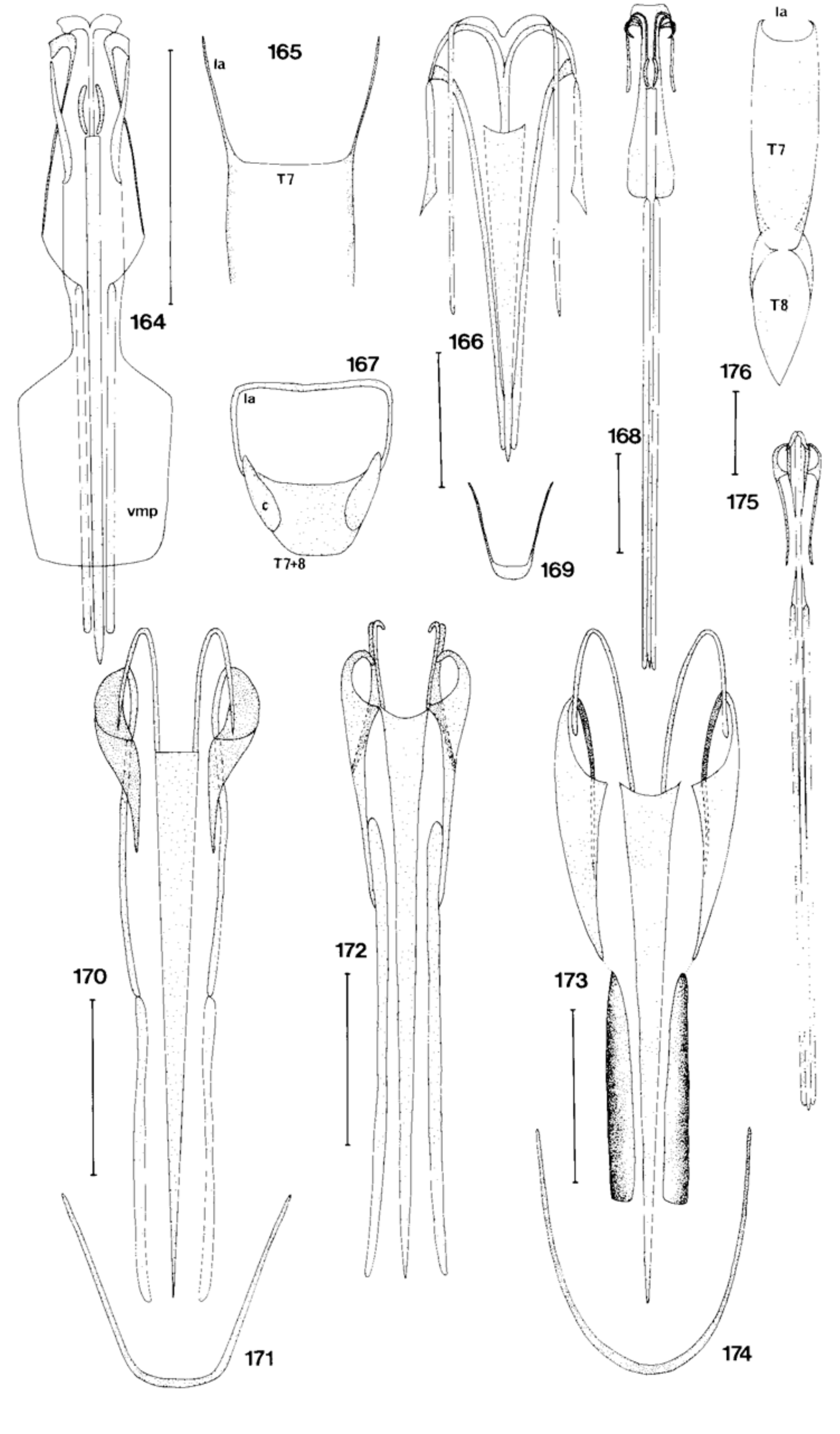
164
vmp
165
la
T7
166
167
la
c
T7+8
168
169
170
171
172
173
174
175
176
la
T7
T8

### Genus *Euxestonotus* Fouts
(Figs 173, 174)

*Diagnosis*

Ovipositor assembly extended and retracted by muscles (i.e. *Ceratobaeus*-type system), robust in form; ovipositor short, about 0·6× length of metasoma; (?) proximal part of ventral membranous plate present; gonoplacs short, broad, about 0·3× length of ovipositor, not reaching to tip of ovipositor; 2nd gonapophyses assembly enlarged and complex; 2nd gonocoxae simple; proximal arms elongate, longer than gonoplacs, about 0·35× length of ovipositor, very broad proximally, tapering distally, rami extending anteriorly well past proximal ends of gonocoxae; lateral apodemes joined by moderately broad U-shaped rod-like T7, very short, about 0·35× length of ovipositor, without membranous plate attached postero-medially; T8 absent; S6 apparently simple, independent latero-ventral apodemes or those attached to S6 absent.

### Genus *Isocybus* Foerster
(Figs 177–179)

*Diagnosis*

Ovipositor assembly extended and retracted by muscles (i.e. *Ceratobaeus*-type system), moderately robust in form; ovipositor short, about 0·6× length of metasoma; (?) proximal part of ventral membranous plate present; gonoplacs moderately elongate, broad, 0·58× length of ovipositor; 2nd gonapophyses assembly simple; 2nd gonocoxae articulated near point of attachment with gonoplacs; proximal arms thin, tapering, extremely elongate, longer than gonoplacs, 0·7× length of ovipositor; lateral apodemes joined by moderately broad U-shaped rod-like T7, very short, about 0·15× length of ovipositor, widely separated and diverging, without membranous plate attached postero-medially; T8 absent; S6 apparently simple, independent latero-ventral apodemes or those attached to S6 absent.

### Genus *Metanopedias* Brues
(Figs 175, 176)

*Diagnosis*

Ovipositor assembly extended and retracted by muscles (i.e. *Ceratobaeus*-type system), moderately delicate in form; ovipositor elongate and narrow, (?) about 0·8× length of metasoma; (?) proximal part of ventral membranous plate present; gonoplacs elongate, narrow, about 0·7× length of ovipositor; 2nd gonapophyses assembly simple; 2nd gonocoxae very narrow, narrower than gonoplacs; proximal arms thin, tapering and short, 0·12× length of ovipositor; T7 represented by elongate membranous plate (possibly fused with distal part of ventral membranous plate), with narrow partly sclerotised bridge that does not meet medially; lateral apodemes very short and curving from distal end of membranous plate of T7; T8 apparently present as pointed membranous extension posterior to sclerotised bridge; cerci absent; S6 apparently simple, independent latero-ventral apodemes or those attached to S6 absent.

### Genus *Trichacis* Foerster
(Figs 184, 185)

*Diagnosis*

Ovipositor assembly extended and retracted by muscles (i.e. *Ceratobaeus*-type system), very robust in form; ovipositor moderately elongate, (?) about 0·7× length of metasoma; (?) proximal

part of ventral membranous plate present; gonoplacs short, broad, 0·32× length of ovipositor; 2nd gonapophyses assembly simple; 2nd gonocoxae not clearly visible; proximal arms extremely elongate, longer than gonoplacs, about 0·53× length of ovipositor, very broad proximally, tapering distally; rami extending anteriorly well past proximal ends of gonocoxae; lateral apodemes joined by moderately broad U-shaped rod-like T7, very short, about 0·2× length of ovipositor, diverging, without membranous plate attached postero-medially; T8 absent; S6 simple, independent latero-ventral apodemes or those attached to S6 absent.

*Comments on* Platygaster-*cluster*

The genera of Platygastridae not treated by Masner and Huggert (1989) all belong to the subfamily Platygastrinae and, following the informal higher-level arrangement proposed by these authors, they can be best treated as two additional generic clusters: the *Platygaster*-cluster and *Synopeas*-cluster. Although the monophyly of these clusters is not established, they can be loosely diagnosed as follows: *Platygaster*-cluster – female antennal clava 5-segmented, palpal formula 2-1, medial keels of propodeum nearly always separated, scutellum mostly unarmed, tibial spur formula 1-2-2; and *Synopeas*-cluster – female antennal clava rarely 3- mostly 4-segmented, papal formula 1-1, medial keels of propodeum approximated or confluent, scutellum armed although sometimes reduced, tibial spur formula 1-1-1 (L. Masner, personal communication). As for other genera in the Platygastrinae, where their biology is known, only the Cecidomyiidae have been recorded as hosts (Table 1). However, compared with all other generic groups, the *Platygaster*- and *Synopeas*-clusters contain most of the described species for the family, with *Platygaster* and *Synopeas* Foerster having approximately 450 (46%) between them (Vlug 1995).

The ovipositor system of the *Platygaster*-cluster is reasonably uniform for most genera, although it can be divided into two distinct groups. The first is characterised by having a very robust ovipositor assembly, proximally exposed rami of the 1st gonapophyses, broad often elongate proximal arms, moderately robust lateral apodemes and short gonoplacs (Figs 170–174, 177, 184, 185). This group contains the genera *Amblyaspis*, *Eritrissomerus*, *Euxestonotus*, *Isocybus* and *Trichacis*. The second group contains *Platygaster, Metanopedias* and possibly *Trichacoides* Dodd (not examined here), and is characterised by having an elongate delicate ovipositor assembly, normally positioned rami, short fine proximal arms, elongate gonoplacs and fine lateral apodemes (Figs 175, 180–183). The only character in the latter group that differs from this pattern is the arrangement of the lateral apodemes in *Metanopedias*, which are very short and curving from the distal end of a membranous plate that forms T7 and possibly parts of the ventral membranous plate. Further, T7 has a narrow lightly sclerotised bridge that does not meet medially and, distal to this, a pointed membranous extension may represent the remnants of T8.

As for many other genera examined, several characters display substantial intrageneric variability. This is most notable for *Platygaster* and *Synopeas*. In the latter genus species can be divided into two groups: one in which the ovipositor is straight and shorter than the mesosoma (Fig. 187) and the other in which the ovipositor is partly coiled into a sternal horn (i.e. *Sactogaster*, Fig. 188). In both cases the mechanism for ovipositor extension and contraction is unknown, but the fact that lateral apodemes are apparently absent indicates that changes in tergal pressure may be involved.

Species of *Platygaster* appear to be even more variable in that they apparently differ in the presence and absence of latero-ventral apodemes, indicating that a fundamental phylogenetic division may exist in this highly speciose genus. Several species also differ in having an extremely elongate membranous tube protruding from the posterior metasoma (Huggert 1980). We have not dissected any such species, but from Huggert's figures it appears as though this tube is homologous to a greatly elongate tubular A9, and what appear to be cerci at the end of the tube (absent in virtually all other platygastrids) are sensory hairs at the ends of the mostly hidden gonoplacs. In one species, *P.* (*Cylindrogaster*) *tubulosa* Brues, the intersegmental membrane between the posterior three metasomal segments is lengthened so that they can be telescoped outwards and thus increase the effective length of the ovipositor (Huggert 1980). This arrangement is not known in any other members of the Platygastroidea and is presumed to

be associated only with this highly derived group within the genus. Species of *Platygaster* may further vary in the lengths and relative robustness of the lateral apodemes (Figs 181, 183), in a way analogous to those in species of the scelionid genus *Ceratobaeus* (Austin 1983).

### *Synopeas*-cluster

### Genus *Synopeas* Foerster

(Figs 187, 188)

*Diagnosis*

Ovipositor assembly extended and retracted by muscles (i.e. *Ceratobaeus*-type system); ovipositor moderately to extremely elongate, 0·75–1·5× length of metasoma, sometimes partly coiled vertically at rest inside ventral horn of S2; proximal part of ventral membranous plate present; gonoplacs moderately elongate to elongate, in species with short straight ovipositor gonoplacs 0·6–0·7× length of ovipositor; 2nd gonapophyses assembly simple; when ovipositor extremely elongate and coiled of proximal head of ovipositor apparently greatly reduced in size; when ovipositor straight (not coiled) proximal head and other elements normally developed; proximal arms extremely elongate, folded and tapering distally, about 0·6× length of ovipositor; rami extending anteriorly well past proximal ends of gonocoxae; distal part of ventral membranous plate enlarged and spade-shaped; lateral apodemes apparently absent; T8 absent; S6 simple, independent latero-ventral apodemes or those attached to S6 absent.

### Genus *Leptacis* Foerster

*Diagnosis*

Ovipositor assembly extended and retracted by muscles (i.e. *Ceratobaeus*-type system); ovipositor very short and robust, 0·38× length of metasoma; (?) proximal part of ventral membranous plate absent; gonoplacs moderately elongate, 0·57× length of ovipositor; 2nd gonapophyses assembly simple; proximal arms extremely elongate, very broad, as long as gonoplacs, 0·57× length of ovipositor; T7 membranous, lateral apodemes absent; T8 absent; S6 simple, independent latero-ventral apodemes or those attached to S6 absent.

### Genus *Piestopleura* Foerster

(Fig. 186)

*Diagnosis*

Ovipositor assembly extended and retracted by muscles (i.e. *Ceratobaeus*-type system); ovipositor moderately elongate, (?) about 0·7× length of metasoma; proximal part of ventral membranous plate absent; gonoplacs moderately elongate, 0·55× length of ovipositor; 2nd

**Figs 177–179.** *Isocybus canadensis* Provancher (*Platygaster*-cluster): *177*, lateral view of ovipositor assembly; *178*, lateral apodemes; *179*, sternites 6+7+8. **Figs 180–182.** *Platygaster* sp. 1 (*Platygaster*-cluster): *180*, ovipositor assembly; *181*, lateral apodemes; *182*, latero-ventral apodemes. **Fig. 183.** Lateral apodemes of *Platygaster* sp. 2 (*Platygaster*-cluster). **Figs 184, 185.** *Trichacis* sp. (*Platygaster*-cluster): *184*, ovipositor assembly; *185*, lateral apodemes. **Fig. 186.** Ovipositor assembly of *Piestopleura* sp. (*Synopeas*-cluster). **Fig. 187.** Ovipositor assembly of *Synopeas* sp. 1 (*Synopeas*-cluster). **Fig. 188.** Lateral view of the metasoma of *Synopeas* (*Sactogaster*) sp. 2 (*Synopeas*-cluster). Scale lines: *177–179*, 125 µm; *180–182*, 125 µm; *183*, 125 µm; *184*, *185*, 125 µm; *186*, 125 µm; *187*, 125 µm; *188*, 125 µm. g, gonoplacs; pa, proximal arms; vmp, ventral membranous plate.

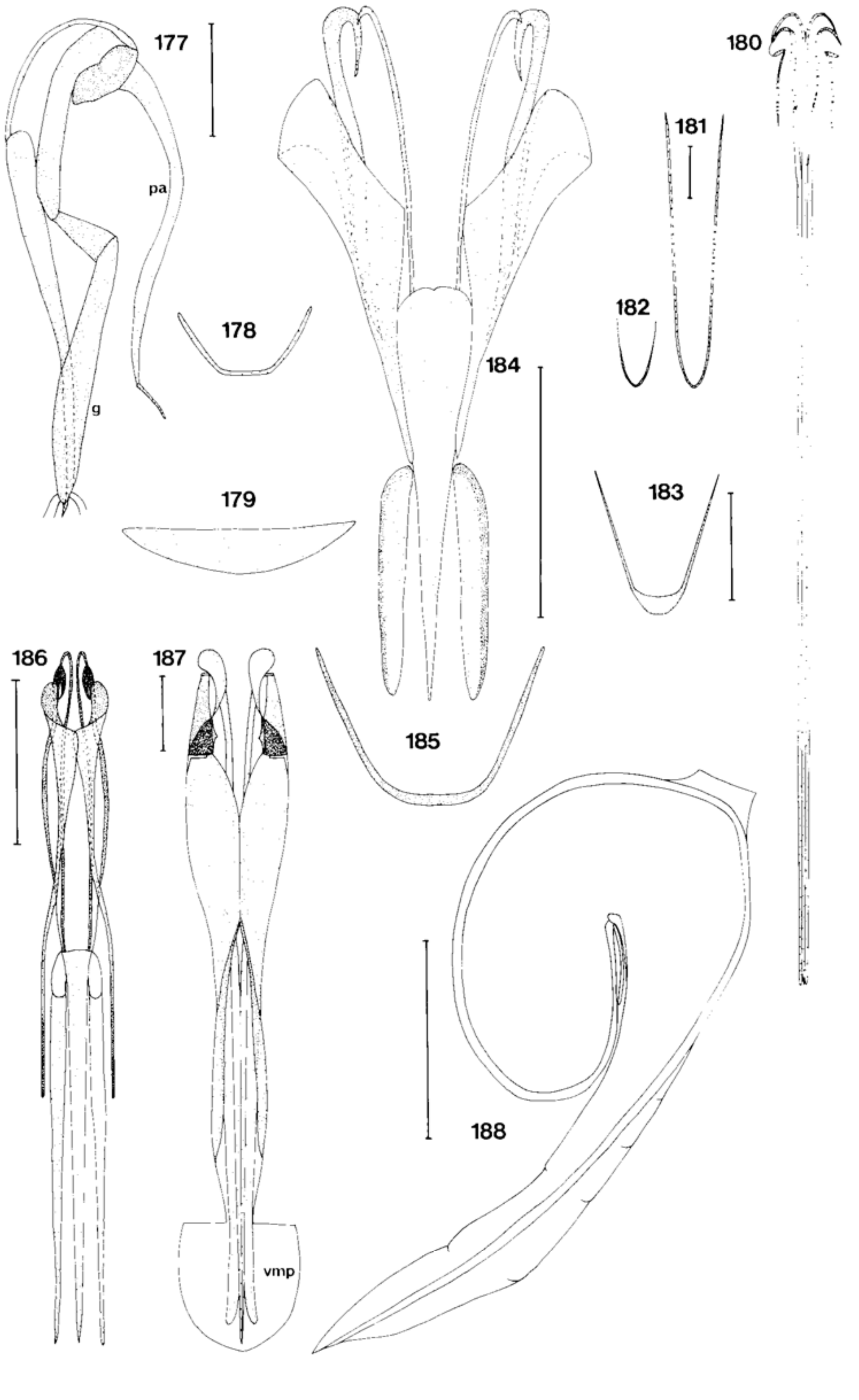
177
pa
g
178
179
180
181
182
183
184
185
186
187
vmp
188

gonapophyses assembly simple; proximal arms extremely elongate, strongly curved, broad proximally, very elongate and thin in distal three-quarters, longer than gonoplacs, 0·59× length of ovipositor; T7 membranous, lateral apodemes absent; T8 absent; S6 simple, independent latero-ventral apodemes or those attached to S6 absent.

*Comments on* Synopeas-*cluster*

More general comments about the *Synopeas*-cluster have been made under the *Platygaster*-cluster, above. The three genera examined here apparently share a number of potentially derived characters; in particular, the proximal arms are extremely elongate (Figs 186, 187), and the lateral apodemes on T7 are apparently absent, although the latter character needs to be verified by further dissections. However, *Synopeas* and *Leptacis* differ from *Piestopleura* in that the gonoplacs are more elongate, while *Synopeas* is unique in that the proximal arms are folded and tapering distally, and the distal part of the ventral membranous plate is enlarged and spade-shaped, at least in some species (Fig. 187). Also, some *Synopeas* have the ovipositor assembly greatly elongate and partly coiled vertically at rest inside a ventral horn of S2 (Fig. 188). In such species, there is apparently an accompanying reduction in the size of other elements associated with the system, particularly the proximal head (comprising the rami of the 1st gonapophyses, proximal arms, and proximal part of the 2nd gonocoxae), and this may indicate an ovipositor system that is extended by changes in hydrostatic pressure rather than muscle contraction, or possibly a combination of both.

The ovipositor system of *Leptacis* differs from other genera in this cluster in that it is very short (less that 0·4× length of metasoma) and extremely robust. In general form it is noticeably similar to *Isocybus* (Fig. 177), including having broad very elongate proximal arms and robust gonoplacs. No other genera of platygastrids share these characters and it is therefore possible that these genera are more closely related than their current placement suggests.

## Phylogenetic Implications

As outlined by Field and Austin (1994), the ovipositor system of scelionid and platygastrid wasps comprises a suite of characters that, in combination with data on external morphology and host relationships, is amenable for analysis of generic and higher-level relationships. However, the fact that both the *Scelio*- and *Ceratobaeus*-type ovipositor systems are unique compared with the rest of the parasitic Hymenoptera makes it difficult to polarise ovipositor characters by outgroup comparison. Field and Austin (1994) propose that the most parsimonious explanation for the evolution of the *Scelio*-type system is that it is the more derived of the two and has evolved from the *Ceratobaeus*-type system, given that the ovipositor in the former group is extended and retracted by hydrostatic pressure. Members of virtually all other wasps families employ muscles to orientate the ovipositor and, in addition, for platygastroids with the *Ceratobaeus*-type, the same muscles are also involved in its extension and retraction from the metasoma (Austin 1983; Field and Austin 1994). Further, our survey of the ovipositor system across the superfamily indicates that a monophyletic group containing the Scelionini *s. str.*, Aradophagini, Neoscelionini, Platyscelionini, Doddiellini, most of the Calliscelionini and Psilanteridini, and several apparently misplaced genera mostly from the Sparasionini and Baryconini (i.e. *Archaeoteleia*, *Bracalba, Chromoteleia, Oxyscelio* and *Sceliacanthella*), are all members of a large monophyletic group, comprising some 55 genera, that can be defined on the basis of the *Scelio*-type system (Fig. 189).

However, this *a priori* hypothesis requires the support of a cladistic analysis that allows characters associated with the ovipositor system to associate independently with each other and with other external characters. Here we score 14 characters for the ovipositor system and polarise them where possible by outgroup comparison with a range of chalcidoid families (i.e. Chalcididae, Copland and King 1971*a*; Eurytomidae, Copland and King 1972*b*; Pteromalidae, Copland and King 1971*b*, 1972*c*; King 1962; Torymidae, Copland and King 1972*a*) and more distantly with the Diapriidae (Naumann 1982), after the relationships suggested by the molecular study of Dowton *et al.* (in press). Several potentially useful characters are not included because we are uncertain as to the homology of some states, or because they are difficult to see in numerous genera and thus may be scored incorrectly, or they appear to be too variable at the generic level (e.g. proximal and distal parts of the ventral membranous plate, 2nd gonapophyses assembly and shape of the gonoplacs).

*Character Discussion*

*Character 1 (size of abdominal tergite 9).* This character has been divided into four discrete states for which outgroup comparison unequivocally shows state 0 to be plesiomorphic. These states can be run unordered or as a transitional series (0-1-2-3). AT9 large (Figs 25, 39, 60) (0); AT9 small (Fig. 13) (1); AT9 tiny, flap-like (Fig. 153) (2); AT9 absent (Fig. 148) (3).

*Character 2 (sclerotisation of abdominal tergite 9).* This is a binary character for which outgroup comparison shows state 0 to be plesiomorphic. AT9 well sclerotised (Fig. 67) (0); AT9 partly sclerotised (flexible or membranous, Fig. 19) (1); AT9 absent (?).

*Character 3 (sensory structures on abdominal tergite 9).* This is a binary character for which outgroup comparison shows state 0 to be plesiomorphic. Position of sensory structures defined (Figs 17, 19, 25, 49) (0); sensory structures absent (Fig. 163) (1); AT9 absent (?).

*Character 4 (definition of abdominal tergites 8 and 9).* This is a binary character for which outgroup comparison shows state 0 to be plesiomorphic. AT8 distinct from AT9 (separated by a space or suture, Figs 46, 60, 120) (0); AT8 completely fused with AT9 (Figs 26, 39, 53) (1); AT9 absent (?).

*Character 5 (lateral apodemes associated with abdominal tergites 8+9).* This is a multistate character for which outgroup comparison shows state 0 to be plesiomorphic. These states can be run as unordered or as a transitional series (0-1-3), with state 2 treated as an independent apomorphic state. Lateral apodemes absent (Fig. 188) (0); lateral apodemes present, free in body cavity, with associated muscles (Fig. 141) (1); lateral apodemes present, connected at proximal ends, free in body cavity (Fig. 167) (2); lateral apodemes incorporated into wall of telescopic tube, without associated muscles (Figs 25, 26) (3).

*Character 6 (length of lateral apodemes).* This is a multistate character for which outgroup comparison shows state 0 to be plesiomorphic. In some cases where the lateral and latero-ventral apodemes are fused they have been treated as one and measured as for lateral apodemes only. These states can be run as unordered or as a transitional series (0-1-2-3). When the lateral and ventro-lateral apodemes are fused sub-basally or sub-apically (see Character 8, state 3), their length is treated as if it were that of the lateral apodemes alone. Lateral apodemes very short, ≤ 0·35× length of ovipositor (0); lateral apodemes short, > 0·35 and < 0·6× length of ovipositor (1); lateral apodemes moderately elongate, ≥ 0·6 and < 0·8× length of ovipositor (2); lateral apodemes elongate, ≥ 0·8× length of ovipositor (3); lateral apodemes absent (?).

*Character 7 (definition of abdominal sternites 7 and 8+9).* This is a binary character for which outgroup comparison shows state 0 to be plesiomorphic. AS7 distinct from AS8+9 (separated by a suture, Figs 28, 118) (0); AS7 completely fused with AS8+9 (Figs 45, 51) (1).

*Character 8 (apodemes associated with abdominal sternites 7+8+9).* This is a complex multistate character for which outgroup comparison shows state 0 to be plesiomorphic (see below). Some states are also possibly not homologous (e.g. state 2) and so should be treated as unordered. Apodemes absent (Fig. 79) (0); medial apodeme present (Fig. 28) (1); latero-ventral apodemes present as extensions from AS7+8+9 (Figs 145, 159) (2); latero-ventral apodemes present, loosely connected or detached from AS7+8+9, but attached to lateral apodemes sub-basally or subapically (Figs 14, 91) (3); latero-ventral apodemes present, loosely connected or detached from AS7+8+9 to form U-shaped structure, attached to lateral apodemes apically (Fig. 140) (4); latero-ventral apodemes present, loosely connected or detached from AS7+8+9 to form U-shaped structure, not attached to lateral apodemes proximally (Figs 150, 155) (5).

*Character 9 (intersegmental membrane between abdominal segment 7 and 8+9).* This is a multistate character for which outgroup comparison shows state 0 to be plesiomorphic. These states can be run as a transitional series (0-1-3), with state 2 treated as an independent apomorphic state. Intersegmental membrane virtually absent so that A8+9 is immovably joined to A7 (Figs 67, 122) (0); intersegmental membrane very short so that A8+9 can move short distance away from A7 (Figs 19, 119) (1); only tergal part of intersegmental membrane slightly lengthened so that A8+9 extended on short 'flap' (Fig. 70) (2); intersegmental membrane very elongate, telescopic, so that A8 + 9 can move substantial distance away from A7 (Figs 6, 34, 84) (3).

*Character 10 (ovipositor assembly).* This is a multistate character that, because of outgroup variability, should be treated as unpolarised. Ovipositor assembly fine and delicate (Figs 40) 47) (0); ovipositor assembly moderately robust (Figs 27, 80) (1); ovipositor assembly robust to very robust (Figs 90, 184) (2).

*Character 11 (ovipositor length).* This is a multistate character that, because of outgroup variability, should be treated as unpolarised. Ovipositor very short, < 0·6× length of metasoma (0); ovipositor short, ≥ 0·6 and < 0·7× length of metasoma (1); ovipositor moderately elongate, ≥ 0·7 and < 0·8× length of metasoma (2); ovipositor elongate, ≥ 0·8 and < 1·0× length of metasoma (3); ovipositor extremely elongate, ≥ 1·0× length of metasoma and straight (4); ovipositor extremely elongate, ≥ 1·0× length of metasoma and coiled (Figs 161, 188) (5).

*Character 12 (length of proximal arms).* This is a multistate character that, because of outgroup variability, should be treated as unpolarised. Proximal arms absent (0); proximal arms very short, < 0·1× length of ovipositor (1); proximal arms short, ≥ 0·1 and < 0·15× length of ovipositor (2); proximal arms moderately elongate, ≥ 0·15 and < 0·25× length of ovipositor (3); proximal arms elongate, ≥ 0·25 and < 0·5× length of ovipositor (4); proximal arms extremely elongate, ≥ 0·5× length of ovipositor (5).

*Character 13 (gonoplac length).* This is a multistate character that, because of outgroup variability, should be treated as unpolarised. Gonoplacs very short, < 0·3× length of ovipositor (0); gonoplacs short, ≥ 0·3 and < 0·55× length of ovipositor (1); gonoplacs moderately elongate, ≥ 0·55 and < 0·65× length of ovipositor (2); gonoplacs elongate, ≥ 0·65 and < 0·8× length of ovipositor (3); gonoplacs extremely elongate, ≥ 0·8× length of ovipositor (4).

*Character 14 (host group).* This is a multistate character that, because of outgroup variability, should be treated as unpolarised (see Table 1 for sources of information). Orthoptera (0); Mantodea (1); Embioptera (2); Heteroptera (3); Homoptera (4); Neuroptera (5); Coleoptera (6); Lepidoptera (7); Diptera (Cecidomyiidae) (8); Hymenoptera (9); Araneae (10).

*Preliminary Analysis*

Analysis of the data set (Table 2) resulted in a maximum number of 6300 trees (due to the available RAM) with a minimum length of 203 steps (strict consensus tree, consistency index = 0·254; retention index = 0·536; rescaled consistency index = 0·136). The effectiveness of the analysis, however, was diminished as it was aborted after 5 days with the TBR option of PAUP having searched only 700 of the 6300 trees. Although these may not represent the shortest possible trees for the given data set, they do suggest the monophyly of a number of major groups that have been discussed *a priori* (see above) or proposed by previous workers (e.g. Kozlov 1970; Masner 1976, 1980*b*; Galloway and Austin 1984; Masner and Huggert 1989). As outlined in Fig. 189, five groups are recognised and these correspond to the Scelionidae (minus the Sparasionini), the 'Scelionini *sensu lato*' (see below), Sparasionini *sensu stricto* (i.e. *Sparasion*+*Sceliomorpha*), Platygastridae, and Sparasionini *s. str.*+Platygastridae. Within these groups there is little or no resolution; that is, the monophyly of the tribes or generic clusters within the Scelionidae and Platygastridae, respectively, are not supported by ovipositor characters alone (see Discussion). The most robust of these groups is the 'Scelionini *s. l.*' for which the unequivocal synapomorphy is the *Scelio*-type ovipositor system (state 3 of Characters 5 and 9). The Platygastridae are supported by the loss or reduction of abdominal T9 (Characters 1–4). The Sparasionini *s. str.* are held together by T9 being small and only partly sclerotised (Characters 1 and 2), and they are placed as the sister-group to the Platygastridae based on their very short apodemes (Character 6). The Scelionidae are supported by elongate lateral apodemes (states 2 and 3 of Character 6) and most genera, with the exception of the 'Scelionini *s. l.*', have a medial apodeme on S6 (state 1 of character 8). On the basis of these characters the only genus that is excluded from the Scelionidae, apart from the Sparasionini, is *Neuroscelio*. However, other (external) characters strongly suggest that this genus is a member of the Gryonini (Galloway *et al.* 1992).

Of all the ovipositor characters examined here, the one that has been most difficult to interpret phylogenetically has been the latero-ventral apodemes (Character 8) and their association with the lateral apodemes. It seems likely that the arrangement of the apodemes in *Sparasion* and *Sceliomorpha* (Fig. 14), in that they are very short and the latero-ventral apodemes (of sternal origin) attach sub-basally to the lateral apodemes (of tergal origin), can be considered as the ground plan for the Platygastroidea. From this plan the arrangement found in most other members of superfamily can be derived. For instance, *Nixonia* is very similar to *Sparasion* and *Sceliomorpha* but the lateral apodemes are greatly elongate (Fig. 13). In most

**Table 2. Matrix of characters associated with the ovipositor and hosts for scelionid and platygastrid genera**

Details of characters and states are given in the text under Character Discussion. Missing data are coded as (–), and host groups are coded from Table 1, but with questionable host records treated as missing data. Genera are arranged in alphabetical order with tribes or other major groupings. *Trimorus*, *Telenomus*, *Platygaster* and *Synopeas* are listed twice to account for apparent variability in some characters (*Trimorus* 1 and 2 are *T. caraborum* and *T. canadensis*, respectively; *Telenomus*, *Platygaster* and *Synopeas* 1 and 2 represent unknown species, see Appendix)

| | 1 | 2 | 3 | 4 | 5 | 6 | 7 | 8 | 9 | 10 | 11 | 12 | 13 | 14 |
|---|---|---|---|---|---|---|---|---|---|---|---|---|---|---|
| **Scelioninae** | | | | | | | | | | | | | | |
| *Nixonia* | 1 | 1 | 0 | 1 | 1 | 2 | - | 3 | 0 | 1 | 2 | 2 | 3 | – |
| *Archaeoteleia* | 2 | 1 | 0 | 1 | 3 | – | – | 0 | 3 | 0 | 3,4 | 3 | 3 | 0 |
| *Sceliomorpha* | 0 | 1 | 0 | 0 | 1 | 0 | – | 3 | 0 | 1 | 3 | 2 | 3 | – |
| *Sparasion* | 0 | 1 | 0 | 0,1 | 1 | 0 | – | 3 | 0 | 1 | 3 | 2 | 3 | 0 |
| *Acanthoscelio* | 0 | 1 | 0 | 1 | 3 | 2 | 1 | 0 | 3 | 1 | 3 | 3,4 | 2 | – |
| *Dicroscelio* | 0 | – | 0 | 1 | 3 | 2 | 1 | 0 | 3 | 1 | 3 | 2 | 2 | – |
| *Heptascelio* | 0 | 1 | 0 | 1 | 3 | 2 | 1 | 0 | 3 | 1 | 3 | 2 | 2 | – |
| *Oreiscelio* | 0 | 1 | 0 | 1 | 3 | 2 | 1 | 0 | 3 | 1 | 3 | 3,4 | 2 | – |
| *Scelio* | 0 | 1 | 0 | 1 | 3 | 2 | 1 | 0 | 3 | 1 | 3 | 2 | 2 | 0 |
| *Synoditella* | 0 | 0 | 0 | 1 | 3 | 2 | 1 | 0 | 3 | 1 | 3 | 2 | 2 | 0 |
| *Baryconus* | 0 | 0 | 0 | 1 | 1 | 3 | 0 | 1 | 0 | 1 | 3 | 3 | 3 | 0 |
| *Bracalba* | 1 | 0 | 0 | 1 | 3 | 3 | – | 0 | 3 | 0 | 3 | 1 | 3 | – |
| *Chromoteleia* | 0 | 0 | 0 | 0 | 3 | 2 | – | 0 | 3 | 1 | 3 | 1 | 3 | – |
| *Oxyscelio* | 0 | 0 | 0 | – | 3 | – | 1 | 0 | 3 | 1 | 3 | 3 | 2,3 | – |
| *Anteromorpha* | 0 | 0 | 0 | 0 | 3 | 3 | 1 | 0 | 3 | 0 | 3,4 | – | 3 | 0 |
| *Calliscelio* | 0 | 0 | 0 | 0 | 3 | 2 | 1 | 0 | 3 | 0 | 4 | – | 2,3 | 0 |
| *Habroteleia* | 1 | 0 | 0 | 1 | 1 | 2 | 1 | 0 | 0 | 1 | 1 | 2 | 3 | – |
| *Holoteleia* | 0 | 0 | 0 | 0 | 3 | – | – | 0 | 3 | 0 | 3 | – | – | – |
| *Lispoteleia* | 0 | 0 | 0 | 0 | 3 | – | – | 0 | 3 | 0 | 3 | – | – | – |
| *Macroteleia* | 0 | 0 | 0 | 0 | 3 | 3 | 1 | 0 | 3 | 1 | 3,4 | 1 | 3 | 0 |
| *Oethecoctonus* | 0 | 0 | 0 | 0 | 3 | – | – | 0 | 3 | 0 | 3 | – | – | 0 |
| *Palpoteleia* | 0 | 0 | 0 | 0 | 1 | 3 | 0 | 1 | 0 | 1 | 2 | 3 | 2 | – |
| *Paridris* | 1 | 1 | 0 | 0 | 3 | 3 | 1 | 0 | 3 | 0 | 3 | 1 | 4 | – |
| *Phoenoteleia* | 0 | 0 | 0 | 0 | 3 | 3 | 1 | 0 | 3 | 1 | 4 | 3 | 3 | – |
| *Probaryconus* | 1 | 1 | 0 | 0 | 3 | 3 | 1 | 0 | 3 | 0 | 3,4 | – | – | 0 |
| *Triteleia* | 0 | 0 | 0 | 0 | 3 | 3 | 1 | 0 | 3 | 0 | 3 | 3 | 2 | – |
| *Anteris* | 0 | 0 | 0 | 0 | 1 | 2 | 1 | 0 | 0 | 2 | 2 | 3 | 3 | – |
| *Calotelea* | 0 | 0 | 0 | 0 | 3 | – | 1 | 0 | 3 | – | 4 | – | – | – |
| *Duta* | 0 | 0 | 0 | 0 | 3 | 3 | 1 | 0 | 3 | 1 | 3 | – | 3 | 0 |
| *Fusicornia* | 0 | 0 | 0 | 1 | 1 | 2 | 1 | 0 | 0 | 1 | 3 | 3 | 2 | – |
| *Harringtonia* | 0 | 0 | 0 | 0 | 3 | 3 | 1 | 0 | 3 | 1 | 3 | – | 3 | – |
| *Leptoteleia* | 0 | 0 | 0 | 0 | 1 | 3 | 0 | 1 | 0 | 1 | 3 | 4 | 1 | – |
| *Lidgbirdius* | 0 | 0 | 0 | – | 3 | – | 1 | – | 3 | – | – | – | – | – |
| *Mallateleia* | 0 | 0 | 0 | – | 3 | – | 1 | – | 3 | – | – | – | – | – |
| *Merriwa* | 0 | 0 | 0 | 0 | 3 | 3 | 1 | 0 | 3 | 0 | 4 | 1 | 3 | – |
| *Nyleta* | 0 | 0 | 0 | – | 3 | – | 1 | – | 3 | – | 3 | – | – | – |
| *Opisthacantha* | 0 | 0 | 0 | 0 | 1 | 2 | 1 | 1 | 0 | 1 | 3 | 2 | 3 | – |
| *Psilanteris* | 0 | 0 | 0 | 1 | 3 | 2 | 1 | 0 | 3 | 1 | 3 | 2 | 1 | – |
| *Shreemana* | 0 | 0 | 0 | – | 3 | – | 1 | – | 3 | – | – | – | – | – |
| *Spiniteleia* | 0 | 0 | 0 | 0 | 3 | 2 | 1 | 0 | 3 | 1 | 3 | 1 | 3 | – |
| *Styloteleia* | 0 | 0 | 0 | 0 | 1 | 2 | 1 | 1 | 0 | 1 | 4 | 3 | 3 | – |
| *Thoronella* | 0 | 0 | 0 | – | 3 | – | 1 | – | 3 | – | 4 | – | – | – |
| *Aradophagus* | 0 | 0 | 0 | 1 | 3 | 1 | 1 | 1 | 3 | 1 | 3 | 2 | 3 | – |
| *Neoscelio* | 0 | 0 | 0 | 1 | 3 | 2 | 1 | 0 | 3 | 1 | 2 | 1 | 3 | – |
| *Cremastobaeus* | 0 | 0 | 0 | 1 | 1 | 1 | 0 | 1 | 0 | 1 | 3 | 2 | 1 | – |

**Table 2.** continued

| | 1 | 2 | 3 | 4 | 5 | 6 | 7 | 8 | 9 | 10 | 11 | 12 | 13 | 14 |
|---|---|---|---|---|---|---|---|---|---|---|---|---|---|---|
| *Mantibaria* | 0 | 0 | 0 | 1 | 1 | 2 | 1 | 5 | 0 | 2 | 2 | 3 | – | 1 |
| *Platyscelio* | 0 | 1 | 0 | 1 | 3 | 2 | 1 | 0 | 3 | 1 | 2 | 3 | 3 | 0 |
| *Doddiella* | 0 | 0 | 0 | 1 | 3 | 1 | 1 | 0 | 3 | 1 | 2 | 2 | 3 | – |
| *Breviscelio* | 0 | 0 | 0 | – | 1 | 2 | – | 1 | 0 | 2 | – | – | – | – |
| *Dyscritobaeus* | 0 | 0 | 0 | 0 | 1 | 2 | 0 | 1 | 0 | 2 | 3 | 3,4 | 2 | – |
| *Encrytoscelio* | 0 | 0 | 0 | 0 | 1 | 2 | 1 | 1 | 0 | 2 | 3 | 4 | 2 | 3 |
| *Gryon* | 0 | 0 | 0 | 1 | 1 | 2 | 0 | 1 | 0 | 2 | 2,3 | 4 | 1 | 3 |
| *Neuroscelio* | 0 | 0 | 0 | 1 | 1 | 0 | 0 | 0 | 0 | 2 | 0 | 4 | 1 | – |
| *Echthrodesis* | 0 | 0 | 0 | 1 | 1 | – | 1 | 0 | 0 | 1 | 1 | – | – | 10 |
| *Embidobia* | 0 | 0 | 0 | 1 | 1 | 2 | 1 | 1 | 0 | 1 | 2 | 2 | 1 | 2 |
| *Embioctonus* | 0 | 0 | 0 | 1 | 1 | – | 1 | 1 | 0 | 1 | – | – | – | 2 |
| *Endecascelio* | 0 | 0 | 0 | 1 | 1 | – | 1 | 1 | 0 | 1 | 4 | – | – | – |
| *Palaeogryon* | 0 | 0 | 0 | 1 | 1 | – | 1 | 1 | 0 | 2 | – | – | – | 2 |
| *Microthoron* | 0 | 0 | 0 | 1 | 1 | – | 1 | 1 | 0 | 1 | – | – | – | – |
| *Pseudanteris* | 0 | 0 | 0 | 1 | 1 | – | 1 | 1 | 0 | 1 | – | – | – | – |
| *Thoron* | 0 | 0 | 0 | 1 | 1 | 1 | 1 | 1 | 0 | 1 | 3 | 1 | 3 | 3 |
| *Tiphodytes* | 0 | 0 | 0 | 1 | 1 | 3 | 1 | 1 | 0 | 1 | 2 | 2 | 1 | 3 |
| *Baeus* | 0 | 0 | 0 | 0 | 1 | 2 | 1 | 1 | 0 | 0 | 3 | 2 | 1 | 10 |
| *Ceratobaeus* | 0 | 0 | 0 | 1 | 1 | 2 | 1 | 1 | 0 | 0 | 4 | 1 | 1,2 | 10 |
| *Cyphacolus* | 0 | 0 | 0 | 1 | 1 | 3 | 1 | 1 | 0 | 0 | 5 | 1 | 1 | 10 |
| *Hickmanella* | 0 | 0 | 0 | 1 | 1 | 2 | 1 | 1 | 0 | 1 | 0,1,2 | 3 | 1 | 10 |
| *Idris* | 0 | 0 | 0 | 1 | 1 | 2 | 1 | 1 | 0 | 1 | 0,1,2 | 3 | 1 | 10 |
| *Mirobaeoides* | 0 | 0 | 0 | 0 | 1 | 2 | 1 | 1 | 0 | 1 | 3 | 3 | 1 | 10 |
| *Mirobaeus* | 0 | 0 | 0 | 1 | 1 | 2 | 1 | 1 | 0 | 1 | 3 | 3 | 1 | 10 |
| *Odontacolus* | 0 | 0 | 0 | 1 | 1 | 3 | 1 | 1 | 0 | 0 | 5 | 1 | 1 | 10 |
| *Parascelio* | 0 | 0 | 0 | 1 | 1 | 2 | 1 | 1 | 0 | 1 | 3 | 3 | 2 | – |
| *Sceliacanthella* | 0 | 0 | 0 | – | 3 | 1 | – | 0 | 3 | 1 | 3 | 2 | 3 | – |
| **Teleasinae** | | | | | | | | | | | | | | |
| *Teleas* | 0 | 0 | 0 | 0 | 1 | 1 | 1 | 0 | 0 | 0 | 3 | 2 | 3 | – |
| *Trimorus* 1 | 0 | 0 | 0 | 0 | 1 | 2 | 0 | 1 | 0 | 1 | 2,3 | 2 | 3 | 6 |
| *Trimorus* 2 | 0 | 0 | 0 | 0 | 1 | 2 | 0 | 1 | 1 | 1 | 3 | 2 | 3 | 6 |
| *Gryonoides* | 0 | 0 | 0 | 0 | 1 | 2 | 1 | 1 | 0 | 1 | 3 | 3 | 2 | – |
| *Xenomerus* | 0 | 0 | 0 | 0 | 1 | 1 | 0 | 0,1 | 0 | 1 | 3 | 3 | 1 | 6 |
| **Telenominae** | | | | | | | | | | | | | | |
| *Eumicrosoma* | 0 | 0 | 0 | 1 | 1 | – | – | 1 | 0 | 1 | 3 | – | – | 3 |
| *Paratelenomus* | 0 | 0 | 0 | – | 1 | – | – | 1 | 0 | 1 | 3 | – | – | 3 |
| *Phanuropsis* | 0 | 0 | 0 | 1 | 1 | – | – | 1 | 0 | 1 | 3 | – | 1 | 3 |
| *Psix* | 0 | 0 | 0 | 1 | 1 | 3 | 0 | 1 | 0 | 1 | 3 | 3 | 1 | 3 |
| *Telenomus* 1 | 0 | 0 | 0 | 1 | 1 | 3 | 1 | 1 | 0 | 1 | 3 | 3 | 1,2,3 | 3,5,7,8 |
| *Telenomus* 2 | 0 | 0 | 0 | 0 | 1 | 3 | 1 | 1 | 0 | 1 | 3 | 3 | 2,3 | 3,5,7,8 |
| *Trissolcus* | 0 | 0 | 0 | 0 | 1 | 2,3 | 1 | 1 | 0 | 1 | 2,3 | 4 | 1,2,3 | 3 |
| **Sceliotrachelinae** | | | | | | | | | | | | | | |
| *Fidiobia* | 3 | ? | ? | ? | 1 | 0 | 1 | 4 | 0 | 1 | 3 | 2 | 0 | 6 |
| *Platygastoides* | 3 | ? | ? | ? | 1 | 0 | 1 | 4 | 0 | 1 | 2 | 2 | 1 | – |
| *Zelandonota* | 2 | 1 | 1 | 0 | 1 | 0 | 1 | 4 | 0 | 1 | 3 | – | 3 | – |
| *Aphanomerella* | 3 | ? | ? | ? | 1 | – | 1 | 2 | 0 | 1 | – | – | 2 | 6 |
| *Aphanomerus* | 2 | 1 | 1 | 0 | 1 | 0 | 1 | 2 | 0 | 1 | 3 | 3 | 1 | 4 |
| *Austromerus* | 3 | ? | ? | ? | 1 | 0 | 0 | 2 | 0 | 2 | 3 | 3 | – | – |
| *Helava* | 3 | ? | ? | ? | 1 | 1 | 1 | 2 | 0 | 1 | 3 | 4 | 1,2 | – |
| *Parabaeus* | 3 | ? | ? | ? | 1 | 0 | 1 | 0 | 0 | 2 | 3,4 | 3 | 1 | – |
| *Tetrabaeus* | 3 | ? | ? | ? | 1 | – | 1 | 2 | 0 | 1 | – | – | – | 9 |
| *Isolia* | 3 | ? | ? | ? | – | – | 1 | 0 | 0 | 1 | 2 | 2,3 | 1,2,3 | – |

**Table 2.** continued

| | 1 | 2 | 3 | 4 | 5 | 6 | 7 | 8 | 9 | 10 | 11 | 12 | 13 | 14 |
|---|---|---|---|---|---|---|---|---|---|---|---|---|---|---|
| *Amitus* | 2 | 0 | 1 | 1 | 1 | 0 | 1 | 0 | 0 | 2 | 2 | 4 | 1 | 4 |
| *Allotropa* | 3 | ? | ? | ? | 1 | 0 | 1 | 0 | 0 | 1 | 2 | 2 | 3 | 4 |
| *Errolium* | 3 | ? | ? | ? | 1 | 1 | 1 | – | 0 | – | 2 | – | – | 4 |
| **Platygastrinae** | | | | | | | | | | | | | | |
| *Almargella* | 2 | 1 | 1 | 0 | 1 | 0 | 1 | 0 | 0 | 1 | 2 | 3 | 2 | – |
| *Magellanium* | 3 | ? | ? | ? | 1 | 0 | 1 | 0 | 0 | 1 | 0 | 3 | 1 | – |
| *Metaclisis* | 3 | ? | ? | ? | 1 | 0 | 1 | 2 | 0 | 1 | 2 | 3 | 1 | 8 |
| *Proplatygaster* | 3 | ? | ? | ? | 1 | 1 | 1 | 0 | 0 | 1 | 1 | 4 | 3 | 8 |
| *Isostasius* | 3 | ? | ? | ? | 0 | ? | 1 | 0 | 0 | 0 | 5 | 1 | – | 8 |
| *Acerotella* | 2 | 1 | 1 | 0 | 1 | 2 | 1 | 0 | 0 | 1 | 3 | 3 | 2 | 8 |
| *Inostemma* | 3 | ? | ? | ? | 1 | 1 | 1 | 0 | 0 | 1 | 3,4 | 3 | 1,2 | 8 |
| *Iphitrachelus* | 2 | 0 | 0 | 1 | 1 | 0 | 1 | 0 | 0 | 2 | 1 | 4 | 4 | – |
| *Alostemma* | 2 | 1 | 1 | 1 | 1 | 0 | 1 | 0 | 0 | 1 | 2 | 1 | 1 | – |
| *Amblyaspis* | 3 | ? | ? | ? | 1 | 0 | 1 | 0 | 0 | 2 | 0 | 4 | 1 | – |
| *Eritrissomerus* | 3 | ? | ? | ? | – | – | 1 | 0 | 0 | 2 | 2 | 4 | 3 | 8 |
| *Euxestonotus* | 3 | ? | ? | ? | 1 | 0 | 1 | 0 | 0 | 2 | 1 | 4 | 0 | 8 |
| *Isocybus* | 3 | ? | ? | ? | 1 | 0 | 1 | 0 | 0 | 2 | 1 | 5 | 2 | – |
| *Metanopedias* | 2 | 1 | 1 | 1 | 1 | 0 | 1 | 0 | 0 | 0 | 3 | 2 | 3 | 8 |
| *Platygaster* 1 | 3 | ? | ? | ? | 1 | 0 | 1 | 4 | 0 | 0 | 3 | 1,2 | 4 | 8 |
| *Platygaster* 2 (183) | 2 | 1 | 1 | 1 | 1 | 0 | 1 | 0 | 0 | 0 | 2,3 | 2 | 3 | 8 |
| *Trichacis* | 3 | ? | ? | ? | 1 | 0 | 1 | 0 | 0 | 2 | 2 | 5 | 1 | 8 |
| *Leptacis* | 3 | ? | ? | ? | 0 | ? | 1 | 0 | 0 | 2 | 0 | 5 | 3 | 8 |
| *Piestopleura* | 3 | ? | ? | ? | – | – | 1 | 0 | 0 | 1 | 2 | 5 | 2 | – |
| *Synopeas* 1 | 3 | ? | ? | ? | – | – | 1 | 0 | 0 | 1 | 2 | 5 | 2,3 | 8 |
| *Synopeas* 2 (*Sactogaster)* | 3 | ? | ? | ? | – | – | 1 | 0 | 0 | 0 | 5 | 1,2 | 3,4 | – |

scelionid genera the lateral apodemes are elongate, the latero-ventral apodemes are lost and S6 has a medial apodeme. In the 'Scelionini *s. l.*' the lateral apodemes have become incorporated into the wall of the telescope tube and have changed function, from points of muscle attachment to strengthening 'rods' in the telescopic tube. Only in *Mantibaria* are the latero-ventral apodemes elongate and then they are free from the lateral apodemes (Fig. 78). Several platygastrid genera (e.g. *Fidiobia, Platygastoides* and *Zelandonota*) have very short lateral and latero-ventral apodemes, as in the Sparasionini, but their attachment point has migrated to the apices (Fig. 140), while the majority of genera possess only lateral apodemes and they lack a medial apodeme of S6. Further, in all described platygastrids, with the exception of *Iphitrachelus* (Fig. 167), abdominal T9 has been lost or is greatly reduced and partly desclerotised so that the lateral apodemes form a U-shaped structure that is not fixed to any other tergal elements.

However, there are several problems with this character, not the least of which is that the homology of some states is in doubt; for instance, state 2 of Character 8 where, in some platygastrids, what are assumed to be the ventro-lateral apodemes are present as extensions of S6 (Figs 145, 159). Also, in some scelionids the position, morphological complexity and various degrees of fusion of the apodemes make it very difficult interpret both the arrangement and homology of the component structures (e.g. in *Leptoteleia, Opisthacantha, Styloteleia*, some *Gryon*, Figs 58, 60, 65, 91, respectively). For these reasons it has only been possible to treat this character in the most simplistic way. Although outgroup comparison indicates that state 2 is ancestral, it may be more realistic to code the state(s) associated with *Sparasion* and *Sceliomorpha* as plesiomorphic, as they are postulated to represent the ground plan. All other states would then be treated as apomorphic, including the apparent loss of apodemes in genera

such as *Isostasius*, *Synopeas*, *Leptacis* and *Piestopleura*. Clearly, among the characters that comprise the ovipositor system of platygastroid wasps, the arrangement and homology of the apodemes requires substantial further study. Further, although the above five groups (Fig. 189) are supported by apomorphic states associated with the ovipositor system, when a complete data matrix of all characters is compiled the status of these groups may change, for instance, the sister-group relationship of Sparasionini *s. str.* + Platygastridae.

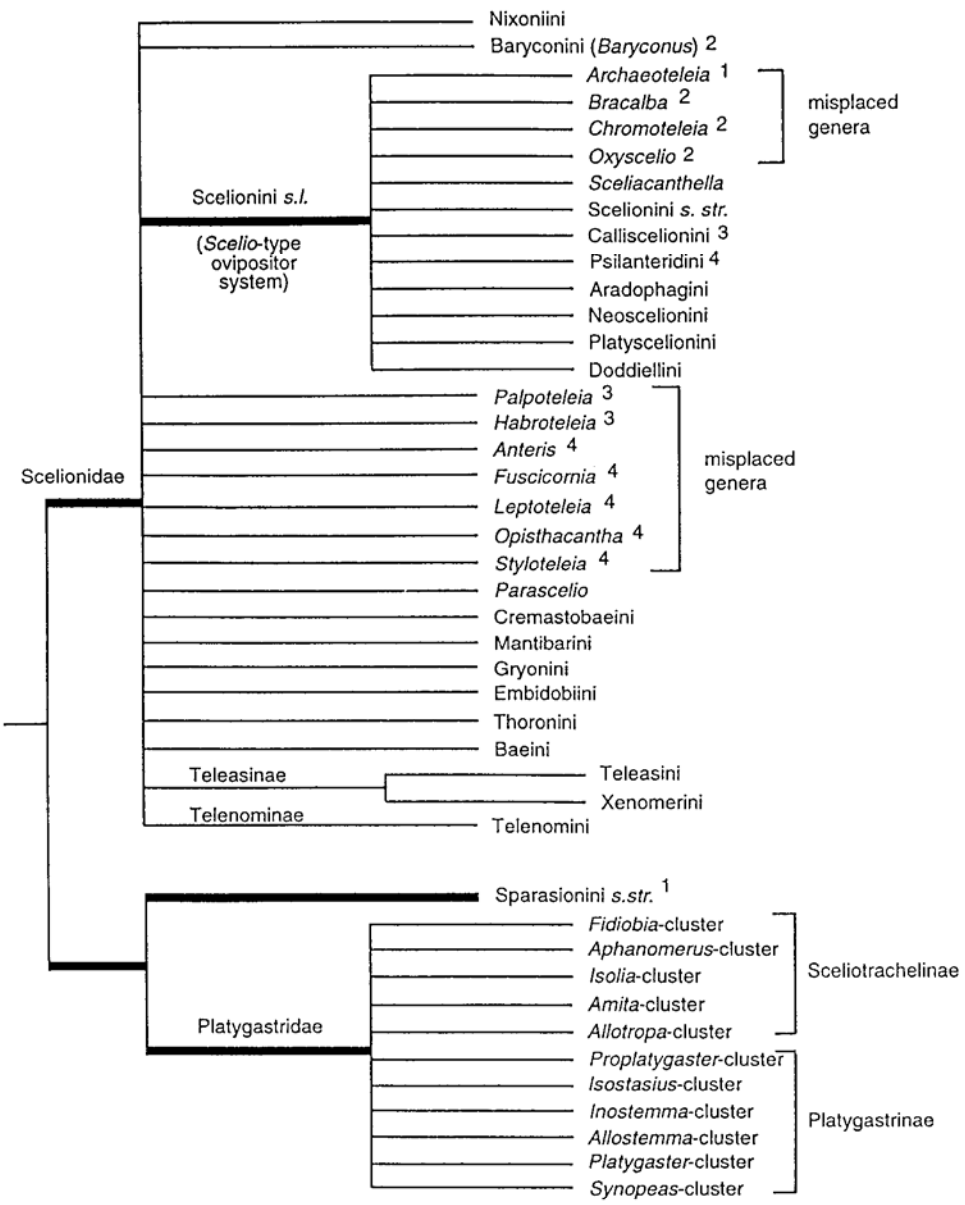


**Fig. 189.** Summary of the consensus tree of the 6300 shortest trees saved of 203 steps for the Platygastroidea with the simple TBR heuristic search option of PAUP 3.1.1 (CI = 0·254; RI = 0·536; RC = 0·136). Only the five clades shown by dark lines were resolved as monophyletic based on 14 ovipositor characters (see text for further explanation). The tribes and generic clusters shown on the tree were not resolved in the analysis. The superscript numbers indicate the tribes in which misplaced genera were previously accommodated.

**Discussion**

The morphology and mechanics of ovipositor extension and retraction in scelionid and platygastrid wasps is completely different from the system found in other parasitic Hymenoptera (Austin 1983; Field and Austin 1984). The most fundamental difference is the desclerotisation of the elongate medial part of abdominal segment 9 to form a collapsible membranous tube (i.e. tubular A9). The development of elongate internal apodemes and associated muscles in the *Ceratobaeus*-type system, and telescopic sections of membranous tube (i.e. intersegmental membrane between T6 and T7+8) and hydrostatic extension of the tube in the *Scelio*-type system, are also features unique to the superfamily. These adaptations greatly reduce the mechanical barriers to posterior extension and allow the ovipositor to be extended and retracted along the longitudinal line of the metasoma (Austin 1983). This is in contrast to other Microhymenoptera such as chalcidoids, where the tergum of A9 is relatively rigid so that the ovipositor is recessed beneath the metasoma and is sometimes partly coiled.

The ability of platygastroid wasps to move the ovipositor in the longitudinal line means that it is kept relatively straight and this provides a functional explanation for two features that have long been associated with the group: the development of a dorsal or ventral horn or hump that acts as a recess for the ovipositor, and the dorso-ventral flattening of the metasoma. In other microwasps the metasoma is very often dorsally convex or laterally compressed so as to provide space for the rotating elements of the ovipositor when it is extended ventrally. Only in very few platygastroid genera (*Odontacolus*, *Cyphacolus*, *Isostasius*, *Rao* and some *Synopeas*) is the ovipositor coiled or partly coiled and in these it is invaginated into the metasoma in a way typical of the superfamily. Such cases have undoubtedly evolved secondarily. Further, the loss of metasomal spiracles in the Platygastroidea can be explained functionally in that the mechanics of both the *Ceratobaeus*- and *Scelio*-type systems rely at least partly on hydrostatic pressure to extend and retract the ovipositor system. Any opening in the metasoma, such as spiracles, would act to release pressure when the haemocoel is squeezed (probably achieved by the contraction of dorso-ventral muscles connecting the terga and sterna), thereby greatly decreasing the efficiency of ovipositor movement. This would be most critical in the *Scelio*-type system that relies solely on hydrostatic pressure and where the telescopic tube is moved several times the length of the metasoma.

As discussed above, the ovipositor system of platygastroid wasps provides an important suite of characters that have substantial phylogenetic potential. This is particularly the case for those genera that possess a *Scelio*-type system. However, a robust phylogeny for the superfamily will only result from the analysis of a full range of morphological characters, in addition to the ovipositor system. It is also clear from recent higher-level treatments of the superfamily (e.g. Masner 1976, 1980*b*; Galloway and Austin 1984) that the monophyly of several long-standing groups should be questioned, particularly given that many of the characters previously used in diagnosing them are now known to be plesiomorphic. A case in point is the ranking of the Platygastridae as a family which, as pointed out by Masner and Huggert (1989), probably renders the Scelionidae a paraphyletic (or, possibly, polyphyletic) group. Therefore, as a prelude to a more comprehensive phylogenetic analysis of the Platygastroidea, we present here a discussion of the major higher-level taxa (focusing mostly on the Scelionidae) and consider whether or not they are supported by putative synapomorphies.

*Platygastroidea.* The monophyly of the superfamily has previously been discussed in little detail and the group has been variously diagnosed by authors (e.g. Richards and Davies 1977; Delvare and Aberlenc 1989; Naumann 1991; Masner 1993). Masner and Huggert (1989) and Masner (1993) have briefly outlined the importance of two characters, the sensilla on the antennal clavomeres and the ovipositor system, as the definitive synapomorphies for the superfamily. However, in the light of the present study the latter character is best viewed as a suite of independent characters and these have now been assessed for most genera in the two families.

The monophyly of the superfamily is supported by several synapomorphies, as follows: (1) ovipositor retracted internally within the metasoma and housed within an extended and desclerotised medial portion of abdominal segment 9 (tA9, Fig. 11); (2) ovipositor extension and retraction achieved by contraction of muscles associated with elongate apodemes and

assisted by changes in hydrostatic pressure, i.e. *Ceratobaeus*-type system (Austin 1983) (Fig. 11), or by hydrostatic pressure alone, i.e. *Scelio*-type ovipositor system (Field and Austin 1994) (Fig. 6); (3) metasomal spiracles lost; (4) antennal clavomeres of female with paired parallel basiconic-like sensilla (Bin 1981; Bin and Vinson 1986) (i.e. now termed multiporous gustatory sensilla; Isidoro *et al.* 1996); and (5) parasitoids of insect and spider eggs, primitively as idiobionts (all scelionids and some platygastrids), and secondarily as koinobionts (where development is delayed and the parasitoid completes its development within postembryonic stages of the host, in some platygastrids). The superfamily can be defined by three further characters (although their polarity is uncertain): (1) the tergum and sternum of metasomal segment 1 (A2) being free (not fused), (2) the fore wing venation greatly reduced, and (3) the toruli being contiguous with the upper epistomal suture of the clypeus, so that the antennae are positioned together and low on the head. The first of these, T1 and S1 being free, is apparently found elsewhere only in a few groups of chalcidoids and is fused or partly so in other Microhymenoptera. The fore wing venation represents a complex of characters in that the stigma is lost, at most only the submarginal, margin and stigmal veins (sometimes postmarginal vein) are tubular, and the stigmal vein is straight rather than curved. The loss of the stigma and/or shape of the stigmal vein distinguish the Platygastroidea from the Ceraphronoidea, although the fore wing venation of platygastroids is similar to many chalcidoids. The position of the toruli is shared with the Ceraphronoidea, as is the loss of metasomal spiracles, although the former character differs in detail between the groups, particularly in that the antennal insertions are usually widely spaced in the Ceraphronoidea and very close together in the Platygastroidea.

*Scelionidae.* Previously no synapomorphy has been found that defines this family and virtually all genera can be derived from its most plesiomorphic members (e.g. *Nixonia*, *Sparasion* and *Sceliomorpha*) by postulating various pathways for loss and/or reduction of characters. However, as discussed above the family (not including the Sparasionini *s. str.*) can be defined by one synapomorphy, the elongate lateral apodemes. Further, many genera, with the exception of those that possess a *Scelio*-type ovipositor system, also have a medial apodeme on S6.

*Scelioninae.* There are no synapomorphies that define this subfamily and the group is diagnosed on a combination of characters that are plesiomorphic, highly variable, or of unknown status.

*Sparasionini* sensu stricto (i.e. Sparasion+Sceliomorpha). The Sparasionini *s. str.* is supported by T9 being small and only partly sclerotised. They also have very short apodemes, as do the Platygastridae, which is considered to be a plesiomorphic character, as are most of the external traits that define the tribe (Masner 1976).

*Group possessing the* Scelio-*type ovipositor system – 'Scelionini s. l.'.* This group is deemed to be monophyletic based on the telescopic and hydrostatically extendable ovipositor system, and can be defined on those characters discussed above (Fig. 189). It comprises some 55 genera including all Scelionini *s. str.*, most of the Calliscelionini and Psilanteridini, the monogeneric tribes Aradophagini, Neoscelionini, Platyscelionini and Doddiellini, and several previously misplaced genera (i.e. *Archaeoteleia*, *Bracalba*, *Chromoteleia*, *Oxyscelio* and *Sceliacanthella*). The relationships within this newly recognised lineage are mostly unresolved and require attention in the light of the present study. Furthermore, the recognition of this group represents a major step towards developing a phylogenetically based classification for the Scelionidae. Given that the relationships among the tribes of Scelioninae and other lineages are yet to be resolved, as an interim measure we propose the informal group name 'Scelionini *sensu lato*' for this group, so that it can be readily identified without invoking premature and unsubstantiated changes to the higher classification of the superfamily. The name 'Scelionini *sensu stricto*' should then be applied to the tribe recognised by Masner (1976).

*Other groups within the Scelioninae.* Three small tribes recognised by Masner (1976), Nixoniini, Mantibariini and Cremastobaeini, are very distinctive and are defined by specific

autapomorphies or, in the case of *Nixonia*, mostly by plesiomorphies (see Masner 1976 for discussion; also see above). The Baryconini, on the basis of the ovipositor system, now includes only the nominal genus and *Apegus* although further research may reveal it to be a group that can naturally subsume some of the genera known to be misplaced from the Calliscelionini and Psilanteridini, or previously unplaced (i.e. *Anteris, Fusicornia, Habroteleia, Leptoteleia, Parascelio, Opisthacantha, Palpoteleia* and *Styloteleia*). However, at present there is no obvious synapomorphy for such a group.

*Gryonini.* Again, superficially this appears to be a distinct lineage, but it is not defined by an obvious synapomorphy. Nominally, the group is recognised by the short, broad metasoma, T2 nearly always the largest tergite, ovipositor assembly robust and moderately short (Galloway *et al.* 1992), and Heteroptera as the only known host group. All of these characters are found in other groups of scelionids.

*Embidobiini.* As for the Gryonini, this tribe is not defined by any unique characters, except for the host group (the eggs of embiids), which even by itself is probably phylogenetically significant. Otherwise, the group is characterised by having the ocelli distant from the orbits (shared with the Teleasinae), the eyes somewhat reduced in size, the postmarginal vein present in the fore wing, the tridentate mandible with the middle tooth smaller, and the body distinctly hairy. Galloway and Austin (1984) have postulated a sister-group relationship between the Baeini and Embidobiini based on their biology, namely, the production of silk around the egg mass by their host groups and the recognition of silk by wasps during host searching.

*Thoronini.* This group is characterised primarily by its biology (parasitising the eggs of aquatic Heteroptera), and the somewhat streamlined smooth body, which is often sparsely covered with long stout hairs, characteristics that are logically interpreted to be adaptations for this habitat. In addition, the clava of the female comprises four semi-compact or compact elements, a character shared with the Baeini and some platygastrids.

*Baeini.* This has been previously recognised as a distinctly homogeneous assemblage, but again, other than the host group (spider eggs), which can probably be considered as synapomorphous, the polarity of other distinguishing characters has not been rigorously determined. The Baeini have the female with the antennal clava composed of four very compact usually completely fused clavomeres; a character shared for instance with the Thoronini, and some platygastrids (e.g. *Aphanomerus, Helava, Parabaeus* and *Tetrabaeus*), where the clava often comprises four elements; the mandibles tridentate with a convex outer surface (L. Masner, personal communication), and the antennal scape not reaching above the anterior ocellus. On the basis of the host group and above characters, *Echthrodesis* should be accommodated in this tribe and not the Embidobiini (see Comments under the latter tribe).

*Teleasinae.* This group is remarkably homogeneous on the basis of the following characters, although none of them, other than the host group, can be unequivocally proposed to be synapomorphous. T3 is always the largest metasomal tergite, the marginal vein is elongate and much longer than the shortened stigmal vein; the hind wing is very broad with the vein almost contiguous with the fore margin; the lateral ocelli are usually positioned away from the margin of the orbits (but not in the *Trimorus ninus* species-group); and these wasps are parasitic on the eggs of Carabidae.

*Telenominae.* Members of this group are united on the absence of laterosternites on the metasoma, the presence of specialised sensory plates of T8 rather than cerci and, less importantly, the females having fewer flagellomeres than males (11 or 10 in females versus 12 in males, respectively). Although some sceliotracheline platygastrids also have the laterosternites absent, this is possibly a convergent loss. Further, although the host relationships of telenomines are varied, they show a trend from hemipteran eggs to lepidopteran eggs, with neuropteran and dipteran eggs being rarely attacked.

*Platygastridae.* This appears to be a well-supported group, albeit mostly on reductional characters. The major synapomorphies for the family are (1) abdominal tergite 9 (T8) reduced, desclerotised and held internally, or more normally completely absent; (2) absence of any sensory structures on T8 (Masner and Huggert 1989), and (3) metapleuron (sometimes also propodeum and T1) with dense carpeting of hairs. In only one taxon, an undescribed genus from Chile (CNCI), is T8 apparently held externally, moderately well sclerotised, but has no sensory structures present (L. Masner, personal communication). In addition to these synapomorphies, the family can be diagnosed on three other characters, although all can be found within the Scelionidae. These are metasomal T2 developed as the largest tergite, and two reductional characters, the antennae of both sexes having 10 or fewer flagellomeres, and the palpal formula being 2-1 or 1-1. Relationships within the Platygastridae are generally not well resolved, although some putative monophyletic elements have been recognised and discussed by Masner and Huggert (1989) (also see Comments above under each generic cluster). Importantly, Masner and Huggert (1989) demonstrate that part of the former subfamily Inostemmatinae are defined on plesiomorphic characters, and they provide substantial stability at generic level by accurately diagnosing over 40 genera, 20 of which are described as new. However, the higher-level clasification of the family is still problematic and, like the Inostemmatinae, the Sceliotrachelinae may still be plesiomorphic compared with the Platygastrinae. The major synapomorphy of the latter group is its restricted biology, viz. parasitoids of cecidomyiids (see Table 1).

*Future Research*

Because of the holistic nature of this project, it has not been possible to examine multiple species of every genus (see the Appendix), and so there is little or no information available on the true level of interspecific variation for some genera. In cases where multiple species within a genus have been studied (e.g. *Ceratobaeus, Gryon* and *Idris*) there is ample evidence to indicate that substantial variation exists for some characters and this may be useful at the species level or in recognising subgeneric groupings. Indeed, differences among some ovipositor characters (e.g. among species of *Anteris, Opisthacantha, Telenomus, Trimorus* and *Platygaster*) indicate that the monophyly of these genera should be questioned. Detailed studies focusing on the ovipositor system might provide the basis for dividing such genera into more natural groups, where external characters by themselves have largely failed. Also, as discussed above (under Preliminary Analysis), there are substantial problems with interpreting the character states associated with the apodemes and determining their homology. Detailed morphological studies on this character system may therefore yield results of both taxonomic and phylogenetic importance.

The matrix of character states compiled here for the ovipositor system and related structures (Table 2) is comprehensive in that it presents information for over 110 platygastroid genera. However, there is still a significant amount of missing data, particularly associated with the ovipositor assembly from the rarer scelionid genera (e.g. some Embidobiini and Thoronini), very small platygastrids that proved difficult to dissect, and host data (unavailable for about half the genera examined here). Although attempting broad-scale rearing of insect eggs to generate new host records maybe somewhat unrealistic in time and effort, new data in the future may be forthcoming by targeting specific host groups, such as acridid, tettigoniid and coleopteran eggs. This has been a particularly useful strategy in expanding the known host range of the Baeini (Austin 1985), and to a lesser extent the Embidobiini (Masner 1980*b*) and Thoronini (Masner 1972*a*). In this respect, interacting with biologists who work on such groups may be a profitable way of acquiring new host data. For very small platygastrids, more tedious methods such as histological sectioning may be required to fully elucidate the morphology of the ovipositor system.

Finally, there is undoubtedly still much to be learnt about the oviposition behaviour of platygastroid wasps and the functional mechanics of the ovipositor system. These aspects have been examined only partially for fewer than five species in two genera, *Ceratobaeus* and *Scelio* (Austin 1983; Field and Austin 1994). Although these studies have revealed much about how the ovipositor system operates in these and related genera, it is difficult to imagine that the diversity of morphological structures observed among tribes and genera in the superfamily

operate in the same way. For instance, the ovipositor system of the *Synopeas*-cluster is worthy of detailed study, given that members of this group apparently lack apodemes for the attachment of extensor and retractor muscles.

## Acknowledgments

This project would not have been possible without the continued input of Lubomir Masner over the last 12 years. We thank him for his discussions, free exchange of information and ideas, the substantial amount of material he donated to the project, and his constructive comments on a draft of the manuscript. We also thank Gary Gibson, Norman Johnson and Ian Naumann for their discussions on the hymenopteran ovipositor system, Jo Berry for additional specimens, Paul Dangerfield for inking the final drawings, taking the scanning electron micrographs and assistance with the phylogenetic analysis, and Jenny Groom for printing the photographs. Financial support for the project was originally obtained by the senior author under a Anglo-Canadian Exchange Scholarship from the Royal Society, London, and later from the Australian Research Council and The University of Adelaide.

## References

Ashmead, W. H. (1893). A monograph on the North American Proctotrypidae. *United States National Museum Bulletin* **45**, 1–472.

Ashmead, W. H. (1901). Classification of the pointed-tail wasps of the superfamily Proctotrypoidea. Part I. *Journal of the New York Entomological Society* **10**, 240–247.

Ashmead, W. H. (1903). Classification of the pointed-tail wasps of the superfamily Proctotrypoidea. Part II. *Journal of the New York Entomological Society* **11**, 86–99.

Austin, A. D. (1983). Morphology and mechanics of the ovipositor system of *Ceratobaeus* Ashmead (Hymenoptera: Scelionidae) and related genera. *International Journal of Insect Morphology and Embryology* **12**, 139–155.

Austin, A. D. (1984). New species of Platygastridae (Hymenoptera) from India which parasitise pests of mango, particularly *Procontarinia* spp. (Diptera: Cecidomyiidae). *Bulletin of Entomological Research* **74**, 549–557.

Austin, A. D. (1985). The function of spider egg sacs in relation to parasitoids and predators, with special reference to the Australian fauna. *Journal of Natural History* **19**, 359–376.

Austin, A. D. (1986). A taxonomic revision of the genus *Mirobaeoides* Dodd (Hymenoptera: Scelionidae). *Australian Journal of Zoology* **34**, 315–337.

Austin, A. D. (1988). A new genus of baeine wasp (Hymenoptera: Scelionidae) from New Zealand associated with moss. *New Zealand Journal of Zoology* **15**, 173–183.

Austin, A. D. (1990). A revision of the Old World *Parabaeus* Kieffer (Hymenoptera: Platygastridae), a group of highly modified apterous parasitoids inhabiting litter. *Invertebrate Taxonomy* **3**, 647–659.

Baumann, C. (1938). Über den Legeapparat von *Baryconus rufipes* Kieff. (Hym. Proct.) und seine Beziehungen zum Adbominalhöcker. 7th International Congress of Entomology, Vol. 2, pp. 653–666.

Bin, F. (1976). New host records in *Baryconus* Foerster (Hymenoptera, Scelionidae). *Entomologica (Bari)* **9**, 63–66.

Bin, F. (1979). *Encyrtoscelio apertus* (Szel.) (Hym., Scelionidae) egg parasite of *Cydnus aterrimus* Foerst. (Het., Cydnidae). *Bollettino del Laboratorio di Entomologia Agraria Fillipo Silvestri di Portici* **36**, 3–7.

Bin, F. (1981). Definition of female antennal clava based on its plate sensilla in Hymenoptera Scelionidae Telenominae. *Redia* **64**, 245–261.

Bin, F., and Vinson, S. B. (1986). Morphology of the antennal sex glands in male *Trissolcus basalis* (Woll.) (Hymenoptera: Scelionidae), an egg parasitoid of the green stink bug, *Nezara viridula* (Hemiptera: Pentatomidae). *International Journal of Insect Morphology and Embryology* **15**, 359–376.

Caleca, V. (1992). New data on *Breviscelio* Sundholm, with description of a new species from South Africa (Hymenoptera, Scelionidae). *Phytophaga* **4**, 49–55.

Caleca, V., and Mineo, G (1993). On the genus *Dyscritobaeus* Perkins: 1910 (Hymenoptera, Proctotrupoidea: Scelionidae). *Bollettino del Laboratorio di Entomologia Agraria Filippo Silvestri di Portici* **50 (1995)**, 9–21.

Carlow, T. (1992). *Thoronella* sp. (Hymenoptera: Scelionidae) discovered on the thorax of an Aeschnidae (Anisoptera). *Notulae Odontatologicae* **3**, 137–156.

Chopard, L. (1923). Les parasites de la mante religieuse. *Annales de la Societé Entomologie de France* **91**, 249–274.

Copland, M. J. (1976). Female reproductive system of the Aphelinidae (Hymenoptera: Chalcidoidea). *International Journal of Insect Morphology and Embryology* **5**, 151–166.

Copland, M. J. W., and King, P. E. (1971*a*). The structure of the female reproductive system in the Chalcididae (Hym.). *Entomologists Monthly Magazine* **107**, 230–239.

Copland, M. J. W., and King, P. E. (1971*b*). The structure and possible function of the reproductive system in some Eulophidae and Tetracampidae. *Entomologist (London)* **104**, 4–28.

Copland, M. J. W., and King, P. E. (1972*a*). The structure of the female reproductive system in the Torymidae (Hymenoptera: Chalcidoidea). *Transactions of the Royal Entomological Society, London* **124**, 191–212.

Copland, M. J. W., and King, P. E. (1972*b*). The structure of the female reproductive system in the Eurytomidae (Chalcidoidea: Hymenoptera). *Journal of Zoology (London)* **166**, 185–212.

Copland, M. J. W., and King, P. E. (1972*c*). The structure of the female reproductive system in the Pteromalidae (Chalcidoidea: Hymenoptera). *Entomologist (London)* **105**, 77–96.

Copland, M. J. W., King, P. E., and Hill, D. S. (1973). The structure of the female reproductive system in the Agaonidae (Chalcidoidea, Hymenoptera). *Journal of Entomology (Series A)* **48**, 25–35.

Delvare, G., and Aberlenc, H.-P. (1989). 'Les Insectes d'Afrique et d'Amerique Tropicale. Clés pour la Reconnaissance des Families.' (PRIFAS, CIRAD-GERDAT: Montpellier).

Dessart, P. (1969). Description complémentaire d'*Uroscelio luteipes* Kieffer, 1914 (Hymenoptera: Proctotrupoidea: Scelionidae). *Proceedings of the Entomological Society, Washington* **71**, 328–335.

Dicke, F. F. (1937). *Eumicrosoma benefica* Gahan as an egg parasite of the hairy chinch bug. *Journal of Economic Entomology* **30**, 376.

Dodd, A. P. (1913). Australian Hymenoptera Proctotrypoidea No. 1. *Transactions of the Royal Society of South Australia* **37**, 130–181.

Dodd, A. P. (1914). Australian Hymenoptera Proctotrypoidea No. 2. *Transactions of the Royal Society of South Australia* **38**, 58–131.

Dodd, A. P. (1915). Australian Hymenoptera Proctotrypoidea No. 3. *Transactions of the Royal Society of South Australia* **39**, 384–454.

Dodd, A. P. (1926). Australian Hymenoptera Proctotrypoidea No. 5. *Transactions of the Royal Society of South Australia* **50**, 298–314.

Dodd, A. P. (1939). Hymenopterous parasites of Embioptera. *Proceedings of the Linnean Society of New South Wales* **50**, 298–314.

Dowton, M., Austin, A. D., Dillon, N., and Bartowsky, E. (in press). Molecular phylogeny of the apocritan wasps with particular reference to the Proctotrupomorpha and Evaniomorpha. *Systematic Entomology*.

Eberhard, W. G. (1975). The ecology and behavior of a subsocial pentatomid bug and two scelionid wasps: strategy and counter strategy in a host and its parasites. *Smithsonian Contributions to Zoology* **205**, 1–39.

Fergusson, N. D. M. (1988). A comparative study of the structures of phylogenetic importance of female genitalia of the Cynipoidea (Hymenoptera). *Systematic Entomology* **13**, 12–30.

Field, S. A., and Austin, A. D. (1994). Morphology and mechanics of the ovipositor system of *Scelio* Latreille (Hymenoptera: Scelionidae) and related genera. *International Journal of Insect Morphology and Embryology* **23**, 135–158.

Galloway, I. D., and Austin, A. D. (1984). Revision of the Scelioninae (Hymenoptera: Scelionidae) in Australia. *Australian Journal of Zoology Supplementary Series* **99**, 1–138.

Galloway, I. D., Austin, A. D., and Masner, L. (1992). Revision of the genus *Neuroscelio* Dodd, primitive scelionids (Hymenoptera: Scelionidae) from Australia, with a discussion of the ovipositor system of the tribe Gryonini. *Invertebrate Taxonomy* **6**, 523–545.

García, J. L., and Masner, M. (1994). A redefinition of *Aradophagus* (Hymenoptera: Scelionidae), with a key to described species. *Canadian Entomologist* **126**, 67–74.

Gauld, I., and Bolton, B. (Eds) (1988). 'The Hymenoptera.' [Oxford University Press: New York and British Museum (Natural History): London.]

Hellén, W. (1971). Die Scelioniden Finnlands (Hymenoptera: Proctotrupoidea). *Fauna Fennica* No. 23.

Huggert, L. (1980). Taxonomical studies on some genera and species of Platygastrinae (Hymenoptera: Proctotrupoidea). *Entomologica Scandinavica* **11**, 97–112.

Huggert, L., and Masner, L. (1983). Two new genera of African scelionid wasps (Hymenoptera, Proctotrupoidea: Scelionidae). *Entomologica Scandinavica* **14**, 173–185.

Isidoro, N., Bin, F., Colazza, S., and Vinson, S. B. (1996). Morphology of antennal gustatory sensilla and glands in some parasitoid Hymenoptera with hypothesis on their role in sex and host recognition. *Journal of Hymenoptera Research* **5**, 206–239.

Johnson, N. F. (1984*a*). Systematics of Nearctic *Telenomus*: classification and revisions of the *podisi* and *phymatae* species groups (Hymenoptera: Scelionidae). *Bulletin of the Ohio Biological Survey* **6**, 1–113.

Johnson, N. F. (1984*b*). Revision of the Nearctic species of the *Telenomus flavipes* group (Hymenoptera: Scelionidae). *Proceedings of the Entomological Society of Washington* **86**, 797–807.

Johnson, N. F. (1985). Phylogenetic relationships of the telenomine genus *Nirupama* (Hymenoptera: Scelionidae). *International Journal of Entomology* **27**, 369–374.

Johnson, N. F. (1987). The Neotropical telenomine genus *Phanuropsis* Girault (Hymenoptera: Scelionidae). *Annals of the Entomological Society of America* **80**, 660–663.

Johnson, N. F., and Bin, F. (1982). Species of *Telenomus* (Hym., Scelionidae), parasitoids of stalked eggs of Neuroptera (Chrysopidae & Berothidae). *Redia* **65**, 189–206.

Johnson, N. F., and Masner, L. (1985). Revision of the genus *Psix* Kozlov & Le (Hymenoptera: Scelionidae). *Systematic Entomology* **10**, 33–58.

Kieffer, J. J. (1926). Hymenoptera. Proctotrupoidea. Scelionidae. *Das Tierreich* **48**, 1–885.

King, P. E. (1962). The muscular structure of the ovipositor and its mode of function in *Nasonia vitripennis* (Walker) (Hymenoptera: Pteromalidae). *Proceedings of the Royal Entomological Society, London* **37**, 121–128.

King, P. E., and Copland, M. J. W. (1969). The structure of the female reproductive system in the Mymaridae (Chalcidoidea: Hymenoptera). *Journal of Natural History* **3**, 349–365.

Kozlov, M. A. (1970). Supergeneric groupings of Proctotrupoidea (Hymenoptera). *Entomologischeskoe Obozrenie* **49**, 203–226 (In Russian) *Entomological Review* **49**, 115–127 (In English).

Krombein, K. V. (1964). Natural History of Plummers Island, Maryland. XVIII. The hibiscus wasp, an abundant rarity and its associates (Hymenoptera : Sphecidae). *Proceedings of the Biological Society of Washington* **77**, 73–112.

Maddison, W. P., and Maddison, D. R. (1992). 'MacClade: Analysis of Phylogeny and Character Evolution. Version 3.' (Sinauer Associates Inc.: Massachusetts.)

Masner, L. (1956). First preliminary report on the occurrence of genera of the group Proctotrupoidea (Hymenoptera) in CRS (first part - family Scelionidae). *Acta Faunistica Entomologica Musei Nationalis Prague* **1**, 99–126.

Masner, L. (1964). Remarks on *Sceliotrachelus* Brues (Hymenoptera, Platygastridae). *Psyche* **71**, 8–11.

Masner, L. (1968*a*). A new genus of Scelionidae (Hymenoptera) with austral disjunctive distribution. *New Zealand Journal of Science* **11**, 652–663.

Masner, L. (1968*b*). A new scelionid wasp from the intertidal zone of South Africa (Hymenoptera: Scelionidae). *Annals of the Natal Museum* **20**, 195–198.

Masner, L. (1972*a*). The classification and interrelationships of Thoronini (Hymenoptera: Proctotrupoidea, Scelionidae). *Canadian Entomologist* **104**, 833–849.

Masner, L. (1972*b*). A new genus of Scelionidae from Trinidad, W.I. (Hymenoptera: Proctotrupoidea). *Canadian Entomologist* **104**, 1213–1216.

Masner, L. (1976). Revisionary notes and keys to world genera of Scelionidae (Hymenoptera: Proctotrupoidea). *Memoirs of the Entomological Society of Canada* **97**, 1–87.

Masner, L. (1978). A revision of the New World species of *Leptoteleia* Kieffer (Hymenoptera: Scelionidae), egg parasites of crickets. *Canadian Entomologist* **110**, 353–380.

Masner, L. (1979). Pleural morphology in scelionid wasps (Hymenoptera: Scelionidae) – an aid to higher classification. *Canadian Entomologist* **111**, 1079–1087.

Masner, L. (1980*a*). A revision of the Nearctic species of *Calotelea* Westwood (Hymenoptera, Proctotrupoidea, Scelionidae). *Canadian Entomologist* **112**, 397–408.

Masner, L. (1980*b*). Key to genera of Scelionidae of the Holarctic Region, with descriptions of new genera and species (Hymenoptera: Proctotrupoidea). *Memoirs of the Entomological Society of Canada* **113**, 1–54.

Masner, L. (1980*c*). The Nearctic species of *Acerotella* Masner (Hymenoptera, Proctotrupoidea, Platygastridae). *Canadian Entomologist* **112**, 1291–1303.

Masner, L. (1981). Revision of the Nearctic species of *Metaclisis* Foerster (Hymenoptera, Proctotrupoidea, Inostemmatinae). *Canadian Entomologist* **113**, 1069–1091.

Masner, L. (1983*a*). The genus *Oethecoctonus* Ashmead in North America (Hymenoptera: Proctotrupoidea: Scelionidae). *Canadian Entomologist* **115**, 17–24.

Masner, L. (1983*b*). A revision of *Gryon* Haliday in North America (Hymenoptera: Proctotrupoidea: Scelionidae). *Canadian Entomologist* **115**, 123–174.

Masner, L. (1983*c*). Revision of the Nearctic species of *Trichacis* Foerster (Hymenoptera: Proctotrupoidea: Platygastridae). *Canadian Entomologist* **115**, 1071–1093.

Masner, L. (1991). The Nearctic species of *Duta* Nixon (Hymenoptera: Scelionidae), egg parasitoids of ground crickets (Orthoptera: Gryllidae). *Canadian Entomologist* **123**, 777–793.

Masner, L. (1993). Superfamily Platygastroidea. In 'Hymenoptera of the World: An Identification Guide to Families'. (Eds H. Goulet and J. T. Huber.) pp. 558–565. (Agriculture Canada: Ottawa.)

Masner, L. (in press). Revision of *Opisthacantha* and *Lapitha* of America north of Mexico (Hymenoptera: Scelionidae). *Canadian Entomologist*.

Masner, L., and Dessart, P. (1967). La reclassification des catégories taxonomiques supérieures des Ceraphronoidea (Hymenoptera). *Bulletin d'Institut Royal des Sciences Naturelles de Belgique* **43**, 1–33.

Masner, L., and Dessart, P. (1972). Notes on Embidobiini (Scelionidae: Hymenoptera) with description of a new genus. *Canadian Entomologist* **104**, 505–510.

Masner, L., and Huggert, L. (1979*a*). Descriptions of new taxa of Thoronini (Hymenoptera, Proctotrupoidea, Scelionidae). *Canadian Entomologist* **111**, 911–917.

Masner, L., and Huggert, L. (1979*b*). Revision of the world species of Aradophagini (Hymenoptera: Scelionidae). *Canadian Entomologist* **111**, 1089–1100.

Masner, L., and Huggert, L. (1989). World review and keys to genera of the subfamily Inostemmatinae with reassignment of the taxa to the Platygastrinae and Sceliotrachelinae (Hymenoptera: Platygastridae). *Memoirs of the Entomological Society of Canada* **147**, 1–214.

Mason, W. R. M. (1983). The abdomen of *Vanhornia eucnemidarum* (Hymenoptera: Proctotrupoidea). *Canadian Entomologist* **115**, 1483–1488.

Mason, W. R. M. (1984). Structure and movement of the abdomen of *Pelecinus polyturator* (Hymenoptera: Pelecinidae). *Canadian Entomologist* **116**, 419–426.

Mineo, G. (1982). Studies on the Scelionidae (Hym. Proctotrupoidea) XV. World distribution of *Maruzza* gen. n. *Bollettino del Laboratorio di Entomologia Agraria Fillipo Silvestri di Portici* **39**, 163–174.

Muesebeck, C. F. W. (1972). Nearctic species of Scelionidae (Hymenoptera, Proctotrupoidea) that parasitize the eggs of grasshoppers. *Smithsonian Contribution to Zoology* **122**, 1–33.

Muesebeck, C. F. W. (1977). The parasitic wasps of the genus *Macroteleia* Westwood of the world (Hymenoptera, Proctotrupoidea: Scelionidae). *U.S.D.A. Technical Bulletin* **1565**, 1–57.

Muesebeck, C. F. W. (1979). Superfamily Proctotrupoidea. In 'Catalog of Hymenoptera in America North of Mexico'. (Eds K. V. Krombein, P. D. Hurd Jr, D. R. Smith and B. D. Burks.) pp. 1121–1186. (Smithsonian Institute Press: Washington, DC.)

Naumann, I. D. (1982). Systematics of the Australian Ambositirnae (Hymenoptera: Diapriidae), with a synopsis on non-Australian genera of the subfamily. *Australian Journal of Zoology Supplementary Series* **85**, 1–239.

Naumann, I. D. (1991). Hymenoptera. In 'The Insects of Australia'. 2nd Edn. pp. 916–1000. (Melbourne University Press and CSIRO: Melbourne.)

Nixon, G. E. J. (1937). A new genus belonging to the subfamily Platygasterinae (Hym., Proctotrupoidea). *Annals of the Magazine of Natural History Series 11* **9**, 372–376.

Oeser, R. (1961). Ovipositor der Hymenoptera. IV. Vergleichende Untersuchungen ueber die Teile des Ovipositors. *Mitteilungen aus dem Zoologischen Museum in Berlin* **37**, 46–61.

Quicke, D. L. J., Fitton, M. G., and Ingram, S. (1992). Phylogenetic implications of the structure and distribution of ovipositor valvilli in the Hymenoptera (Insecta). *Journal of Natural History* **26**, 587–608.

Quicke, D. L. J., Fitton, M. G., Tunstead, J. R., Ingram, S., and Gaitens, P. V. (1994). Ovipositor structure and relationships within in the Hymenoptera, with special reference to the Ichneumonoidea. *Journal of Natural History* **28**, 635–682.

Rasnitsyn, A. P. (1988). An outline of evolution of the hymenopterous insects (Order Vespida). *Oriental Insects* **22**, 115–145.

Richards, O. W., and Davies, R. G. (1977). 'Imm's General Textbook of Entomology.' 2 vols, 10th Edn. (Chapman and Hall: London.)

Scudder, G. G. E. (1961*a*). The functional morphology and interpretation of the insect ovipositor. *Canadian Entomologist* **93**, 267–271.

Scudder, G. G. E. (1961*b*). The comparative morphology of the insect ovipositor. *Transactions of the Royal Entomological Society, London* **113**, 25–40.

Scudder, G. G. E. (1971). Comparative morphology of insect genitalia. *Annual Review of Entomology* **16**, 379–406.

Severin, H. C. (1935). The common black field cricket a serious pest in South Dakota. *Bulletin of the South Dakotan Agricultural Experimental Station* **295**, 1–51.

Smith, E. L. (1970). Evolutionary morphology of the external insect genitalia. 2. Hymenoptera. *Annals of the Entomological Society of America* **63**, 1–27.

Swofford, D. L. (1993). 'PAUP: Phylogenetic Analysis Using Parsimony. Version 3.1.1.' (Computer program distributed by Illinois Natural History Survey: Champaign, Illinois.)

Szabó, J. B. (1966). Ökologische ethologische, tiergeographische und systematische Untersuchungen an paläarktischen Gryoninen (Hymenoptera: Proctotrupoidea, Scelionidae). *Acta Zoologica Academiae Scientiarum Hungaricae* **12**, 419–449.

Vlug, H. J. (1995). 'Catalogue of the Platygastridae (Platygastroidea) of the World (Insecta: Hymenoptera).' Part 19: Hymenopterorum Catalogus. (Ed. C. van Achterberg.) (SPB Academic Publishing: The Hague.)

**Index to Taxonomic Names** (that appear in the text)

*Abuko* Masner & Huggert 32
*Acanthoscelio* Ashmead 11
*Acerotella* Masner 60
*Aceroteta* Kozlov & Masner 59
*Aleyroctonus* Masner & Huggert 55
*Alfredella* Masner & Huggert 55
*Allostemma* Masner & Huggert 61
*Allostemma*-cluster 61
*Allotropa* Foerster 56
*Allotropa*-cluster 56
*Almargella* Masner & Huggert 56
*Amblyaspis* Foerster 62
*Amitus* Haldeman 55
*Amitus*-cluster 55
*Annettella* Masner & Huggert 59
Anteridini Hellén 40
*Anteris* Foerster 26
*Anteromorpha* Dodd 20
*Apegus* Foerster 20
*Aphanomerella* Dodd 53
*Aphanomerus* Perkins 52
*Aphanomerus*-cluster 52
Aradophagini Kozlov 31
*Aradophagus* Ashmead 31
*Archaeoteleia* Masner 10
*Austromerus* M. & H 52

Baeini Ashmead 42
*Baeus* Haliday 42
Baryconini Kozlov 17
*Baryconus* Foerster 17
*bilineatus* Thomson, *Anteris* 26
*Bracalba* Dodd 18
*Breviscelio* Sundholm 39

*Calliscelio* Ashmead 20
Calliscelionini Masner 20
*Calotelea* Westwood 29
*canadensis* (Ashmead), *Trimorus* 49
*Ceratobaeus* Ashmead 44, 7
*Chromoteleia* Ashmead 18
*Crama* Galloway 24
Cremastobaeini Masner 33
*Cremastobaeus* Ashmead 33
*cuspicornutus* Austin, *Ceratobaeus* 44
*Cylindrogaster* Huggert 65
*Cyphacolus* Priesner 44

*Dichoteleas* Kieffer 24
*Dicroscelio* Kieffer 11
*Doddiella* Kieffer 36
Doddiellini Masner 36
*Duta* Nixon 24
*Dyscritobaeus* Perkins 39

*Echthrodesis* Masner 39
*Embidobia* Ashmead 39
Embidobiini Kozlov 39
*Embioctonus* Masner 39
*Encrytoscelio* Dodd 39
*Endecascelio* Masner & Dessart 39
*Eritrissomerus* Ashmead 62
*Errolium* Masner & Huggert 56
*Eumicrosoma* Gahan 50
*Euxestonotus* Fouts 64

*Fidiobia* Ashmead 51
*Fidiobia*-cluster 51
*flavipes* (Thomson), *Trissolcus* 51
*Fusicornia* Risbec 27, 24

*Genatropis* Galloway 29
*Gryon* Haliday 36
Gryonini Szabó 36
*Gryonoides* Dodd 46

*Habroteleia* Kieffer 24
*Harringtonia* Masner 26
*Helava* Masner & Huggert 53
*Heptascelio* Kieffer 11
*Hickmanella* Austin 44
*Holoteleia* Kieffer 22

*Idris* Foerster 44
*Inostemma* Haliday 59
*Inostemma*-cluster 59
*Iphitrachelus* Walker 60
*Isocybus* Foerster 64
*Isolia* Foerster 53
*Isolia*-cluster 53
*Isostasius* Foerster 57
*Isostasius*-cluster 57

*Ladora* Masner & Huggert 32
*Leptacis* Foerster 66
*Leptoteleia* Kieffer 27
*Lidgbirdius* Galloway 29
*Lispoteleia* Galloway 22

*Macroteleia* Westwood 22
*Magellanium* Masner & Huggert 57
*Mallateleia* Dodd 29
*Mantibaria* Kirby 34
Mantibariini Kozlov 34
*Mecix* Masner 29
*mellipes* Ashmead, *Opisthacantha* 27
*Merriwa* Dodd 26
*Metaclisis* Foerster 57
*Metanopedias* Brues 64
*Microthoron* Masner 40
*Mirobaeoides* Dodd 42
*Mirobaeus* Dodd 44
*Mirotelenomus* Dodd 39

*Nanomerus* Masner & Huggert 55
*Neobia* Masner & Huggert 52
*Neoscelio* Dodd 33
Neoscelionini Kozlov 33
*Neuroscelio* Galloway, Austin & Masner 39
*Nirupana* Nixon 50
*Nixonia* Masner 8
Nixoniini Masner 8
*Nyleta* Dodd 29

**Index.** continued


*Odontacolus* Kieffer 44
*Oethecoctonus* Ashmead 22
*Oligomerella* Masner & Huggert 52
*Opisthacantha* Ashmead 27
*Oreiscelio* Kieffer 11
*Oxyscelio* Kieffer 18
*Oxyteleia* Kieffer 29

*Palaeogryon* Masner 39
*Palpoteleia* Kieffer 20
*Parabaeus* Kieffer 53
*Paraduta* Szabó 29
*Parascelio* Dodd 44
*Paratelenomus* Dodd 50
*Paridris* Kieffer 22
*Phanuromyia* Dodd 50
*Phanuropsis* Girault 50
*Phoenoteleia* Kieffer 22
*Piestopleura* Foerster 66
*Platygaster* Latreille 61
*Platygaster*-cluster 61
*Platygastoides* Dodd 51
*Platyscelio* Kieffer 34
Platyscelionini Kozlov 34
*Plutomerus* Masner & Huggert 52
*podisi*-group, *Telenomus* 50
*Probaryconus* Kieffer 22, 7
*Proplatygaster* Kieffer 56
*Proplatygaster*-cluster 56
*Pseudanteris* Fouts 40
*Pseudoheptascelio* Szabó 11
Psilanteridini Kozlov 24
*Psilanteris* Kieffer 24
*Psix* Kozlov & Le 50

*Rao* Masner & Huggert 59

*Sacespalus* Kieffer 60
Sactogaster 65
*Sceliacanthella* Dodd 45
*Scelio* Latreille 11, 7
*Sceliomorpha* Ashmead 10
Scelionini Foerster 11
*Sceliotrachelus* Brues 55
*Shreemana* Nixon 29
*Sparasion* Latreille 10
Sparasionini Masner 10
*Spiniteleia* Masner 26
*Styloteleia* Kieffer 29
*Synoditella* Muesebeck 11
*Synopeas* Foerster 66
*Synopeas*-cluster 66

*Teleas* Latreille 45
Teleasini Ashmead 45
Telenomini Thomson 49
*Telenomus* Haliday 49
*Tetrabaeus* Kieffer 53
*Thoron* Haliday 40
*Thoronella* Masner 29
Thoronini Kozlov 40
*Tiphodytes* Bradley 40
*Trichacis* Foerster 64
*Trichacoides* Dodd 65
*Trimorus* Foerster 45
*Trisacantha* Ashmead 46
*Trissolcus* Ashmead 49
*Triteleia* Kieffer 22
*tubulosa* Brues, *Cylindrogaster* 65

Xenomerini Kozlov 46
*Xenomerus* Walker 46

*Zelamerus* Masner & Huggert 52
*Zelandonota* Masner & Huggert 51
*Zelostemma* Masner & Huggert 57

**Appendix. Species examined during this study, their tribal classification (after Masner 1976, 1980*b*; Galloway and Austin 1984; Masner and Huggert 1989) and the origin of the material** *, donated by L. Masner, CNCI; #, material from A. D. Austin, WARI; ^, species illustrated by line drawing; ", species illustrated by scanning electron microscopy

| Species | Origin |
|---|---|
| **SCELIONIDAE** | |
| **Scelioninae** | |
| **Nixoniini** | |
| *Nixonia* sp. 1^ | Sri Lanka* |
| *Nixonia* sp. 2" | Angola* |
| **Sparasionini** | |
| *Archaeoteleia mellea* Masner^" | Chile* |
| *Achaeoteleia* sp. | New Zealand* |
| *Sceliomorpha* sp. 1" | Trinidad* |
| *Sceliomorpha* sp. 2 | Costa Rica* |
| *Sceliomorpha* sp. 3 | Brazil* |
| *Sparasion* sp. 1 | N America* |
| *Sparasion* sp. 2^ | N America* |
| *Sparasion* sp. 3 | Malay Peninsula# |
| **Scelionini** | |
| *Acanthoscelio* sp. | Ecuador* |
| *Dicroscelio* sp. | Zimbabwe* |
| *Heptascelio* sp.^ | Sri Lanka* |
| *Oriescelio* sp. | Zimbabwe* |
| *Pseudoheptascelio* sp. | Ecuador* |
| *Scelio fulgidus* Crawford^ | Australia# |
| *Scelio parvicornis* Dodd | Australia |
| *Scelio* sp. 1 | Trinidad* |
| *Scelio* sp. 2 | N America* |
| *Scelio* sp. 3 | Ghana* |
| *Synoditella bisulcata* (Kieffer) | Jamaica* |
| **Baryconini** | |
| *Baryconus americanus* Ritchie & Masner | N America* |
| *Baryconus erythrothorax* (Kieffer) | N America* |
| *Baryconus* sp.^ | Ecuador* |
| *Bracalba* sp.^ | Australia# |
| *Chromoteleia* sp." | Brazil* |
| *Oxyscelio* sp.^ | Australia# |
| **Calliscelionini** | |
| *Anteromorpha* sp. 1 | Australia# |
| *Anteromorpha* sp. 2" | Zimbabwe* |
| *Anteromorpha* sp. 3 | New Caledonia# |
| *Calliscelio marlattii* (Ashmead) | N America* |
| *Calliscelio* sp. 1" | Malay Peninsula# |
| *Calliscelio* sp. 2^ | Zimbabwe* |
| *Calliscelio* sp. 3 | Australia# |
| *Calliscelio* sp. 4 | Malay Peninsula# |
| *Calliscelio* sp. 5 | Papua New Guinea# |
| *Crama* sp. | Australia* |
| *Dichoteleas* sp. | Malawi* |
| *Habroteleia* sp. | Sulawesi* |

| Species | Origin |
|---|---|
| **Calliscelionini** (continued) | |
| *Holoteleia* sp. | N America* |
| *Lispoteleia* sp. | Sulawesi# |
| *Macroteleia* sp. 1 | Australia# |
| *Macroteleia* sp. 2 | Zimbabwe* |
| *Oethecoctonus* sp. | Brazil* |
| *Palpoteleia* sp. 1^ | Mozambique* |
| *Palpoteleia* sp. 2 | Zimbabwe* |
| *Paridris laeviceps* (Ashmead) | N America* |
| *Paridris* sp. 1^ | Sarawak# |
| *Paridris* sp. 2" | Ivory Coast* |
| *Paridris* sp. 3 | Zimbabwe* |
| *Phoenoteleia* sp. | Indonesia* |
| *Probaryconus* sp. 1 | N America* |
| *Probaryconus* sp. 2 | Australia# |
| *Triteleia* sp. 1^ | Papua New Guinea# |
| *Triteleia* sp. 2 | Brazil* |
| **Psilanteridini** | |
| *Anteris bilineatus* Thomson^ | Bohemia* |
| *Anteris* sp. | N America* |
| *Calotelea* sp. 1 | India* |
| *Calotelea* sp. 2 | N America* |
| *Calotelea* sp. 3 | Trinidad* |
| *Duta* sp. 1 | N America* |
| *Duta* sp. 2 | Australia# |
| *Duta* sp. 3 | Sulawesi# |
| *Fusicornia* sp. 1 | Zimbabwe* |
| *Fusicornia* sp. 2 | Ivory Coast* |
| *Fusicornia* sp. 3^ | Nigeria* |
| *Genatropis* sp. | Australia* |
| *Harringtonia* sp. | N America* |
| *Leptoteleia marcelae* Masner | Brazil* |
| *Leptoteleia* sp.^ | India* |
| *Lidgbirdius terrestris* (Dodd) | Australia* |
| *Lidgbirdius* sp. | Australia* |
| *Mallateleia* sp. | Australia# |
| *Mecix texana* Masner | N America* |
| *Merriwa* sp.^ | Sarawak* |
| *Nyleta* sp. 1 | Australia# |
| *Nyleta* sp. 2 | Austral[illegible] |
| *Opisthacantha mellipes* Ashmead" | N America* |
| *Opisthacantha* sp. 1^ | N Ameri[illegible]* |
| *Opisthacantha* sp. 2 | India* |
| *Oxyteleia* sp. | Indonesia[illegible] |
| *Paraduta* sp. | Zimbabwe* |
| *Psilanteris* sp. 1^ | Zimb[illegible]e* |
| *Psilanteris* sp. 2 | New Ca[illegible]onia* |
| *Shreemana* sp. | S Africa[illegible] |
| *Spiniteleia campbelli* Masner" | N America* |
| *Styloteleia* sp. 1^ | Indonesia# |
| *Styloteleia* sp. 2" | New Caledonia* |
| *Thoronella* sp." | N America* |

**Appendix.** continued

| Species | Origin |
|---|---|
| **Aradophagini** | |
| *Aradophagus fasciatus* Ashmead^" | N America* |
| *A. sarotes* (Masner & Huggert) | Zimbabwe* |
| *Aradophagus* sp. | Australia* |
| **Neoscelionini** | |
| *Neoscelio agilis* Dodd^ | Australia# |
| *Neoscelio gloriosus* Dodd | Australia* |
| *Neoscelio pulchralis* Dodd | Australia# |
| **Cremastobaeini** | |
| *Cremastobaeus* sp. 1 | Trinidad* |
| *Cremastobaeus* sp. 2 | Columbia* |
| *Cremastobaeus* sp. 3^ | Australia# |
| **Mantibariini** | |
| *Mantibaria* sp. | Spain# |
| *Mantibaria* sp.^ | Congo* |
| **Platyscelionini** | |
| *Platyscelio* sp. 1 | Ivory Coast* |
| *Platyscelio* sp. 2^ | Sarawak# |
| **Doddiellini** | |
| *Doddiella nigriceps* Kieffer^ | Zimbabwe* |
| *Doddiella* sp. sp. 1 | Ivory Coast* |
| *Doddiella* sp. sp. 2 | Japan* |
| **Gryonini** | |
| *Breviscelio* sp. | Zimbabwe* |
| *Dyscritobaeus flavicornis* (Dodd) | Australia# |
| *Dyscritobaeus parvipennis* (Dodd) | Australia# |
| *Dyscritobaeus* sp. 1 | Australia# |
| *Dyscritobaeus* sp. 2 | Zimbabwe* |
| *Encrytoscelio mirissimus* Dodd | Australia# |
| *Encrytoscelio* sp. | Zimbabwe* |
| *Gryon reticulatus* (Dodd) | Australia# |
| *Gryon pentatomus* (Dodd)^ | Australia# |
| *Gryon rugulosum* (Fouts) | Papua New Guinea# |
| *Gryon variicorne* (Fouts) | Guyana* |
| *Gryon* sp. 1 | Australia# |
| *Gryon* sp. 2 | Zimbabwe* |
| *Neuroscelio doddi* Galloway, Austin, Masner^ | Australia# |
| **Embidobiini** | |
| *Echthrodesis lamorali* Masner | S Africa* |
| *Embidobia* sp.^ | Trinidad* |
| *Embioctonus* sp. | Brazil* |
| *Endecascelio* sp. | S Africa* |
| *Palaeogryon* sp. | N America* |
| **Thoronini** | |
| *Microthoron baeoides* Masner | Zimbabwe* |
| *Mic*[illegible]*thoron* sp. | Ivory Coast* |
| *Ps*[illegible]*nteris insignis* Fouts | N America* |
| [illegible] *metallicus* Halliday^ | N America* |
| [illegible]*odytes* sp.^[illegible] | N America* |
| **Baeini** | |
| *Baeus* sp. 1^ | Australia# |
| **Baeini** (continued) | |
| *Baeus* sp. 2 | Australia# |
| *Ceratobaeus clubionus* Austin | Australia# |
| *Ceratobaeus cuspicornutus* Austin | Australia# |
| *Ceratobaeus intrudae* Austin | Australia# |
| *Ceratobaeus lamponae* (Hickman) | Australia# |
| *Ceratobaeus masneri* Austin^ | Australia# |
| *Cyphacolus* sp. | Zimbabwe* |
| *Hickmanella holoplatysa* Austin | Australia# |
| *Hickmanella intrudens* (Hickman) | Australia# |
| *Idris helpidis* (Hickman) | Australia# |
| *Idris ixeutici* (Hickman) | Australia# |
| *Idris* sp. 1 | Australia# |
| *Idris* sp. 2 | Zimbabwe* |
| *Mirobaeoides setosus* Austin^ | Australia# |
| *Odontacolus* sp. | Australia# |
| **Unplaced Scelioninae** | |
| *Parascelio* sp.^ | Trinidad* |
| *Sceliacanthella* sp." | Australia* |
| **Teleasinae** | |
| **Teleasini** | |
| *Teleas neptuni* Sharkey^ | N America* |
| *Trimorus canadensis* (Ashmead)^ | N America* |
| *Trimorus caraborum* (Riley)^ | N America* |
| *Trimorus* sp. 1^ | Australia# |
| *Trimorus* sp. 2" | Australia# |
| **Xenomerini** | |
| *Gryonoides* sp. 1^ | Paraguay* |
| *Gryonoides* sp. 2 | Venezuela* |
| *Xenomerus* sp. 1^ | Australia# |
| *Xenomerus* sp. 2 | S Africa* |
| genus nov. sp. | Brazil* |
| **Telenominae** | |
| **Telenomini** | |
| *Eumicrosoma* sp. | Australia# |
| *Paratelenomus* sp. | Malaysia# |
| *Phanuropsis semiflaviventris* Girault | Trinidad# |
| *Psix lacunatus* Johnson & Masner^ | Australia# |
| *Telenomus podisi* Ashmead | N America* |
| *Telenomus* sp. 1^ | Australia# |
| *Telenomus* sp. 2 | N America* |
| *Trissolcus basalis* (Wollaston) | Australia# |
| *Trissolcus oenone* (Dodd)^ | Australia# |
| *Trissolcus* sp.1 | Zimbabwe* |
| *Trissolcus* sp.2 | Malaysia# |
| **PLATYGASTRIDAE** | |
| **Sceliotrachelinae** | |
| ***Fidiobia*-cluster** | |
| *Fidiobia* sp.^ | N America* |
| *Platygastoides* sp. 1^ | Australia# |
| *Platygastoides* sp. 2 | Australia# |
| *Zelandonota* sp. | New Zealand* |

**Appendix.** continued

| Species | Origin | Species | Origin |
|---|---|---|---|
| ***Aphanomerus*-cluster** | | ***Inostemma*-cluster** | |
| *Aphanomerella* sp.^ | Australia* | *Acerotella aceris* Masner^ | N America* |
| *Aphanomerus* sp. 1^ | Australia* | *Inostemma oculare* Austin^ | India# |
| *Aphanomerus* sp. 2 | Australia# | *Inostemma* sp. 1 | Australia# |
| *Austromerus* sp.^ | Australia# | *Inostemma* sp. 2 | Australia# |
| *Helava* sp. 1 | Ecuador* | *Iphitrachelus lar* Walker^ | N America* |
| *Helava* sp. 2 | Venezuela* | ***Allostemma*-cluster** | |
| *Parabaeus peckorum* Austin^ | Africa# | *Allostemma* sp.^ | Australia* |
| *Pseudaphanomerus hyalinatus* Szelényi | N America* | ***Platygaster*-cluster** | |
| *Tetrabaeus americanus* (Brues) | N America* | *Amblyaspis* sp.^ | Australia# |
| ***Isolia*-cluster** | | *Eritrissomerus* sp.^ | N America* |
| *Isolia* sp. 1^ | Palaearctic* | *Euxestonotus* sp. 1^ | Australia# |
| *Isolia* sp. 2 | Thailand* | *Euxestonotus* sp. 2 | N America* |
| ***Amitus*-cluster** | | *Isocybus canadensis* Provancher^ | N America* |
| *Amitus* sp.^ | N America* | *Metanopedias brunncipes* (Ashmead) | N America* |
| ***Allotropa*-cluster** | | *Metanopedias* sp.^ | Australia# |
| *Allotropa* sp. 1^ | Australia* | *Platygaster* sp. 1^ | N America* |
| *Allotropa* sp. 2 | Australia# | *Platygaster* sp. 2^ | N America* |
| *Errolium* sp. | New Zealand* | *Platygaster* sp. 3 | Australia# |
| **Platygastrinae** | | *Trichacis* sp.^ | N America* |
| ***Proplatygaster*-cluster** | | ***Synopeas*-cluster** | |
| *Almargella* sp.^ | Chile* | *Leptacis* sp. | Costa Rica* |
| *Magellanium* sp. | Chile* | *Piestopleura* sp. 1^ | Australia# |
| *Metaclisis* sp.^ | Mexico* | *Piestopleura* sp. 2 | N America* |
| *Proplatygaster* sp. 1^ | Australia* | *Synopeas procon* Austin | India# |
| *Proplatygaster* sp. 2 | Chile* | *Synopeas* sp. 1^ | N America* |
| ***Isostasius*-cluster** | | *Synopeas* (=*Sactogaster*) sp. 2^ | N America* |
| *Annettella* sp. | Australia* | *Synopeas* sp. 3 | Australia# |
| *Isostasius* sp. 1^ | Australia* | | |
| *Isostasius* sp. 2 | Australia# | | |

Manuscript received 28 November 1995; revised and accepted 12 April 1996
