# Supplementary material for: Spectral measure of color variation of black-orange-black (BOB) pattern in small parasitoid wasps (Hymenoptera: Scelionidae), a statistical approach
Source: PLoS One. 2019 Oct 24;14(10):e0218061. doi: 10.1371/journal.pone.0218061 (PMC6812806; doi:10.1371/journal.pone.0218061)
Supplement: Attachment — Submitted filename: Austin2005.pdf [file pone.0218061.s009.pdf]

Annu. Rev. Entomol. 2005. 50:553–82
doi: 10.1146/annurev.ento.50.071803.130500
Copyright © 2005 by Annual Reviews. All rights reserved
First published online as a Review in Advance on September 27, 2004

SYSTEMATICS, EVOLUTION, AND BIOLOGY OF SCELIONID AND PLATYGASTRID WASPS

A.D. Austin,[1] N.F. Johnson,[2] and M. Dowton[3]
[1]*Center for Evolutionary Biology and Biodiversity, School of Earth and Environmental Sciences, The University of Adelaide, S.A. 5005, Australia; email: andrew.austin@adelaide.edu.au*
[2]*Department of Entomology, The Ohio State University, Columbus, Ohio 43212; email: Johnson.2@osu.edu*
[3]*Institutes of Biomolecular Science and Conservation Biology, Department of Biology, Wollongong University, Wollongong, NSW 2522, Australia; email: mdowton@uow.edu.au*

Annu. Rev. Entomol. 2005.50:553-582. Downloaded from www.annualreviews.org
Access provided by CASA Institution Identity on 06/12/19. For personal use only.

Key Words host relationships, morphology, molecular systematics, Scelionidae, Platygastridae

■ **Abstract** The Platygastroidea comprises two families of parasitoids, Scelionidae and Platygastridae, and nearly 4500 described species. They parasitize a diverse array of insects as well as spiders. Idiobiont endoparasitism of eggs is the putative ground plan biology, as reflected by all scelionids, but most Platygastridae are koinobiont endoparasitoids of immature Auchenorrhyncha, Sternorrhyncha, and Cecidomyiidae. The superfamily is demonstrably monophyletic but its phylogenetic position remains uncertain. Relationships within the Platygastroidea are also poorly known and the group is in need of comprehensive phylogenetic study. Significant information is available on host relationships and biology, although much of this is biased to a few genera of Telenominae that are employed as biocontrol agents. Hosts for many genera are unknown, in particular those that inhabit leaf litter or parasitize solitary host eggs. The *Trissolcus basalis–Nezara viridula* parasitoid-host association has become a favored model system in ecological, behavioral, and physiological research on insects.

INTRODUCTION

The Hymenoptera is the most species-rich insect order and therefore the most diverse ordinal-level group of organisms (84), encompassing about 10% of all known species on our planet. This tremendous diversity is partitioned among three major groups: the aculeate wasps (including the stinging wasps, ants, and bees), the sawflies (which are mostly phytophagous as larvae), and a number of groups collectively referred to as the parasitic Hymenoptera. The Platygastroidea and the two families it includes, the Scelionidae and Platygastridae, is the third largest of the parasitic superfamilies after the Ichneumonoidea and Chalcidoidea and represents some 4460 described species worldwide. They are found in virtually all

Annu. Rev. Entomol. 2005.50:553-582. Downloaded from www.annualreviews.org
Access provided by CASA Institution Identity on 06/12/19. For personal use only.

habitats except for the polar regions and are particularly diverse and abundant in the wet forests of the tropics and subtropics. Platygastroids are small, ranging in size from 0.5 to 12 mm in body length, but most are less than 2.5 mm. The great majority of species are morphologically simplified compared with other parasitic Hymenoptera, a trend that is largely size dependent. Most have greatly reduced wing venation, have the antennae inserted close together just above the mouth, lack a prepectus behind the lateral pronotum (typical of the Chalcidoidea), and, compared with many other microwasps, are well sclerotized and often intricately sculptured (93, 96, 97).

All scelionid wasps are endoparasitoids of the eggs of insects and spiders, and this is probably the ground plan biology for the superfamily. Although egg endoparasitism occurs in numerous groups of parasitic Hymenoptera, few family-level taxa exclusively use eggs as hosts. The only other families known for which this biology is characteristic belong to the Chalcidoidea, i.e., the Trichogrammatidae and Mymaridae (see reviews: 58, 105), and possibly the Mymarommatidae, whose biology is unknown.

Female scelionids have a hypodermic-like ovipositor that they use to pierce the chorion of a host egg (5) and lay their own single egg or sometimes several eggs. The wasp larva that hatches consumes the contents of the host egg and pupates within it. A wide range of taxa serve as hosts: In addition to spiders, insect hosts include the Odonata, Orthoptera, Mantodea, Embiidina (= Embioptera), Hemiptera, Neuroptera, Coleoptera, Diptera, and Lepidoptera (13, 93) (Table 1). Like the Scelionidae, several basal lineages of Platygastridae are also endoparasitoids of eggs, largely of Coleoptera, but they also parasitize later stages of sessile hosts such as planthoppers, whiteflies, aphids, and mealybugs (Auchenorrhyncha and Sternorrhyncha) (Table 2). However, most platygastrid species attack gall flies (Diptera: Cecidomyiidae), ovipositing either in the egg or early larva of the host and completing their development in the larvae (100, 131).

Many hosts of platygastroids are pests of considerable importance in agriculture, forestry, and both human and animal health, e.g., the gypsy moth (*Lymantria dispar*), locusts (*Locusta migratoria*, *Choriocetes terminifera*), Hessian fly (*Mayetiola destructor*), the sunn pest (*Eurygaster integriceps*), southern green stink bug (*Nezara viridula*), kissing bugs (*Triatoma*, *Rhodnius*), and horse flies (*Tabanus* spp.). A number of species have been used as biological control agents with notable success (26, 29, 40, 109).

A review of the systematics, evolution, and biology of the Scelionidae and Platygastridae is timely given their use as natural enemies of pest species, but also because of their importance over the past 20 years in serving as model systems in entomological research. This has largely utilized the *Trissolcus basalis–N. viridula* parasitoid-host relationship and has included kairomone research (22, 102, 108, 127) and studies on sex ratio allocation (31, 107, 132), patch defense behavior (1, 42, 44, 45), competition (2), and more theoretical aspects of biological control (30).

Here we review the Platygastroidea, focusing mainly on their phylogeny, classification, and taxonomy, and areas related to these such as species diversity, ovipositional behavior, host relationships, and their potential as model systems

Annu. Rev. Entomol. 2005.50:553-582. Downloaded from www.annualreviews.org
Access provided by CASA Institution Identity on 06/12/19. For personal use only.

TABLE 1 Higher-level classifications proposed for the Scelionidae and associated host groups[a]

Kozlov 1970	Masner 1976	Austin & Field 1997	Host groups (based on type genera)
**Scelioninae**	**Scelioninae**	**Scelioninae**	
—	Sparasionini	Sparasionini	Tettigoniidae
Caloteleini	Nixonini	Nixonini	Tettigoniidae
Baryconini	Baryconini	Baryconini	Tettigoniidae
		**Scelionini s. l.**[b]	
Scelionini	Scelionini	Scelionini s. s.	Acrididae
Caloteleini	Calliscelionini	Calliscelionini	Gryllidae
Psilanteridini	Psilanteridini	Psilanteridini	Gryllidae
—	Aradophagini	Aradophagini	Unknown
—	—	Neoscelionini	Unknown
Platyscelionini	Platyscelionini	Platyscelionini	?Tettigoniidae
—	Doddiellini	Doddiellini	Unknown
—	Cremastobaeini	Cremastobaeini	?Gryllidae
Mantibarini	Mantibarini	Mantibarini	Mantodea
Gryonini	Gryonini	Gryonini	Various Heteroptera, Lepidoptera
Embidobiini	Embidobiini	Embidobiini	Embiidina
Pseudanteridini	—	—	NA
**Baeini**	—	—	
Thoronini	Thoronini	Thoronini	Nepidae
Baeini	Baeini	Baeini s. l.	Araneae
Idrini	Idrini	—	
**Teleasinae**	**Teleasinae**	**Teleasinae**	
Teleasini	Teleasini	Teleasini	Coleoptera
Xenomerini	Xenomerini	Xenomerini	Coleoptera
**Telenominae**	**Telenominae**	**Telenominae**	
Telenomini	Telenomini	Telenomini	Heteroptera, Auchenorrhyncha, Lepidoptera, Neuroptera, Diptera
Aradophagini	—	—	
Tiphodytini	—	—	Gerridae

[a]The table is best read from right to left in that some tribes proposed by Kozlov (81) are no longer recognized or employ different names.

[b]The Scelionini s. l. contains the nominal tribe Scelionini s. s. plus Calliscelionini, Psilanteridini, Aradophagini, Neoscelionini, Platyscelionini, and Doddiellini. (The position of *Thoronella* within the Scelionini s. l. is uncertain but is the only scelionid known to parasitize eggs of Odonata.)

TABLE 2 Higher-level classifications proposed for the Platygastridae and associated host groups[a]

Kozlov 1970	Masner & Huggert 1989	Austin & Field 1997	Host groups (based on type genera)
**Sceliotrachelinae**	**Sceliotrachelinae**	**Sceliotrachelinae**	
Sceliotrachelini	—	—	
Fidobiini	*Fidobia*-cluster	*Fidiobia*-cluster	Coleoptera
Aphanomerini (I)[b]	*Aphanomerus*-cluster	*Aphanomerus*-cluster	Fulgoroidea
—	*Isolia*-cluster	*Isolia*-cluster	Unknown
—	*Amitus*-cluster	*Amitus*-cluster	Aleyrodidae
Allotropini (I)	*Allotropa*-cluster	*Allotropa*-cluster	Pseudococcidae
**Platygastrinae**	**Platygastrinae**	**Platygastrinae**	
Metaclisiini (I)	—	—	
—	*Proplatygaster*-cluster	*Proplatygaster*-cluster	Cecidomyiidae
Inostemmatini (I)	*Isostasius*-cluster	*Isostasius*-cluster	Cecidomyiidae
Inostemmatini (I)	*Inostemma*-cluster	*Inostemma*-cluster	Cecidomyiidae
—	*Allostemma*-cluster	*Allostemma*-cluster	Unknown
Platygastrini	—	*Platygaster*-cluster	Cecidomyiidae
Synopeadini	—	*Synopeus*-cluster	Cecidomyiidae
Coelopeltini	—	—	

[a]The table is best read from right to left in that the classifications by Austin & Field (13) and Masner & Huggert (100) do not recognize the Inostemmatinae sensu Kozlov (81) and previous authors.

[b]I, Inostemmatinae.

Annu. Rev. Entomol. 2005.50:553-582. Downloaded from www.annualreviews.org
Access provided by CASA Institution Identity on 06/12/19. For personal use only.

in areas other than those mentioned above. We have not dealt with their ecology, behavior, and application in biological control, simply because the literature in these areas is too extensive and is better treated separately at a future date.

RELATIONSHIPS AND MONOPHYLY OF THE SUPERFAMILY

Morphological Studies

Prior to the 1970s the Scelionidae and Platygastridae were variously treated within the Hymenoptera, but they were mostly placed within the superfamily Proctotrupoidea, a taxon that has no unifying characters and has served to accommodate a heterogeneous assemblage of apocritan families whose relationships are largely unknown (49, 96, 106). During this time, little information was forthcoming on the relationships of the two families with other Hymenoptera, other than generalized statements or classifications that pointed to a close relationship between them. An exception to this was Masner (86), who first proposed that Scelionidae and Platygastridae be classified as a superfamily separate from the Proctotrupoidea.

Annu. Rev. Entomol. 2005.50:553-582. Downloaded from www.annualreviews.org
Access provided by CASA Institution Identity on 06/12/19. For personal use only.

This concept has gradually become more refined (13, 96, 100) and is now widely accepted by most hymenopteran systematists.

The 1970s heralded a period of invigorated interest in relationships among the Hymenoptera, and these were underpinned by detailed character discussion and, later on, quantitative analysis employing the implicit methods of phylogenetic systematics. The first significant studies were by Königsmann (76–78), who based his work mostly on characters extracted from the literature. Although his phylogeny for the Hymenoptera showed little structure at the superfamily/family level, he did postulate a closer relationship between Scelionidae+Platygastridae and Diapriidae. A similar set of relationships between these three families had been previously proposed by Kozlov (81), but with Platygastridae said to be a "derivative" of Diapriidae because of their common host relationships, i.e., parasitoids of Diptera. Rasnitsyn (115) presented an almost fully resolved phylogeny for the Hymenoptera that included fossil taxa and detailed discussion of characters supporting each node in the tree. This hypothesis also postulated a link between Platygastridae and Diapriidae, an unlikely association between Scelionidae and the chalcidoid family Mymaridae (51), and placed these families into an expanded Diaprioidea along with the Monomachidae and Austroniidae. Later, Rasnitsyn (116) presented a more refined version of his earlier concepts in English that took into account a number of detailed morphological studies including internal structures (50, 51, 101). This later tree postulated a sister-group relationship of Scelionidae+Platygastridae with the Chalcidoidea (including Mymaridae) and Mymarommatidae, with this clade being sister to a partly unresolved but paraphyletic Proctotrupoidea. Rasnitsyn's (115, 116) work was not cladistically based and his trees were generated intuitively.

What followed in the intervening years were a number of detailed investigations into a range of internal and external character systems, many of which have included character scoring for the Platygastroidea (15–17, 51, 52, 69, 111, 113, 136). Particularly relevant is the comparative study of Gibson (52), who argued against a relationship between Platygastroidea and Chalcidoidea+Mymarommatoidea (36, 116). Rather, he postulated a monophyletic group comprising Platygastroidea and three proctotrupoid families (Pelecinidae, Proctotrupidae, and Vanhorniidae), on the basis of their possession of an annular pronotum and a mesopleural-mesotrochanteral muscle. Although these studies have identified a number of potentially informative character systems, none have yet been incorporated into rigorous broad-scale phylogenetic analyses of apocritan relationships that might better resolve the position of the Platygastroidea relative to other apocritan superfamilies. Whitfield (134, 135) synthesized much of the available morphological information into consensus trees to examine patterns of host utilization in parasitic Hymenoptera, but again, the phylogenies he used were not cladistic and largely reiterated Rasnitsyn (116) in supporting a Platygastroidea+(Chalcidoidea+Mymarommatoidea) relationship.

Two of the most informative studies undertaken to date have reanalyzed Rasnitsyn's (116) data using parsimony-based cladistic methods. In doing so, Ronquist

Annu. Rev. Entomol. 2005.50:553-582. Downloaded from www.annualreviews.org
Access provided by CASA Institution Identity on 06/12/19. For personal use only.

et al. (118) reinterpreted some characters and revealed a substantially different set of relationships compared with those predicted in Rasnitsyn's intuitive tree. Importantly, the Ceraphronoidea, placed with the Evanioidea, Stephanidae, Megalyridae, and Trigonalidae by Rasnitsyn (116), were resolved as sister to the Platygastroidea, with Chalcidoidea sister to these two. Recently, Sharkey & Roy (121) have highlighted a number of shortcomings with Rasnitsyn's original data and the Ronquist et al. (118) reanalysis (e.g., that many characters are not cladistic in nature and all have been coded for hypothetical ground plan taxa, not exemplars) and shown that much of the structure in the Ronquist et al. tree is due to reductional characters associated with the wings. Sharkey & Roy (121) revised all wing characters and showed that the resulting parsimony tree was largely comb-like, but with similar relationships for the Platygastroidea (i.e., Chalcidoidea+Mymarommatidae as the sister to an unresolved trichotomy, Scelionidae+Platygastridae+Ceraphronoidea).

Molecular Studies

The only molecular work published to date relevant to the Platygastroidea is a series of sequential sequencing-based studies that have attempted to resolve relationships among the Apocrita (25, 33, 36). These have gradually increased the number of taxa sampled and the number of genes sequenced. The most recent and comprehensive of these (35) sequenced the 16S, 28S, and COI genes for 84 apocritan taxa and employed different models of parsimony analysis. In all cases but one, the Platygastroidea were resolved as the sister group to the Chalcidoidea, and the Ceraphronoidea were always remote from this clade. When the morphological matrix of Ronquist et al. (118) was combined with the molecular data, this relationship was disrupted in 50% of the analyses, with the position of the Platygastroidea being otherwise unresolved or sister to the Cynipoidea or Ceraphronoidea. However, the morphological data are probably overly influential in these combined analyses given that the Ronquist et al. (118) matrix is coded at the family level; these codings were necessarily applied to each family exemplar represented in the Dowton & Austin (35) molecular matrix, rather than each character being scored separately for each species.

Mitochondrial gene rearrangements also have the potential to recover phylogeny (38), and a number of these that may delimit monophyletic groups within the Platygastroidea have been identified. For example, the region between the cytochrome oxidase II (cox2) and ATPase8 (a8) genes normally contains two tRNA genes, one for lysine (K) and another for aspartate (D). Examination of this genome region across a range of hymenopterans and other insects firmly indicates that the ground plan organization for the Hymenoptera is cox2-K-D-a8 (Figure 1*A*) (34). However, no platygastroids that retain this organization have yet been found. The Scelionidae so far assessed have a reversal of the tRNA genes (i.e., cox2-D-K-a8; Figure 1*B*), although one species (of the genus *Macroteleia*) is missing the K gene (i.e., cox2-D-a8; Figure 1*C*). Presumably, this has moved elsewhere in the genome and may represent a character for assessing relationships within the Calliscelionini.

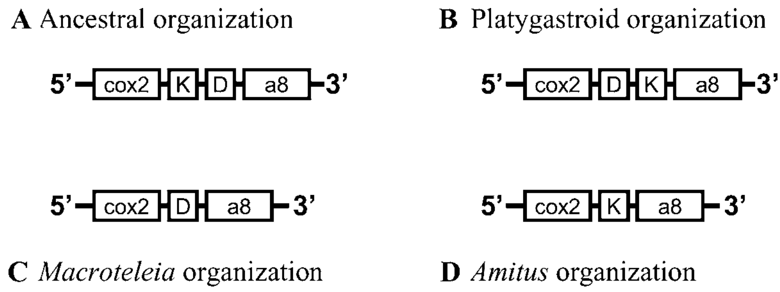

Figure 1 Mitochondrial gene region between the cytochrome oxidase II (cox2) and ATPase8 (a8) genes showing: (*a*) the ancestral organization for the two tRNA genes, lysine (K) and aspartate (D), found in many Hymenoptera. (*b*) The arrangement in several genera of Scelionidae in which the position of K and D is reversed. (*c*) The arrangement in *Macroteleia* (Scelionidae) in which the K gene is translocated to another part of the mitochondrial genome. (*d*) The arrangement in *Amitus* (Platygastridae) in which the D gene is translocated to another part of the mitochondrial genome.

Annu. Rev. Entomol. 2005.50:553-582. Downloaded from www.annualreviews.org
Access provided by CASA Institution Identity on 06/12/19. For personal use only.

Only a single member of the Platygastridae has so far been assessed (*Amitus*), and this has a distinct (derived) arrangement—here the D gene is missing (cox2-K-a8; Figure 1*D*). Broader surveys of this gene region thus have the potential to identify a number of monophyletic groups within the Platygastroidea. Moreover, if such a survey confirmed that the hymenopteran ancestral organization is absent from the superfamily, there may be a gene rearrangement synapomorphy to assess potential platygastroid sister groups. Here, investigating a range of basal Chalcidoidea might help support or refute other molecular evidence that these two superfamilies are sister groups (35).

Similarly, the mitochondrial region between the NADH dehydrogenase 3 (nad3) and NADH dehydrogenase 5 (nad5) genes is rearranged among certain members of the Platygastroidea (39). The ground plan organization for the Hymenoptera is inferred as nad3-A-R-N-S-E-F-nad5, with six tRNA genes (denoted by single letters) normally between the two protein coding genes (nad3 and nad5). This mitochondrial region has been sequenced in just two platygastroids, both from the Scelionidae. *Trissolcus* retains the ancestral organization, whereas *Ceratobaeus* has nad3-A-N-F-S-E-R-nad5. A broader survey of this region should thus identify a monophyletic group among the scelionids.

In summary, the relationships of the Platygastroidea are not clear, with analyses based on molecular data supporting a sister group relationship with Chalcidoidea, and available morphological data indicating a relationship with the Ceraphronoidea. To date, the limited information that has been analyzed cladistically does not support a clade comprising Platygastroidea+Pelecinidae+Proctotrupidae +Vanhorniidae sensu Gibson (52). Answering the question of what is the extant sister group to the Platygastroidea is largely synonymous with the larger problem

of defining monophyletic families within the Apocrita and resolving a robust phylogeny among them. Given the ease with which molecular data can now be generated, the wealth of morphological information available, and a move toward setting up international collaborative groups to tackle various aspects of the "Tree of Life," the task of determining the exact relationships of the Platygastroidea should be easily achievable within a relatively short period (12).

Monophyly of Platygastroidea

Although sister group relationships of the Platygastroidea are not yet finalized, the monophyly of the superfamily is undisputed. The evidence supporting this comes primarily from two character systems: the modified structure of the abdomen in relation to ovipositor extension and retraction, and the unique sensilla of the female antenna. The dorsal and ventral plates of the abdominal segments of platygastroids are locked together (via laterotergites) and the spiracles of the tracheal system are reduced and nonfunctional. Together, these characters convert the abdomen into a pumping organ used to change the hydrostatic pressure of the hemolymph to assist with the extension and retraction of the ovipositor, which, at rest, is withdrawn back inside the abdomen within an elongate membranous tube (13, 47, 96, 100). This arrangement has been postulated as an adaptation for protecting the delicate hypodermic-like ovipositor when not used to pierce the chorion of host eggs, and it has several unique features paralleled at a gross level only by the telescopic ovipositor system in the aculeate family Chrysididae (114). In platygastroids this system is complex and has resulted in the proximal elements of the ovipositor (i.e., gonocoxae and gonopophyses) being disassociated from the posterior segments of the abdomen, unlike most other parasitic Hymenoptera in which the ovipositor and sheaths often protrude from the posterior abdomen (114, 130). Furthermore, two functionally different arrangements occur within the superfamily. In the first (termed the *Ceratobaeus* type), ovipositor extension is achieved by contraction of muscles attached to elongated apodemes assisted by a change in hydrostatic pressure (5), whereas in the second and putatively more apomorphic arrangement (termed the *Scelio* type), no such muscles are present and the ovipositor is extended at the end of a telescopic membranous tube, homologous to intersegmental membrane, by hydrostatic pressure alone (13, 43). The platygastroid ovipositor system is best treated as a functionally related suite of independent characters that includes the arrangement of abdominal sternal and tergal plates, abdominal musculature, abdominal spiracles, apodemes associated with the posterior segments and associated musculature, development of an elongate membranous tube to house the ovipositor, and disassociation of a number of sclerotized elements that are normally closely apposed in other Hymenoptera (13).

The Platygastroidea also possess a second character that is unique in the Hymenoptera. The apical segments of the female antenna are enlarged in size and form a clava comprising three to seven segments, as occur in other microwasps. However, on the underside of each of these segments, amid the numerous tactile

Annu. Rev. Entomol. 2005.50:553-582. Downloaded from www.annualreviews.org
Access provided by CASA Institution Identity on 06/12/19. For personal use only.

Annu. Rev. Entomol. 2005.50:553-582. Downloaded from www.annualreviews.org
Access provided by CASA Institution Identity on 06/12/19. For personal use only.

sensilla, are one or two unique sensilla, termed "multiporous gustatory sensilla," that have now been shown to be involved in the recognition of host-associated cues (20, 66, 66a).

PHYLOGENY AND CLASSIFICATION

Classification Systems

The development of a workable classification system for the Platygastroidea began in earnest at the end of the nineteenth century with the publication of Ashmead's (3) influential work on the Nearctic fauna. He recognized two subfamilies, Platygastrinae and Scelioninae, within a single family, Proctotrupidae, and further divided these two groups into two and four tribes, respectively: Inostemmatini and Platygastrini (Platygastrinae), and Scelionini, Teleasini, Baeini, and Telenomini (Scelioninae). These taxa were arbitrarily raised in rank some years later (4), with Platygastridae having two subfamilies and Scelionidae four subfamilies. Brues (23) erected an additional subfamily, Sceliotrachelinae, within the Scelionidae but this was later transferred to the Platygastridae (75) without recognition of its previous higher rank. Monographing the known world fauna, Kieffer (75) modified Ashmead's scheme by reducing Platygastridae to a fifth subfamily with the Scelionidae. Subsequent authors during the following decades mostly followed one or other of these two schemes invoking only minor changes. More substantial changes were proposed by (*a*) Szabó (128), who divided the Platygastridae into four tribes, Inostemmatini, Platygastrini, Iphitrachelini, and Amitini (the latter subsequently synonymized with Sceliotrachelini; 88); (*b*) Szabó (129), who proposed an additional subfamily, the Gryoninae, for the Scelionidae; and (*c*) Hellén (56), who put forward a somewhat anomalous classification for the Scelionidae that recognized three subfamilies, Scelioninae, Telenominae, and Platygastrinae. Not one of these changes was incorporated into more contemporary classifications.

Although for much of the twentieth century platygastroid classification provided a reasonable framework for the description of new taxa, a number of genera were misplaced at the family level owing to morphological convergences associated with the structure of the antenna or general body form. For example, the consolidation of the apical segments of the antenna into a shortened, fused clava has occurred independently in several platygastrid genera (e.g., *Aphanomerus*, *Parabaeus*, and *Tetrabaeus*) and numerous members of the Scelionidae (particularly the Thoronini and Baeini; 9, 93). This led to numerous authors erroneously treating these and other platygastrids as members of the Scelionidae (75, 81). In the case of *Parabaeus*, the convergence in body form associated with the loss of wings so closely matches the unusual structure of *Baeus* (compare Figure 2*A* with 2*B*) that this genus was incorrectly placed in the Scelionidae for more than 60 years (11).

As one might expect, homoplasies in a range of morphological characters have led to a number of genera being putatively misplaced at subfamily and tribal levels.

Annu. Rev. Entomol. 2005.50:553-582. Downloaded from www.annualreviews.org
Access provided by CASA Institution Identity on 06/12/19. For personal use only.

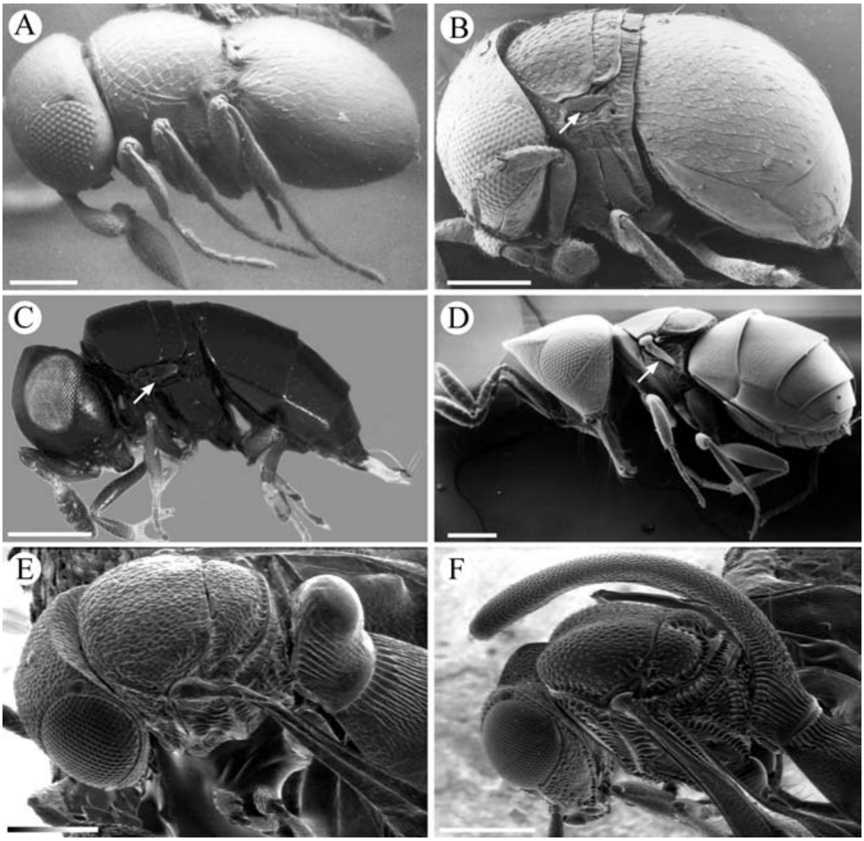

Figure 2 Female Platygastroidea that show substantial modification to the body associated with loss or reduction of wings and living in crytobiotic niches. In all cases the metasoma is broadly abutted against the mesosoma, which has its posterior surface flattened vertically. (*a*) *Parabaeus peckorum* (Platygastridae: Sceliotrachelinae: *Aphanomerus*-cluster) is completely apterous (from Reference 11 with permission of CSIRO Publishing). (*b*) *Baeus* sp. (Scelionidae: Scelioninae: Baeini) is micropterous with wings reduced to tiny sclerites (*arrowed*) (A.D. Austin, unpublished). (*c*) *Platyscelidris fossorius* (Scelionidae: Scelioninae: Scelionini sensu lato) is micropterous (*arrowed*) (from Reference 74 with permission of the Entomological Society of Washington). (*d*) *Encyrtoscelio mirissimus* (Scelionidae: Scelioninae: Gryonini) is micropterous (*arrowed*) (from Reference 25 with permission of CSIRO Publishing). (*e*) *Ceratobaeus haqi* (Scelionidae: Baeini) has a short metasomal horn (from Reference 63 with permission of the South Australian Museum). (*f*) *Ceratobaeus longicornutus* (Scelionidae: Baeini) has an extremely elongate metasomal horn (from Reference 63 with permission of the South Australian Museum). Scale bars $= 100\ \mu m$.

For example, the scelionine genera *Aradophagus* and *Tiphodytes*, which lack a submarginal ridge on the abdominal sternites and therefore have free laterotergites as in the Telenominae (48, 92, 93, 99), were misplaced in this subfamily for several decades (81, 82). However, the most striking impact of a homoplasious character on the classification of the superfamily is associated with the independent loss of the single forewing vein in many Platygastridae. For nearly 90 years, following the publication of Ashmead's work (3, 4), the subfamily classification was largely based on the presence (Inostemmatinae) or absence (Platygastrinae) of a submarginal (R) vein. However, a detailed comparative study of a large proportion of the extant fauna by Masner & Huggert (100) postulated that this vein has been independently reduced or lost multiple times and that it may be present or absent even within the same genus (e.g., *Fidiobia*). The consequences of this finding are profound for developing a workable classification for the family and for understanding how the group has evolved.

The current higher-level classification for the Platygastroidea is partly based on the work of Kozlov (81), who proposed a radical, though somewhat mechanistic, tribal classification for the two families. A large part of his scheme for the Scelionidae has been adopted in subsequent studies (13, 46, 93), but with a number of significant modifications. These include incorporating the Baeinae into the Scelioninae, first proposed by Masner (89), and the reorganization and repositioning of several tribes, chiefly Aradophagini, Tiphodytini, Caloteleini, and Pseudanteridini (= Psilanteridini in part) (Table 1). However, the subfamily and tribal arrangement for the Platygastridae proposed by Kozlov (81) has now been completely superseded by the reclassification of Masner & Huggert (100) through their reinterpretation of available morphological data. The impact of this scheme is shown in Table 2, in which taxa previously assigned to the tribes of Inostemmatinae sensu Kozlov (81) and other simpler schemes (104) are now distributed across the two newly redefined subfamilies. Below this level, Masner & Huggert (100) refrained from erecting a formal tribal classification because of the difficulty in assessing relationships among genera and instead proposed a series of informal generic groups (termed clusters). Since then, the only contribution to the higher classification of the Platygastridae has been by Austin & Field (13), who added two additional groups, the *Platygaster*- and *Synopeas*-clusters, to cover the genera not treated by Masner & Huggert (100).

Phylogeny

Surprisingly, the Platygastroidea have not been exposed to any comprehensive phylogenetic analysis aimed at resolving higher-level relationships. This contrasts with the detailed morphological and molecular phylogenetic studies that have been undertaken on the other large parasitoid superfamilies Ichneumonoidea (18, 19, 112), Chalcidoidea (27), and Cynipoidea (117). However, numerous studies have generated explicit phylogenetic questions, or such questions can be directly inferred from the current higher classifications (Tables 1 and 2). Probably most important is whether the two families are monophyletic. In this respect, the Platygastridae is

Annu. Rev. Entomol. 2005.50:553-582. Downloaded from www.annualreviews.org
Access provided by CASA Institution Identity on 06/12/19. For personal use only.

putatively a well-supported group, albeit mostly on the basis of a series of character reductions, i.e., abdominal tergite 9 desclerotized; reduced or lost posterior abdominal sensory structures (cerci); reduced venation, antennal segments, and mouthpart palps; and metapleuron with dense mat of hairs (13, 100). In comparison, the Scelionidae are not defined by any unique synapomorphy, and the family is likely to be paraphyletic (13, 49, 100). There is now some evidence to support this proposal, although the data are limited. A cladistic analysis of 13 characters aimed at investigating character transformation in the ovipositor system postulated that the scelionid genus *Sparasion* was the sister group to a monophyletic Platygastridae (13), whereas in a multi-gene phylogenetic study of parasitoid families (35), the Scelionidae was never monophyletic and often placed *Sparasion* as the sister-group to the nine remaining exemplars used for the superfamily.

Relationships at lower taxonomic levels are also problematic, although the current classification for the Scelionidae provides a useful starting point. Where earlier classifications contained largely unsubstantiated statements about subfamily or tribal relationships (e.g., tribe A is closest to tribe B), studies over the past 30 years have tended to include more precise phylogenetic inferences as cladistic methodology gained acceptance (93). A recent assessment of the phylogenetic status of currently recognized higher-level groups (13) concluded that the largest subfamily, Scelioninae, is defined only by symplesiomorphies and is probably paraphyletic, while the Teleasinae, Telenominae, and many tribes probably represent natural assemblages, although many are not convincingly supported on morphological grounds.

Of all characters examined in recent years, the ovipositor system has been the most useful for inferring putative relationships. The telescopic ovipositor tube of the *Scelio*-type system putatively supports a clade comprising seven tribes and approximately 55 genera (13), and this is probably functionally linked to a single lineage, the members of which all parasitize orthopteran eggs in hidden locations. This group has tentatively been called the Scelionini sensu lato to identify it within the current classification (Table 1). However, the monophyly of this group still needs to be examined as part of a broader phylogenetic analysis to show more definitively that this character system has not evolved independently and does indeed support a single lineage within the superfamily.

Little work has been undertaken on the phylogeny of individual tribes. A sister-group relationship has been postulated between the Embidobiini and Baeini (46), with host searching for silk (embiids versus spiders) being proposed as a possible link between these tribes. The tribe Thoronini, the members of which are all thought to parasitize eggs in aquatic habitats (Hemiptera and Odonata), contains genera with both types of ovipositor. Thus its monophyly is highly suspect, and the smooth body and long stiff bristles of its members may be convergent adaptations linked to underwater behavior (73). A similar putative adaptation also occurs in the unrelated embidobine genus *Echthrodesis*, which has a pilose surface and parasitizes the eggs of spiders in marine tidal pools in South Africa (90). Further, the position and relationships of several primitive genera such as *Nixonia*,

Annu. Rev. Entomol. 2005.50:553-582. Downloaded from www.annualreviews.org
Access provided by CASA Institution Identity on 06/12/19. For personal use only.

Annu. Rev. Entomol. 2005.50:553-582. Downloaded from www.annualreviews.org
Access provided by CASA Institution Identity on 06/12/19. For personal use only.

Archaeoteleia, and *Neuroscelio* (47, 87, 91) is clouded by their retention of putative plesiomorphic characters (e.g., larger number of antennal and palpal segments, more complete wing venation, elongate and uniformly segmented abdomen) that are common in fossil taxa. At the generic level, there have been few cladistic studies on any Platygastroidea; those of relationships among genera of Baeini (64), Australian species of *Scelio* (32), species of the telenomine genus *Psix* (72), and Australian species of *Trissolcus* (70) are among the few. In addition to these studies, differences among character states associated with the ovipositor system and associated structures have led to questioning the monophyly of numerous large genera (e.g., *Anteris*, *Ceratobaeus*, *Idris*, *Opisthacantha*, *Telenomus*, *Trimorus*, and *Platygaster*) (13, 63).

Where a framework at least exists for testing specific higher-level hypotheses for the Scelionidae, solid information on relationships within the Platygastridae is virtually absent. As discussed above, the presence or absence of a submarginal vein in the forewing has been the long-term basis of an artificial subfamily classification for this family. However, the reassignment of genera into two newly constituted subfamilies (Sceliotrachelinae and Platygastrinae) by Masner & Huggert (100), following the most exhaustive morphological study yet undertaken, has still not shed much light on the evolution of the group. Of the two subfamilies, the Platygastrinae is possibly monophyletic, but this proposal is supported more on having a common host group, the Cecidomyiidae, than on any recognizable morphological synapomorphies. However, the Sceliotrachelinae is probably paraphyletic, as indicated by its lack of defining features, more diverse array of hosts, and a larger proportion of genera restricted to a single biogeographic region, particularly to the southern continents (100). Of the nine informal generic groups recognized by Masner & Huggert (100) and the additional two proposed by Austin & Field (13) (Table 2), none are demonstrably monophyletic and most are defined on reductional synapomorphies or similarities in body form, antennal structure, or wing characteristics. However, Masner & Huggert (100) have usefully coded a matrix for 43 morphological characters for approximately three quarters of the valid platygastrid genera, and this will provide substantial input into any future cladistic analysis of family or superfamily relationships.

The current lack of a robust phylogenetic scheme for the Platygastroidea is a major drawback for current systematic and biological studies in that it prevents any meaningful assessment of relationships within the group and constrains our ability to track character transformations and evolution, host relationships, and other biological traits. On the basis of the evidence to date, the level of reductional homoplasy compromises the utility of morphological characters in developing a robust hypothesis of phylogeny. DNA sequence data from multiple genes will undoubtedly be valuable, in conjunction with morphological data, in resolving this question. In the absence of other influential characters, the effect of reductional characters has been aptly demonstrated for one tribe, the Baeini, in which the removal of wing reduction states caused the complete collapse of an otherwise well-resolved tree (64).

Annu. Rev. Entomol. 2005.50:553-582. Downloaded from www.annualreviews.org
Access provided by CASA Institution Identity on 06/12/19. For personal use only.

Fossil Taxa

The oldest fossils of Platygastroidea reported in the literature date to the early Cretaceous (115) and are identified only as Scelionidae. According to Grimaldi et al. (54), scelionids typically outnumber all other Hymenoptera inclusions in amber from Myanmar from the Middle Cretaceous, a statement consistent with Siberian Cretaceous amber, in which Scelionidae comprise 37% of all wasps (139). Although numerous fossils are available, only three species have been described: *Cenomanoscelio pulcher*, *Proteroscelio antennalis*, and *Baryconus fulleri*. *Baryconus* is an extant genus, with 69 recognized species; however, this generic assignment has never been confirmed. The other two genera are known only from the single specimens upon which the original descriptions were based. Grimaldi et al. (54) assert that the greatest diversity of scelionids is found in the Mesozoic and that the family has dwindled in diversity during the Cenozoic. This claim has not been substantiated and is possibly incorrect given the extreme diversity that is being uncovered for the extant fauna (see below).

Records of Scelionidae from Cenozoic deposits, particularly amber, are more numerous. Thirty-five species in 27 genera have been described (24, 28). Some of these are from relatively recent copal and belong to extant genera, sometimes apparently even to living species. However, much of the taxonomy has not been reconciled with modern concepts. Species from Baltic amber are the most abundant (26 species). Although some of these inclusions have been referred to modern genera, perhaps correctly so, the relative abundance of subfamilies differs strikingly from the extant fauna. The Platygastridae are represented by only a single species from Baltic amber, referred to the sceliotracheline genus *Parabaeus*, and two compression fossils of Oligocene age are assigned to the collective name *Platygasterites*. The Telenominae, with 837 extant valid species, are represented by only a single species. Teleasinae, with 480 known living species, have not been described from fossil material. No platygastroids from Cenozoic amber deposits other than those from the Baltic and Chiapas have yet been described.

SPECIES DIVERSITY AND TAXONOMY

Species Diversity

At present there are 3308 valid species of Scelionidae and 1153 species of Platygastridae (60), with only 15 genera recorded with more than 50 described species: i.e., *Telenomus* (612 spp.), *Platygaster* (419 spp.), *Trimorus* (389 spp.), *Gryon* (273 spp.), *Scelio* (246 spp.), *Trissolcus* (170 spp.), *Synopeas* (166 spp.), *Ceratobaeus* (161 spp.), *Idris* (145 spp.), *Sparasion* (140 spp.), *Macroteleia* (128 spp.), *Leptacis* (124 spp.), *Inostemma* (82 spp.), *Baryconus* (69 spp.), and *Calliscelio* (60 spp.). Although in some cases species richness of individual genera is biased by a single large study for a particular region (63, 83), the number of diverse genera based on described species is moderately low given that there are 244 genera recognized globally. However, as for many other groups of parasitoids, these figures probably

Annu. Rev. Entomol. 2005.50:553-582. Downloaded from www.annualreviews.org
Access provided by CASA Institution Identity on 06/12/19. For personal use only.

bear little relationship to the magnitude of their true size, although several recent studies do provide an indication of the relative scale of platygastroid diversity. The fauna of tropical regions and the southern continents remains largely unstudied and, as indicated by studies on terrestrial arthropods in Australia, comprehensive taxonomic revisions often lead to a 5- to 10-fold increase in species (138). Recent long-term assessment of the platygastroid fauna of Costa Rica (97) has led to estimates of the country's Platygastroidea between 1650 and 3300 species, representing an unrealistic 37% to 74% of the described world fauna. At a much finer geographic scale, long-term evaluation of parasitoid diversity associated with litter and soil at one site using emergence traps in southern Australia yielded 75 species of Scelionidae and 40 species of Platygastridae from just 5 m^2 of ground (C. Stephens, personal communication), representing nearly 20% of the current described Australian fauna. Furthermore, pitfall trapping over multiple seasons has shown that the ground fauna of baeine scelionids overlaps by 55% to 80% among fragmented reserves (separated by tens of kilometers), but at a continental level (separated by hundreds to thousands of kilometers) there is virtually no overlap in species composition (62), which indicates a high level of regional endemism.

More extensive collection and study of platygastroids from a worldwide perspective have revealed a surprising number of tramp species. *Psix tunetanus*, a parasitoid of pentatomoids found from northern Africa to India, was apparently introduced twice into the New World: in the southwestern United States and near Lake Maracaibo, Venezuela (72). It is an aggressive species that has now spread as far east as Illinois. *Trissolcus basalis* was described initially from Madeira and is widespread throughout the Old World. It was discovered no later than 1894 in the Caribbean but has no close relatives in the Americas. Similarly, *Palpoteleia atra*, originally described from the Seychelles, is widespread throughout the Old World, from West Africa to Korea, Japan, and Australia, exhibiting little or no morphological variation. *Aradophagus fasciatus*, found in both North America and Europe, seems to be a synanthropic species, although its host has yet to be determined.

Morphological Trends

Platygastroids display a number of morphological trends that, although not unique within the group, are more prevalent than those displayed in other parasitoids and have been important taxonomically as well as a source of confusion. Three characteristics are discussed: the development of a metasomatic horn, wing reduction, and venation reduction. The metasomatic (tergal) horn is formed from the dorsal expansion of the first metasomatic tergite into a large hump or tubular horn-like structure. It is possibly specific to the Platygastroidea, occurs in numerous unrelated genera of both Scelionidae and Platygastridae, and, importantly, acts as a recess for the internally retracted ovipositor. This characteristic is best developed in the genera *Ceratobaeus* (Scelionidae) (63) and *Baryconus* (Scelionidae) and in some species of *Inostemma* (Platygastridae) (100). These genera show a great range in form, particularly *Ceratobaeus*, from species with a short horn

(Figure 2*E*) to species in which the tubular horn bends over the dorsal mesosoma almost touching the head (Figure 2*F*). The length of the horn is correlated with the length of the ovipositor and presumably represents an adaptation to protect the hypodermic-like ovipositor at rest, but horn length also allows for a more elongate ovipositor that can reach and parasitize protected or hidden hosts (63, 100). For *Ceratobaeus* these are the eggs of spiders contained within a silken egg sac of various thickness (8), while *Baryconus* parasitizes the eggs of Tettigoniidae (Orthoptera), and *Inostemma* parasitizes the eggs and/or larvae of Cecidomyiidae within plant tissue.

Wing reduction is prevalent in both families and is most common in the female sex. Although wings are said to be absent in females of several genera (46), this is rarely the case. These females are in fact micropterous; their wings are reduced to tiny sclerotized flaps (Figure 2*B–D*). The Old World species of *Parabaeus* are the only known members of the Platygastroidea that lack tegulae and are completely wingless (Figure 2*A*). Numerous genera that contain mostly fully winged species also have species with micropterous or brachypterous females (see Reference 65 for definition), for example, *Idris*, *Ceratobaeus*, *Probaryconus*, *Gryon*, *Dyscritobaeus*, *Trimorus*, *Platygastoides*, *Fidiobia*, *Inostemma*, and *Metaclisis.* Although in most cases it is thought that wing reduction is fixed and a good species-level taxonomic character, in at least some *Idris* (59) and possibly some *Ceratobaeus* and *Inostemma*, wing size is polymorphic. In nearly all cases of wing reduction, females are associated with litter or soil, and this is assumed to be an adaptation for living in and searching for hosts in this confined habitat (46). In virtually all species for which the sexes are accurately associated and females are micropterous or brachypterous, the males are fully winged. Because of this, different selection pressures are postulated to operate on the sexes, with males being short-lived and spending most time searching for mates (10). Wing reduction is also common for species living on islands and at high altitudes, and although they also inhabit litter, the major difference, compared with their continental sister taxa, is that the males are also micropterous (6, 10). For several genera, microptery in females is also associated with extreme modification to the shape of the body that results in a "flea-like" appearance, sometimes involving fusion of sclerites (Figure 2*B*). Again, this has been postulated as an adaptation for litter-inhabiting taxa, but in *Baeus* and *Mirobaeoides* it may also provide a streamlined form for penetrating the silk egg sacs of their spider hosts (8, 10).

Compared with other Hymenoptera, most scelionids have their forewing venation reduced to at most only two tubular veins; one is along or near the fore margin (Sc+R) and the other is situated obliquely to this (r), so that the pattern is similar to that of many Chalcidoidea. The scelionid hindwing is usually represented only by a single tubular vein (Sc+R). The Platygastridae have at most only a foreshortened section of Sc+R in the forewing and sometimes in the hindwing, with many taxa lacking these elements so that the wings are completely veinless. However, several taxa across both families have venation that is substantially more complete than these general patterns, although the additional veins are not tubular

Annu. Rev. Entomol. 2005.50:553-582. Downloaded from www.annualreviews.org
Access provided by CASA Institution Identity on 06/12/19. For personal use only.

and are represented only by pigmented lines (spectral veins). These were properly documented first for the Gondwanan genus *Archaeoteleia* (91), which has a number of distal veins in the forewing and more complete basal hindwing venation, and then for *Neuroscelio* (47). However, similar venational patterns also occur in some species of *Scelio* (32) and to a slightly lesser degree in the forewing of some Platygastridae, such as *Metaclisis*, *Proplatygaster*, and *Platygastoides* (100). These more complete venational patterns are similar to those of the fossil genera *Brachyscelio* (32) and *Archaeoscelio* (91) and other undescribed amber fossils, and these patterns can be used to trace the pathway of wing vein reduction to those taxa in the superfamily that are completely veinless.

Annu. Rev. Entomol. 2005.50:553-582. Downloaded from www.annualreviews.org
Access provided by CASA Institution Identity on 06/12/19. For personal use only.

Taxonomic Resources

The world species of Platygastroidea were most recently cataloged by Johnson (71) and Vlug (131), in which the taxonomic literature since 1758 is summarized. The last modern taxonomic treatment of the world fauna of Scelionidae is found in Masner (93). The literature for the Platygastridae is scattered among numerous regional treatments, and no comprehensive keys to genera have been attempted in over 75 years. Major systematic publications have included regional works for Australia (46), the Soviet Union (79, 82, 83), and the Holarctic region (94) and numerous revisions of the major genera (32, 59, 63, 67a, 80, 95, 98, 103, 120).

The primary natural history institutions around the world have moderate to good regional collections, e.g., the Australian National Insect Collection (Canberra), the Zoological Institute (St. Petersburg, Russia), and the Ukrainian Academy of Sciences (Kiev, Russia). The major European and North American collections (e.g., the National Museum of Natural History in Washington, DC, and the Muséum d'Histoire Naturelle in Paris) house a good deal of the type material. The types of Australian species are concentrated in the South Australian Museum (Adelaide). The Natural History Museum in London has a significant number of types as well as a wide sampling of specimens from around the world. However, the preeminent collection of platygastroids is that of the Canadian National Collection of Insects in Ottawa, which surpasses many other major collections combined for the depth and breadth of material it contains from all biogeographic regions.

Web-based information sources are quickly becoming available and, because of the capacity for rapid updates, can be more current than traditional publications. The existing cyber-infrastructure for platygastroid systematics is among the most thorough for any group of organisms. Information on the fauna of Australia is available through the Australian Faunal Directory (14). The database maintained at Ohio State University is accessible through three interrelated portals, the Hymenoptera Name Server (60), Hymenoptera On-Line (61), and The Genera of Scelionidae (Hymenoptera: Platygastroidea) of the World (119). These provide information on nomenclature, distribution, images, biology, and identification, as well as links to other electronic resources.

Annu. Rev. Entomol. 2005.50:553-582. Downloaded from www.annualreviews.org
Access provided by CASA Institution Identity on 06/12/19. For personal use only.

HOST RELATIONSHIPS

Platygastroids utilize a wide spectrum of hosts (Tables 1 and 2). In addition, they also show a clear pattern of host group specificity that is possibly only matched by the Braconidae in the parasitic Hymenoptera (133). This is said to occur when a higher-level taxon, a tribe or subfamily, is associated with a single host group. For example, the Scelionini are known only to parasitize eggs of acridid grasshoppers, Gryonini parasitize the eggs of various Heteroptera (and rarely from Lepidoptera), Mantibarini parasitize the eggs of Mantodea, Baeini parasitize the eggs of Araneae, and Teleasinae parasitize the eggs of Coleoptera. Within the Platygastridae the various generic clusters show similar group specificity, whereas the Platygastrinae are known only to parasitize Cecidomyiidae (Table 2). Furthermore, the Scelionini (sensu lato), defined by having a telescopic ovipositor system, are apparently restricted to the eggs of various Orthoptera (although other genera of Scelionidae also parasitize orthopteran eggs, e.g., *Sparasion*, *Nixonia*). Telenominae show the widest host group associations, but most species attack heteropteran and lepidopteran eggs, with relatively few species exploiting the eggs of Neuroptera, Auchenorrhynca, and Diptera (67a).

A number of interesting questions arise from this spectrum of host associations. For example, is Orthoptera the plesiomorphic host group for the superfamily (96), given that it is the most commonly utilized order across the Scelioninae and is the host group of the putative basal genera *Sparasion* and *Nixonia*? Among the Telenominae, has the evolutionary transition in host utilization been from the eggs of hemimetabolous insects to those of holometabolous insects? Has the switch to parasitizing spider eggs occurred independently in the Embidobiini and Baeini? Where did the switch to coleopteran eggs occur (i.e., what is the sister group to the Teleasinae)? Currently it is impossible to even begin to answer these major questions in platygastroid evolution because of the absence of any phylogenetic scheme for higher-level taxa.

The other limitation to understanding how host associations have evolved across the superfamily is a lack of detailed and corroborated host data for many groups. Hosts for most genera and some tribes are unknown (13), and for the Teleasinae, a subfamily containing 480 described species, the number of known hosts is limited to just a handful of species reared from the eggs of Carabidae. An assumption that this is the only host group for the subfamily may be incorrect given the paucity of data, and the Teleasinae could just as easily parasitize a great range of coleopteran families. The situation for the Teleasinae also highlights a more general problem in that there is a substantial bias in host information across the superfamily, particularly against hosts associated with cryptobiotic habitats such as rotting wood, litter, and soil, in which host eggs are difficult to locate. Also there is a bias against hosts that lay their eggs singly as opposed to egg masses in which at least a couple of emerging hosts may allow identification of the eggs. As a consequence, numerous platygastroid genera, and indeed other parasitic Hymenoptera, that are commonly encountered in these habitats with mass-collecting devices have no

Annu. Rev. Entomol. 2005.50:553-582. Downloaded from www.annualreviews.org
Access provided by CASA Institution Identity on 06/12/19. For personal use only.

available host data. Alternatively, hosts that lay their eggs in more accessible places, or represent economically or ecologically important groups such as Heteroptera, Orthoptera, and Lepidoptera, have more comprehensive host information.

Platygastroids show a relatively high degree of host specificity, with many associated with just a single host species. Others appear to be restricted to a single host genus or host family; instances of broad host ranges (across an order of insects in the case of one species of *Telenomus*; 67a) are apparently rare. In some cases, parasitizing a large number of host species from across a family (such as for *Trissolcus basalis* and *T. euschisti* for numerous species of Pentatomidae; 68) may occur when the parasitoid involved searches for egg masses of similar appearance in the same habitat. This example, however, reveals a weakness in data on host relationships. Most host records for *T. basalis* document it as a parasitoid of *Nezara*, principally *N. viridula*. However, the wasp has been both accidentally and intentionally introduced into locations around the world where its host is found. The apparent broad host range for this species is probably inaccurate; the species is more likely oligophagous, but under unnatural conditions or when normally suitable hosts cannot be found, it may accept and successfully reproduce in the eggs of other species. In contrast, numerous records (67) document *Trissolcus euschisti* as a parasitoid of a wide variety of stink bug hosts. Recent summaries of host data for platygastroid genera (13), scelionid tribes (46, 93, 104), platygastrid genera (100, 131), Baeini (8, 63), and Scelionini s. s. (32) are available.

BIOLOGY

The biology of several large genera of Scelionidae have been reviewed in detail as part of contemporary taxonomic studies [*Telenomus* (67a), *Ceratobaeus* (63), and *Scelio* (32)], and these include many aspects that relate more generally to other members of the family. In addition, several texts on Hymenoptera provide useful overviews of platygastroid biology (49, 53, 96, 97, 110), although most available information is for the Scelionidae and, by comparison, little is known for the Platygastridae. The most detailed biological studies have been undertaken on scelionids that are used as or have potential as biological control agents, for example, *Trissolcus*, *Telenomus*, and *Scelio*. As a consequence, information is strongly biased toward the Telenominae, and care should be taken in extrapolating from these taxa to other members of the Scelionidae that are associated with different hosts.

Host Finding

Host finding and acceptance in the Telenominae, and possibly the Gryonini, appears to be largely influenced by airborne and surface kairomones on the eggs (102, 127), although sex pheromones from the adult host or host plant volatiles may also be possibilities for some scelionids, as has been demonstrated for some *Trichogramma* (129a). Members of some Baeini appear to search first for the bark of trees and

Annu. Rev. Entomol. 2005.50:553-582. Downloaded from www.annualreviews.org
Access provided by CASA Institution Identity on 06/12/19. For personal use only.

then for the silk of their spider host nests under bark (7). Silk recognition is possibly chemically mediated, given that at least two species discriminate between the silk nests of host and nonhost spiders (7). The mechanism of host finding for scelionids that parasitize host eggs in soil such as *Scelio*, *Sparasion*, many Teleasinae, and Sceliotrachelinae is unknown and may be mediated by chemical residues from host oviposition or changes in the texture of the soil surface. Probably most unusual are the scelionid genera associated with aquatic hosts, such as species of *Tiphodytes* that parasitize gerrid eggs often below the surface of ponds (122), and *Echthrodesis* that parasitizes spider eggs in intertidal pools (90). In both cases, the hairs over the surface of the body may act as a plastron or, in the case of *Echthrodesis,* it may use the air cavity of its host underwater. Again, nothing is known of how these species locate their hosts. Phoresy occurs in a few genera, but it is not common among the Scelionidae and is unknown in the Platygastridae. It has been recorded for species of *Sceliocerdo* and *Synoditella* associated with acridid hosts; *Mantibaria* on mantids; *Thoronella* on Odonata; *Epigryon*, *Protelenomus*, and *Telenomus* associated with Heteroptera; and *Telenomus* on Lepidoptera (32, 97). Phoresy represents one adaptation for accessing eggs before they become unsuitable as hosts.

Oviposition, Development, and Emergence

Once the site of host eggs is recognized, the process of oviposition appears to be similar for most species; however, preovipositional behavior is strongly influenced by the immediate habitat or conditions around the host eggs. For example, *Scelio* and *Sparasion* burrow headfirst through soil to reach the egg mass, reverse their position so that the head points upward, and oviposit into a number of eggs (32, 55). Baeines either oviposit through the silk wall of spider egg sacs or burrow through the wall to access the eggs directly (8); various telenomines and *Gryon* oviposit into exposed heteropteran eggs by positioning themselves on the dorsal surface of the egg mass and oviposit downward, or they stand beside the egg mass, facing away from it, and orient themselves to oviposit posteriorly in line with the body (41, 137).

Scelionids are virtually all solitary idiobiont primary parasitoids, and reports that diverge from this biology are rare and often unsubstantiated. However, there are now numerous, confirmed records of gregarious parasitism: e.g., *Telenomus monilicornis* (*T. sphingis auctorum*) in large sphingid eggs and two species attacking large reduviid eggs (N.F. Johnson, unpublished data). Many species exploit hosts that lay clumped batches of eggs, such as spiders, Heteroptera, Orthoptera, Mantodea, and Coleoptera, and adaptations to prevent superparasitism are common. These fall mostly into two categories: the first involves marking host eggs either externally or internally, and the second involves female wasps defending egg batches from competitors (41, 45). External or internal chemical marks are applied via the ovipositor (41, 137) and are secretions from the abdominal accessory glands (123). External marks are recognized by sensilla on the antenna and

Annu. Rev. Entomol. 2005.50:553-582. Downloaded from www.annualreviews.org
Access provided by CASA Institution Identity on 06/12/19. For personal use only.

are most commonly applied to exposed host egg batches such as those of Heteroptera and Lepidoptera (67a), and internal marks are probably more common and largely detected by ovipositor sensilla. In both cases they result in females avoiding ovipositing into eggs marked by themselves or other females, although for externally marked eggs, naïve females are more prone to superparasitize hosts (67a). External marks are reported to be of short duration, whereas internal marks are more persistent but do not operate immediately (97, 123). Host eggs are not suitable for all of their embryonic period, and successful oviposition occurs mostly in the early stages. This is either because the chorion (e.g., exposed heteropteran eggs) or froth around the eggs (e.g., Acrididae, Mantodea) becomes too hard for the ovipositor to penetrate (32, 41), or because of developmental changes within the host egg, as may occur in spider eggs (7) and possibly other hosts. Some *Telenomus* species that parasitize lepidopteran eggs inject venom that halts host development and causes disruption of the embryonic tissues (126), and teratocytes from the scelionid egg cause necrosis of host tissues and also destroy eggs of other parasitoids (123).

Parasitized host eggs change color at some stage during development, usually becoming much darker than unparasitized eggs. Once the parasitoid has completed its development, it chews its way through the host egg chorion and then spends a substantial period preening wings, antennae, and legs. Most scelionids that parasitize clumped eggs show a strongly biased female sex ratio (7, 41, 67a) and have males that emerge first and mate with their siblings. This has led to the use of scelionids, particularly *Trissolcus*, to examine more theoretical aspects of sex allocation strategies and male-male competition (53, 110, 124, 132). Several species of *Telenomus* have males that guard eggs containing unemerged wasps and fight to exclude other males from the egg mass. At least two species, *Telenomus polyrmorphus* and *T. sulculus*, have polymorphic males.

Given their taxonomic diversity, the biology of Platygastridae is poorly known, but the information available indicates that it is different from that of the Scelionidae. Most biological information comes from a few studies on Holarctic species of *Platygaster* and *Inostemma* (Platygastrinae) that are solitary koinobionts in cecidomyiid hosts. These taxa oviposit into eggs or newly emerged larvae, but development is delayed until the host larva is almost fully grown. In at least some cases the parasitoid develops preferentially in particular host tissues, such as brain, nerve cord, and gut. Few species are gregarious within their host, and polyembryony has been confirmed for only one species, *Platygaster zosine* (85, 97, 125), a parasitoid of the Hessian fly. At least some species of *Platygaster* have an unusual form of development that involves a series of nuclear divisions and the development of a syncytical trophamnion that surrounds the embryo and has a surface of microvilli for the absorption of nutrients from the host (57). Furthermore, after parasitoid eclosion, the trophamnion may disintegrate into a number of "pseudogerms," some of which are thought to have a teratocyte function, and after further growth others act as a food source for the developing parasitoid (see References 49 and 97 for reviews).

The biology of the Sceliotrachelinae is even less known than that of the Platygastrinae. The genera that parasitize coleopteran eggs and Fulgoroidea are solitary idiobiont endoparasitoids and are thought to develop in a way similar to that of scelionids (97), whereas others are solitary koinobionts in immobile Coccoidea and Aleyrodidae. Most appear to be solitary, but at least one species of *Allotropa* is gregarious in a mealybug host.

PROSPECTS

The Platygastroidea are diverse, both taxonomically and biologically, and are ideal as a representative parasitoid group for many areas of research. Their application in biological control (26, 29, 40, 109) has provided a solid body of information for several genera that has been the basis of their use as models in kairomone research and in studies on sex ratio allocation, patch defense behavior, and competition (see above). However, they also have significant potential as model systems in other areas of entomology, in particular, for assessing parasitoid diversity, in studying host-endoparasitoid interactions at a physiological and molecular level, and in understanding the evolution of parasitoid-host relationships and mitochondrial genomes.

Although our taxonomic knowledge of the superfamily is still naïve, this is largely the case for other major groups of parasitoids. However, as targets for biodiversity research, especially of grassland and litter faunas, platygastroids have a number of useful attributes: (*a*) They are readily trapped in mass-collecting devices; (*b*) they are more heavily sclerotized than most other groups of microwasps and therefore do not disintegrate if left in traps for prolonged periods; (*c*) many, if not most, genera are readily identifiable; (*d*) the species of many genera (particularly from the Scelioninae, Teleasinae, and Sceliotrachelinae) can be separated into morphospecies with a reasonable level of reliability; and (*e*) host associations are moderately well-known at the tribal level such that quantitative inferences can be made about the diversity of platygastroids and their corresponding host groups (e.g., diversity of baeine species in leaf litter versus the diversity of spider species in the same habitat).

Up to the 1980s, endoparasitism of eggs was considered a passive process in that the parasitoid larva simply hatched and fed on the surrounding yolk and embryo, pupated, and emerged as an adult wasp. However, several reports for members of the Telenominae indicate that this process is not so straightforward and that the parasitoid proactively neutralizes the host with venoms and via teratocyte activity (123, 126). This points to a more dynamic relationship at a physiological and possibly molecular level for what are otherwise anatomically simple hosts that lack a cellular immune system. This is an area worthy of more detailed research, and future investigations could profitably examine how widespread is the use of venoms across the superfamily and what is their mode of action.

The apparent hierarchical nature of platygastroid host relationships makes them an ideal group in which to study host switching and the possible biological attributes

Annu. Rev. Entomol. 2005.50:553-582. Downloaded from www.annualreviews.org
Access provided by CASA Institution Identity on 06/12/19. For personal use only.

that may have been involved in each case. This requires a solid phylogenetic hypothesis for the group, which is currently lacking. Development of a robust set of higher-level relationships should be a priority for platygastroid systematists: Only then will it be possible to answer fundamental questions as to which groups are monophyletic, what are the limits to their host relationships, and at what point on the phylogeny has host switching occurred.

Recent preliminary work has shown that gene rearrangements are widespread within the mitochondrial genome of the Platygastroidea, and that they are likely to be useful as phylogenetic markers (37, 38). As the number of taxa sequenced for the K-D and A-R-N-S-E-F tRNA regions increases, these data will provide a useful and independent adjunct to more traditional DNA sequence information for resolving deep-level phylogenetic relationships. However, they are also likely to serve as a useful model for investigating the nature of mitochondrial gene rearrangements per se, given that the few taxa sequenced to date show both simple (nearest-neighbor exchanges; Figure 2*B*) and more complex (translocations; Figure 2*C*, *D*) gene rearrangements. These complex gene movements cannot be easily explained by the duplication/random loss model invoked for vertebrates, and more sophisticated mechanisms need to be considered and explored (38).

Annu. Rev. Entomol. 2005.50:553-582. Downloaded from www.annualreviews.org
Access provided by CASA Institution Identity on 06/12/19. For personal use only.

ACKNOWLEDGMENTS

Research on the Platygastroidea by ADA has been generously supported by the Australian Research Council and the Australian Biological Resources Study, and for NFJ by the National Science Foundation. We are grateful to John Jennings and Jim Whitfield for commenting on drafts of the manuscript, and to Nick Stevens for assisting with references and figures.

The *Annual Review of Entomology* is online at http://ento.annualreviews.org

LITERATURE CITED

1. Amarasekare P. 2000. Spatial dynamics in a host-multiparasitoid community. *J. Anim. Ecol.* 69:201–13
2. Amarasekare P. 2000. Coexistence of competing parasitoids on a patchily distributed host: local vs. spatial mechanisms. *Ecology* 81:1286–96
3. Ashmead WH. 1893. A monograph of the North American Proctotrypidae. *Bull. U.S. Nat. Mus.* 45:1–472
4. Ashmead WH. 1903. Classification of the pointed-tail wasps of the superfamily Proctotrypoidea. *J. NY Entomol. Soc.* 11:86–99
5. Austin AD. 1983. Morphology and mechanics of the ovipositor system of *Ceratobaeus* Ashmead (Hymenoptera: Scelionidae) and related genera. *Int. J. Insect Morphol. Embryol.* 12:139–55
6. Austin AD. 1984. A new genus of apterous scelionid from Lord Howe Island (Hymenoptera: Scelionidae). *Syst. Entomol.* 9:121–25

Annu. Rev. Entomol. 2005.50:553-582. Downloaded from www.annualreviews.org
Access provided by CASA Institution Identity on 06/12/19. For personal use only.

7. Austin AD. 1984. The fecundity, development and host relationship of *Ceratobaeus* spp. (Hymenoptera: Scelionidae), parasites of spider eggs. *Ecol. Entomol.* 9:125–38
8. Austin AD. 1985. The function of spider egg sacs in relation to parasitoids and predators, with special reference to the Australian fauna. *J. Nat. Hist.* 19:359–76
9. Austin AD. 1986. A taxonomic revision of the genus *Mirobaeoides* Dodd (Hymenoptera: Scelionidae). *Aust. J. Zool.* 34:315–37
10. Austin AD. 1988. A new genus of baeine wasp (Hymenoptera: Scelionidae) from New Zealand associated with moss. *NZ J. Zool.* 15:173–83
11. Austin AD. 1990. A revision of old world *Parabaeus* Kieffer (Hymenoptera: Platygastridae), a group of highly modified apterous parasitoids inhabiting litter. *Invert. Taxon.* 3:647–59
11a. Austin AD, Dowton M, eds. 2000. *The Hymenoptera: Evolution, Biodiversity and Biological Control.* Melbourne: CSIRO
12. Austin AD, Dowton M, Deans A. 2004. The pattern of relationships among superfamilies of apocritan Hymenoptera derived from recent morphological and molecular analyses. *Proc. 1st Dresden Meet. Insect Phylogeny Entomol. Abh.* 61:160–61
13. Austin AD, Field SA. 1997. The ovipositor system of scelionid and platygastrid wasps (Hymenoptera: Platygastroidea): comparative morphology and phylogenetic implications. *Invert. Taxon.* 11:1–87
14. Aust. Faunal Dir.: Checklist for Platygastroidea. 2004. http://www.deh.gov.au/biodiversity/abrs/online-resources/abif/fauna/index.html
15. Basibuyuk HH, Quicke DLJ. 1999. Grooming behaviours in the Hymenoptera (Insecta): potential phylogenetic significance. *Zool. J. Linn. Soc.* 125:349–82
16. Basibuyuk HH, Quicke DLJ. 1999. Gross morphology of multiporous plate sensilla in the Hymenoptera (Insecta). *Zool. Scr.* 28:51–67
17. Basibuyuk HH, Quicke DLJ, Rasnitsyn AP, Fitton MG. 2000. Morphology and sensilla of the orbicula, a sclerite between the tarsal claws, in the Hymenoptera. *Ann. Entomol. Soc. Am.* 93:625–36
18. Belshaw R, Dowton M, Quicke DLJ, Austin AD. 2000. Estimating ancestral geographic distributions: a Gondwanan origin for the aphid parasitoids. *Proc. R. Soc. London Ser. B* 267:491–96
19. Belshaw R, Fitton M, Herniou E, Gimeno C, Quicke DLJ. 1998. A phylogenetic reconstruction of the Ichneumonoidea (Hymenoptera) based on the D2 variable region of 28S ribosomal RNA. *Syst. Entomol.* 23:109–23
20. Bin F. 1981. Definition of female antennal clava based on its plate sensilla in Hymenoptera Scelionidae Telenominae. *Redia* 64:245–61
21. Deleted in proof
22. Bin F, Vinson SB, Strand MR, Colazza S, Jones WA Jr. 1993. Source of an egg kairomone for *Trissolcus basalis*, a parasitoid of *Nezara viridula*. *Physiol. Entomol.* 18:7–15
23. Brues CT. 1908. Scelionidae. *Genera Insectorum* 80:13
24. Brues CT. 1940. Fossil parasitic Hymenoptera of the family Scelionidae in Baltic amber. *Proc. Am. Acad. Arts Sci.* 74:69–90
25. Caleca V, Bin F. 1995. World revision of the genus *Encytoscelio* Dodd (Hymenoptera: Scelionidae). *Invert. Taxon.* 9:1021–45
26. Caltagirone LE. 1981. Landmark examples in classical biological control. *Annu. Rev. Entomol.* 26:213–32
27. Campbell B, Heraty J, Rasplus J-Y, Chan K, Steffen-Campbell J, et al. 2000. Molecular systematics of the

Annu. Rev. Entomol. 2005.50:553-582. Downloaded from www.annualreviews.org
Access provided by CASA Institution Identity on 06/12/19. For personal use only.

Chalcidoidea using 28S-D2 rDNA. See Ref. 11a, pp. 54–73

28. Carpenter F. 1992. *Treatise on Invertebrate Paleontology, Part R. Arthropoda,* Vol. 4. *Superclass Hexapoda.* Boulder, CO: The Geol. Soc. Am. 655 pp.
29. Clarke AR. 1990. The control of *Nezara viridula* L. with introduced egg parasitoids in Australia. A review of a 'landmark' example of classical biological control. *Aust. J. Agric. Res.* 41:1127–46
30. Clarke AR, Walter GH. 1995. "Strains" and the classical biological control of insect pests. *Can. J. Zool.* 73:1777–90
31. Colazza S, Wajnberg E. 1998. Effects of host egg mass size on sex ratio and oviposition sequence of *Trissolcus basalis* (Hymenoptera: Scelionidae). *Environ. Entomol.* 27:329–36
32. Dangerfield P, Austin A, Baker G. 2001. *Biology, Ecology and Systematics of Australian Scelio (Hymenoptera: Scelionidae).* Melbourne: CSIRO
33. Dowton M, Austin AD. 1994. Molecular phylogeny of the insect order Hymenoptera: apocritan relationships. *Proc. Natl. Acad. Sci. USA* 91:9911–15
34. Dowton M, Austin AD. 1999. Evolutionary dynamics of a gene rearrangement 'hotspot' in the hymenoptera mitochondrial genome. *Mol. Biol. Evol.* 16:298–309
35. Dowton M, Austin AD. 2001. Simultaneous analysis of 16S, 28S, COI and morphology in the Hymenoptera: Apocrita—evolutionary transitions among parasitic wasps. *Biol. J. Linn. Soc.* 74:87–111
36. Dowton M, Austin AD, Dillon N, Bartowsky E. 1997. Molecular phylogeny of the apocritan wasps with particular reference to the Proctotrupomorpha and Evaniomorpha. *Syst. Entomol.* 22:245–55
37. Dowton M, Belshaw R, Austin AD, Quicke DLJ. 2002. Simultaneous molecular and morphological analysis of braconid relationships (Insecta: Hymenoptera: Braconidae) indicates independent mt-tRNA gene inversions within a single wasp family. *J. Mol. Evol.* 54:210–26
38. Dowton M, Castro LR, Austin AD. 2002. Mitochondrial gene rearrangements as phylogenetic characters in the invertebrates: the examination of genome 'morphology'. *Invertebr. Syst.* 16:345–56
39. Dowton M, Castro LR, Campbell SL, Bargon SD, Austin AD. 2003. Frequent mitochondrial gene rearrangments at the hymenopteran nad3-nad5 junction. *J. Mol. Evol.* 56:517–26
40. Drooz AT, Bustillo AE, Fedde GF, Fedd VH. 1977. North American egg parasite successfully controls a different host genus in South America. *Science* 197:390–91
41. Eberhard WG. 1975. The ecology and behavior of a subsocial pentatomid bug and two scelionid wasps: strategy and counter strategy in a host and its parasites. *Smithson. Contrib. Zool.* 205:1–39
42. Field SA. 1998. Patch exploitation, patch-leaving and pre-emptive patch defence in the parasitoid wasp *Trissolcus basalis* (Insecta: Scelionidae). *Ethology* 104:323–38
43. Field SA, Austin AD. 1994. Anatomy and mechanics of the telescopic ovipositor system of *Scelio* Latreille (Hymenoptera: Scelionidae) and related genera. *Int. J. Insect Morphol. Embryol.* 23:135–58
44. Field SA, Calbert G. 1998. Patch defence in the parasitoid wasp *Trissolcus basalis*: when to begin fighting? *Behaviour* 135:629–42
45. Field SA, Calbert G. 1999. Don't count your eggs before they're parasitized: contest resolution and the trade-offs during patch defense in a parasitoid wasp. *Behav. Ecol.* 10:122–27
46. Galloway ID, Austin AD. 1984. Revision of the Scelioninae (Hymenoptera: Scelionidae) in Australia. *Aust. J. Zool. Suppl. Ser.* 99:1–1138

Annu. Rev. Entomol. 2005.50:553-582. Downloaded from www.annualreviews.org
Access provided by CASA Institution Identity on 06/12/19. For personal use only.

47. Galloway ID, Austin AD, Masner L. 1992. A revision of the enigmatic genus *Neuroscelio* Dodd, a group of primitive scelionids from Australia (Hymenoptera: Scelionidae), with a discussion of the ovipositor system of the tribe Gryonini. *Invertebr. Taxon.* 6:523–45
48. Garcia JL, Masner L. 1994. A redefinition of *Aradophagus* (Hymenoptera: Scelionidae), with a key to described species. *Can. Entomol.* 126:67–74
49. Gauld ID, Bolton B, eds. 1996. *The Hymenoptera*. Oxford: Oxford Univ. Press/Br. Mus. Nat. Hist. 2nd ed.
50. Gibson GAP. 1985. Some pro- and mesothoracic characters important for phylogenetic analysis of Hymenoptera, with a review of terms used for structures. *Can. Entomol.* 117:1395–443
51. Gibson GAP. 1986. Evidence for monophyly and relationships of Chalcidoidea, Mymaridae, and Mymarommatidae (Hymenoptera: Terebrantes). *Can. Entomol.* 118:205–40
52. Gibson GAP. 1999. Sister group relationships of the Platygastroidea and Chalcidoidea (Hymenoptera): an alternate hypothesis to Rasnitsyn 1988. *Zool. Scr.* 28:125–38
53. Godfray HCJ. 1994. *Parasitoids: Behaviour and Evolutionary Ecology*. Princeton, NJ: Princeton Univ. Press
54. Grimaldi DA, Engel MS, Nascimbene PC. 2002. Fossiliferous Cretaceous amber from Myanmar (Burma): its rediscovery, biotic diversity, and paleontological significance. *Am. Mus. Novit.* 3361:1–71
55. Grissell EE. 1997. Biological notes on *Sparasion* Latreille (Hymenoptera: Scelionidae), an egg parasitoid of *Atlanticus gibbosus* Scudder (Orthoptera: Tettigoniidae). *Proc. Entomol. Soc. Wash.* 99:693–96
56. Hellén W. 1971. Die Scelioninen Finnlands (Hymenoptera: Proctotrupoidea). *Fauna Fenn.* 18:1–68
57. Hill CC, Emery WT. 1937. The biology of *Platygaster herrickii*, a parasite of the Hessian fly. *J. Agric. Res.* 55:199–213
58. Huber JT. 1986. Systematics, biology and hosts of the Myrmaridae and Mymarommatidae (Insecta: Hymenoptera): 1758–1984. *Entomography* 4:185–243
59. Huggert L. 1979. Revision of the west Palaearctic species of the genus *Idris* Foerster, s.l. (Hymenoptera: Proctotrupoidea: Scelionidae). *Entomol. Scand. Suppl.* 12:1–60
60. Hymenoptera Name Server. 1998. http://atbi.biosci.ohio-state.edu:210/hymenoptera/nomenclator.home_page
61. Hymenoptera On-Line Database. 1998. http://iris.biosci.ohio-state.edu/hymenoptera/
62. Iqbal M, Austin AD. 1997. Species richness and endemism of baeine wasps (Hymenoptera: Scelionidae) in Australia. *Mem. Mus. Vic.* 56:455–60
63. Iqbal M, Austin AD. 2000. Systematics of *Ceratobaeus* Ashmead (Hymenoptera: Scelionidae) from Australasia. *Rec. S. Aust. Mus. Monogr. Ser.* 6:1–164
64. Iqbal M, Austin AD. 2000. A preliminary phylogeny for the Baeini (Hymenoptera: Scelionidae): endoparasitoids of spider eggs. See Ref. 11a, pp. 178–91
65. Iqbal M, Austin AD, Belokobylskij SA. 2003. Diversity and biogeography of reduced-winged doryctine wasps (Hymenoptera: Braconidae) in Australasia. *Rec. S. Aust. Mus. Monogr. Ser.* 7:243–47
66. Isidoro N, Bin F, Colazza S, Vinson B. 1996. Morphology of the antennal gustatory sensilla and glands in some parasitic Hymenoptera with hypothesis on their role in sex and host recognition. *J. Hymen. Res.* 5:206–39

66a. Isidoro N, Romani R, Bin F. 2001. Antennal multiporous sensilla: their gustatory features for host recognitions in female parasitic wasps (Insecta, Hymenoptera: Platygastroidea). *Microsc. Res. Technol.* 55:350–58

Annu. Rev. Entomol. 2005.50:553-582. Downloaded from www.annualreviews.org
Access provided by CASA Institution Identity on 06/12/19. For personal use only.

67. Johnson NF. 1984. Revision of the Nearctic species of the *Trissolcus flavipes* group (Hymenoptera: Scelionidae). *Proc. Entomol. Soc. Wash.* 86: 797–807

67a. Johnson NF. 1984. Systematics of Nearctic *Telenomus*: classification and revisions of the *podisi* and *phymatae* species groups (Hymenoptera: Scelionidae). *Bull. Ohio Biol. Surv.* 6:1–113

68. Johnson NF. 1985. Systematics of New World *Trissolcus* (Hymenoptera: Scelionidae) species related to *T. basalis*. *Can. Entomol.* 117:431–45

69. Johnson NF. 1988. Midcoxal articulations and the phylogeny of the order Hymenoptera. *Ann. Entomol. Soc. Am.* 81:870–81

70. Johnson NF. 1991. Revision of Australasian *Trissolcus* species (Hymenoptera: Scelionidae). *Invert. Taxon.* 5: 211–39

71. Johnson NF. 1992. Catalog of world Proctotrupoidea excluding Platygastridae. *Mem. Am. Entomol. Inst.* 51:1–825

72. Johnson NF, Masner L. 1985. Revision of the genus *Psix* Kozlov & Le (Hymenoptera: Scelionidae). *Syst. Entomol.* 10:33–58

73. Johnson NF, Masner L. 2004. The genus *Thoron* Haliday (Hymenoptera: Scelionidae), egg-parasitoids of waterscorpions (Hemiptera: Nepidae) with a key to world species. *Am. Mus. Novit.* 3452:1–16

74. Johnson NF, Musetti L. 2002. Rediscovery of the genus *Platyscelidris* Szabó (Hymenoptera: Scelionidae) and description of a new species. *Proc. Entomol. Soc. Wash.* 104:948–54

75. Kieffer JJ. 1926. Hymenoptera, Proctotrupoidea, Scelionidae. *Das Tierreich* 48:1–885

76. Königsmann E. 1977. Das phylogenetische System der Hymenoptera. Teil 2: Symphyta. *Dtsch. Entomol. Z.* 24:1–40

77. Königsmann E. 1978. Das phylogenetische System der Hymenoptera. Teil 3: Terebrantes (Unterordnung Apocrita). *Dtsch. Entomol. Z.* 25:1–55

78. Königsmann E. 1978. Das phylogenetische System der Hymenoptera. Teil 4: Aculeata (Unterordnung Apocrita). *Dtsch. Entomol. Z.* 25:365–435

79. Kononova SV, Kozlov MA. 2001. *Scelionid Wasps of the Palaearctics. (Hymenoptera, Scelionidae) Subfamilies Teleasinae, Baeinae*. Kiev: Akademperiodika. 439 pp.

80. Kononova SV, Petrov S. 2002. Review of scelionids of the genus *Sparasion* (Hymenoptera, Scelionidae) of Palaearctic region. Communication 1. Characteristics of the genus and description of new species. *Vest. Zool.* 35:23–42

81. Kozlov MA. 1970. Supergeneric groupings of the Proctotrupoidea (Hymenoptera). *Entomol. Rev.* 49:115–27

82. Kozlov MA, Kononova SV. 1983. *Telenominae of the Fauna of the USSR.* Zool. Inst. USSR Acad. Sci. No. 136. 336 pp.

83. Kozlov MA, Kononova SV. 1990. *Scelioninae of the Fauna of the USSR (Hymenoptera, Scelionidae, Scelioninae).* Leningrad: Nauka. 344 pp.

84. LaSalle J, Gauld ID. 1993. Hymenoptera: their diversity, and their impact on the diversity of other organisms. In *Hymenoptera and Biodiversity*, ed. J LaSalle, ID Gauld, pp. 1–26. London: CABI

85. Leiby RW. 1924. The polyembryonic development of *Platygaster vernalis*. *J. Agric. Res.* 28:829–39

86. Masner L. 1956. First preliminary report on the occurrence of genera of the group Proctotrupoidea (Hymenoptera) in CRS (first part—family Scelionidae). *Acta Faun. Entomol. Mus. Nation Prague* 1: 99–126

87. Masner L. 1958. An interesting new genus of Scelionidae from S.W. Africa (Hymenoptera: Proctotrupoidea). *Proc. Entomol. Soc. London Ser. B* 27:101–4

Annu. Rev. Entomol. 2005.50:553-582. Downloaded from www.annualreviews.org
Access provided by CASA Institution Identity on 06/12/19. For personal use only.

88. Masner L. 1964. Remarks on the taxonomy of *Sceliotrachelus* Brues and allied genera (Hymenoptera, Platygastridae). *Psyche* 31:52–56
89. Masner L. 1965. The types of Proctotrupoidea (Hymenoptera) in the British Museum (Natural History) and the Hope Department of Entomology, Oxford. *Bull. Br. Mus. Nat. Hist. Suppl.* 1:1–154
90. Masner L. 1968. A new scelionid wasp from the intertidal zone of South Africa (Hymenoptera: Proctotrupoidea). *Ann. Natal Mus.* 20:195–98
91. Masner L. 1968. A new genus of Scelionidae (Hymenoptera) with austral disjunct distribution. *NZ J. Sci.* 11:652–63
92. Masner L. 1972. The classification and interrelationships of Thoronini (Hymenoptera: Proctotrupoidea, Scelionidae). *Can. Entomol.* 104:833–49
93. Masner L. 1976. Revisionary notes and keys to world genera of Scelionidae (Hymenoptera: Proctotrupoidea). *Mem. Entomol. Soc. Can.* 97:1–87
94. Masner L. 1980. Key to genera of Scelionidae of the Holarctic region, with descriptions of new genera and species (Hymenoptera: Proctotrupoidea). *Mem. Entomol. Soc. Can.* 113:1–54
95. Masner L. 1983. A revision of *Gryon* Haliday in North America (Hymenoptera: Proctotrupoidea: Scelionidae). *Can. Entomol.* 115:123–74
96. Masner L. 1993. Superfamily Platygastroidea. In *Hymenoptera of the World: An Identification Guide to Families*, ed. H Goulet, JT Huber, pp. 558–65. Ottawa: Agric. Can.
97. Masner L. 1995. The proctotrupoid families. In *The Hymenoptera of Costa Rica*, ed. PE Hanson, ID Gauld, pp. 209–65. Oxford: Oxford Univ. Press
98. Masner L, Denis J. 1996. The Nearctic species of *Idris* Foerster. Part I: the *melleus* group (Hymenoptera: Scelionidae). *Can. Entomol.* 128:85–114
99. Masner L, Huggert L. 1979. Revision of world species of Aradophagini (Hymenoptera: Scelionidae). *Can. Entomol.* 111:1089–100
100. Masner L, Huggert L. 1989. World review and keys to genera of the subfamily Inostemmatine with reassignment of the taxa to the Platygastrinae and Sceliotrachelinae (Hymenoptera: Platygastridae). *Mem. Entomol. Soc. Can.* 147:1–214
101. Mason WRM. 1983. The abdomen of *Vanhornia eucnemidarum* (Hymenoptera: Proctotrupoidea) *Can. Entomol.* 115:1483–88
102. Mattiacci L, Vinson SB, Williams HJ, Aldrich JR, Bin F. 1993. A long-range attractant kairomone for egg parasitoid *Trissolcus basalis*, isolated from defensive secretion of its host, *Nezara viridula*. *J. Chem. Ecol.* 19:1167–81
103. Muesebeck CFW. 1977. The parasitic wasps of the genus *Macroteleia* Westwood of the New World (Hymenoptera, Proctotrupoidea, Scelionidae). *Tech. Bull. US Dep. Agric. No. 1565*. 57 pp.
104. Muesebeck CFW. 1979. Proctotrupoidea. In *Catalog of Hymenoptera of America North of Mexico*, ed. KV Krombein, PD Hurd Jr, DR Smith, BD Burks, 1:1121–86. Washington, DC: Smithson. Inst. Press
105. Nagarkatti S, Nagaraja H. 1977. Biosystematics of *Trichogramma* and Trichogrammatoidea. *Annu. Rev. Entomol.* 22:157–76
106. Naumann ID, Masner L. 1985. Parasitic wasps of the proctotrupoid complex: a new family from Australia and a key to world families (Hymenoptera: Proctotrupoidea *sensu lato*). *Aust. J. Zool.* 33:761–83
107. Noda T, Hirose Y. 1989. 'Male second' strategy in the allocation of sexes by the parasitic wasp, *Gryon japonicum*. *Oecologia* 81:145–48
108. Nordlund DA, Strand MR, Lewis WJ, Vinson SB. 1987. Role of kairomones from host accessory gland secretion in host recognition by *Telenomus remus* and

Annu. Rev. Entomol. 2005.50:553-582. Downloaded from www.annualreviews.org
Access provided by CASA Institution Identity on 06/12/19. For personal use only.

Trichogramma pretiosum, with partial characterization. *Entomol. Exp. Appl.* 44:37–43

109. Orr DB. 1988. Scelionid wasps as biological control agents: a review. *Fla. Entomol.* 71:506–28
110. Quicke DLJ. 1997. *Parasitic Wasps*. London: Chapman & Hall
111. Quicke DLJ, Fitton MG, Ingram SN. 1992. Phylogenetic implications of the structure and distribution of ovipositor valvilli in the Hymenoptera. *J. Nat. Hist.* 26:587–608
112. Quicke DLJ, Fitton MG, Notton DG, Broad GR, Dolphin K. 2000. Phylogeny of the subfamilies of Ichneumonidae (Hymenoptera): a simultaneous molecular and morphological analysis. See Ref. 11a, pp. 74–83
113. Quicke DLJ, Fitton MG, Tunstead J, Ingram SN, Gaitens PV. 1994. Ovipositor structure and relationships within the Hymenoptera, with special reference to the Ichneumonoidea. *J. Nat. Hist.* 28:635–82
114. Quicke DLJ, Leralec A, Vilhelmsen L. 1999. Ovipositor structure and function in the parasitic Hymenoptera with an explanation of new hypotheses. *Rendiconti* 47:197–239
115. Rasnitsyn AP. 1980. Origin and evolution of Hymenoptera. *Tran. Paleontol. Inst. Acad. Sci. USSR* 174:1–192
116. Rasnitsyn AP. 1988. An outline of evolution of hymenopterous insects (order Vespida). *Orient. Insects* 22:115–45
117. Ronquist F. 1999. Phylogeny, classification and evolution of the Cynipoidea. *Zool. Scr.* 28:139–64
118. Ronquist F, Rasnitsy AP, Roy A, Eriksson K, Lindgren M. 1999. Phylogeny of the Hymenoptera: a cladistic reanalysis of Rasnitsyn's (1988) data. *Zool. Scr.* 28:13–50
119. Scelionidae. 2004. http://atbi.biosci.ohio-state.edu:210/hymenoptera/eol_scelionidae.home
120. Sharkey MJ. 1981. A revision of the Nearctic species of *Teleas* Latreille (Hymenoptera, Proctotrupoidea, Scelionidae). *Can. Entomol.* 113:907–29
121. Sharkey MJ, Roy A. 2002. Phylogeny of the Hymenoptera: a reanalysis of the Ronquist et al. (1999) reanalysis, with an emphasis on wing venation and apocritan relationships. *Zool. Scr.* 31:57–66
122. Spence JR. 1986. Interactions between the scelionid egg parasitoid *Tiphodytes gerriphagus* (Hymenoptera) and its gerrid host (Heteroptera). *Can. J. Zool.* 64: 2728–38
123. Strand MR. 1986. The physiological interactions of parasitoids with their hosts and their influence on reproductive strategies. In *Insect Parasitoids*, ed. J Waage, D Greathead, pp. 97–136. London: Academic
124. Strand MR. 1988. Variable sex ratio strategy of *Telenomus heliothidis* (Hymenoptera: Scelionidae): adaptation to host and conspecific density. *Oecologia* 77:219–24
125. Strand MR, Grbic M. 1997. The development and evolution of polyembryonic insects. *Curr. Top. Dev. Biol.* 35:121–59
126. Strand MR, Meola SM, Vinson SB. 1986. Correlated pathological symptoms in *Heliothis virescens* eggs with development of the parasitoid *Telenomus heliothidis*. *J. Insect Physiol.* 32:389–402
127. Strand MR, Vinson SB. 1983. Analyses of an egg recognition kairomone of *Telenomus heliothidis* (Hymenoptera: Scelionidae) isolation and host function. *J. Chem. Ecol.* 9:423–32
128. Szabó JB. 1959. Notes on the new tribe Amitini with the description of a new genus and some new species of the Arctogaea (Hymenoptera: Proctotrupoidea: Platygastridae) *Ann. Hist. Nat. Mus. Nat Hung.* 51:389–96
129. Szabó JB. 1966. Ökologische, ethologische, tiergeographishe und systematische Untersuchungen an paläatktischen Gryoninen (Hymenoptera: Proctotrupoidea,

Scelionidae). *Acta Zool. Acad. Sci. Hung.* 12:419–49

129a. Vet LEM, Dicke M. 1992. Ecology of infochemical use by natural enemies in a tritrophic context. *Annu. Rev. Entomol.* 37:141–72

130. Vilhelmsen L. 2003. Flexible ovipositor sheaths in parasitoid Hymenoptera (Insecta). *Arthropod Struct. Dev.* 32:277–87

131. Vlug HJ. 1995. Catalogue of the Platygastridae (Platygastroidea) of the world (Insecta: Hymenoptera). Hymenopterorum Catalogus, No. 1–168. Amsterdam: SPB Acad.

132. Waage JK. 1982. Sib-mating and sex ratio strategies in scelionid wasps. *Ecol. Entomol.* 7:103–12

133. Wharton RA. 1993. Bionomics of the Braconidae. *Annu. Rev. Entomol.* 38: 121–43

134. Whitfield JB. 1992. Phylogeny of the non-aculeate Apocrita and the evolution of parasitism in the Hymenoptera. *J. Hymen. Res.* 1:3–14

135. Whitfield JB. 1998. Phylogeny and evolution of host-parasitoid interactions in Hymenoptera. *Annu. Rev. Entomol.* 43: 129–51

136. Whitfield JB, Johnson NF, Hamerski MR. 1989. Identity and phylogenetic significance of the metapostnotum in nonaculeate Hymenoptera. *Ann. Entomol. Soc. Am.* 82:663–73

137. Wilson F. 1961. Adult reproductive behaviour in *Asolcus basalis* (Hymenoptera: Scelionidae). *Aust. J. Zool.* 9: 737–51

138. Yeates DK, Harvey MS, Austin AD. 2003. New estimates for terrestrial arthropod species-richness in Australia. *Rec. S. Aust. Mus. Monogr. Ser.* 7:231–41

139. Zherikhin VV, Sukachëva ID. 1971. On Cretaceous insect-bearing ambers (retinites) of northern Sibera. In *Doklady 24 Ezhegodnom Chtenii pamyati NA Kholodkovskogo*, pp. 3–48. Leningrad: Publ. House Nauka

Annu. Rev. Entomol. 2005.50:553-582. Downloaded from www.annualreviews.org
Access provided by CASA Institution Identity on 06/12/19. For personal use only.

Annual Review of Entomology
Volume 50, 2005

CONTENTS

BIOLOGY AND MANAGEMENT OF INSECT PESTS IN NORTH AMERICAN INTENSIVELY MANAGED HARDWOOD FOREST SYSTEMS, *David R. Coyle, T. Evan Nebeker, Elwood R. Hart, and William J. Mattson* 1

THE EVOLUTION OF COTTON PEST MANAGEMENT PRACTICES IN CHINA, *K.M. Wu and Y.Y. Guo* 31

MOSQUITO BEHAVIOR AND VECTOR CONTROL, *Helen Pates and Christopher Curtis* 53

THE GENETICS AND GENOMICS OF THE SILKWORM, *BOMBYX MORI*, *Marian R. Goldsmith, Toru Shimada, and Hiroaki Abe* 71

TSETSE GENETICS: CONTRIBUTIONS TO BIOLOGY, SYSTEMATICS, AND CONTROL OF TSETSE FLIES, *R.H. Gooding and E.S. Krafsur* 101

MECHANISMS OF HOPPERBURN: AN OVERVIEW OF INSECT TAXONOMY, BEHAVIOR, AND PHYSIOLOGY, *Elaine A. Backus, Miguel S. Serrano, and Christopher M. Ranger* 125

FECAL RESIDUES OF VETERINARY PARASITICIDES: NONTARGET EFFECTS IN THE PASTURE ENVIRONMENT, *Kevin D. Floate, Keith G. Wardhaugh, Alistair B.A. Boxall, and Thomas N. Sherratt* 153

THE MEVALONATE PATHWAY AND THE SYNTHESIS OF JUVENILE HORMONE IN INSECTS, *Xavier Bellés, David Martín, and Maria-Dolors Piulachs* 181

FOLSOMIA CANDIDA (COLLEMBOLA): A "STANDARD" SOIL ARTHROPOD, *Michelle T. Fountain and Steve P. Hopkin* 201

CHEMICAL ECOLOGY OF LOCUSTS AND RELATED ACRIDIDS, *Ahmed Hassanali, Peter G.N. Njagi, and Magzoub Omer Bashir* 223

THYSANOPTERA: DIVERSITY AND INTERACTIONS, *Laurence A. Mound* 247

EFFECTS OF PLANTS GENETICALLY MODIFIED FOR INSECT RESISTANCE ON NONTARGET ORGANISMS, *Maureen O'Callaghan, Travis R. Glare, Elisabeth P.J. Burgess, and Louise A. Malone* 271

Annu. Rev. Entomol. 2005.50:553-582. Downloaded from www.annualreviews.org
Access provided by CASA Institution Identity on 06/12/19. For personal use only.

INVASIVE PHYTOPHAGOUS PESTS ARISING THROUGH A RECENT TROPICAL EVOLUTIONARY RADIATION: THE *BACTROCERA DORSALIS* COMPLEX OF FRUIT FLIES, *Anthony R. Clarke, Karen F. Armstrong, Amy E. Carmichael, John R. Milne, S. Raghu, George K. Roderick, and David K. Yeates* 293

PHEROMONE-MEDIATED AGGREGATION IN NONSOCIAL ARTHROPODS: AN EVOLUTIONARY ECOLOGICAL PERSPECTIVE, *Bregje Wertheim, Erik-Jan A. van Baalen, Marcel Dicke, and Louise E.M. Vet* 321

EGG DUMPING IN INSECTS, *Douglas W. Tallamy* 347

ECOLOGICAL, BEHAVIORAL, AND BIOCHEMICAL ASPECTS OF INSECT HYDROCARBONS, *Ralph W. Howard and Gary J. Blomquist* 371

THE EVOLUTION OF MALE TRAITS IN SOCIAL INSECTS, *Jacobus J. Boomsma, Boris Baer, and Jürgen Heinze* 395

EVOLUTIONARY AND MECHANISTIC THEORIES OF AGING, *Kimberly A. Hughes and Rose M. Reynolds* 421

TYRAMINE AND OCTOPAMINE: RULING BEHAVIOR AND METABOLISM, *Thomas Roeder* 447

ECOLOGY OF INTERACTIONS BETWEEN WEEDS AND ARTHROPODS, *Robert F. Norris and Marcos Kogan* 479

NATURAL HISTORY OF PLAGUE: PERSPECTIVES FROM MORE THAN A CENTURY OF RESEARCH, *Kenneth L. Gage and Michael Y. Kosoy* 505

EVOLUTIONARY ECOLOGY OF INSECT IMMUNE DEFENSES, *Paul Schmid-Hempel* 529

SYSTEMATICS, EVOLUTION, AND BIOLOGY OF SCELIONID AND PLATYGASTRID WASPS, *A.D. Austin, N.F. Johnson, and M. Dowton* 553

INDEXES

Subject Index 583
Cumulative Index of Contributing Authors, Volumes 41–50 611
Cumulative Index of Chapter Titles, Volumes 41–50 616

ERRATA

An online log of corrections to *Annual Review of Entomology* chapters may be found at http://ento.annualreviews.org/errata.shtml

Annu. Rev. Entomol. 2005.50:553-582. Downloaded from www.annualreviews.org
Access provided by CASA Institution Identity on 06/12/19. For personal use only.
