# Supplementary material for: Spectral measure of color variation of black-orange-black (BOB) pattern in small parasitoid wasps (Hymenoptera: Scelionidae), a statistical approach
Source: PLoS One. 2019 Oct 24;14(10):e0218061. doi: 10.1371/journal.pone.0218061 (PMC6812806; doi:10.1371/journal.pone.0218061)
Supplement: Attachment — Submitted filename: Murphy2007.pdf [file pone.0218061.s010.pdf]

Biological Journal of the Linnean Society, 2007, **91**, 653–669. With 4 figures

Phylogeny of the platygastroid wasps (Hymenoptera) based on sequences from the 18S rRNA, 28S rRNA and cytochrome oxidase *I* genes: implications for the evolution of the ovipositor system and host relationships

NICHOLAS P. MURPHY[1], DANIELLE CAREY[1], LYDA R. CASTRO[2], MARK DOWTON[2] and ANDREW D. AUSTIN[1]*

[1]*Centre for Evolutionary Biology and Biodiversity, School of Earth and Environmental Sciences, The University of Adelaide, South Australia 5005, Australia*
[2]*Institutes of Biomolecular Science and Conservation Biology, Department of Biology, Wollongong University, Wollongong, New South Wales 2522, Australia*

Received 3 March 2006; accepted for publication 2 October 2006

The Platygastroidea are a diverse group of mostly small to tiny wasps, the common biology for which is endoparasitism of insect and spider eggs. No analytically-based phylogeny exists for the superfamily, and the current suprageneric classification is flawed in part because of its reliance on homoplasious and pleisiomorphic morphological characters. To determine platygastroid relationships as a basis for investigating host and ovipositor evolution, phylogenies of >70 in-group species (representing 55 genera) were reconstructed by parsimony and Bayesian methods using three molecular markers; the mitochondrial cytochrome oxidase *I* and the nuclear genes *28S* and *18S rRNA*. The results strongly support the monophyly of the superfamily and one of the two families, Platygastridae, but the Scelionidae are most likely polyphyletic. However, within the Scelionidae, there is a well supported 'main scelionid clade' that contains the majority of genera assigned to the family. At the subfamilial level, both putative subfamilies of Platygastridae, the Platygastrinae, and Sceliotrachelinae, are likely to be polyphyletic. Within the Scelionidae, both the Teleasinae and Telenominae are monophyletic, but the Scelioninae is clearly not so. The current tribal classification for the Scelionidae is in need of major reassessment because no tribes, with the exception of the Scelionini *s.s.*, were found to be monophyletic. Further illustrating the problems associated with the current classification is the nonmonophyly of a number of genera, namely *Opisthacantha Caloteleia*, *Telenomus*, *Trimorus*, *Teleas* and *Idris*. Analysis of ovipositor evolution in the superfamily revealed that the *Ceratobaeus*-type ovipositor system is ancestral; however, this trait was lost prior to the evolution of the main scelionid clade, for which the *Scelio*-type ovipositor system is ancestral and defines a mostly paraphyletic assemblage. Ancestral state analysis indicates that the *Ceratobaeus*-type ovipositor was subsequently re-evolved in the main scelionid clade, representing a possible contradiction of Dollo's law. Previously, the tribal placement has been used to predict the host associations of genera for which host data were unavailable. However, the fact that most tribes are not monophyletic throws into doubt any such speculation based on the current classification. © 2007 The Linnean Society of London, *Biological Journal of the Linnean Society*, 2007, **91**, 653–669.

ADDITIONAL KEYWORDS: Platygastidae – Scelionidae – Scelioninae – Teleasinae – Telenominae.

INTRODUCTION

The Platygastroidea and the two families it includes, the Scelionidae and Platygastridae, is a diverse assemblage of parasitic wasps comprising some 4500 described species worldwide (Johnson, 1992). They are generally small, with most species being less than 2.5 mm. The great majority of species are morphologically simplified compared with other parasitic Hymenoptera. All scelionids are endoparasitoids of the eggs of insects and spiders, with numerous higher-level taxa apparently specializing in specific host

*E-mail: andy.austin@adelaide.edu.au

groups (Masner, 1976; Austin and Field, 1997; Austin, Johnson & Dowton, 2005). Like the Scelionidae, several groups of Platygastridae are also endoparasitoids of eggs, mostly of Coleoptera, as well as the later stages of sessile hosts, such as planthoppers, whiteflies, aphids and mealybugs. However, the majority of platygastrids attack gall flies (Cecidomyiidae), ovipositing either in the egg or early larva of the host, and completing their development in the larva (Masner and Huggert, 1989; Austin and Field, 1997; Austin *et al*., 2005).

Although the relationships of the Platygastroidea to other parasitic Hymenoptera remain unclear (Ronquist *et al*., 1999; Dowton and Austin, 2001; Sharkey and Roy, 2002; Austin *et al*., 2005), the monophyly of the superfamily is undisputed. The evidence supporting this comes from two unique character systems within the Hymenoptera; a modification of the abdomen into a pumping organ used to change the hydrostatic pressure of the haemolymph to assist with ovipositor extension and retraction, and the sensilla of the female antenna (Masner, 1993, 1995; Austin *et al*., 2005). Two functionally different arrangements of the ovipositor system occur within the superfamily. In the first ('*Ceratobaeus* type'), ovipositor extension is achieved by contraction of muscles assisted by a change in hydrostatic pressure (Austin, 1983; Austin and Field, 1997). In the second arrangement (termed the '*Scelio*-type'), no such apodemes and associated muscles are present and the ovipositor is extended at the end of a telescopic membranous tube by hydrostatic pressure alone (Field and Austin, 1994; Austin and Field, 1997). Austin and Field (1997) postulated that the '*Ceratobaeus*-type' was plesiomorphic within the superfamily as it was found across a wide range of scelionid tribes, including several putatively primitive genera, as well as in all Platygastridae. The '*Scelio*-type' was considered to be more derived as it involved the apparent loss of muscles and, for some genera, structures apparently homologous to the apodemes in the '*Ceratobaeus*-type' are present but incorporated into the wall of the telescopic membranous tube (Field and Austin, 1994; Austin and Field, 1997).

The other character unique to the Platygastroidea is that the apical segments of the female antenna have one or two large multiporous basiconic sensilla per segment that are apparently used for recognition of host-associated cues (Bin, 1981; Bin and Vinson, 1986; Isidoro *et al*., 1996).

Surprisingly, given the diversity and biological interest in the group, the Platygastroidea have not been exposed to any comprehensive phylogenetic analysis aimed at resolving higher-level relationships. Most important is whether or not the two families are monophyletic. In this respect, the Platygastridae is putatively a well-supported group, albeit mostly on the basis of a series of character reductions. In comparison, the Scelionidae are not defined by any unique synapomorphy, and the family is possibly paraphyletic (Austin *et al*., 2005), as indicated by analysis of the ovipositor system and a molecular phylogeny aimed at resolving broader issues associated with the phylogeny of apocritan families and superfamilies (Dowton and Austin, 2001).

Relationships at lower taxonomic levels are also problematic, although the current classification for the Scelionidae provides a useful starting point. A recent assessment of the phylogenetic status of currently recognized higher-level groups (Austin and Field, 1997) concluded that the largest subfamily, Scelioninae, is defined only by symplesiomorphies and is probably paraphyletic, whereas the Teleasinae, Telenominae, and several tribes probably represent natural assemblages, although many are not convincingly supported on morphological grounds. Of all characters examined in recent years, the ovipositor system has been the most useful for inferring putative relationships, with the telescopic *Scelio*-type system putatively supporting a natural assemblage comprising seven tribes and approximately 55 genera (Austin and Field, 1997). This group has tentatively been referred to as the Scelionini *s.l.* to identify it within the current classification (Austin and Field, 1997). However, the monophyly of this group has not been examined within a broader phylogenetic analysis and the question still remains: has this character system evolved independently several times or does it, indeed, support a single lineage within the superfamily?

Little work has been undertaken on the phylogeny of individual tribes. A sister-group relationship has been postulated between the Embidobiini and Baeini (Galloway and Austin, 1984), with searching for host silk (embiids and spiders) being proposed as a possible synapomorphy for these tribes. However, a recent molecular study of the Baeini has shown it to be polyphyletic (Carey, Murphy & Austin, 2006). The monophyly of other tribes remains untested and most appear to be characterized by a suite of plesiomorphic and putatively apomorphic characters, as well as others of unknown polarity (Masner, 1976; Austin and Field, 1997). Several contain genera with both types of ovipositor (e.g. Baryconini, Psilanteridini, Calliscelionini; *sensu* Masner, 1976) and, given the nonmonophyly of Baeini, much of the tribal classification may be in doubt. Furthermore, the position and relationships of several 'primitive' genera such as *Nixonia*, *Archaeoteleia*, and *Neuroscelio* (Masner, 1958, 1968; Galloway, Austin & Masner, 1992) is clouded by their retention of putative plesiomorphic characters that are common in fossil taxa.

Where a framework at least exists for testing specific higher-level hypotheses for the Scelionidae, solid

information on relationships within the Platygastridae is virtually absent. The reassignment of genera into two newly constituted subfamilies (Sceliotrachelinae and Platygastrinae) by Masner and Huggert (1989), following a detailed morphological study, has still not shed much light on the evolution of the group. Of the two currently recognized subfamilies, the Platygastrinae is possibly monophyletic but this proposal is supported more on having a common host group, the Cecidomyiidae, than on any recognizable morphological synapomorphies. However, the Sceliotrachelinae is probably paraphyletic as indicated by its lack of defining features and more diverse array of hosts. Of the nine recognized informal generic groups (Masner and Huggert, 1989; Austin and Field, 1997), none are demonstrably monophyletic and most are defined on reductional synapomorphies or similarities in body form, antennal structure or wing characteristics.

The current lack of a robust phylogenetic scheme for the Platygastroidea is a major drawback to current systematic and biological studies that prevents any meaningful assessment of relationships and evolution within the group. Based on the evidence to date (Austin *et al*., 2005), it is clear that the extreme level of reductional homoplasy compromises the utility of morphological characters in developing a robust hypothesis. Although morphological studies will eventually contribute to such hypotheses, no comprehensive data sets are yet available. In the present study, we use DNA sequence data from multiple genes to examine the major questions associated with platygastroid evolutionary relationships, to assess the robustness of the current higher-level classification, and examine the evolutionary significance of the ovipositor system and the pattern of host associations.

MATERIAL AND METHODS

Taxonomic sampling

The taxa sampled are listed in Tables 1, 2, 3. In total, 83 platygastroid species were sampled representing 58 genera from all currently recognized platygastroid subfamilies and the majority of tribes. Where possible, multiple genera from subfamilies and tribes, and multiple species from speciose genera, were included to give a more representative taxon coverage. Outgroup taxa (Table 3) were chosen from a broad range of families because the sister group to the Platygastroidea is still unresolved.

Due to the small size of many species (> 2 mm), whole animal extractions were necessary for some specimens, whereas others were extracted from significant parts of the abdomen and thorax. Where possible, voucher specimens have been retained in the collection of A. Austin using the code numbers in Tables 1, 2, 3. A high definition AutoMontage image library of specimens used for sequencing was also constructed to allow for future identification of specific taxa (available from A. D. Austin).

DNA extraction, amplification, and sequencing

Genomic DNA was extracted from ethanol preserved tissue using the Puregene DNA Purification Kit (Gentra Systems Inc.). Partial sequences were obtained from an approximately 600-bp product obtained by polymerase chain reaction (PCR) amplification of the mitochondrial protein coding cytochrome oxidase *I* gene (*COI*) using the primers C1-J-1718 and C1-N-2329 (Simon *et al*., 1994). Amplification of an approximately 800-bp segment of the nuclear *28S rDNA* gene, spanning the D2 and D3 segments, was

Table 1. Platygastridae included in analysis and genes amplified for each species

Subfamily	Genus	Reference number	*18S*	*28S*	*COI*
Platygastrinae	*Allostemma* sp.	M327	Y	Y	Y
	Inostemma sp.	M350	Y	Y	Y
	Iphitrachelus sp.	M326	Y	Y	X
	Trichacis sp.	M384	Y	Y	X
	Piestopleura sp.	M318	Y	Y	Y
	Synopeas sp. 1	M334	Y	Y	Y
	Synopeas sp. 2	M411	Y	Y	X
Sceliotrachelinae	*Allotropa* sp.*	M332	Y	X	X
	Amitus sp. 1	M142	Y	Y	Y
	Amitus sp. 2	M406	X	Y	Y
	Aphanomerus sp.	M246	Y	Y	Y
	Fidobia sp.	M331	Y	Y	Y
	Platygastoides sp.	M279	Y	Y	Y

*Only one gene amplified and species not included in final analysis.

Table 2. Scelionidae included in analysis and genes amplified for each species

Subfamily/tribe	Genus	Reference number	*18S*	*28S*	*COI*
Aradophagini	*Aradophagus* sp.	M172	Y	Y	Y
Baeini	*Baeus* sp.	M383	X	Y	Y
	Ceratobaeus sp.	M145	Y	Y	Y
	Idris sp. 1	M144	Y	Y	Y
	Idris sp. 2	M280	Y	Y	Y
	Mirobaeoides sp.	M282	Y	Y	Y
	Neobaeus sp.	M438	X	Y	Y
	Odontocolus sp.*	M290	X	Y	X
Baryconini	*Baryconus* sp. 1*	M175	X	Y	X
	Baryconus sp. 2	M195	Y	Y	Y
	Baryconus sp. 3	M412	Y	Y	X
	Chromoteleia sp.	M381	Y	Y	X
	Oxyscelio sp. 1*	M173	X	Y	X
	Oxyscelio sp. 2	M422	Y	Y	Y
	Oxyscelio sp. 3	M433	Y	Y	Y
Calliscelionini	*Anteromorpha* sp.	M421	Y	Y	Y
	Calliscelio laticinctus	M379	Y	Y	Y
	Holoteleia sp.*	M300	X	Y	X
	Macroteleia sp. 1*	M091	X	Y	X
	Macroteleia sp. 2	M303	Y	Y	X
	Macroteleia sp. 3	M368	Y	Y	Y
	Macroteleia sp. 4	M385	Y	Y	X
	Oethecoctonus sp.	M373	Y	Y	Y
	Paridris sp.*	M413	X	Y	X
	Probaryconus sp. 1	M286	Y	Y	Y
	Probaryconus sp. 2	M415	Y	Y	X
	Triteleia sp.	M409	Y	Y	Y
Cremastobaeini	*Cremastobaeus* sp.	M312	Y	Y	Y
Embidobiini	*Embidobia* sp.	M428	Y	Y	Y
Gryonini	*Dyscritobaeus* sp. 1	M278	Y	Y	X
	Dyscritobaeus sp. 2	M429	Y	Y	Y
	Gryon sp. 1*	M170	X	Y	X
	Gryon sp. 2*	M171	X	Y	X
	Gryon varicornis	M410	Y	X	Y
	Gryon saxatlis	M427	Y	Y	Y
	Gryon exsctella	M430	Y	Y	Y
	Neuroscelio doddi	M437	Y	Y	X
Neoscelionini	*Neoscelio* sp.	M393	Y	X	Y
Psilanteridini	*Caloteleia* sp. 1	M335	Y	Y	Y
	Caloteleia sp. 2	M420	Y	Y	Y
	Duta sp.	M387	Y	Y	Y
	Genatropis sp.	M360	Y	Y	X
	Mallateleia sp.	M051	Y	Y	Y
	Nyleta sp.	M323	Y	Y	Y
	Opistacantha sp. 1*	M191	X	Y	X
	Opistacantha sp. 2	M361	Y	Y	Y
	Opistacantha millipes	M405	Y	Y	Y
	Psilanteris sp.*	M363	X	X	Y
Scelionini	*Scelio fulgidus*	M003	Y	Y	Y
	Scelio sp.	M436	Y	Y	Y
	Acanthoscelio acutus	M419	Y	Y	Y
Sparasionini	*Archaeoteleia* sp.	M293	Y	Y	Y
	Sceliomorpha sp.	M356	Y	Y	X
	Sparasion sp.	M143	Y	Y	Y

Table 2. *Continued*

Subfamily/tribe	Genus	Reference number	*18S*	*28S*	*COI*
Teleasinae	*Teleas* sp.	M285	Y	Y	X
	Unknown Telaesinae	M329	Y	Y	Y
	Trimorus sp. 1	M172	Y	Y	Y
	Trimorus sp. 2	M371	Y	Y	X
	Trimorus sp. 3	M403	X	Y	Y
	Gryonoides glabriceps	M341	Y	Y	Y
	Gryonella sp.*	M183	X	Y	X
Telenominae	*Phanuromyia* sp.	M372	Y	Y	Y
	Telenomus busseolae	M133	Y	Y	Y
	Telenomus crassicalva	M304	Y	Y	Y
	Telenomus podisi	M305	Y	Y	X
	Telenomus sp.	M432	Y	Y	Y
	Trissolcus sp. 1*	M058	X	Y	X
	Trissolcus sp. 2	M289	X	Y	Y
	Trissolcus sp. 3	M424	Y	Y	Y
Thoronini	*Thoron* sp.	M193	Y	Y	Y
	Tiphodytes sp.	M194	Y	Y	Y
Unknown	*Sceliacanthella* sp.	M357	Y	Y	X

*Only one gene amplified and species not included in final analysis.

Table 3. Outgroup taxa included in analysis

Family	Genus	Reference number	*18S*	*28S*	*COI*
Megaspilidae	*Dendrocerus* sp.	M370	Y	Y	Y
Stephanidae	*Megischus texanus*	M039	Y	Y	Y
Maamingidae	*Maaminga rangi*	M070	Y	Y	Y
Vanhorniidae	*Vanhornia eunemidarum*	M012	Y	Y	Y
Roproniddae	*Ropronia garmani*	M018	Y	Y	Y
Diapriidae	*Diphoropria* sp.	M229	Y	Y	Y
Figitidae	*Anarcharis zealandica*	M064	Y	Y	Y
Eulophidae	*Melitobia australica*	M159	Y	Y	Y
Pelecinidae	*Pelecinus polyturator*	M022	Y	Y	Y
Pteromalidae	*Pteromalus puparum*	–	Y	Y	Y

implemented using the primers D2-3665 F and D3-4413 R (Gillespie, Yoder & Wharton, 2005). Amplification of an approximately 800-bp segment of the nuclear *18S rDNA* gene was implemented using the primers *18S*ai and *18S*bi (Whiting *et al.*, 1997; Castro & Dowton, 2006).

PCR amplifications were carried out in 25 µL containing PCR buffer, 0.2 mM of each dNTP, 0.4 µM of each primer, 2 mM $MgCl_2$, 0.5 units of AmpliTaq Gold DNA Polymerase (Applied Biosystems Inc.) and 25–100 ng of genomic DNA. Thermocycling conditions were: an initial denaturation step of 95 °C for 5 min, followed by 35 cycles of 95 °C for 30 s, an annealing temperature of 50 °C for 30 s, and an extension temperature of 72 °C for 30 s. This was then followed by an additional extension of 72 °C for 3 min. PCR products were purified using the Ultraclean PCR Clean-up Kit (MOBIO Laboratories Inc.). Sequencing reactions were performed using ABI Big Dye Terminator Chemistry; fragments were resolved on an ABI 3700 sequencer.

Sequence alignment and analysis

The *COI* gene was aligned by eye because few insertion/deletion events were present. The amino acid sequence was translated as a test of the presence of nuclear paralogues (e.g. stop codons). Alignment of both nuclear RNA genes (*28S* and *18S rRNA*) was undertaken manually according to secondary structure following the convention of Kjer (2004) and Gillespie (2004). Initial alignments were based on published secondary struc-

ture models, *28S rRNA* according to ichneumonoid (Gillespie *et al*., 2005) and chrysomelid (Gillespie *et al*., 2004), and *18S rRNA* according to the general insect secondary structure proposed by Kjer (2004). Variable regions specific to individual species and platygastroids in general were determined using the program *mfold*, version 2.1 (Mathews *et al*., 1999; Zuker, 2003), which estimates rRNA secondary structure based on free energy minimizations.

Manual alignment based on secondary structure has a number of advantages, the most significant being that biological information can be considered when making alignment considerations. Another advantage of secondary structure alignments is the ability to implement the doublet model of sequence evolution (Schoniger and von Haeseler, 1994) for the paired bases of the stem regions of ribosomal sequences. There are, however, disadvantages to the manual alignment method, in that the alignment is considerably time consuming, especially when compared with algorithmic approaches such as that implemented by Clustal X. The subjective nature of manual alignment of ambiguous regions also invokes a philosophical argument concerning the repeatability of such approaches (Ogden, Whiting & Wheeler, 2005). Despite the arguments for and against different alignment methods, if the data being examined are robust then differing alignment methods should have little effect on the overall outcomes of phylogenetic analysis.

To test the robustness of the data set against the possible shortcomings of manual alignment techniques (i.e. subjective and prone to bias), a comparison was made between Clustal X alignments and the manual alignments. Alignment of the *28S rRNA* and *18S rRNA* genes using Clustal X (Thompson *et al*., 1997) employed a number of gap opening/gap extension schemes (gap to change costs: 1 : 1, 1 : 2, 1 : 5, 1 : 10). Regions of uncertain alignment that varied markedly between different alignment schemes were deleted (Gatesy, Desalle & Wheeler, 1993). A Bayesian approach was undertaken to compare the alignment methods by examining differences in the proportion of significantly supported nodes between trees derived from the two alignments for each gene.

Impact of incomplete data

Due to problems such as degraded DNA and primer mismatch, it is often not possible to obtain a complete nucleotide data set for all taxa chosen. Reducing the data set to contain only taxa for which the entire sequence data is available limits the taxonomic scope (Hughes and Vogler, 2004) and may result in the deletion of taxa integral to the overall conclusions. Although the impact of missing data on phylogenetic analysis has not been thoroughly tested, Wiens (1998b) found in a simulated study that adding taxa with missing data generally increased phylogenetic accuracy, whereas an empirical study by Hughes and Vogler (2004) found that including taxa with missing data increased the resolution. Given that few studies have examined the effect of incomplete data, it is important to fully explore its impact on any new phylogenetic analysis. To assess the impact of incomplete data sets, a Bayesian approach was undertaken examining changes in the proportion of significantly supported nodes for between data sets containing only taxa with complete nucleotide data vs. those containing two of three and one of three genes.

Phylogenetic reconstruction

As significant heterogeneity in base composition can lead to erroneous grouping of unrelated taxa with similar composition (Lockhart *et al*., 1994), homogeneity chi-squared analysis of each gene was undertaken using PAUP* 4.0b10 (Swofford, 2000) to identify significant differences in base composition. The presence of nucleotide saturation, in particular at the third codon position of *COI*, was examined by plotting observed transitions and transversions against the overall nucleotide divergence.

The combination of data partitions during phylogenetic analyses should increase phylogenetic accuracy provided that the partitions share a common evolutionary history. Recent studies have shown that the ILD test is unreliable for assessing both incongruent histories and combinability of data partitions (Dowton and Austin, 2002). Therefore, the presence of significant heterogeneity between partitions (genes) was assessed using the approach outlined by Wiens (1998a), comparing the confidence and support in different topologies derived from the different genes, and was implemented using Bayesian posterior probabilities (Reeder, 2003).

Maximum parsimony (MP) analyses were performed in PAUP* using the heuristic search algorithm with 100 random sequence addition replicates to eliminate any bias from taxon ordering in the data sets. Gaps were treated as missing data and two weighting schemes were analysed; characters were either weighted equally, or transition substitutions at the third codon position in *COI* were ignored. Confidence in the MP trees was assessed from 1000 nonparametric bootstrap pseudo-replications.

Bayesian phylogenetic analyses were performed using MrBayes (Huelsenbeck & Ronquist, 2001). The appropriate model of evolution was chosen by the Akaike information criterion using Modeltest (Posada and Crandall, 1998) on all data partitions separately (inferred stem and loop regions of *28S rRNA* and *18S rRNA* and the separate codon positions of *COI*). The

optimal partitioning strategy was examined using Bayes factor analysis (Brandley, Schmitz & Reeder, 2005) comparing the harmonic means of the log likelihoods of different possible partition combinations; for example; no partitions; three partitions (*18S* + *28S* + *COI*); seven partitions (paired *18S* + unpaired *18S* + paired *28S* + unpaired *28S* + *COI* first codon position + *COI* second codon position + *COI* third codon position). Bayes factor analysis was also undertaken to examine any differences between coding the paired rRNA sites with the doublet model and coding them with the 4 × 4 model of sequence evolution. Once the partitioning strategy was chosen, the model parameters were unlinked and estimated separately for each partition.

MrBayes analyses were run across four chains for five million generations sampling every 100 generations, and stationarity was determined from an examination of log likelihoods and model parameters. Trees recovered prior to stationarity were discarded and Bayesian posterior probabilities of each bipartition, representing the percentage of times each node was recovered, were calculated from the remaining trees. Multiple runs were performed to assess that all parameters were not considerably different at stationarity based on alternate prior probabilities.

The AU test (Shimodaira, 2002) was used to assess a number of *a posteriori* alternative hypotheses based on current classifications. Ancestral states and transitions between *Scelio*- and *Ceratobaeus*-type ovipositor systems were reconstructed on the MP and Bayesian phylogenies using both symmetrical and asymmetrical maximum likelihood methods implemented in Mesquite (Maddison & Maddison, 2003a, b). Ovipositor system states were taken from Austin and Field (1997). Rates of change were estimated from the data using both character distribution and tree (including branch length information). Maximum likelihood methods of ancestral character state reconstruction are valuable because they use branch length information and quantify uncertainty in character state reconstruction (Pagel, 1999). An ancestral state at a given node was considered significant and preferred over the other if its likelihood value was higher by at least two log units than the likelihood value of the other ancestral state [likelihood decision threshold values (T) set to two by default in Mesquite].

RESULTS

Sequence variation and alignment

Not all taxa could be successfully sequenced, with only 46 platygastroids being sequenced for all three genes, 70 taxa for at least two of the three genes and 83 for at least one of the three genes (Tables 1, 2, 3). All sequences have been deposited in GenBank (accession nos DQ888372–DQ888571). The alignment of *COI* introduced gaps at two positions corresponding to amino acid indel events. Comparison of the *COI* sequences against other Hymenoptera *COI* sequences from GenBank suggests that at one site, a deletion of three codons has occurred in all platygastroids and at another site, a single codon deletion has occurred in *Acanthoscelio* sp., *Baryconus* sp. 2, *Macrotelia* sp. 3 *Teleas* sp., and *Trimorus* sp. 3, and a deletion of two codons has occurred in *Mallotelia* sp., *Thoron* sp., all *Gryon* species and all Telenominae, with the exception of *Phanuromyia* sp. and *Telenomus crassicalva* where a three codon deletion has occurred.

Alignment of the *28S rRNA* according to the secondary structure resulted in 510 bp being deemed as 'paired' or putative stem sites and 561 bp as 'unpaired' or putative loops; of these, 185 bp were deemed unalignable and removed from further analysis. The Clustal X alignment of the *28S rRNA* gene resulted in approximately 200 bp being removed due to ambiguous alignment. A direct comparison between the two alignments showed that the majority of ambiguous regions removed were shared between the two alignment methods; however, there were minor differences. Bayesian phylogenetic trees were constructed for the *28S rRNA* gene, for both the Clustal X alignment and the secondary structure alignment. No major differences in support (> 0.95) were found for the alternate topologies.

The alignment of the *18S rRNA* according to the secondary structure method resulted in 272 bp being deemed as 'paired' or putative stem sites and 582 bp as 'unpaired' or putative loops; of these, 188 bp were deemed unalignable and removed from further analysis. The Clustal X alignment of the *18S rRNA* gene resulted in approximately 80 bp being removed due to ambiguous alignment. A direct comparison between alignments was made as per the *28S rRNA* gene above, with no major differences in support (> 0.95) for the alternate topologies.

No significant difference in base composition across taxa was detected for any of the genes (*28S rRNA*: $P = 0.2867$, *18S rRNA*: $P = 1.00$, *COI*: $P = 0.7736$). However, analysis of separate codon positions in the *COI* gene showed that the third base position contained significant heterogeneity in base composition ($P < 0.05$), whereas there were no significant differences at the other two base positions (first position: $P = 1.00$, second position: $P = 1.00$). Saturation plots of the different partitions indicated that the *COI* third codon positions might be influenced by saturation, whereas all other partitions appear to be unaffected (data not shown).

Data combinability

A comparison of the topologies and Bayesian support between the gene regions suggested no major phylo-

genetic incongruence. There were no conflicting significantly supported clades between the phylogenetic trees derived from the three genes (trees not shown, available on request). The *28S rRNA* tree contained the most highly supported nodes, whilst *18S rRNA* and *COI* contained slightly fewer. Based on these results, the decision was made to combine the three genes for further analysis.

Data partitions and model

Table 4 shows the models chosen for each partition by Modeltest using the Akaike information criterion. The Bayes factor analysis of the harmonic means of the data partitions revealed that the partitioning of the nuclear rRNA genes into separate paired and unpaired partitions but keeping the *COI* as a single partition was the best strategy (ln –32920). The comparison between the 4 × 4 nucleotide model (ln –32920) and the doublet model (ln –31968) for the paired nuclear rRNA sites suggests that the doublet model of nucleotide evolution was the best option for our data set.

Impact of missing data

The Bayesian analysis of the combined data sets containing taxa for which at least three of three (46 taxa), two of three (70 taxa) or one of three (83 taxa) genes were available demonstrated that the taxa set for which at least one of three genes were available contained 30% of nodes with significant support (< 0.95), whereas the taxa set for which at least two of three genes and that for which all genes were available produced 46% of nodes with significant support. Given the similarity of results between the data set containing at least two of three genes and that containing all three genes, it was decided that further phylogenetic analyses would be undertaken on taxa where at least two of the three genes had been sequenced. This allowed a much greater number of taxa to be included.

Table 4. Models chosen for data partitions by the Akaike information criterion in Modeltest (Posada and Crandall, 1998)

Partition	Model chosen
COI: first amino acid position	TRN + I + Γ
COI: second amino acid position	TVM + I + Γ
COI: third amino acid position	Reference number + I + Γ
COI: all	GTR + I + Γ
28S rRNA paired sites	GTR + I + Γ
28S rRNA unpaired sites	GTR + I + Γ
28S rRNA all	GTR + I + Γ
18S rRNA paired sites	TRN + I + Γ
18S rRNA unpaired sites	GTR + I + Γ
28S rRNA all	GTR + I + Γ

Phylogenetic analysis

The phylogenetic trees resulting from the MP and Bayesian analyses are shown in Figures 1, 2. Although there are differences between the two trees, none of the differences are supported by either significant posterior probabilities or high nonparametric bootstraps. The Platygastroidea is clearly supported as a monophyletic clade [1.00 poster probability (pp) and 60 bootstrap support (bs)], and the species included from the Platygastridae also form a strongly supported monophyletic clade (1.00 pp, 98 bs). The Scelionidae are not monophyletic; Bayesian analysis found two scelionids, *Archaeoteleia* and *Neuroscelio*, to be the sister group to the rest of the platygastroids, whereas two other scelionid genera, *Sceliomorpha* and *Sparasion*, effectively form an unresolved trichotomy with the platygastrids and the majority of the scelionids due to very low posterior probabilities (0.61). MP analysis found *Sparasion* to form a clade with *Archaeoteleia* and *Neuroscelio*, and resolution of this node could further render the scelionids polyphyletic.

The main Scelionidae clade (sans *Archaeoteleia*, *Neuroscelio*, *Sceliomorpha*, and *Sparasion*) is well supported (1.00 pp, 73 bs). Within this clade, the Bayesian analysis supports a division at the deeper nodes with *Neoscelio* + *Nyleta* (0.99 pp), and then *Probaryconus* + *Oethecoctonus* split from the main scelionid clade (0.99 pp). MP analysis also suggests that these taxa, along with *Aradophagus*, are at the base of the main scelionid clade; however, it does not suggest a sister relationship between *Neoscelio* and *Nyleta*. None of these nodes, with the exception of *Oethecoctonus* + *Probaryconus* demonstrate MP bootstrap support greater than 50. Relationships within the remainder of the scelionid clade are, to a large extent, unresolved. Although the subfamilies Telenominae (1.00 pp, 89 bs) and Teleasinae (0.99 pp, 93 bs) are both well supported, the relationships between these clades and amongst other scelionid groups are not supported. Other clades showing significant support include *Genatropis* sp. + *Caloteleia* sp. 1 (1.00 pp, 79 bs), and *Opisthacantha* sp. 2 + *Sceliacanthella* sp. (1.00 pp, 89 bs).

For the tribes of Scelionidae with more than one genus included, none are monophyletic, with the exception of Scelionini. However, monophyly of this tribe (*Acanthoscelio* + *Scelio*) is only found in the Bayesian analysis (Fig. 2) and does not receive significant support. Tribes that are not monophyletic are the Baeini (*sensu* Austin and Field, 1997) where

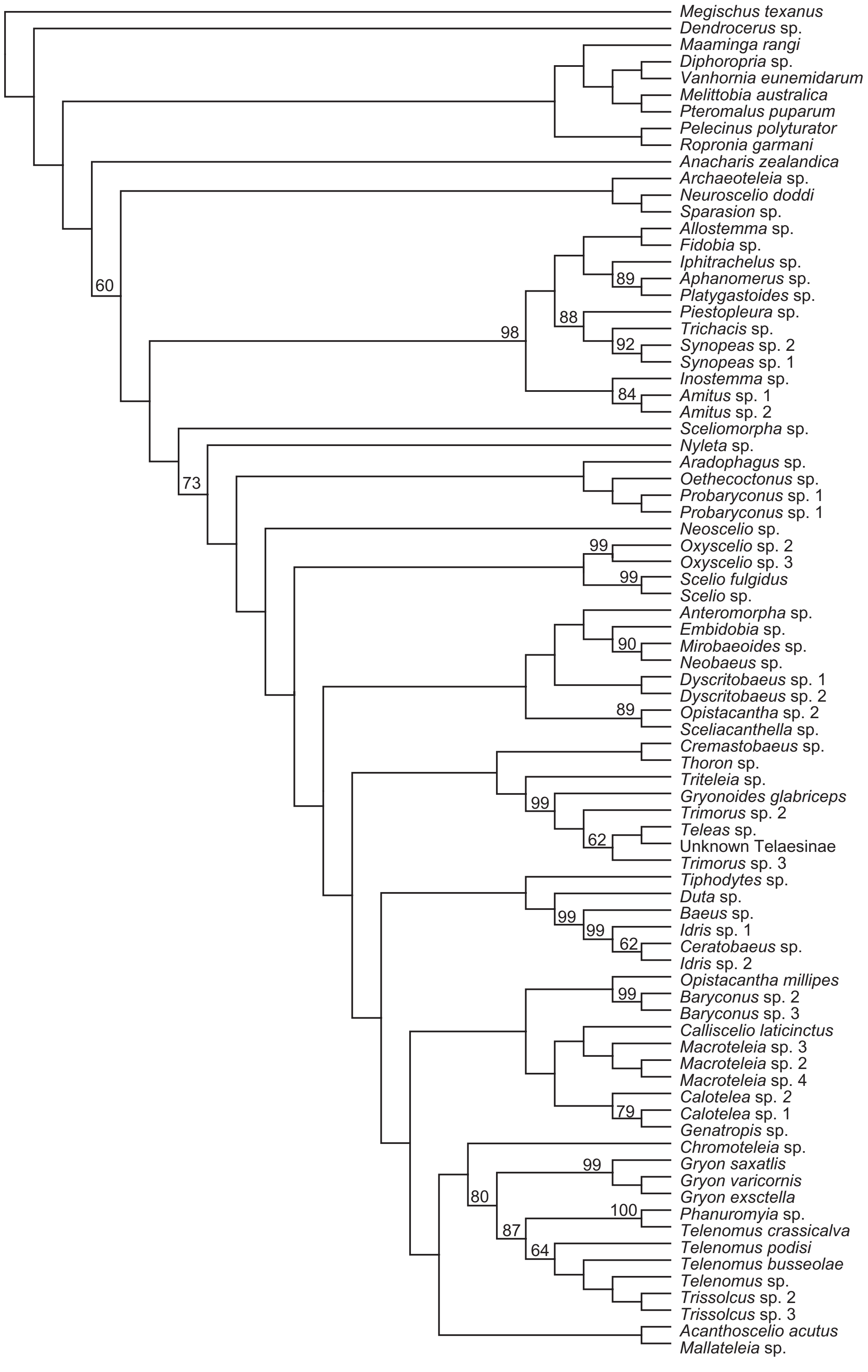

Figure 1. One of ten maximum parsimony trees resulting from analysis of combined *COI*, *28S rRNA*, and *18S rRNA* genes. Variation between the ten trees occurred only within well supported tip clades (e.g. between the three *Macroteleia* spp). Numbers on branches represent non-parametric bootstrap support.

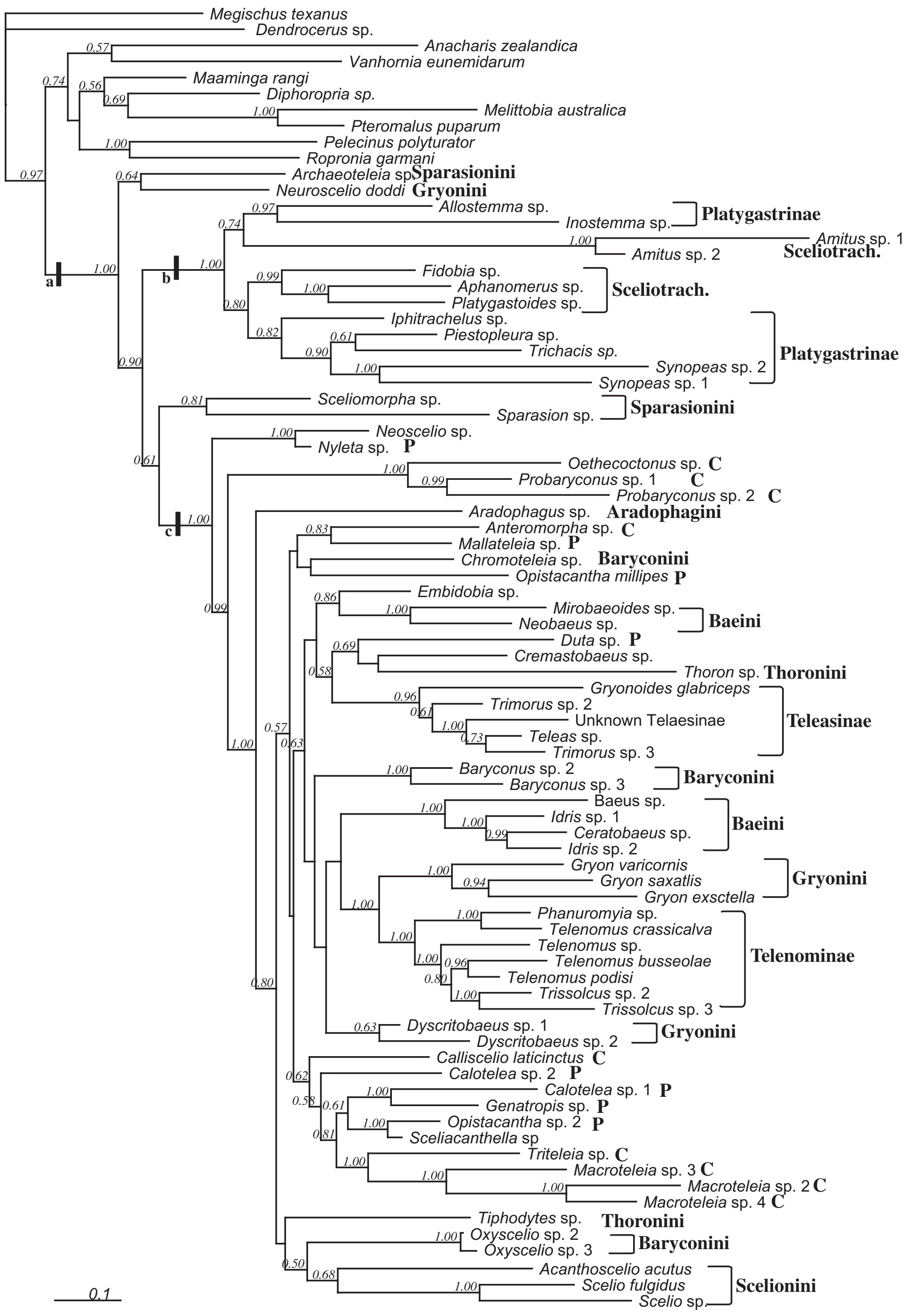

Figure 2. Bayesian phylogenetic analysis of combined *COI*, *28S rRNA*, and *18S rRNA* genes. Tree produced from 2×10^6 generations with sequence evolution models (Table 2) unlinked across partitions and the doublet model of evolution enforced for the paired rRNA sites. Numbers on branches indicate posterior probabilities, only branches receiving posterior probabilities greater than 0.50 are resolved. Dark bars on nodes: a, Platygastroidea; b, Platygastridae; c, well supported Scelionidae clade. Subfamily and tribal classifications are denoted after taxon names (P, Psilanteridini; C, Calliscelionini).

Neobaeus + *Mirobaeoides* (1.00 pp, 75 bs) are associated instead with *Embidobia* (not significant) and not closely affiliated with the monophyletic group of *Idris*, *Ceratobaeus* and *Baeus* (1.00 pp, 97 bs); and Gryonini, where *Gryon* is sister to the Telenominae (1.00 pp, 80 bs) and not affiliated with *Dyscritobaeus* and *Neuroscelio.* Calliscelionini and Psilanteridini (*sensu* Masner, 1976) are rampantly polyphyletic with genera belonging to these tribes being distributed throughout the main scelionid clade. Although few exemplars were included, Sparasionini, Baryconini and Thoronini (*sensu* Masner, 1976) are also not monophyletic. There are also a number of genera that are not monophyletic, most significantly *Opisthacantha* and *Caloteleia*, whereas, within the Telenominae, *Telenomus* is polyphyletic (significant pp), and within the Teleasinae, *Trimorus* is polyphyletic (significant pp) and *Teleas* is paraphyletic (not significant). Within the Platygastridae, neither of the two putative subfamilies, Platygastrinae or Sceliotrachelinae (*sensu* Masner and Huggert, 1989), is monophyletic.

Hypothesis tests

A number of *a posteriori*, alternative phylogenetic hypotheses containing topological constraints were tested against the Bayesian tree (Fig. 2) using the AU test. The subfamilies and tribes shown to be nonmonophyletic had their monophyly enforced to examine whether this created significantly worse representations of the data. Results of the AU topology tests (Table 5) demonstrate that trees constrained, to enforce Baeini, Baryconini, Gryonini, Psilanteridini and Calliscelionini are all significantly inferior ($P > 0.05$) to the optimal topology. All other groups tested demonstrated inferior likelihoods; however, none were statistically significant.

Ovipositor system

Scelionids that possess a '*Scelio*-type' ovipositor system do not form a monophyletic group and the AU test demonstrates that the constrained tree enforcing this monophyly is significantly inferior ($P > 0.05$). Ancestral state reconstruction of this character onto the Bayesian topology using the symmetrical likelihood model and the asymmetrical analysis produced similar results (Fig. 3; only the symmetrical model is shown). These analyses suggest that the *Ceratobaeus*-type ovipositor system is the ancestral state for the platygastroids as a whole, a result that is not surprising given that all platygastrids possess this system. However, one genus found in the deeper nodes, *Archaeoteleia*, has the *Scelio*-type ovipositor. Although species possessing the *Scelio*-type ovipositor system do not form a monophyletic group, it is the ancestral state for the main scelionid clade. The majority of species in this clade with a *Ceratobaeus*-type ovipositor system are monophyletic, and ancestral state reconstruction suggests that this system is the more derived in the main scelionid clade. Ancestral state reconstruction suggests that the *Ceratobaeus*-type ovipositor system has re-evolved independently in the main scelionid clade five times; four times by nonrelated species in the basal lineages and once by the majority of scelionids with this ovipositor system. Within this clade, only one species, *Duta*, possesses the *Scelio*-type system, suggesting that this trait has re-evolved once. It

Table 5. Results of AU tests (Shimodaira, 2002) for comparison between optimal Bayesian topology and alternate constrained topologies

Topology	Ti	AU	SE	PF
MrBayes tree	–37.3	0.912	–0.004	0.486
Monophyly of Thoronini	37.9	0.274	–0.012	0.115
Monophyly of Sceliotrachelinae	37.3	0.250	–0.010	0.725
Monophyly of Sparasionini	45.1	0.183	–0.013	0.306
Monophyly of Scelionini	51	0.105	–0.010	0.091
Monophyly of Platygastrinae	65.2	0.063	–0.010	0.316
Monophyly of Baeini*	72.8	0.035	–0.007	0.289
Monophyly of Baryconini*	263	0.003	–0.005	0.842
Monophyly of Psilanteridini*	180.1	0.003	–0.006	0.981
Monophyly of Gryonini*	149.1	0.002	–0.002	0.406
Monophyly of Calliscelionini*	334.1	0.001	0	1

For species included in each alternate topology, see Tables 1–3.
Ti, best log-likelihood score for data partition, or difference relative to it; AU, *P*-value from the approximately unbiased test; SE, standard error of AU; PF, *P*-value for the breakdown of asymptotic theory.
*Significant difference in log-likelihood scores at 0.05.

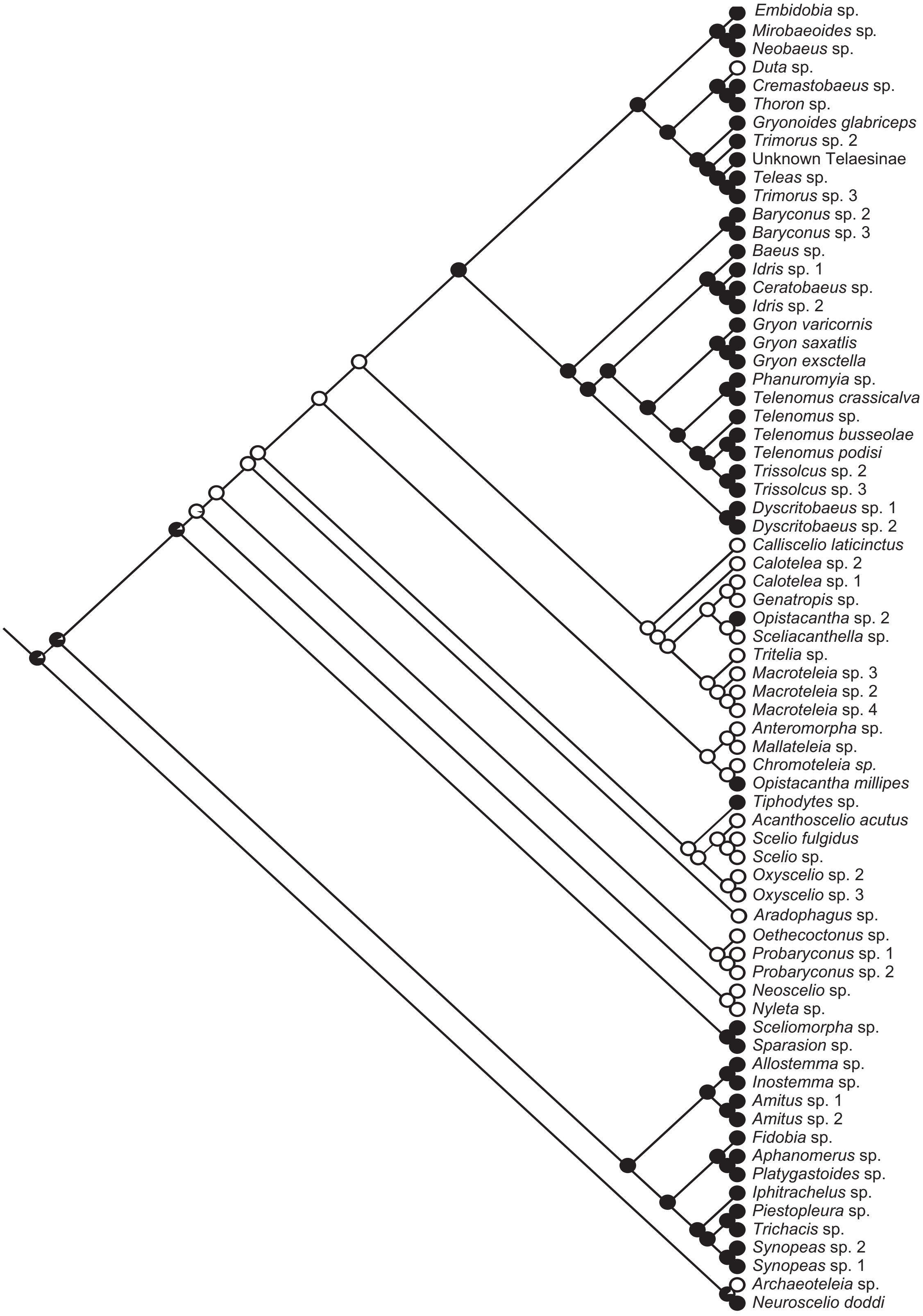

Figure 3. Maximum likelihood ancestral state reconstructions of ovipositor evolution building based on the Bayesian tree The example shown is using the symmetrical (equal rates of loss and gain) likelihood models. Pie diagrams (circles) at nodes indicate the likelihood of the *Scelio*-type (white) or *Ceratobaeus*-type ovipositor (black).

should be considered, however, that these reconstructions are tentative due to many nodes having weak or nonsignificant support. Ancestral state reconstructions on the MP tree (not shown), also indicate that the *Ceratobaeus*-type ovipositor system is the ancestral state for the platygastroids as a whole and that the *Scelio*-type system is ancestral for the main scelionid clade.

DISCUSSION

Platygastroidea classification

Within the Platygastroidea, the Platygastridae were found to be clearly monophyletic, a result that was not unexpected due to the available morphological evidence supporting this (Masner and Huggert, 1989; Austin and Field, 1997; Austin *et al*., 2005). Scelionidae, however, was not monophyletic, the Bayesian analysis resolving *Neuroscelio* and *Archaeoteleia* outside of the main scelionid clade, whereas the MP analysis included *Sparasion* in this group and both analyses suggested that the position of *Sceliomorpha* within the main scelionid clade was not supported. Interestingly, *Archaeoteleia*, *Sparasion*, and *Sceliomorpha* are all recognized as possessing primitive characteristics (Masner, 1976), and previous molecular studies have shown *Sparasion* to be sister to the remaining exemplars used for the superfamily (Dowton and Austin, 2001). *Neuroscelio* was putatively assigned to the Gryonini by Galloway *et al*. (1992); however, the genus is clearly misplaced within this tribe.

The remainder of the Scelionidae formed a well-supported monophyletic group. Apart from the basal nodes, the majority of relationships within the scelionids are not well supported. The subfamily Scelioninae is unsurprisingly not monophyletic, as postulated previously (Austin and Field, 1997; Austin *et al*., 2005). However, both the Telenominae and the Teleasinae do form strongly supported clades.

At tribal level, only the Scelionini *s.s.* is resolved as monophyletic by Bayesian analysis but the other tribes with multiple representatives are not monophyletic. This is not surprising for the large tribes Calliscelionini and Psilanteridini because both contain species with the *Scelio*-type and *Ceratobaeus*-type ovipositor systems. Species belonging to these tribes with a *Ceratobaeus*-type ovipositor were considered as misplaced by Austin and Field (1997); however, even if limited to species that possess one or the other ovipositor system, neither tribe is monophyletic. The nonmonophyly of the Baeini recently has been proposed by Carey *et al*. (2006) and is confirmed here within a broader representation of scelionids. Both members of the tribe Thoronini, the members of which are all thought to parasitize eggs of aquatic Hemiptera, included in this study, *Thoron* and *Tiphodytes*, exhibit the *Ceratobaeus*-type ovipositor but do not appear to be closely allied, confirming that the smooth body and long stiff bristles of Thoronini may be convergent adaptations to underwater behaviour (Johnson and Masner, 2004). The Gryonini (*sensu* Masner, 1976) are also polyphyletic. Although the group has been assumed to be monophyletic based in part on their host biology (i.e. parasitiods of Heteroptera), it is not defined by any obvious synapomorphy, and several genera including two that were sequenced in this study, namely *Dyscritobaeus* and *Neuroscelio*, do not have any recorded host data.

Given the low levels of support for many of the deeper nodes, and that many genera were not included in the study, there is no obvious subfamily and tribal classification that could be proposed based on the results obtained here. Strong support is demonstrated for a close relationship between Telenominae and *Gryon*. This close association may be due to similar host groups because a number of Telenominae also parasitize Heteroptera. There is a weakly supported association between *Embidobia* and two genera previously assigned to the Baeini, *Mirobaeoides* and *Neobaeus*, as found by Carey *et al*. (2006), lending support to the notion that this group shares a common evolutionary history of silk searching (Galloway and Austin, 1984). However this appears to have evolved independently in the remainder of the baeines (Carey *et al*., 2006).

Within the platygastrids, neither of the two currently recognized subfamilies, Sceliotrachelinae and Platygastrinae (*sensu* Masner and Huggert, 1989) are monophyletic. This result is somewhat unexpected for the Platygastrinae because this group shares a common host group (Cecidomyiidae) (Masner and Huggert, 1989; Austin and Field, 1997). The taxonomic coverage of the platygastrids was not sufficient to satisfactorily test the genus group relationships proposed by Masner and Huggert (1989); however, none of these groups were resolved as monophyletic.

The recognition of monophyletic subfamilies and tribes within the Platygastroidea has been problematic due to much of the classification being reliant on reductional characters, symplesiomorphies, or simply similar body forms (Austin *et al*., 2005). This has hampered the recognition of natural groups and, given the results of this study, many of these reductional characters appear to have evolved convergently. Further illustrating the problems associated with the current classification is the nonmonophyly of a number of genera. Although several genera have been previously suggested as nonmonophyletic (Johnson, 1984; Austin and Field, 1997; Iqbal and Austin, 2000), the large disparity between some of these taxa, for example between the two *Opisthacan-*

tha species included, emphatically highlights the problems inherent in developing a robust classification for the superfamily.

EVOLUTION OF THE OVIPOSITOR SYSTEM

Given that the ovipositor system was thought to be less prone to reductions in complexity, it was proposed as a significantly phylogenetically informative suite of characters (Field and Austin, 1994; Austin and Field, 1997). On this basis, Austin and Field (1997) proposed that the *Scelio*-type system was apomorphic within the superfamily and supports a monophyletic clade (Scelionini *s.l.*) comprising several tribes and a large number of genera.

Ancestral character state reconstructions (Fig. 3) strongly suggest that the *Scelio*-type system does not define a monophyletic group, and that the *Ceratobaeus*-type system is the ancestral trait in the Platygastroidea. The results also suggest a single origin for the majority of species exhibiting the *Scelio*-type system and, more significantly, imply that the ancestral state for the main scelionid clade is the *Scelio*-type ovipositor. In fact, ancestral state reconstruction on the Bayesian tree suggests that instead of the *Scelio*-type system being apomorphic, the *Ceratobaeus*-type system is more derived within this clade, and the trait has evolved independently from the Platygastridae and deeper platygastroid lineages. Thus, this system appears to have been lost early in the Scelionidae and then regained in the more derived scelionid lineages.

The apparent independent origin of the *Ceratobaeus*-type system within the Platygastroidea is unexpected and, at first, appears to contradict Dollo's Law (i.e. complex ancestral states, if lost, cannot be reacquired; Gould, 1970). However, there are two possible hypotheses that might explain the presence of the ancestral ovipositor system in independent lineages. First, the genes for the ancestral condition may have remained present in all lineages and simply remained dormant. This is known to have occurred in some organisms but truly dormant genes are not thought to survive for more than approximately 10 million years at most (Marshall, Raff & Raff, 1994; Lynch and Conery, 2000). Furthermore, although the timing of the origins of the *Ceratobaeus*- and *Scelio*-type systems is unknown, many scelionid fossils that are congeneric with extant taxa would indicate that these splits occurred much further back in the evolution of the superfamily. An alternative hypothesis is heterotopy where the ancestral genes for the ovipositor system are present in taxa with the *Scelio*-type but that they code for other possibly related traits (Pagel, 2004). This is postulated as an explanation for the independent gains and losses of wings in the Phasmatodea (Whiting, Bradler & Maxwell, 2003). The fact that the apodemes in the *Ceratobaeus*-type system are retained in some genera with a *Scelio*-type ovipositor system, and that they probably serve a different function by strengthening the telescopic tube, points to heterotopy as a possible explanation.

There is also no doubt that a detailed re-examination of the ovipositor systems across the superfamily would be worthwhile. In many respects, the study by Austin and Field (1997) only scratched the surface in documenting variation within the ovipositor system of both the *Ceratobaeus*- and *Scelio*-type systems. Their study revealed significant morphological differences, particularly within the unusual ovipositor systems of numerous genera, suggesting that such a re-examination may be profitable phylogenetically.

HOST UTILIZATION IN THE PLATYGASTROIDEA

Platygastroids have been proposed to show a clear pattern of host group specificity and a number of subfamilies and tribes are associated with a single host group (Austin and Field, 1997; Austin *et al.*, 2005). However, given that the vast majority of subfamilies and tribes are not monophyletic, based on the result presented here, the use of host associations for inferring evolutionary relationships appears to be ineffective. Instead, there is evidence for significant convergent evolution onto similar hosts. The best examples of this are the nonmonophyly of Baeini, which requires there to have been at least two origins of parasitism of spider eggs, a significant switch from insect eggs, and the nonmonophyly of the Thoronini, which entails multiple evolution into exploiting the eggs of aquatic insect.

The main limitation to understanding how host associations have evolved across the superfamily is a lack of detailed and corroborated host data for many groups. For example, the Gryonini are considered to parasitize the eggs of various Heteroptera; however, in the present study, only *Gryon* has had host data recorded. There is also a substantial bias in host information across the superfamily, particularly against hosts associated with cryptobiotic habitats such as rotting wood, litter, and soil, in which host eggs are difficult to locate (Austin *et al.*, 2005). In addition, there is a bias against hosts that lay their eggs singly as opposed to in egg masses in which at least a couple of emerging hosts may allow for host identification. Alternatively, hosts that lay their eggs in more accessible places, or represent economically or ecologically important groups such as Heteroptera, Orthoptera, and Lepidoptera, have more comprehensive host information. Further records of host associations may reveal more monophyletic lineages based on hosts and instances of host switching.

Platygastroid evolution

The lack of resolution in the deeper nodes of the scelionid clade may be due to the nature of platygastrid evolutionary history. Radiation into arthropod eggs as hosts undoubtedly opened up a vast spectrum of possibilities for early diversification of the group. These opportunities could have facilitated rapid diversification in the early lineages because niche partitioning and host-directed adaptation could have instigated morphological and behavioural differentiation, leading to reproductive isolation and speciation (Quicke, 1997). If this did occur, the phylogenetic legacy of this period would be short branch lengths that are difficult to resolve, even with large amounts of data. An unrooted Bayesian tree shows a star-like pattern in the main scelionid clade (Fig. 4), suggesting a period of rapid radiation early on in the scelionids, whereas the platygastrids show a more tree-like phylogeny. There is strong evidence for an increase in the rate of sequence divergence across all of the parasitic Hymenoptera (Mardulyn and Whitfield, 1999), and this could have been especially pronounced in the early evolution of the Scelionidae. This situation has been observed in the braconid subfamily Microgastrinae, associated with adaptive radiation into lepidopteran larvae as hosts (Mardulyn and Whitfield, 1999).

There are other possible explanations for poor resolution, such as insufficient taxon sampling or misleading evolution of individual genes (Brocchieri, 2001; Sanderson and Shaffer, 2002; Zwickl and Hillis, 2002; Reyes *et al.*, 2004). Given that only a small proportion of genera were sequenced for this study, a more comprehensive sampling of taxa may introduce more 'shallow' lineages, breaking down the star-like phylogeny. The platygastrids, however, form a much more resolved phylogeny. Consequently it appears that the scelionids have undergone a rapid burst of diversification, whereas the platygastrids have not.

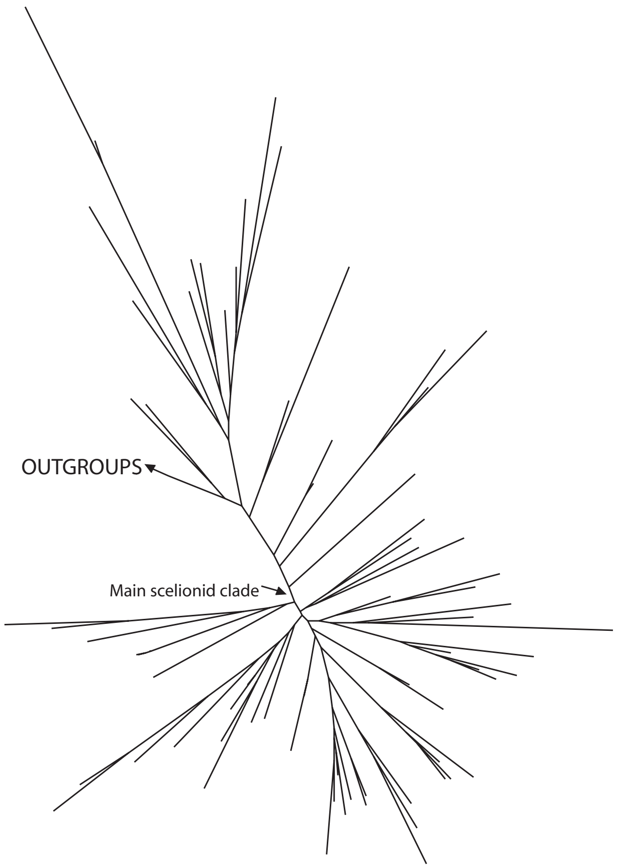

Figure 4. Unrooted Bayesian phylogenetic tree constructed using the same methodology as in Fig. 1.

ACKNOWLEDGEMENTS

We wish to thank Lubomir Masner and Norm Johnson for providing us with valuable material for this project, and for their discussions about platygastroid systematics over many years. Thanks to Lars Vilhelmsen for his comments on a penultimate draft of the manuscript. The Australian Research Council, the Australian Biological Resources Study, and The University of Adelaide provided financial support for the project.

REFERENCES

Austin AD. 1983. Morphology and mechanics of the ovipositor system of *Ceratobaeus* Ashmead (Hymenoptera: Scelionidae) and related genera. *International Journal of Insect Morphology and Embryology* **12:** 139–155.

Austin AD, Field SA. 1997. The ovipositor system of scelionid and platygastrid wasps (Hymenoptera: Platygastroidea): comparative morphology and phylogenetic implications. *Invertebrate Taxonomy* **11:** 1–87.

Austin AD, Johnson NF, Dowton M. 2005. Systematics, evolution and biology of scelionid and platygastrid wasps. *Annual Review of Entomology* **50:** 553–582.

Bin F. 1981. Definition of female antennal clava based on its plate sensilla in Hymenoptera Scelionidae Telenominae. *Redia* **64:** 245–261.

Bin F, Vinson SB. 1986. Morphology of the antennal sexgland in male *Trissolcus basalis* (Woll) (Hymenoptera: Scelionidae), an egg parasitoid of the green stink bug, *Nezara viridula* (Hemiptera: Pentatomidae). *International Journal of Insect Morphology and Embryology* **15:** 129–138.

Brandley MC, Schmitz A, Reeder TW. 2005. Partitioned Bayesian analyses, partition choice, and the phylogenetic relationships of scincid lizards. *Systematic Biology* **54:** 373–390.

Brocchieri L. 2001. Phylogenetic inferences from molecular sequences: review and critique. *Theoretical Population Biology* **59:** 27–40.

Carey D, Murphy NP, Austin AD. 2006. Molecular phylogenetics and the evolution of wing reduction in a tribe of parasitoid wasps (Hymenoptera : Scelionidae : Baeini) *Invertebrate Systematics* **20:** 489–501.

Castro L, Dowton M. 2006. Molecular analyses of the Apocrita (Insecta: Hymenoptera) suggest that the Chalcidoidea are sister to the diaprioid complex. *Invertebrate Systematics* **20:** 603–614.

Dowton M, Austin AD. 2001. Simultaneous analysis of 16S, 28S, COI and morphology in the Hymenoptera: Apocrita – evolutionary transitions among parasitic wasps. *Biological Journal of the Linnean Society* **74:** 87–111.

Dowton M, Austin AD. 2002. Increased congruence does not necessarily indicate increased phylogenetic accuracy – the behavior of the incongruence length difference test in mixed-model analyses. *Systematic Biology* **5:** 19–31.

Field SA, Austin AD. 1994. Anatomy and mechanics of the telescopic ovipositor system of *Scelio Latreille* (Hymenoptera: Scelionidae) and related genera. *International Journal of Insect Morphology and Embryology* **23:** 135–158.

Galloway ID, Austin AD. 1984. Revision of the Scelioninae (Hymenoptera: Scelionidae) in Australia. *Australian Journal of Zoology Supplementary Series* **99:** 1–1138.

Galloway ID, Austin AD, Masner L. 1992. A revision of the enigmatic genus *Neuroscelio Dodd*, a group of primitive scelionids from Australia (Hymenoptera: Scelionidae), with a discussion of the ovipositor system of the tribe Gryonini. *Invertebrate Taxonomy* **6:** 523–545.

Gatesy J, Desalle R, Wheeler W. 1993. Alignment-ambiguous nucleotide sites and exclusion of systematic data. *Molecular Phylogenetics and Evolution* **2:** 152–157.

Gillespie JJ. 2004. Characterizing regions of ambiguous alignment caused by the expansion and contraction of hairpin-stem loops in ribosomal RNA molecules. *Molecular Phylogenetics and Evolution* **33:** 936–943.

Gillespie JJ, Cannone JJ, Gutell RR, Cognato AI. 2004. A secondary structural model of the 28S rRNA expansion segments D2 and D3 from rootworms and related leaf beetles (Coleoptera: Chrysomelidae; Galerucinae). *Insect Molecular Biology* **13:** 495–518.

Gillespie JJ, Yoder MJ, Wharton RA. 2005. Predicted secondary structures for 28S and 18S rRNA from *Ichneumonoidea* (Insecta: Hymenoptera: Apocrita): impact on sequence alignment and phylogeny estimation. *Journal of Molecular Evolution* **61:** 114–137.

Gould SJ. 1970. Dollo's law: irreversibility and the status of evolutionary laws. *Journal of the History of Biology* **3:** 189–212.

Huelsenbeck JP, Ronquist F. 2001. MrBayes: Bayesian inference of phylogenetic trees. *Bioinformatics* **17:** 754–755.

Hughes J, Vogler AP. 2004. The phylogeny of acorn weevils (genus *Curculio*) from mitochondrial and nuclear DNA sequences: the problem of incomplete data. *Molecular Phylogenetics and Evolution* **32:** 601–615.

Iqbal M, Austin AD. 2000. A preliminary phylogeny for the *Baeini* (Hymenoptera: Scelionidae): endoparasitoids of spider eggs. In: Austin AD, Dowton M, eds. *The Hymenoptera: evolution, biodiversity and biological control*. Melbourne: CSIRO Publishing, 178–191.

Isidoro N, Bin F, Colazza S, Vinson B. 1996. Morphology of the antennal gustatory sensilla and glands in some parasitic Hymenoptera with hypothesis on their role in sex and host recognition. *Journal of Hymenoptera Research* **5:** 206–239.

Johnson NF. 1984. Systematics of *Nearctic Telenomus*: classification and revisions of the podisi and phymatae species groups (Hymenoptera: Scelionidae). *Bulletin of the Ohio Biological Survey* **6:** 1–113.

Johnson NF. 1992. Catalogue of world Proctotrupoidea excluding Platygastridae. *Memoirs of the American Entomological Institute* **51:** 1–825.

Johnson NF, Masner L. 2004. The genus *Thoron Haliday* (Hymenoptera: Scelionidae), egg-parasitoids of waterscorpions (Hemiptera: Nepidae) with a key to world species. *American Muesum Novitates* **3452:** 1–16.

Kjer KM. 2004. Aligned 18S and insect phylogeny. *Systematic Biology* **53:** 506–514.

Lockhart PJ, Steel MA, Hendy MD, Penny D. 1994. Recovering evolutionary trees under a more realistic model of sequence evolution. *Molecular Biology and Evolution* **11:** 605–612.

Lynch M, Conery JS. 2000. The evolutionary fate and consequences of duplicate genes. *Science* **290:** 1151–1155.

Maddison WP, Maddison DR. 2003a. *Mesquite, a modular system for evolutionary analysis*, Version 1.0. Available at: http://mesquiteproject.org.

Maddison WP, Maddison DR. 2003b. *Stoch.Char: a package of Mesquite modules for stochastic models of character evolution*, Version 1.0. available at: http://mesquiteproject.org.

Mardulyn P, Whitfield JB. 1999. Phylogenetic signal in the COI, 16S and 28S genes for inferring relationships among genera of Microgastrinae (Hymenoptera: Braconidae); evidence of a high diversification rate in this group of parasitoids. *Molecular Phylogenetics and Evolution* **12:** 282–294.

Marshall CR, Raff EC, Raff RA. 1994. Dollo's law and the death and resurrection of genes. *Proceeding of the National Academy of Science of the United States of America* **91:** 12283–12287.

Masner L. 1958. An interesting new genus of Scelionidae from S.W. Africa (Hymenoptera: Proctotrupoidea). *Proceedings of the Entomological Society, London B* **27:** 101–104.

Masner L. 1968. A new genus of Scelionidae (Hymenoptera) with austral disjunct distribution. *New Zealand Journal of Science* **11:** 652–663.

Masner L. 1976. Revisionary notes and keys to world genera of Scelionidae (Hymenoptera: Proctotrupoidea). *Memoirs of the Entomological Society of Canada* **97:** 1–87.

Masner L. 1993. Superfamily Platygastroidea. In: Goulet H, Huber JT, eds. *Hymenoptera of the world: an identification guide to families*. Ottawa: Agriculture Canada. 558–565.

Masner L. 1995. The proctotrupoid families. In: Hanson P, Gauld ID, eds. *Hymenoptera of Costa Rica*. Oxford: Oxford University Press, 209–245.

Masner L, Huggert L. 1989. World review and keys to genera of the subfamily Inostemmatine with reassignment of

the taxa to the Platygastrinae and Sceliotrachelinae (Hymenoptera: Platygastridae). *Memoirs of the Entomological Society of Canada* **147:** 1–214.

Mathews DH, Sabina J, Zuker M, Turner DH. 1999. Expanded sequence dependence of thermodynamic parameters improves prediction of RNA secondary structure. *Journal of Molecular Biology* **288:** 911–940.

Ogden TH, Whiting MF, Wheeler WC. 2005. Poor taxon sampling, poor character sampling, and non-repeatable analyses of a contrived dataset do not provide a more credible estimate of insect phylogeny: a reply to Kjer. *Cladistics* **21:** 295–302.

Pagel M. 1999. The maximum likelihood approach to reconstructing ancestral chracter states of discrete characters on phylogenies. *Systematic Biology* **48:** 612–622.

Pagel M. 2004. Limpets break Dollo's law. *Trends in Ecology and Evolution* **19:** 278–280.

Posada D, Crandall KA. 1998. MODELTEST: testing the model of DNA substitution. *Bioinformatics* **14:** 817–818.

Quicke DLJ. 1997. *Parasitic wasps*. London: Chapman & Hall.

Reeder TW. 2003. A phylogeny of the Australian Sphenomorphus group (Scincidae: Squamata) and the phylogenetic placement of the crocodile skinks (Tribolonotus): Bayesian approaches to assessing congruence and obtaining confidence in maximum likelihood inferred relationships. *Molecular Phylogenetics and Evolution* **27:** 384–397.

Reyes A, Gissi C, Catzeflis F, Nevo E, Pesole G, Saccone C. 2004. Congruent mammalian trees from mitochondrial and nuclear genes using Bayesian methods. *Molecular Biology and Evolution* **21:** 397–403.

Ronquist F, Rasnitsy AP, Roy A, Eriksson K, Lindgren M. 1999. Phylogeny of the Hymenoptera: a cladistic reanalysis of Rasnitsyn's (1988) data. *Zoologica Scripta* **28:** 13–50.

Sanderson MJ, Shaffer HB. 2002. Troubleshooting molecular phylogenetic analyses. *Annual Review of Ecology and Systematics* **33:** 49–72.

Schoniger M, von Haeseler A. 1994. A stochastic model and the evolution of autocorrelated DNA sequences. *Molecular Phylogenetics and Evolution* **3:** 240–247.

Sharkey MJ, Roy A. 2002. Phylogeny of the Hymenoptera: a reanalysis of the Ronquist *et al.* (1999) reanalysis, with an emphasis on wing venation and apocritan relationships. *Zoologica Scripta* **31:** 57–66.

Shimodaira H. 2002. An approximately unbiased test of phylogenetic tree selection. *Systematic Biology* **51:** 492–508.

Simon C, Frati F, Beckenbach A, Crespi B, Liu H, Flook P. 1994. Evolution, weighting and phylogenetic utility of mitochondrial gene sequences and a compilation of conserved polymerase chain reaction primers. *Annals of the Entomological Society of America* **87:** 651–701.

Swofford DL. 2000. *PAUP* phylogenetic analysis using parsimony (* and other methods)*, Version 4. Sunderland, MA: Sinauer.

Thompson JD, Gibson TJ, Plewniak F, Jeanmougin F, Higgens DG. 1997. The ClustalX windows interface: flexible strategies for multiple sequence alignment aided by quality analysis tools. *Nucleic Acids Research* **24:** 4876–4882.

Whiting MF, Bradler S, Maxwell T. 2003. Loss and recovery of wings in stick insects. *Nature* **241:** 264–267.

Whiting MF, Carpenter JM, Wheeler QD, Wheeler WC. 1997. The Strepsiptera problem: phylogeny of the holometabolous insect orders inferred from 18S and 28S ribosomal DNA sequences and morphology. *Systematic Biology* **46:** 1–68.

Wiens JJ. 1998a. Combining data with different phylogenetic histories. *Systematic Biology* **47:** 568–581.

Wiens JJ. 1998b. Does adding characters with missing data increase or decrease phylogenetic accuracy. *Systematic Biology* **47:** 625–640.

Zuker M. 2003. Mfold web server for nucleic acid folding and hybridization prediction. *Nucleic Acids Research* **31:** 3406–3415.

Zwickl DJ, Hillis DM. 2002. Increased taxon sampling greatly reduces phylogenetic error. *Systematic Biology* **51:** 588–598.
