# Supplementary material for: Spectral measure of color variation of black-orange-black (BOB) pattern in small parasitoid wasps (Hymenoptera: Scelionidae), a statistical approach
Source: PLoS One. 2019 Oct 24;14(10):e0218061. doi: 10.1371/journal.pone.0218061 (PMC6812806; doi:10.1371/journal.pone.0218061)
Supplement: Attachment — Submitted filename: review.pdf [file pone.0218061.s011.pdf]

This manuscript takes a quantitative approach to variation in animal color pattern. I have not found any issues with the data presented, but the manuscript needs intensive revision to maximize its effectiveness. A few additional, small analyses may be necessary to account for phylogeny.

In its current form the manuscript is very unclear, in a way that will greatly diminish its impact. The manuscript is also rather sloppy (for example, many citations are needed).

I have a strong background in the evolution of color pattern, a weaker background in the ecology of color pattern, and no background whatsoever in the methods used by these authors. In this sense, I am perhaps the target audience for this manuscript. I am not qualified to comment on the measurements that the authors made or on the quantitative/statistical methods used to analyze the data. Instead, my comments are geared toward general cohesion, biological appropriateness, and accessibility for organismal biologists. I would be very glad to review a revised version of this manuscript.

1 Cohesion

1.1 The point of the article

I cannot tell what this article is really about. At different points while reading the article, I wondered whether any of these may be the main point:

- Is this a case study, meant to show that FDA is better than univariate analysis for quantifying differences in biological color pattern?
- Is this a case study, meant to show that more rigorous measurements can and should be used when quantifying differences in biological color pattern?
- Is the point of this study that colors that appear very similar from the point of view of a human observer can in fact be significantly different from each other?

1.2 The motivation for the article

- Why did the authors choose this family of wasps? Why didn't they make these measurements with other parasitic wasps? Or with flies? Or with flowers?
- What is the additional benefit of analyzing the orange colors, instead of only analyzing the black colors?
- How do the authors hope that this article will influence their colleagues' questions/methods in future studies?

None of these questions need to be answered explicitly, but it should be possible for me to confidently guess at the answers after having read the manuscript.

1.3 Organization

The abstract doesn't explain the motivation for the study and doesn't communicate the study's significance. I recommend that the authors follow this guide, which I have found helpful for many years: http://www.easterbrook.ca/steve/2010/01/how-to-write-a-scientific-abstract-in-six-easy-steps/

PLOS ONE allows a Conclusions section at the end of the manuscript, and this is something that would be very helpful here. I recommend that the authors write out individual sentences containing each of their main conclusions (significant differences in color, the superiority of FDA over univariate analyses, etc.), arrange those sentences from most to least important starting with the main point of the paper, and then turn their list of sentences into a cohesive paragraph by adding transition phrases.

1.4 Clarity

- The Introduction, Methods, and Discussion center on how wonderful FDA is, but neither the phrase 'Functional Data Analysis' nor the abbreviation 'FDA' appear in the Results. If you want to convince people to use a certain method, you must make the results of that method clear.
- Abstract. 'describe and test the color differences between genera' What are these 'color differences'? If a color is found to be 'different' between two genera, what does that mean?
- Line 157. Which 'means' are the authors talking about? (This question is answered a few lines down, in the bullet points, but the reader shouldn't have to wonder as they finish this paragraph.)
- Line 223. 'independence between spots' Independence in what sense—independence between the black and orange spots on the same specimen? Between different specimens belonging to the same genus?
- Line 224-6. 'there is statistical evidence of a difference between mean reflectance of color for at least one genus and zero, controlling by specimen and spot.' This is not entirely clear. Do the authors mean that, in at least one genus examined, the mean reflectance of the orange spot is significantly different from the mean reflectance of the black spot?
- Line 247. 'the differences due to spots' What does this mean?
- Line 259-61. 'In contrast, the distance between Acanthoscelio (AC) and Acanthoscelio (AC) curves for the same color (in vermilion) clearly include the threshold and can be taken as not significantly different.' The curves being compared are individual curves from different specimens, right?

2 Biological appropriateness

2.1 Accounting for phylogeny

Some of the genera examined here are more closely related to each other than others, and this must be accounted for. I have attached three papers that give an overview of scelionid phylogeny/systematics. Page 662 of Murphy et al. 2007 (attached), which the authors cited once in the Discussion, contains a phylogeny that includes various genera examined in this study. Even if it is not possible to implement existing phylogenetic comparative methods in this manuscript, the authors should discuss whether the genera included in the Murray phylogeny that are most closely related to each other are most similar in terms of their color.

So far the authors do this for only one pair of genera ('the orange color of Macroteleia and Sceliomorpha group together for the green and red components (Fig. 7B) despite these genera not being closely related', lines 360-2). This needs to happen in a comprehensive, deliberate manner.

2.2 Taxonomy

When a genus or species is mentioned for the first time, its name must be followed by its taxonomic authority: the author(s) who described the genus/species. For example, *Acanthoscelio* is *Acanthoscelio* Ashmead.

Also, the two-letter abbreviations for the generic names just add a layer of unnecessary information to the manuscript. These abbreviations should be removed, and replaced with the full names of the genera.

3 Accessibility for organismal biologists

- Line 91. Explain what the CIE color space is and why it was used here.
- Line 102-3. Explain the biological meaning of delta-E and delta-P.
- Line 138. Explain the biological meaning of the 'response variable'.
- Line 239-40. Restate the biological meaning of 'the overall average change in all comparisons'.
- Line 243. Restate the biological meaning of delta-E when it is first used in the Results.

4 Line-by-line comments

- Line 2-11. The first two paragraphs of the Introduction say the same thing; one of them should be deleted. Also, the biological statements here ('These markings contribute to important behavioral, neurological, physiological, visual, morphological and developmental traits') are too vague/general.
- Line 22-5. 'The wide distribution of the BOB pattern in Hymenoptera suggests that the coloration arose due to changes in shared metabolic or genetic routes as reported in other taxa, and strongly suggests that it has some biological function [10].' Not necessarily, at least according to the information the authors have provided here. This color pattern could be ancestral (and not serve any biological function) in the hymenopteran clade in question, with widespread, convergent losses.
- Line 26. 'It has been reported that most animals perceive color differently than humans' Differently in what way?
- Line 27. 'which has created a new surge of interest' Citations needed to demonstrate that there is a 'surge'.
- Line 39-40. What data were fed into the PCA?
- Line 40. 'while recognizing its limitations' What are these 'limitations'?
- Line 41-42. 'Functional data analysis (FDA) is a powerful method to study spectrally measured data' Powerful how? How does it work, how is it different/better than PCA?
- Line 67-8. 'They were found and caught with entomological nets over a period of 12 months.' This raises the question of whether there are seasonal changes in color pattern.
- Line 68. Evaniidae is a family, not a genus.
- Line 78-9. 'A previous study demonstrated that males and females do not differ in color' This finding holds across a large clade that includes Scelionidae and also Evaniidae?
- Line 85-86. 'For each specimen three samples were taken, from the mesosoma (orange) and from the metasoma (black).' Three samples were taken from the mesosoma and three were taken from the metasoma? This sentence is unclear.
- Line 88-89. 'Fig 2 shows the spectral reflectance of the eight genera for the black and orange segments together with the control genus.' This sentence belongs in the Results, not Methods. Also in what sense is the evaniid a 'control'? This seems like the wrong word to use.
- Line 109. Spell out the whole term instead of using the abbreviation 'MSP'—this abbreviation is not used frequently enough to justify making the reader memorize it.
- Line 164. Define 'FANOVA'. I suspect the authors should cite its origination.
- Line 165-7. 'A statistical test was performed to interpret the contrast results for the mean difference curves.' Which statistical test?
- Line 171-2. 'Here also, specimen and spot were controlled.' This is unclear.
- Line 173-4. 'the usual univariate practice' The authors imply that the univariate method is commonly used, but I don't recall seeing any citations that show this to be the case.
- Line 174-5. 'to highlight the importance of taking into account all the information from the difference curves.' It's disconcerting to see the authors state, in the Methods, that they designed their study based on the assumption that one analysis will perform significantly better than another.
- Line 176. It's odd to see the same equation used for the univariate and functional responses, since each response was analyzed with a different kind of ANOVA and the functional response was analyzed with a metric computed with a special R package.

- Line 178. What is the 'p =' doing in there? Why not just write 'i = 1, 2, ... , 8'?
- Line 178. Change 'j = 1, 2, .., r = 4 ' to 'j = 1, 2, 3, 4'.
- Line 197. I recommend that the authors cite the R package used to make their figures.
- Line 205. The caption for Figure 2 is not provided anywhere.
- Line 211-2. 'Moreover, the effect of topography of the cuticle and homogeneity of the pigment distribution can partly explain the data dispersion in some of the genera.' The authors need to substantiate this statement with a figure, data, something.
- Line 213. 'The difference reflectance of the curves' I think this is gramatically incorrect.
- Line 216-7. 'in the range between 550 nm and 750 nm, and the region between 750 nm and 900 nm' Why not change this to 'in the range between 550 nm and 900 nm'? Also the measurements go above 900 nm.
- Figure 3. There is so much overlap between the different ribbons that it is often impossible to distinguish them. I suggest that the authors use a thick line for the mean value and thinner lines to mark the upper and lower extremes of each ribbon, leaving the interior blank (without any fill) so that it's easier to distinguish the ribbons.
- Line 220-1 (Figure 3 caption). What does it mean if the curve is below the black line versus above it?
- Line 222-3. 'The best fit in all cases was from the models that assume independence between spots, so linear models were used subsequently.' I'm not sure exactly what mean by this (as outlined in an above section), but the genera examined here are not statistically independent from each other because some are more closely related to each other than others.
- Line 233-4. 'there is evidence of at least one genera difference being different from the rest' This is unclear phrasing. I think the authors meant to say, 'the difference between the black and orange spots in at least one genus is different from the difference between the black and orange spots in the other genera'?
- Line 236-7. 'From such an analysis it can be concluded that the univariate approach is not suitable for treating this type of data.' Why can it 'be concluded that the univariate approach is not suitable'? Also, this is Discussion, not Results.
- Line 248. 'statistically significant but negligible' The authors need to justify their contention that 'statistically significant' differences can be considered 'negligible'. Also, if statistically significant differences can be negligible, then what is the point of this analysis?
- Line 270-1. 'pointing to the specific contrast with wider differences' I do not understand what this is supposed to mean.
- Line 275-7. 'The hypothesis in this case is that the differences that were found in terms of can be explained in terms of color components.' If the differences cannot be explained in terms of color components, what *could* explain the differences?
- Figure 6. Why on earth aren't the same genera used for panels A, B, and C (AC, BA, and TR) and also D (AC and SM)? At best, this is sloppy.
- Tables 3 and 4. The captions for both tables say, 'Overall minimum mean is 2.96 and maximum mean is 45.08'. However, this is not true—the overall maximum mean in Table 4 is 19.64, not 45.08.
- Line 289-91. 'Our group has found evidence of a correlation between the color and/or reflectance spectra of beetles of the genus Chrysina (Scarabaeidae) with respect to its phylogenetic tree.' Is there a publication that the authors can cite? If not, this statement must be removed. You can't sneak definitive statements such as these into the refereed literature without subjecting the data behind those statements to peer review. And if this statement can be backed with a citation and does remain in the manuscript, it still doesn't belong in the Results.

- Figure 7. The y-axis should be standardized by row and the x-axis should be standardized by column so that it is easier for the viewer to compare different panels that occur within the same column.
- Line 294-5. 'The mean is plotted as a solid circle of the same color for each set of faded data points.' It would be far more effective to plot the actual data as solid/opaque points, and replace the large dots with semi-transparent convex hulls that encompass the data for each genus.
- Line 301-10. These pairwise comparisons need to be discussed in terms of phylogeny/relatedness.
- Line 312. Table 5 contains Results, so it needs to be cited before the Discussion.
- Line 334-5. 'there is a homogeneity of the sampling' What does this mean?
- Line 339. Specify what these 'more specific contrasts' are.
- Line 342-7. The authors need to cite publications that support their use of spectral comparisons for speculation about whether these taxa have the same pigments. At what threshold of spectral dissimilarity would the authors no longer speculate that these taxa have the same pigments?
- Line 348-9. 'Recent studies report that the color phenotype is clearly associated with the concentration of certain pigment forms' This statement requires multiple citations (one per study, at least two studies).
- Line 349-50. 'there could be other relevant factors besides concentration related to the expression of color' Name those factors.
- Line 350-2. 'In the case of pigments of different color (black vs. orange), the spectral blue components remain almost identical, suggesting that there is a common compound for the pigments.' Why do the authors believe this? Are there any known black and orange pigments that share a common compound that is characterized by its spectral blue component?
- Line 352-5. 'Preliminary chemical characterization by means of infrared spectroscopy showed subtle differences between spectra, suggesting that the pigments differ only by the nature of the functional group attached to the molecule.' Which data are the authors referring to here? They need to reference one of their figures or tables so the reader can see this for themselves.
- Line 358-9. 'A correlation between spectral color and phylogenetic relationship in beetles of the genus Chrysina (Scarabaeidae) has been observed in ongoing research by our group.' See my above comment about lines 289-91.
- Line 363-4. 'Among the few phylogenetic correlations detected was for Evaniella' This does not make sense. What is Evaniella correlated with, and in what sense is this correlation phylogenetic?
- Line 366-8. 'This is an interesting result in that it suggests that the orange color shows more convergent evolution than does the black color.' This is a dangerously bold claim to make, because this claim is based off of just one single (poorly explained) comparison.
- Line 370-1. 'In some other insects, contrasting black and orange color patterns are known to serve as aposematic (warning) coloration for potenital predators [32].' How big are those insects—are they anywhere near the size of the wasps examined here? One wonders whether the predators that might be deterred by aposematic coloration on these wasps would have the visual acuity to notice the orange-on-black coloration in the first place.
- Line 370-2. 'In some other insects, contrasting black and orange color patterns are known to serve as aposematic (warning) coloration for potenital predators [32] and it is likely that the same is true of the BOB pattern, although this has not yet been tested.' In order for this color pattern to be aposematic in these wasps, they must be poisonous/venomous, or they must be mimicking a poisonous/venomous taxon. If the authors wish to argue that these color patterns are 'likely' aposematic, they must explain which poisonous/venomous taxa are involved.

- Line 376-7. ‘it is hoped that the results of the present spectral analyses provide a framework for future research.’ The authors should say how this would be the case—according to this study, how should future research change?

- At the end of the manuscript, the figures are in a random order. If I review this manuscript again I would like to see the figures presented in the correct order, and I would like to see the figure captions included directly below each figure so that I do not need to scroll back and forth.

- Various figures contain unnecessary legends that should be removed: figures 4, 5, 6, and especially 2 and 8.
