# Supplementary material for: Spectral measure of color variation of black-orange-black (BOB) pattern in small parasitoid wasps (Hymenoptera: Scelionidae), a statistical approach
Source: PLoS One. 2019 Oct 24;14(10):e0218061. doi: 10.1371/journal.pone.0218061 (PMC6812806; doi:10.1371/journal.pone.0218061)
Supplement: Attachment — Submitted filename: Response to Reviewers.pdf [file pone.0218061.s014.pdf]

Academic Editor
PLOS ONE

Dear Phillip Barden,

We would like to thank the reviewers for their critical assessment of our work. In the following rebuttal letter, we address each point raised by the academic editor and reviewer(s).

**Comments from Editor and Reviewers**	**Our response (including specific changes made to the document)**
**Chosen color to highlight PLOS ONE comments in the manuscript**<br><br>1. In your Methods section, please provide additional location information, including geographic coordinates for the data set if available.	Done ✔
2. To comply with PLOS ONE submissions requirements for field studies, please provide the following information in the Methods section of the manuscript and in the "Ethics Statement" field of the submission form (via "Edit Submission"):<br>a) Provide the name of the authority who issued the permission for each location (for example, the authority responsible for a national park or other protected area of land or sea, the relevant regulatory body concerned with protection of wildlife, etc.). If the study was carried out on private land, please confirm that the owner of the land gave permission to conduct a study on this site.<br>b) For any locations/activities for which specific permission was not required, please	Information provided. Done ✔

- i. state clearly that no specific permissions were required for these locations/activities, and provide details on why this is the case<br>- ii. confirm that the field studies did not involve endangered or protected species<br>c) For vertebrate studies only, please provide the following additional information:<br>- i. Full details of collection and sampling methods, including method of sacrifice if applicable<br>- ii. State whether the vertebrate work was approved by an Institutional Animal Care and Use Committee (IACUC) or equivalent animal ethics committee. If no approval was obtained, please explain why it was not required.<br>- iii. State clearly whether all sampling procedures and/or experimental manipulations were reviewed or specifically approved as part of obtaining the field permit.<br>For more information about PLOS ONE submissions requirements for field studies, please refer to http://journals.plos.org/plosone/s/submission-guidelines#loc-animal-research.	
3. We note you have included a table to which you do not refer in the text of your manuscript. Please ensure that you refer to Tables 6 & 7 in your text; if accepted, production will need this reference to link the reader to the Table.	The reference was pointing to the supplementary section, but it has been corrected to reference the tables in the following way: "Similarly, top ten comparisons of different genera and the same genus are presented in Table~\ref{tab:T8app} and Table~\ref{tab:T9app}, included in \nameref{S3_Table} in the supplementary material.".<br>Done ✔

<table>
<tr><td>From Reviewer's Responses to Questions
1. Is the manuscript technically sound, and do the data support the conclusions?
Reviewer #1: Partly</td><td>It is not clear to us why reviewer 1 said partly in this case.
Done ✔</td></tr>
<tr><td>From Reviewer's Responses to Questions
3. Have the authors made all data underlying the findings in their manuscript fully available?
Reviewer #2: No</td><td>Data and code is fully available in a Github repo, plus, summary stats were presented in the manuscript. The complete repository will be added as Supplementary Material in a .zip file.

Done ✔</td></tr>
<tr><td colspan="2">The deletions of text suggested by the reviewers were done, having been deleted, they are not observed in color.</td></tr>
<tr><td>Reviewer 1 (chosen color to highlight the suggestions of this reviewer in the manuscript)</td><td></td></tr>
<tr><td>Reviewer 1: The point and motivation of the article

a. Is this a case study, meant to show that FDA is better than univariate analysis for quantifying differences in biological color pattern?
b. Is this a case study, meant to show that more rigorous measurements can and should be used when quantifying differences in biological color pattern?
c.Is the point of this study that colors that appear very similar from the point of view of a human observer can in fact be significantly different from each other?
d. Why did the authors choose this family of wasps?</td><td>A-B This is a case study, meant to show that more rigorous measurements can and should be used when quantifying differences in biological color patterns.

C. No. This phrase was eliminated.

D-E. The Introduction was modified in order to incorporate reviewers suggestions.

F. The BOB pattern includes both colors.

G. See Discussion

Done ✔</td></tr>
</table>

e. Why didn't they make these measurements with other parasitic wasps? Or with flies? Or with flowers?<br>f. What is the additional benefit of analyzing the orange colors, instead of only analyzing the black colors?<br>g. How do the authors hope that this article will influence their colleagues' questions/methods in future studies?	
Reviewer 1: rewrite the abstract following this guide: http://www.easterbrook.ca/steve/2010/01/how-to-write-a-scientific-abstract-in-six-easy-steps/	Done ✔
Reviewer 1: clarity in Abstract. 'describe and test the color differences between genera' What are these 'color differences'? If a color is found to be 'different' between two genera, what does that mean?	Done ✔
Reviewer 1: add a conclusion section with individual sentences - containing each of their main conclusions (significant differences in color, the superiority of FDA over univariate analyses, etc.), arrange those sentences from most to least important starting with the main point of the paper, and then turn their list of sentences into a cohesive paragraph by adding transition phrases.	Done ✔
Reviewer 1: clarity. The Introduction, Methods, and Discussion center on how wonderful FDA is, but neither the phrase 'Functional Data Analysis' nor the abbreviation 'FDA' appear in the Results. If you want to convince people to use a certain method, you	Results using FDA methods were presented in the Results, as well as were commented in the Discussion. The abbreviation FDA was added in those sections in order to make it more clear.<br>Done ✔

must make the results of that method clear.	
Reviewer 1: clarity / Line 157. Which ‘means’ are the authors talking about? (This question is answered a few lines down, in the bullet points, but the reader shouldn’t have to wonder as they finish this paragraph.)	Added “color reflectance”. Done ✔
Reviewer 1: Line 223. ‘independence between spots’ Independence in what sense—independence between the black and orange spots on the same specimen? Between different specimens belonging to the same genus?	Added “on the same specimen”. Done ✔
Reviewer 1: Line 224-6. ‘there is statistical evidence of a difference between mean reflectance of color for at least one genus and zero, controlling by specimen and spot.’ This is not entirely clear. Do the authors mean that, in at least one genus examined, the mean reflectance of the orange spot is significantly different from the mean reflectance of the black spot?	Deleted “there is statistical evidence of a difference between mean reflectance of color for at least one genus and zero” and added “in at least one genus examined, the mean reflectance of the orange spot is significantly different from the mean reflectance of the black spot”. Done ✔
Reviewer 1: Line 247. ‘the differences due to spots’ What does this mean?	Deleted “the differences due to spots” and added “the mean reflectance difference between measurements done in the same specimen but in different spots”.<br>Done ✔
Reviewer 1: Line 259-61. ‘In contrast, the distance between Acanthoscelio (AC) and Acanthoscelio (AC) curves for the same color (in vermilion) clearly include the threshold and can be taken as not significantly different.’ The curves being compared are	Correct, individual curves from different specimens. Added the phrase “and different specimens” for clarity.<br>Done ✔

individual curves from different specimens, right?	
Reviewer 1: Even if it is not possible to implement existing phylogenetic comparative methods in this manuscript, the authors should discuss whether the genera included in the Murray phylogeny that are most closely related to each other are most similar in terms of their color. So far the authors do this for only one pair of genera (‘the orange color of Macroteleia and Sceliomorpha group together for the green and red components (Fig. 7B) despite these genera not being closely related’, lines 360-2). This needs to happen in a comprehensive, deliberate manner.	We have done this in a rewritten paragraph. Done ✔
Reviewer 1: When a genus or species is mentioned for the first time, its name must be followed by its taxonomic authority: the author(s) who described the genus/species. For example, Acanthoscelio is Acanthoscelio Ashmead. Also, the two-letter abbreviation for the generic names just add a layer of unnecessary information to the manuscript. These abbreviations should be removed, and replaced with the full names of the genera.	Done ✔
Reviewer 1: accessibility. Line 91. Explain what the CIE color space is and why it was used here. •	The following lines were added:<br>“Regarding geometrical representation of color stimuli, the CIE color spaces are recognized to have important characteristics such as being device-independent and having a perceptual linearity \cite{tkalcic2003colour}. Nevertheless, not all color spaces are suitable for comparing colors because distances between

	coordinates are not easily quantifiable due to the lack of uniformity of the space. For these effects, the CIE recommends using CIELAB color space due to its uniformity\cite{schanda_colorimetry:_2007}."<br>Done ✔
Reviewer 1: accessibility.Reviewer 1: accessibility. Line 102-3. Explain the biological meaning of delta-E and delta-P. • Line 138. Explain the biological meaning of the 'response variable'. • Line 239-40. Restate the biological meaning of 'the overall average change in all comparisons'. • Line 243. Restate the biological meaning of delta-E when it is first used in the Results.	interpretation<br>delta-E: change or distance between reflectance curves.<br>delta-P: change or distance between L, a or b components. In order to avoid confusion, it was written explicitly delta-L, delta-a, delta-b and the term delta-P was eliminated. The new line reads as follows:<br>"Here, the quantity Delta L, Delta a, Delta b, represent the subtraction of the corresponding coordinates of the two colors being compared and Delta E represents the distance between the two colors within the CIELAB space.<br><br>Response variable (biological meaning): difference of reflectance or a measure of reflectance.<br>Overall average change in all comparisons (biological meaning): average difference between all measurements.<br>Done ✔
Reviewer 1: Line 2-11. The first two paragraphs of the Introduction say the same thing; one of them should be deleted. Also, the biological statements here ('These markings contribute to important behavioral, neurological, physiological, visual, morphological and developmental traits') are too vague/general.	First paragraph deleted. The vague statement has been rewritten to be more specific.<br>Done ✔
Reviewer 1: Line 22-5. 'The wide distribution of the BOB pattern in Hymenoptera suggests that the	These are important observations regarding the original manuscript and we have

coloration arose due to changes in shared metabolic or genetic routes as reported in other taxa, and strongly suggests that it has some biological function [10].' Not necessarily, at least according to the information the authors have provided here. This color pattern could be ancestral (and not serve any biological function) in the hymenopteran clade in question, with widespread, convergent losses.	therefore totally rewritten this section (third paragraph in the Introduction).<br>Done ✔
Reviewer 1: Line 26. 'It has been reported that most animals perceive color differently than humans' Differently in what way?	This phrase has been deleted, as it is not necessary.<br>Done ✔
Reviewer 1: Line 27. 'which has created a new surge of interest' Citations needed to demonstrate that there is a 'surge'.	This phrase has been deleted, as it is not necessary.<br>Done ✔
Reviewer 1: Line 39-40. What data were fed into the PCA?	Added "on the radiance spectra".<br>Done ✔
Reviewer 1: Line 40. 'while recognizing its limitations' What are these 'limitations'?	Added "specifically, those related to the violation of assumptions for statistical tests to discriminate between colors".<br>Done ✔
Reviewer 1: Line 41-42. 'Functional data analysis (FDA) is a powerful method to study spectrally measured data' Powerful how? How does it work, how is it different/better than PCA?	Added "since it assumes that the underlying process generating the data is smooth, and hence provides tools to analyse a spectral measurement as a function and not as a series of data points".<br>Done ✔
Reviewer 1: • Line 67-8. ''They were found and caught with entomological nets over a period of 12 months.' This raises the question of whether there are seasonal changes in color pattern.	There are only two seasons in Costa Rica, dry and wet season, and there is no evidence of change in color pattern during the year. Indeed there appears to be very little intra-specific variation in color (Mora & Hanson 2019).<br>Done ✔

Reviewer 1: Line 68. Evaniidae is a family, not a genus.	We added the genus of Evaniidae<br>Done ✔
Reviewer 1: Line 78-9. 'A previous study demonstrated that males and females do not differ in color' This finding holds across a large clade that includes Scelionidae and also Evaniidae?	Yes. Changed to read: "A previous study demonstrated that the vast majority of hymenopterans with the BOB pattern, and those that are all black, do not show sexual dimorphism in color, at least in specimens that have been identified to species level in museum collections [15]."<br>Done ✔
Reviewer 1: Line 85-86. 'For each specimen three samples were taken from the mesosoma (orange) and from the metasoma (black).' Three samples were taken from the mesosoma and three were taken from the metasoma? This sentence is unclear.	We have clarified this sentence.<br>Done ✔
Reviewer 1: Line 88-89. 'Fig 2 shows the spectral reflectance of the eight genera for the black and orange segments together with the control genus.' This sentence belongs in the Results, not Methods. Also in what sense is the evaniid a 'control'? This seems like the wrong word to use.	Instead of "control" we now use "distantly related", as explained in a previous paragraph.<br>Done✔
Reviewer 1: Line 109. Spell out the whole term instead of using the abbreviation 'MSP'—this abbreviation is not used frequently enough to justify making the reader memorize it.	Done✔
Reviewer 1: Line 164. Define 'FANOVA'. I suspect the authors should cite its origination.	Added reference.<br>Done ✔
Reviewer 1: Line 165-7. 'A statistical test was performed to interpret the contrast results for the mean	Rephrased.<br>Done ✔

difference curves.' Which statistical test?	
Reviewer 1: Line 171-2. 'Here also, specimen and spot were controlled.' This is unclear.	Deleted "controlled", and added "included as factors in the linear equation".<br>Done ✔
Reviewer 1: Line 173-4. ``the usual univariate practice' The authors imply that the univariate method is commonly used, but I don't recall seeing any citations that show this to be the case.	Deleted "the usual univariate practice" and added "a univariate analysis approach".<br>Done ✔
Reviewer 1: Line 174-5. 'to highlight the importance of taking into account all the information from the difference curves.' It's disconcerting to see the authors state, in the Methods, that they designed their study based on the assumption that one analysis will perform significantly better than another.	Deleted "highlight the importance" and added "compare the results when".<br>Done ✔
Reviewer 1: Line 176. It's odd to see the same equation used for the univariate and functional responses, since each response was analyzed with a different kind of ANOVA and the functional response was analyzed with a metric computed with a special R package.	In order to simplify the section, we didn't include separate equations. The detail is that for FDA each observation is a curve, so the only thing that changes is the response: from Y to Y(lambda), the rest of the factors are the same, since they continue being measured in the same way. This has very important implications mathematically, hence the different packages, but it can be noisy to include a separate equation.<br>Done ✔
Reviewer 1: Line 178. What is the 'p =' doing in there? Why not just write 'i = 1, 2, ... , 8'?	Deleted p.<br>Done ✔
Reviewer 1: Line 178. Change 'j = 1, 2, .., r = 4 ' to 'j = 1, 2, 3, 4'.	Deleted r.<br>Done ✔

Reviewer 1: Line 197. I recommend that the authors cite the R package used to make their figures	Excellent suggestion. We added the citation. Added "Main data format and figures were done using the collection of R packages Tidyverse \cite{tidy}".<br>Done ✔
Reviewer 1: Line 205. The caption for Figure 2 is not provided anywhere.	Perhaps you missed it, its at the top of page 4.<br>Done ✔
Reviewer 1: Line 211-2. 'Moreover, the effect of topography of the cuticle and homogeneity of the pigment distribution can partly explain the data dispersion in some of the genera.' The authors need to substantiate this statement with a figure, data, something.	"Because the spot size of 200µm x 200µm used the reflectance measurements is more than 20 times larger than the features that comprise the mesosoma, the effect of topography of the cuticle and homogeneity of the pigment distribution can partly explain the data dispersion in some of the genera. See supplementary material S4"<br>Done ✔
Reviewer 1: Line 213. 'The difference reflectance of the curves' I think this is grammatically incorrect.	We are referring to the curve calculated as the difference, wavelength by wavelength, between two reflectance curves.<br>Changed the beginning of the sentence to "The difference curves for reflectance spectra".<br>Done ✔
Reviewer 1: Line 216-7. 'in the range between 550 nm and 750 nm, and the region between 750 nm and 900 nm' Why not change this to 'in the range between 550 nm and 900 nm'? Also the measurements go above 900 nm.	We meant to draw attention to the fact that the behavior of the curves can be described according to the wavelength. We substituted the whole sentence by:<br><br>" For each genus, the behavior of the difference curves between black and orange can be described according to the wavelength in the following way. Before 550nm, differences are around zero, meaning that the spectra are similar in this region. Between 550nm and 750nm the differences are below zero and are similar between genera. And finally, for wavelengths greater than 750nm differences are both above and below zero and are

	dispersed with respect to genus.<br>Also, eliminated the sentence: “Thus the differences between colors in each genus can be explained depending on the wavelength range” because that idea was already mentioned with the new sentence.<br>Done ✔
Reviewer 1: Figure 3. There is so much overlap between the different ribbons that it is often impossible to distinguish them. I suggest that the authors use a thick line for the mean value and thinner lines to mark the upper and lower extremes of each ribbon, leaving the interior blank (without any fill) so that it’s easier to distinguish the ribbons	Corrected the plot (See Fig3.tiff)<br>Done ✔
Reviewer 1: Line 220-1 (Figure 3 caption). What does it mean if the curve is below the black line versus above it?	Changed to caption to: “Difference (black-orange) of mean reflectance functions in each genus}}. Solid lines represent functional means, colored areas represent the area where difference functions in each genus are observed. A black dashed line represents the null hypothesis of no difference between colors in each genus. Curves above the black line mean that the black curves have higher reflectance values than the orange ones.”<br>Done ✔
Reviewer 1: Line 222-3. ‘The best fit in all cases was from the models that assume independence between spots, so linear models were used subsequently.’ I’m not sure exactly what mean by this (as outlined in the above section), but the genera examined here are not statistically independent from each other because some are more closely related to each other than others.	When fitting statistical models one can assume dependence and then compare those models taking into account dependence with others assuming independence. This phrase intends to clarify that the models assuming independence had a better fit than those that did not assume it. Therefore, we continued using the later ones.<br>Done ✔

Reviewer 1: Line 233-4. 'there is evidence of at least one genera difference being different from the rest' This is unclear phrasing. I think the authors meant to say, 'the difference between the black and orange spots in at least one genus is different from the difference between the black and orange spots in the other genera'?	Deleted "there is evidence of at least one genera difference being different from the rest" and added "the difference between the black and orange spots in at least one genus is different from the difference between the black and orange spots in the other genera".<br>Done ✔
Reviewer 1: Line 236-7. 'From such an analysis it can be concluded that the univariate approach is not suitable for treating this type of data.' Why can it be concluded that the univariate approach is not suitable'? Also, this is Discussion, not Results.	Deleted "From such an analysis it can be concluded that the univariate approach is not suitable for treating this type of data."<br>Done ✔
Reviewer 1: Line 248. 'statistically significant but negligible' The authors need to justify their contention that 'statistically significant' differences can be considered 'negligible'. Also, if statistically significant differences can be negligible, then what is the point of this analysis?	The significance tests should have a context in terms of the application. In this case, we found a bibliographical reference for a threshold. The point of this analysis is to compare differences statistically, taking into account application-based thresholds. See: https://amstat.tandfonline.com/doi/full/10.1080/00031305.2016.1154108#.XUmT3JMzbOR<br>Done ✔
Reviewer 1: Line 270-1. 'pointing to the specific contrast with wider differences' I do not understand what this is supposed to mean.	In statistics, a contrast is a comparison test among two groups or a linear combination of groups. Here, we refer to specific comparisons between pairs of genera and/or specific colors. We replaced the word contrast with comparisons.<br>Done ✔
Reviewer 1: Line 275-7. 'The hypothesis in this case is that the differences that were found in terms of can be explained in terms of color components.' If the differences cannot be explained in terms of color	Here we meant to address the possibility of using spectral color components as an analysis method for accessing information about the optical properties related to pigments without using color coordinates, that is, avoiding the question of whether the color is orange, red, brown or black to

components, what could explain the differences?	human vision. When calculating Delta E, all the information regarding the different wavelengths is integrated into a number. When using spectral components analysis, it becomes clear whether this difference comes from shorter or longer wavelengths. This information is important because optical properties are related to energetic process due to electronic transitions in the pigment molecules when interacting with electromagnetic radiation. The shorter the wavelength, the more energetic the process is and therefore it becomes more relevant in the identification of the pigment. In order to clarify the importance of this information, we replaced the first paragraph of the section named "RGB spectral components" of the **Materials and Methods Section** with the following paragraph:<br><br>"When calculating Delta E, all the spectral information is condensed into a single number and it is not clear whether the difference comes from shorter or longer wavelengths. In this sense, the use of a spectral space instead of a color space would provide a more objective description of the physical variation among stimuli and also allows the identification of different physical mechanisms producing the same perception of color but having different spectral behavior [Renault2017]Therefore, in order to visualize the characteristics of the reflectance curves in different regions of the electromagnetic spectrum, in this work, we propose a trichromatic spectral space defined using the IE color matching functions (CMFs) as follows:"<br><br>Also, the line mentioned by Referee 1 was deleted because the paragraph was changed.<br>Done ✔
Reviewer 1: Figure 6. Why on earth aren't the same genera used for panels A, B, and C (AC, BA, and TR)	Very good point. Redo the plot. We included only AC and TR on panel D for clarity, since

and also D (AC and SM)? At best, this is sloppy.	including a third set of curves could make it difficult to contrast the curves. See Fig6.tiff.<br>Done ✔
Reviewer 1: Tables 3 and 4. The captions for both tables say, 'Overall minimum mean is 2.96 and maximum mean is 45.08'. However, this is not true—the overall maximum mean in Table 4 is 19.64, not 45.08.	In this case, we are talking about the whole sample, and each table represents the top ten, thus, the caption is meant to clarify the descriptive statistics of the whole sample and not only from the top ten. Added to the captions: "The top 10 was extracted from a sample with overall minimum mean equal to 2.96 and maximum mean to 45.08"<br>Done ✔
Reviewer 1: Line 289-91. 'Our group has found evidence of a correlation between the color and/or reflectance spectra of beetles of the genus Chrysina (Scarabaeidae) with respect to its phylogenetic tree.' Is there a publication that the authors can cite? If not, this statement must be removed. You can't sneak definitive statements such as these into the refereed literature without subjecting the data behind those statements to peer review. And if this statement can be backed with a citation and does remain in the manuscript, it still doesn't belong in the Results.	This is a recent finding of our group. We are still working on this, therefore it hasn't been published yet. Following the recommendation, the statement was deleted from the manuscript.<br><br>We added instead :<br><br>"Mapping color variants in a spectral space can yield to information about the relationship between genotypic space and evolution [Whibley et al.2016]. In this context, in order to visualize the data provided by the spectral component analysis as a function of the genus, Fig 7 is presented."<br>Done ✔
Reviewer 1: Figure 7. The y-axis should be standardized by row and the x-axis should be standardized by column so that it is easier for the viewer to compare different panels that occur within the same column.	Redo the plot<br>See Fig7.tiff<br>Done ✔
Reviewer 1: Line 294-5. 'The mean is plotted as a solid circle of the same color for each set of faded data points.' It would be far more effective to plot the actual data as	Convex hulls add more noise to the plot, so we left it with the semitransparent points.<br>Done ✔

solid/opaque points, and replace the large dots with semi-transparent convex hulls that encompass the data for each genus.	
Reviewer 1: Line 301-10. These pairwise comparisons need to be discussed in terms of phylogeny/relatedness.	We have expanded this part to read: "Comparing the results shown in Fig. 7 with a recent phylogenetic reconstruction of scelionid genera [35], a few other relationships between spectral properties and phylogeny appear to be present. For example, *Baryconus* and *Chromoteleia* are paired in the green and red components for both black and orange, and these two genera are hypothesized to be closely related. *Opisthacantha* and *Triteleia* group together in the blue and green components of the orange coloration, and these two genera are also closely related. On the other hand, the orange color of *Macroteleia* and *Sceliomorpha* group together for the green and red components (Fig. 7B) despite these genera not being closely related. These results are intriguing and merit further investigation to determine whether certain spectral properties parallel the phylogeny."<br><br>Done ✔
Reviewer 1: Line 312. Table 5 contains Results, so it needs to be cited before the Discussion.	Done ✔
Reviewer 1: Line 334-5. 'there is a homogeneity of the sampling' What does this mean?	Deleted "a homogeneity of the sampling" and added "low variability in the sample spectral measurements".<br>Done ✔
Reviewer 1: Line 339. Specify what these 'more specific contrasts' are.	Added "between genera and color".<br>Done ✔
Reviewer 1: Line 342-7. The authors need to cite publications that support their use of spectral comparisons for speculation about whether these taxa	We agree, but it seems to us that this acclaration belongs more to the **"RGB spectral components**" section of the **Materials and Methods Section instead of Discussion. In this section,** we added the line:

have the same pigments. At what threshold of spectral dissimilarity would the authors no longer speculate that these taxa have the same pigments?	"It is recognized that spectral spaces have the advantage of providing information about the different processes generating phenotypic color diversity[Renuolt2017], and therefore we propose this RGB spectral component method as an alternative for the case when a direct chemical characterization can not be performed"<br><br>The question about the threshold is very important. We hoped to address this issue by the use of comparisons between color component curves in terms of the distance between them, and using experimental design techniques to measure the uncertainty associated with the difference between curves, and hence reporting the statistical differences along with the practical differences (see comment for Reviewer 1: Line 248).<br><br>Results are summarized in Fig 6 for three different genera, and also the Top 10 most different pairs of curves are summarized in Tables 4,5,6,7.<br>Done ✔
Reviewer 1: Line 348-9. 'Recent studies report that the color phenotype is clearly associated with the concentration of certain pigment forms' This statement requires multiple citations (one per study, at least two studies).	Done ✔
Reviewer 1: Line 349-50. 'there could be other relevant factors besides concentration related to the expression of color' Name those factors.	Changed to "...but other factors such as nanostructures of the integument can affect the expression of color [Shawkey & D'Alba 2017]<br><br>Shawkey MD & D'Alba L. 2017 Interactions between colour-producing mechanisms and their effects on the integumentary colour palette. Philosophical Transactions of the

	Royal Society B 372: 20160536.<br>Done ✔
Reviewer 1: Line 350-2. 'In the case of pigments of different color (black vs. orange), the spectral blue components remain almost identical, suggesting that there is a common compound for the pigments.' Why do the authors believe this? Are there any known black and orange pigments that share a common compound that is characterized by its spectral blue component?	Since wavelengths involved in the blue component are shorter than the ones involved in the red and even in the green component, electronic transitions associated with the characteristics of the spectrum in this region are considered of importance. The fact that even for different colors (black vs. orange) the components are identical is an important piece of information regarding the nature of the pigments involved in the black and orange coloration observed. See also comment about line 275-7.<br><br>The following line was added:<br><br>"This information is important because optical properties are related to energetic process due to electronic transitions when the pigment molecules interact with electromagnetic radiation. The shorter the wavelength, the more energetic the process is and therefore differences can be interpreted in terms of differences of electronic structure, which is related to the chemical composition of the pigments."<br>Done ✔
Reviewer 1: Line 352-5. 'Preliminary chemical characterization by means of infrared spectroscopy showed subtle differences between spectra, suggesting that the pigments differ only by the nature of the functional group attached to the molecule.' Which data are the authors referring to here? They need to reference one of their figures or tables so the reader can see this for themselves.	We realize the ideal method for determining whether the pigments are the same or no, is a chemical characterization by other means such as liquid chromatography coupled to a mass spectrometer or infrared spectroscopies, just to mention two widely used techniques. Nevertheless, such techniques require to perform a chemical extraction of the pigment, which was not attempted due to the small amount of material available. The use of optical properties is then proposed as an alternative method when the chemical extraction can not be performed. We attempted infrared spectroscopy directly on the cuticle of the insects. But since in this case there is a matrix in which the pigments are embedded, results

	showed subtle differences, but were not conclusive. This data is not included in the manuscript but it can be included if the referee considers it is necessary. Otherwise we have substituted this statement by the following:<br><br>"A chemical characterization using infrared spectroscopy was attempted directly on the cuticle and results showed subtle differences suggesting a similar nature of the functional group attached to the molecule. Such results are not shown here because they are not conclusive due to the fact that the exact chemical composition of the cuticle for each color is unknown. A definitive identification of the pigment requires to perform a chemical extraction which at the moment could not be done due to the small amount of cuticle available."<br>Done ✔
Reviewer 1: Line 358-9. 'A correlation between spectral color and phylogenetic relationship in beetles of the genus Chrysina (Scarabaeidae) has been observed in ongoing research by our group.' See my above comment about lines 289-91.	Recommendation taken. See below.<br>Done ✔
Reviewer 1: Line 363-4. 'Among the few phylogenetic correlations detected was for Evaniella' This does not make sense. What is Evaniella correlated with, and in what sense is this correlation phylogenetic?	The paragraph now reads:<br>*Evaniella* belongs to a completely different family (Evaniidae) and might therefore be expected to differ markedly from all the other genera. This indeed appears to be the case for its black color, but not its orange color (Fig. 7). This is an interesting result in that it might suggest that the orange color shows more convergent evolution than does the black color, but it remains to be demonstrated that the pigments are actually convergent. Nonetheless, within Scelionidae there are more differences in the orange color than in the black (Table 4). Comparing the results shown in Fig. 7 with a recent

	phylogenetic reconstruction of scelionid genera [35], a few other relationships between spectral properties and phylogeny appear to be present. For example, *Baryconus* and *Chromoteleia* are paired in the green and red components for both black and orange, and these two genera are hypothesized to be closely related. *Opisthacantha* and *Triteleia* group together in the blue and green components of the orange coloration, and these two genera are also closely related. On the other hand, the orange color of *Macroteleia* and *Sceliomorpha* group together for the green and red components (Fig. 7B) despite these genera not being closely related. These results are intriguing and merit further investigation to determine whether certain spectral properties parallel the phylogeny."
Reviewer 1: Line 366-8. 'This is an interesting result in that it suggests that the orange color shows more convergent evolution than does the black color.' This is a dangerously bold claim to make, because this claim is based off of just one single (poorly explained) comparison.	See above.
Reviewer 1: Line 370-1. 'In some other insects, contrasting black and orange color patterns are known to serve as aposematic (warning) coloration for potential predators [32].' How big are those insects—are they anywhere near the size of the wasps examined here? One wonders whether the predators that might be deterred by aposematic coloration on these wasps would have the visual acuity to notice the orangeon-black coloration in the first place.	Ladybird beetles are slightly larger than scelionid wasps, but not by much. The largest scelionid overlaps in size with the smallest ladybird.<br><br>We are currently carrying out experiments with jumping spiders, which appear to have the necessary visual acuity to detect the scelionid orange-on-black

Reviewer 1: Line 370-2. 'In some other insects, contrasting black and orange color patterns are known to serve as aposematic (warning) coloration for potential predators [32] and it is likely that the same is true of the BOB pattern, although this has not yet been tested.' In order for this color pattern to be aposematic in these wasps, they must be poisonous/venomous, or they must be mimicking a poisonous/venomous taxon. If the authors wish to argue that these color patterns are 'likely' aposematic, they must explain which poisonous/venomous taxa are involved.	We agree and have therefore changed "likely" to "possible". The indirect evidence provided in Mora & Hanson 2019 suggests aposematism and/or mimicry, but future research is needed to address this question.
Reviewer 1: Line 376-7. 'it is hoped that the results of the present spectral analyses provide a framework for future research.' The authors should say how this would be the case—according to this study, how should future research change?	Added:<br>"...especially when a direct chemical characterization of the pigments is not possible and the optical and statistical methods proposed in this work can be applied." at the end of this line.<br><br>Done ✔
Reviewer 1: At the end of the manuscript, the figures are in a random order. If I review this manuscript again I would like to see the figures presented in the correct order, and I would like to see the figure captions included directly below each figure so that I do not need to scroll back and forth.	We followed the instructions on the submission system and we are not entirely sure on why the figures are in a random order (we submitted them in order). Also, the figure captions are included directly below the first reference to the figure in the text as indicated in the PLOS one system instructions: "**Place** figure captions in the manuscript text in read order, immediately following the paragraph where the figure is first cited. Do not include captions as part of the figure files or submit them in a separate document." https://journals.plos.org/plosone/s/figures#loc-how-to-submit-figures-and-captions<br>Done ✔

Reviewer 1: Various figures contain unnecessary legends that should be removed: figures 4, 5, 6, and especially 2 and 8.	Removed legends in figures 2 (Fig2.tiff) 4 (Fig4.tiff), 5 (Fig5.tiff), 6 *Fig6.tiff) and 8 (Fig8.tiff).<br>Done ✔
Reviewer 2 (chosen color to highlight the suggestions of this reviewer in the manuscript)<br>I would recommend that the authors make the introduction and methods sections more concise; the methods section is on the long side and some information could perhaps be moved to the supplementary material.	Same suggestion as Reviewer 1. See above for comments.
Reviewer 2: the discussion section is lacking somewhat with regard to the potential usefulness and impact of the study and would appreciate some more substantive explanation in this regard. Nevertheless, if this approach will form the basis of future studies from this group then I am intrigued to see results of the correlation between spectral colour and phylogenetic relationships mentioned in the case of the *Chrysina* beetle genus.	The following paragraph was added at the beginning of the Discussion for the guidance of the reader:<br><br>"The following discussion is focused mainly on the contribution of the different statistical approaches to the physical and biological interpretation of the data obtained following the procedures proposed, with the aim of establishing a quantitative analysis method for the study of cuticle color of insects. This section has two main parts: discussion about differences and suitability of the statistical methods and the interpretation of the physical results within a biological context. "<br><br>The following line was rewritten and moved right after the new first paragraph of Discussion:<br><br>"Regarding the suitability of the statistical methods applied, the present study highlights the differences in terms of results that could arise using univariate methods, which ignore physical information contained in the reflectance measurement as a function of wavelength, versus analyzing data using FDA techniques."<br><br>Also, we think that some of the changes that have been made in the manuscript as a

	whole contribute to stating more clearly the potential impact of our study:<br><br>● In response to the comments of the referees, the aim of our work is now stated more clearly in the introduction, and we hope this clarifies the impact of the study.<br>● As suggested by a referee, we have added a conclusion section, which clarifies the contribution of our research.<br>● As a result of comments made by another referee, the mention of Chrysina studies was eliminated from the manuscript since this research is not yet ready for publication. Nevertheless, we want to clarify that it was the work on Chrysina that inspired us to try to find a phylogenetic correlation in this study, not the other way around.<br>Done ✔
Reviewer 2: Line 18 – pheomelanin is a type of melanin so with regard to browncoloured cuticle, eumelanin should be specified rather than saying melanin	Done ✔
Reviewer 2: Line 23 – change 'arose' to 'arises' to keep tense consistent	Done ✔
Reviewer 2: Line 24 – can the authors mention some examples of other taxa with respect to shared metabolic and genetic routes	The part about metabolic and genetic routes is confusing and so we have deleted it.<br>Done ✔
Reviewer 2: Figure 1 caption – what information is the photograph (Fig. 1C?) providing? I couldn't see any highlighted square? The two highlighted areas both look circular to me.	Done ✔

Reviewer 2: Line 44 – 'A statistical approach of reflectance measurements…' does not read well. I suggest changing 'of' to something like 'based upon'	Replaced "of" for "based upon.<br>Done ✔
Reviewer 2: Line 44-51 – This final paragraph of the introduction feels more suited to the methods section<br>Reviewer 2: Line 137 – As someone who's not an expert on parasitic wasps I think a short explanation of why Evianella is a suitable control specimen would be helpful.	Done ✔<br><br>Done ✔
Reviewer 2: Line 147 – presumably should read 'as defined in Eq. 1'	Done ✔
Reviewer 2: Line 213 – 'The difference reflectance…' does not make sense. Change to something like 'The difference in reflectance measured for the curves…'	Reviewer 1 also commented on this line. Changed the beginning of the sentence to "The difference curves for reflectance spectra"<br>Done ✔
Reviewer 2: Line 282 – 'Fig 6 in panel (D)' should be changed to 'Fig 6D shows' or 'Panel (D) of Fig 6 shows'	Changed 'Fig 6 in panel (D)' for 'Panel (D) of Fig 6 shows'.<br><br>Done ✔
Reviewer 2: Line 291 – typo in figure reference link	Done ✔
Reviewer 3: **(chosen color to highlight the suggestions of this reviewer in the manuscript)**<br><br>For the first time , abstract	Done ✔
Reviewer 3: word corrector eliminations line 7, 8 abstract	Done ✔
Reviewer 3: word corrector eliminations line 13,15 and 16, introduction	Done ✔

Reviewer 3: line 20 comment<br>This is not correct. Scelionidae is a large and cosmopolitan family and the vast majority of them do not have the BOB pattern. Furthermore, this pattern is limited to the Neotropics. The same genera in N. America, Africa, etc. do not have this pattern.	We have incorporated this important clarification: "Various scelionid wasps, principally neotropical species ranging from 3 to 10 mm in length, show a recurring color pattern of …" However, the color pattern does occur in other parts of the world, especially in non-scelionid hymenopterans (see Mora & Hanson, 2019).<br>Done ✔
Reviewer 3: line 23 comment<br>I strongly disagree. The shared metabolic and genetic routes of the genera treated here are also shared by these genera outside of the Neotropics, where these color patterns do not occur. So, it cannot be a function of shared ancestry.	Done ✔<br>We have deleted this part about "shared metabolic and genetic routes".
Reviewer 3: Figure 1 legend, one word corrector elimination and meaning of 0,6 mm measurement	Done ✔
Reviewer 3: line 38, 39 and 42 word corrector eliminations and verb past tense	Done ✔
Reviewer 3:line 41<br>Functional data analysis (FDA) is a powerfu<br>These words are all capitalized earlier in the manuscript. Please be consistent about capitalization.	Done ✔
Reviewer 3: line 45 and 46 taxonomy/typos	Done ✔

Reviewer 3: line 65,66,67 author of genus	Done ✔
Reviewer 3: line 66<br>Lapitha (LA), Macroteleia (MA), Scelio (SC), [illegible]<br>Lapitha is not a valid genus. It was treated as a junior synonym of Opisthacantha by Masner 1976 and has not been resurrected. The author of Opisthacantha is Ashmead	Done ✔
Reviewer 3: line 67 change words for collected	Done ✔
Reviewer 3: line 68 author of genus	Done ✔
Reviewer 3: line 69 extra ,	Done ✔
Reviewer 3: line 73 word corrector elimination	Done ✔
Reviewer 3: line 74<br>Specimens were identified by R.M. and P.H.<br>Identified using what key? Masner 1976 should be referenced if that was how the specimens were identified.	We added this reference, which was indeed used for identification.<br>Done ✔
Reviewer 3: line 76<br>Due to the lack of taxonomic studies, species<br>Acanthoscelio was revised in 2001 (Dotseth & Johnson); Chromoteleia was revised in 2018 (Chen et al.: https://zookeys.pensoft.net/article/25775/)	This detail has been incorporated into the text<br>Done ✔
Reviewer 3: line 76 and 79 word corrector elimination and word incorporation	Done ✔

Reviewer 3: line 79<br>If the taxonomy has not been studied, how do you know that males and females are correctly associated?	Now reads: "...the vast majority hymenopterans with the BOB pattern, and those that are all black, do not show sexual dimorphism in color, at least in specimens that have been identified to species level in museum collections [15]."<br>Done ✔
Reviewer 3: line 83 and 84, 3 word corrector suggestions	Done ✔
Reviewer 3: Line 89<br>I suggest using a term other than "segment", which has a specific meaning in insect morphology. How about " section"?	Done ✔
Reviewer 3: line 110 word corrector suggestion	Done ✔
Reviewer 3: line 150, word corrector elimination	Done ✔
Reviewer 3, line 196<br>download in https://github.com/malfaro2<br>This should also be included as supplemental data with the publication.	We will try to include it in the submission system as Supplemental material in a .zip file, but it is worth noticing that the zip file is 86.3MB, and we do not know if it exceeds the limit.
Reviewer 3, Line 210, 213, 215 word corrector incorporations	Done ✔
Reviewer 3, Line 234 word corrector change of word	Done ✔
Reviewer 3, Line 241 simplify the sentence (The most important factor in this case is when there is a change of color, since the estimate of η is 242 26.2081, which means that keeping all the other factors constant, the	Deleted "The most important factor in this case is when there is a change of color, since"<br>Done ✔.

average ΔE is 26 243 units greater than the overall mean when the curves differ in color).	
Reviewer 3, Line 262 word correction	Done ✔
Reviewer 3, Line 274<br>Are green, red, and blue used because they correspond to human photoreceptors? If so, that should be clarified, and what are the wavelengths attributed to each of these colors?	Yes. This calculation is based on the human photoreceptors. We are referring to spectral components, not to a single wavelength. Nevertheless, it is possible to define a characteristic wavelength for each curve, which is the wavelength at which the maximum of the color matching function occurs. Since a comment in this sense corresponds more to the description of the calculation of the color components, we added the information about the corresponding wavelengths but in the section **“RGB components”** of the **Methods section** instead of the Results section.<br><br>“We label these components as Green, Red and Blue (RGB) because their maximum amplitude occur at 700 nm, 546 nm and 436 nm respectively.”<br>Done ✔
Reviewer 3, Line 283 word corrections	Done ✔
Reviewer 3, Line<br>spectra of beetles of the genus *Chrysina* (Sca<br>tree. Following the same line of thought. Fig<br>"with respect to its phylogenetric tree" can mean many things. Please clarify what you mean. Also, please follow Chrysina with the author of this genus name.	This line was eliminated, due to the fact that it refers to results that are still in study and have not been published yet<br>Done ✔
Reviewer 3, Line 313 and 315 word corrections	Done ✔
Reviewer 3, Line 350	No. The spectral components are curves that were normalized using the area under the curve. This procedure make the curves

Could it also be the case the spectral blue components are unrelated to the pigments that create the orange-black colors?	comparable between them. So, even if orange is expected to have less blue than black, for example, the information related to chemical composition of the pigment is contained in features such as the shape of the curves and the wavelength at which the maximum occurs. For the sake of clarity, the line was modified as follows:<br><br>"In the case of pigments of different colors (black vs. orange), the spectral blue components remain almost identical in terms of shape and characteristic wavelength (where the maximum occurs), suggesting that there is a common compound for the pigments. Notice that the normalization of the spectral components, equations (2), (3) and (4), allows the use of these features for comparison instead instead of intensity, which is related to the quantity of red, green or blue present in each color.<br>Done ✔
Reviewer 3, Line 367<br>Having a similar spectral profile is not the same as convergent evolution. You would first need to provide evidence that the pigments are actually convergent in some way. It could be the case that very different pigments simply have a similar spectral profile.	We have made this clarification in the text: "This is an interesting result in that it might suggest that the orange color shows more convergent evolution than does the black color, but it remains to be demonstrated that the pigments are actually convergent."<br>Done ✔
Reviewer 3, Line 371 word corrector elimination	Done ✔
Reviewer 3, Line 374	We have made this clarification in the text: "...thermoregulation (black absorbs more sunlight) might play a role at these elevations; it is also possible that predators or aposematic models vary with elevation."<br>Done ✔

Is this asserting that they would be black to absorb more sunlight? It could also be that there are different predators at high elevation, or the aposematic model of the mimicry complex is not present at higher elevation. It should be noted that BOB occurs in large scelionids.	
Please ensure that your manuscript meets PLOS ONE's style requirements, including those for file naming. The PLOS ONE style templates can be found at http://www.journals.plos.org/plosone/s/file?id=wjVg/PLOSOne_formatting_sample_main_body.pdf and http://www.journals.plos.org/plosone/s/file?id=ba62/PLOSOne_formatting_sample_title_authors_affiliations.pdf	Done ✔
While revising your submission, please upload your figure files to the Preflight Analysis and Conversion Engine (PACE) digital diagnostic tool, https://pacev2.apexcovantage.com/ . PACE helps ensure that figures meet PLOS requirements. To use PACE, you must first register as a user. Registration is free. Then, login and navigate to the UPLOAD tab, where you will find detailed instructions on how to use the tool. If you encounter any issues or have any questions when using PACE, please email us at figures@plos.org. Please note that Supporting Information files do not need this step.	Done ✔
