# Supplementary material for: Spectral measure of color variation of black-orange-black (BOB) pattern in small parasitoid wasps (Hymenoptera: Scelionidae), a statistical approach
Source: PLoS One. 2019 Oct 24;14(10):e0218061. doi: 10.1371/journal.pone.0218061 (PMC6812806; doi:10.1371/journal.pone.0218061)
Supplement: Attachment — Submitted filename: PONE-D-19-14642_R1.pdf [file pone.0218061.s015.pdf]

PLOS ONE

Spectral measure of color variation of black - orange - black (BOB) pattern in small parasitic wasps (Hymenoptera: Scelionidae), a statistical approach

--Manuscript Draft--

**Manuscript Number:**	PONE-D-19-14642R1
**Article Type:**	Research Article
**Full Title:**	Spectral measure of color variation of black - orange - black (BOB) pattern in small parasitic wasps (Hymenoptera: Scelionidae), a statistical approach
**Short Title:**	Statistical study of color in Hymenoptera (Scelionidae)
**Corresponding Author:**	Rebeca Mora-Castro<br>Universidad de Costa Rica<br>COSTA RICA
**Keywords:**	Hymenoptera; Scelionidae; color pattern; microspectrophotometry; functional data analysis.
**Abstract:**	Small parasitic wasps are abundant and extremely diverse, yet their colors remain mostly uncharacterized. One of the more common color patterns observed in these wasps is a black-orange-black pattern, which is especially common among neotropical species of Scelionidae ranging in size from 2 to 10 mm. Due to the methodological challenges involved in extracting and characterizing pigments from small-sized insects, other methods for examining colors need to be explored. In this work, we propose the use of microspectrophotometry in combination with statistical analysis methods in order to study the spectral properties in such cases. We examined 8 scelionid genera and 1 genus from a distantly related family (Evaniidae), all showing the black-orange-black pattern. Functional Data Analysis and statistical analysis of Euclidean distances for color components were applied to study color differences both inter and intragenera. The Functional Data Analysis proved to be a better method for treating the reflectance data because it gave a better representation of the physical information. Also, the reflectance spectra were separated into spectral color component contributions and each component was labeled according to its own dominant wavelength at the maximum of the spectrum: Red, Green and Blue. When comparing spectral components curves, the spectral blue components of the orange and black colors, independent of the genera being compared, result almost identical, suggesting that there is a common compound for the pigments. The results also suggest that cuticle from different genera, but with the same color might have a similar chemical composition. This is the first time that the black and orange colors in small parasitic wasps has been characterized and our results provide a basis for future research on the color patterns of an abundant but neglected group of insects.
**Order of Authors:**	Rebeca Mora-Castro
	Marcela Hernández-Jiménez
	Marcela Alfaro-Córdoba
	Esteban Avendaño-Soto
	Paul E. Hanson-Snortum
**Response to Reviewers:**	Please see "Response to reviewers" document. We wrote all responses to the reviewers in the document 'Response to Reviewers', including the 6 questions labeled as "Comments to the Author". For each comment from the editor and reviewer, we have a response that includes specific changes that have been made. Also, all the figures and code have been corrected according to their suggestions, and new figure versions have been uploaded.
**Additional Information:**	
**Question**	**Response**
**Financial Disclosure**	This project was supported by the University of Costa Rica (https://vinv.ucr.ac.cr/) grant No.111-B2-A51 (RM) and 801 A5B50 (RM). The funders had no role in study design,

Enter a financial disclosure statement that describes the sources of funding for the work included in this submission. Review the submission guidelines for detailed requirements. View published research articles from *PLOS ONE* for specific examples.<br><br>This statement is required for submission and **will appear in the published article** if the submission is accepted. Please make sure it is accurate.<br><br>**Unfunded studies**<br>Enter: *The author(s) received no specific funding for this work.*<br><br>**Funded studies**<br>Enter a statement with the following details:<br>• Initials of the authors who received each award<br>• Grant numbers awarded to each author<br>• The full name of each funder<br>• URL of each funder website<br>• Did the sponsors or funders play any role in the study design, data collection and analysis, decision to publish, or preparation of the manuscript?<br>• **NO** - Include this sentence at the end of your statement: *The funders had no role in study design, data collection and analysis, decision to publish, or preparation of the manuscript.*<br>• **YES** - Specify the role(s) played.<br><br>* typeset	data collection and analysis, decision to publish, or preparation of the manuscript.
**Competing Interests**<br><br>Use the instructions below to enter a competing interest statement for this submission. On behalf of all authors, disclose any competing interests that could be perceived to bias this work—acknowledging all financial support and any other relevant financial or non-financial competing interests.<br><br>This statement **will appear in the published article** if the submission is accepted. Please make sure it is accurate. View published research articles from *PLOS ONE* for specific examples.	The authors have declared that no competing interests exist.

Powered by Editorial Manager® and ProduXion Manager® from Aries Systems Corporation

**NO authors have competing interests**<br><br>Enter: *The authors have declared that no competing interests exist.*<br><br>**Authors with competing interests**<br><br>Enter competing interest details beginning with this statement:<br><br>*I have read the journal's policy and the authors of this manuscript have the following competing interests: [insert competing interests here]*<br><br>* typeset	
**Ethics Statement**<br><br>Enter an ethics statement for this submission. This statement is required if the study involved:<br><br>• Human participants<br>• Human specimens or tissue<br>• Vertebrate animals or cephalopods<br>• Vertebrate embryos or tissues<br>• Field research<br><br>Write "N/A" if the submission does not require an ethics statement.<br><br>General guidance is provided below. Consult the submission guidelines for detailed instructions. **Make sure that all information entered here is included in the Methods section of the manuscript.**	A Scientific-academic license from the National System of Conservation Areas on behalf of the Ministry of Environment and Energy, N. ACTo-050-18 was attached in the submission in system. It was also specified at the beginning of the Methods section, as required.

Powered by Editorial Manager® and ProduXion Manager® from Aries Systems Corporation

**Format for specific study types**<br><br>**Human Subject Research (involving human participants and/or tissue)**<br>• Give the name of the institutional review board or ethics committee that approved the study<br>• Include the approval number and/or a statement indicating approval of this research<br>• Indicate the form of consent obtained (written/oral) or the reason that consent was not obtained (e.g. the data were analyzed anonymously)<br><br>**Animal Research (involving vertebrate animals, embryos or tissues)**<br>• Provide the name of the Institutional Animal Care and Use Committee (IACUC) or other relevant ethics board that reviewed the study protocol, and indicate whether they approved this research or granted a formal waiver of ethical approval<br>• Include an approval number if one was obtained<br>• If the study involved *non-human primates*, add *additional details* about animal welfare and steps taken to ameliorate suffering<br>• If anesthesia, euthanasia, or any kind of animal sacrifice is part of the study, include briefly which substances and/or methods were applied<br><br>**Field Research**<br><br>Include the following details if this study involves the collection of plant, animal, or other materials from a natural setting:<br>• Field permit number<br>• Name of the institution or relevant body that granted permission	
**Data Availability**<br><br>Authors are required to make all data underlying the findings described fully available, without restriction, and from the time of publication. PLOS allows rare exceptions to address legal and ethical concerns. See the PLOS Data Policy and FAQ for detailed information.	Yes - all data are fully available without restriction

A Data Availability Statement describing where the data can be found is required at submission. Your answers to this question constitute the Data Availability Statement and **will be published in the article**, if accepted.<br><br>**Important:** Stating 'data available on request from the author' is not sufficient. If your data are only available upon request, select 'No' for the first question and explain your exceptional situation in the text box.<br><br>Do the authors confirm that all data underlying the findings described in their manuscript are fully available without restriction?	
**Describe where the data may be found in full sentences. If you are copying our sample text, replace any instances of XXX with the appropriate details.**<br><br>• If the data are **held or will be held in a public repository**, include URLs, accession numbers or DOIs. If this information will only be available after acceptance, indicate this by ticking the box below. For example: *All XXX files are available from the XXX database (accession number(s) XXX, XXX.*).<br>• If the data are all contained **within the manuscript and/or Supporting Information files**, enter the following: *All relevant data are within the manuscript and its Supporting Information files.*<br>• If neither of these applies but you are able to provide **details of access elsewhere**, with or without limitations, please do so. For example:<br><br>*Data cannot be shared publicly because of [XXX]. Data are available from the XXX Institutional Data Access / Ethics Committee (contact via XXX) for researchers who meet the criteria for access to confidential data.*<br><br>*The data underlying the results presented in the study are available from (include the name of the third party*	All data and code files are available from the public repo https://github.com/malfaro2/Mora_et_al. Following the suggestion from one reviewer we tried to upload the repository contents in a zip file to the submission system, but it seems that the file size exceeds the limit (zip file is 86 MB). The GitHub repository is updated.

Powered by Editorial Manager® and ProduXion Manager® from Aries Systems Corporation

and contact information or URL).<br>• This text is appropriate if the data are owned by a third party and authors do not have permission to share the data.<br><br>* typeset	
Additional data availability information:	

Powered by Editorial Manager® and ProduXion Manager® from Aries Systems Corporation

Manuscript in .pdf Click here to access/download;Manuscript;Manuscript.pdf

Spectral measure of color variation of black - orange - black (BOB) pattern in small parasitic wasps (Hymenoptera: Scelionidae), a statistical approach

Rebeca Mora-Castro[1,2,3*],Marcela Hernandez-Jimenez[2,4],Marcela Alfaro-Córdoba[5,6],Esteban Avendaño-Soto[2,4], and Paul Hanson-Snortum[3]

1 Centro de Investigación en Biología Celular y Molecular, Universidad de Costa Rica, San José, Costa Rica.
2 Centro de Investigación en Ciencia e Ingeniería de Materiales, Universidad de Costa Rica, San José, Costa Rica.
3 Escuela de Biología, Universidad de Costa Rica, San José, Costa Rica.
4 Escuela de Física, Universidad de Costa Rica, San José, Costa Rica.
5 Centro de Investigación en Matemática Pura y Aplicada, Universidad de Costa Rica, San José, Costa Rica.
6 Escuela de Estadística, Universidad de Costa Rica, San José, Costa Rica.

* Corresponding author
E-mail: rebeca.mora@ucr.ac.cr (RMC)

Abstract

Small parasitic wasps are abundant and extremely diverse, yet their colors remain mostly uncharacterized. One of the more common color patterns observed in these wasps is a black-orange-black pattern, which is especially common among neotropical species of Scelionidae ranging in size from 2 to 10 mm. Due to the methodological challenges involved in extracting and characterizing pigments from small-sized insects, other methods for examining colors need to be explored. In this work, we propose the use of microspectrophotometry in combination with statistical analysis methods in order to study the spectral properties in such cases. We examined 8 scelionid genera and 1 genus from a distantly related family (Evaniidae), all showing the black-orange-black pattern. Functional Data Analysis and statistical analysis of Euclidean distances for color components were applied to study color differences both inter and intragenera. The Functional Data Analysis proved to be a better method for treating the reflectance data because it gave a better representation of the physical information. Also, the reflectance spectra were separated into spectral color component contributions and each component was labeled according to its own dominant wavelength at the maximum of the spectrum: Red, Green and Blue. When comparing spectral components curves, the spectral blue components of the orange and black colors, independent of the genera being compared, result almost identical, suggesting that there is a common compound for the pigments. The results also suggest that cuticle from different genera, but with the same color might have a similar chemical composition. This is the first time that the black and orange colors in small parasitic wasps has been characterized and our results provide a basis for future research on the color patterns of an abundant but neglected group of insects.

Introduction

The reflection, absorption or scattering of specific wavelengths of light by surface structures, the deposition of different chemical pigments in the outer body layers, or the interaction between various mechanisms such as pigments amplifying iridescence in butterflies [?], produce a remarkable variety of colors with complex and diverse patterns in insects. These color patterns play important roles in the biology of insects, for example as secondary sexual characters [?], in aposematism and mimicry [?, ?], and crypsis [?].

Compared with Lepidoptera and Coleoptera, there are relatively few studies of color patterns in the order Hymenoptera. Studies are mostly related to the yellow and black patterns in Vespidae [?, ?] and the variation in color patterns in *Bombus* [?]. In Hymenoptera there are also few studies regarding the chemical and physical nature of the color patterns exhibited, some of the pigments reported being pheomelanins (black, orange-red, yellow variations in velvet ants and bumble bees) [?], eumelanin (brown-colored cuticle) and xanthopterin (yellow-colored cuticle) in *Vespa orientalis* [?]. Various scelionid wasps [?], principally neotropical species ranging from 3 to 10 mm in length, show a recurring color pattern of black head, orange mesosoma, and black metasoma (BOB pattern; Fig ??), which has also been documented in species of numerous other families of Hymenoptera [?]. Several lines of evidence suggest that this color pattern has evolved many times independently within scelionids: the phylogenetically most basal scelionids [?] do not show this pattern; though quite common in the Neotropics only a minority of species show this pattern; species showing the BOB pattern occur in genera that are widely scattered in the phylogeny and not all species in these genera have this pattern [?]. This in turn strongly suggests that the BOB color pattern has some biological function but there are currently no studies addressing this question. Nor have the orange and black colors present in these wasps been characterized, which is the primary objective of the present investigation.

Fig 1. BOB Pattern Illustration. (A) Schematic detail of the area (dotted circles) used for measurements of both colors, zone of 1 mm2 of mesoscutum (1) and third tergite of metasoma (2). (B) Focus-stacked macro photography of a scelionid wasp with blak-orange-black color pattern obtained with a Reflex 850 camera coupled to a 20x microscope lens. The photograph at the far right, shows the striking black-orange-black color pattern of a group of scelionid specimens recently collected from the field; it was obtained with a Canon SX10 IS camera and a 105mm macro lens.

Color characterization of specimens, especially in taxonomy, has been done mostly subjectively, although more quantitative methods have been proposed [?]. There are few reports treating the measurement of color rigorously or analyzing spectral data and its variability as a functional measurement, which potentially can show differences detected by insects, and shed a light on their biology.

Among many studies that measure and compare colors in insects, the methods vary widely [?, ?, ?]. Endler et al. [?] took into account the continuous nature of the wavelengths as measures of color and used Principal Component Analysis (PCA) on the radiance spectra to analyze patterns of color, while recognizing its limitations, specifically, those related to the violation of assumptions for statistical tests to discriminate between colors. Functional Data Analysis (FDA) is a powerful method to study spectral data since it assumes that the underlying process generating the data is smooth, and hence provides tools to analyse a spectral measurement as a function and not as a series of data points [?]. For example, Rivas et al. [?] proposed the use of FDA techniques to test color changes in stone, and present a comparison with a multivariate method.

Materials and Methods

All experimental protocols were carried out in accordance with the involved institution's guidelines and regulations, thus, a scientific-academic license was obtained by the Ministry of Environment and Energy in relation to the National System of Conservation Areas, document N° ACTo-050-18, for the sampling and collection of the material under study.

A statistical approach based upon reflectance measurements of the dorsal surface of eight scelionid genera was used in order to study the BOB color pattern. The reflectance spectra were measured by means of a microspectrophotometer. A statistical description of the BOB color pattern was obtained by means of different statistical approaches, applied to the spectral information as a function of genus, color, spots of measurement and specimens. This approach was used due to the small size of these wasps and the difficulty involved in obtaining sufficient fresh material for chemical analysis of the pigments.

Microspectrophotometry

The reflectance spectra of 8 genera belonging to the Hymenopteran family Scelionidae were measured with a 508 PV UV-Visible-NIR Microscope Spectrophotometer (CRAIC, Los Angeles, USA). The spectrophotometer is coupled to an Eclipse LV100 ND Microscope using episcopic illumination (Nikon, Tokyo, Japan). A spot size area of 200μm x 200μm was measured. A spectralon standard was used as a white reference because of the diffuse reflection produced by the roughness of the surface of the cuticle. Table **??** includes further information regarding the measurement settings.

Table 1. Information about the analysis of color data.

**Microspectrophotometry technical details**	**Information**
Light source	12V-100W halogen lamp
Magnification	5X, CFI60 2 TU Plan Fluor BD, N.A 0.15, WD 18.0mm, (Nikon,Tokyo,Japan)
White reference	Spectralon Diffuse Reflectance Standard USRS-00-010(Labsphere, North Sutton,NH, USA)
Dark reference	Internal attenuators
Software for spectral capture	Lambda Fire (CRAIC,Los Angeles,USA)
Integration time	150 ms
Number of spectra averaged	100

Specimens and sampling for optical measurements

Recently collected specimens are crucial to ensure the good condition of the external cuticle structures. Since Scelionidae are small in size and their hosts (insect eggs) are difficult to find, collecting specimens requires considerable effort.

Specimens of 8 genera were collected: *Acanthoscelio*Ashmead, *Baryconus* Foerster, *Chromoteleia* Ashmead, *Macroteleia* Westwood,*Opisthacantha* Ashmead (*Lapitha* Ashmead is currently considered to be a junior synonym [**?**]), *Scelio* Latreille, *Sceliomorpha* Ashmead, *Triteleia* Kieffer. They were collected with entomological nets over a period of 12 months. Additionally some specimens of the genus *Evaniella* Bradley (Evaniidae) were included, because they exhibit a similar BOB pattern, but are quite far removed from scelionids in the phylogeny of Hymenoptera [**?**].

The collection site was a patch within the protected zone of El Rodeo, which is located in the Colon district of the San José province, between the geographical

coordinates 9º 52´ - 9º 56´ N and 84º 14´-84º 20´ W (Abra 3345 I and Río Grande 3345 IV cartographic sheets, National Geographic Institute 1989), at a distance of 18 km in a straight line from the capital city. The natural boundaries of the area are the Virilla River to the north, the Jaris river to the southeast and the Quebrada Honda river to the northeast. This natural reserve is composed of a secondary forest and a remnant of primary forest (approximately 200 ha). Specimens were identified by R.M. and P.H. using the keys to the scelionid genera [?] and voucher specimens were deposited in the Museum of Zoology of the University of Costa Rica . Due to the lack of taxonomic studies for six of the eight genera, species level identifications were not carried out . A previous study demonstrated that the vast majority hymenopterans with the BOB pattern, and those that are all black, do not show sexual dimorphism in color, at least in specimens that have been identified to species level in museum collections [?].

The specimens were killed by freezing and then, by using a needle cut and polished on both ends, one square millimeter or a small notch of area was dissected in the mesoscutum (orange) and in the third tergite of the metasoma (black) (Fig **??**). The orange and black samples of 4 specimens of each of the 8 genera were analyzed. For each specimen three samples were taken from the mesosoma (orange) and three from the metasoma (black).

Fig 2. Reflection functions. Functions $Z(\lambda)$ in y axis, for each color within each genus. Black lines show the functional mean per case.

Colorimetric differences

Fig **??** shows the spectral reflectance of the eight scelionid genera for the black and orange sections together with the distantly related genus, *Evaniella.* The data are presented with the average reflectance in black and the rough data showing the dispersion in gray and orange.

Regarding geometrical representation of color stimuli, the CIE color spaces are recognized to have important characteristics such as being device-independent and having a perceptual linearity [?]. Nevertheless, not all color spaces are suitable for comparing colors because distances between coordinates are not easily quantifiable due to the lack of uniformity of the space. For these effects, the CIE recommends using CIELAB color space due to its uniformity [?] . The corresponding coordinates are calculated using the procedure described in supplementary material S1.

The CIELAB color space is Euclidean, therefore distances between points can be used to represent approximately the perceived magnitude of color differences between object color stimuli of the same size and shape, viewed under similar conditions. The CIE 1976 L, a, b (CIELAB) color difference definition was adopted [?]:

$$\Delta E = \sqrt{\Delta L^2 + \Delta a^2 + \Delta b^2} \tag{1}$$

Here, the quantities ΔL, Δa, Δb, represent the subtraction of the corresponding coordinates of the two colors being compared and ΔE represents the distance between the two colors within the CIELAB space. There are other CIE definitions for color differences, see for example the ones explained in reference [?]. Nevertheless, as discussed by Melgosa [?], the definition in Eq **??** yields results that are not very different from more recent and complex definitions. Since the purpose of this work is not to evaluate the color space metrics, but to consolidate a quantitative framework to compare colors using the information available from microspectrophotometry, the CIE 1976 definition (Eq **??**) was chosen for the sake of simplicity. Regarding the definition of a color difference threshold, it can be argued that CIE color spaces are based on human

vision sensibility, and the color difference threshold will depend not only on the spectral region but also on the observer. Nevertheless, in order to establish a mean threshold value to define whether two colors are different, we used the value 2.3 as reported by [**?**] specifically for CIELAB. In this sense, when the color difference ΔE is less than this value, colors are considered as equal. Otherwise, colors are said to be different.

RGB spectral components

When calculating ΔE, all the spectral information is condensed into a single number and it is not clear whether the difference comes from shorter or longer wavelengths. In this sense, the use of a spectral space instead of a color space would provide a more objective description of the physical variation among stimuli and also allows the identification of different physical mechanisms producing the same perception of color but having different spectral behavior [**?**]. Therefore, in order to visualize the characteristics of the the reflectance curves in different regions of the electromagnetic spectrum, in this work, we propose a trichromatic spectral space defined as follows using the CIE color matching functions (CMFs):

$$R(\lambda) = \frac{\bar{x}(\lambda) Z(\lambda)}{\int \bar{x}(\lambda) Z(\lambda) d\lambda}, \tag{2}$$

$$G(\lambda) = \frac{\bar{y}(\lambda) Z(\lambda)}{\int \bar{y}(\lambda) Z(\lambda) d\lambda} \tag{3}$$

and

$$B(\lambda) = \frac{\bar{z}(\lambda) Z(\lambda)}{\int \bar{z}(\lambda) Z(\lambda) d\lambda}, \tag{4}$$

where $Z(\lambda)$ is the measured reflectance, $\bar{x}(\lambda)$, $\bar{y}(\lambda)$ and $\bar{z}(\lambda)$ are the CIE 1976 CMF's [**?**].

We label these components as Red, Green and Blue (RGB) because their maximum amplitudes occur at 600 nm, 555 nm and 446 nm respectively. .

It is recognized that spectral spaces have the advantage of providing information about the different processes generating phenotypic color diversity [**?**], and therefore we propose this RGB spectral component method as an alternative for the case when a direct chemical characterization can not be performed.

Statistical Analysis

Data sets

Each of the observations has 1185 recorded points: the reflectance (Y) as a function of wavelength value (λ), where λ is a sequence of 1185 equidistant points from 420.2 nm to 919.7 nm. The use of (λ) instead of the usual (s) in FDA, is done to match the representation of a wavelength in the physics literature. For the remainder of this paper, the 1185 points will be referred as reflectance curves, which were recorded in the following way: each curve was constructed with the reflectance of color in mesoscutum spots per colored area, and was measured three times in each specimen, in different spots. The observations correspond to the reflectance measured in the orange mesoscutum areas versus the black metasoma areas for 36 specimens: 4 specimens for each of the 8 genera, and 4 specimens for *Evaniella*, which was observed as a control (the latter genus belongs to a different family, which is not closely related to scelionids). The response variable is either a difference of reflectance or a measure of reflectance, and it can be measured in three different levels:

- The univariate mean measure: m_{ijk} where m is the mean difference between black and orange measures for all possible wavelengths in curve ijk, where k represents repetitions, j specimens, and i genera. This is is used as a naive base comparison.
- The functional measure: $Y_{ijk}(\lambda)$ where the difference in reflectance between black and orange curves $Z(\lambda)$ is represented by Y, and was measured for each wavelength λ, and repeated k times in each specimen j, and genera i. In this case, $Y_{ijk}(\lambda)$ describes a reflectance difference curve.
- The multivariate distance measure: a Euclidean distance ΔE as defined in **??** for each pair of curves being compared.

A table was created relating the different factors (spots, specimen, and genera) with the response in the univariate and functional case. In total, 96 combinations were recorded. In the multivariate distance case, a total of $N = C_2^{192} = 18336$ comparisons were used as observations, and then it was recorded whether or not each pair had a difference: genus, color, spot and specimen.

Data Analysis

Prior to the analysis, descriptive statistics were generated for each genus and color section (black and orange). The objective of the data analysis is to contrast the null hypothesis of no difference between color reflectance means of each combination of genus and color area, and for that, univariate and functional ANOVA were performed. The multivariate distance case was also analyzed to match the physics literature on color differences. The following methods were used in each case:

- Univariate method: A one-way ANOVA was used to compare the mean difference between black and orange for all possible bandwidths m_{ijk} with genus as the factor and controlling for specimen and spot.
- Functional method: A FANOVA [**?**] was used to compare the difference curves $Y_{ijk}(\lambda)$ with genus as the factor, and controlling for specimen and spot. The statistical F tests were included to interpret the contrast results for the mean difference curves. R packages erpFtest [**?**], and fdANOVA [**?**] were used.
- Multivariate distance method: A two-way ANOVA was used to compare the Euclidean distance ΔE between each combination of color and genus, using dichotomous variables as factors: color as 0 if the pair is from the same color, 1 if it differs; genus as 0 if the pair is from the same genus and 1 if it differs. Here also, specimen and spot were included as factors in the linear equation.

The objective is to illustrate the functional method compared to a univariate analysis approach, and with a multivariate distance alternative, to compare the results when taking into account all the information from the difference curves. More specifically, for the univariate and functional responses, the ANOVA model can be written as the following:

$$Y_{ijk} = \mu + \alpha_i + \beta_j + \epsilon_{ijk}, \tag{5}$$

for $i = 1, 2, ..., 8$ species, $j = 1, 2, ..., 4$ specimens, and $k = 1, 2, 3$ sampled spots in each colored area from each of the 4 specimens. Usual restrictions and assumptions for the ANOVA model apply:

$$\sum_{i=1}^{8} \alpha_i = 0, \qquad \sum_{j=1}^{4} \beta_j = 0, \qquad \epsilon_{ijk} \sim N(0, \sigma^2). \tag{6}$$

In the model, the global mean $\mu = 0$ given that we are working with difference curves, and α_i is the genera effect of level i and β_j is a block effect to explain the intra-genus variance . In order to account for the dependence between spots from the same specimens (in the two colored areas), a random effect η_k was tested, and different covariance structures for Σ_η were fitted (assuming dependence $\eta_k \sim N(0, \Sigma_\eta)$ or independence between spots). Lastly, ϵ_{ijk} is the residual accounting for the unexplained variation specific to the kth observation, ith genus and jth specimen. Depending on the response Y_{ijk}, the residuals ϵ_{ijk} are a number (univariate) or a curve (functional). Assumptions were tested for all the model options.

For the multivariate distances, the ANOVA model differed in the factors:

$$\Delta E_i = \mu + \gamma_i + \eta_i + \omega_i + \rho_i + \gamma_i * \eta_i + \epsilon_i, \quad (7)$$

where the global mean μ represents the average change for the N comparisons, and then each of the factors add the change given that the pair has different genera (γ_i), color (η_i), spots (ω_i) or specimens (ρ_i) for each pair i. Finally, ϵ_i is the error modeled as white noise. Assumptions were tested for this model as well.

Statistical analysis was performed using the computing environment R ([?]). The code and data to perform each of the tests mentioned in this paper are available for download in `https://github.com/malfaro2/Mora_et_al`. Data formatting and figures were prepared using the collection of R packages Tidyverse [?].

Results

The data provide support for reflectance difference variations between and within genera of the scelionids observed, when testing both the univariate data and the functional data (FDA method) (p-value < 0.00001 in all cases). The result is supported by the multivariate distance test, where there is evidence to say that overall change μ is greater than zero (p-value < 0.00001). Details about the significant factors that explain that change are summarized in the following sections.

Overall differences

Fig **??** presents descriptive statistics for measured spectra for each genus and each of the spectral colors. There are some genera with more variability, and there is a tendency to have low reflectance at lower wavelengths for both black and orange spots. It is important to point out that the measurements were performed grouping genera and not species, a factor that may account for the variability in some cases such as *Chromoteleia*. Because the spot size of 200μm x 200μm used in the reflectance measurements is more than 20 times larger that the features that comprise the mesosoma, the effect of topography of the cuticle and homogeneity of the pigment distribution can partly explain the data dispersion in some of the genera. See supplementary material **??**.

The difference curves for reflectance spectra, defined as $Y_{ijk}(\lambda)$ are plotted in Fig **??**. The patterns from Fig **??** appear more evident. For each genus, the behavior of the difference curves between black and orange can be described according to the wavelength in the following way. Before 550nm, differences are around zero, meaning that the spectra are similar in this region. Between 550nm and 750nm the differences are below zero and are similar between genera. And finally, for wavelengths greater than 750nm differences are both above and below zero and are dispersed with respect to genus. This analysis cannot be done if only the univariate measures are studied, given the information reduction as presented in the supplementary material **??**.

In order to test the null hypothesis of $H_0 : \alpha_i = 0$ for all genera, models were used to fit the data for each response. The best fit in all cases was from the models that assume

Fig 3. Difference (black-orange) of mean reflectance functions in each genus. Solid lines represent functional means, colored areas represent the area where difference functions in each genus are observed. A black dashed line represents the null hypothesis of no difference between colors in each genus. Curves above the black line mean that the black curves have higher reflectance values than the orange ones.

independence between spots on the same specimen, so linear models were used subsequently. The univariate (p-value < 0.0001) and functional (p-value < 0.0001) results coincide in that in at least one genus examined, the mean reflectance of the orange spot is significantly different from the mean reflectance of the black spot, controlling for specimen and spot.

The statistical tests confirm what the descriptive plots showed. For each genus, the difference between black and orange are significantly different from zero for both the univariate and functional (FDA) case. Results from the functional ANOVA and a permutation test based on a Fourier basis function representation, to test whether there is a mean difference between colors and genera, show that the calculated statistic is $T = 18.66961$ [**?**] with an associated p-value < 0.0001, which means that the difference between the black and orange spots in at least one genus is different from the difference between the black and orange spots in the other genera, as shown in Fig **??**. The univariate differences between genera shown in Table **??** only take into account the aggregated data collected at all wavelengths considered. The results for univariate comparison between genera can be found in Supplementary material **??**.

Table 2. Mean reflectance m_i for all possible bandwidths, by genus. Genera are ordered according to the difference magnitude. ** The univariate difference is not significantly different from zero. ^{a,b,c} Represent a group different from the rest, when doing univariate multiple comparisons. All analyses use $\alpha = 0.05$. Color representation corresponds to the CIE 1976 (L* a* b*) color space coordinates calculated from the mean reflectance curves from Fig **??**.

**Genera**	**Black**	**Orange**	**Absolute Difference**
Baryconus	29.5307	33.692	$4.1613^{**,a}$
Opisthacantha	29.5492	33.7317	$4.1825^{**,a}$
Scelio	29.3678	33.8015	$4.4337^{**,a}$
Acanthoscelio	29.2331	34.3834	5.1503^{a}
Chromoteleia	26.5133	33.3825	$6.8692^{a,c}$
Triteleia	24.0089	34.1785	$10.1696^{b,c}$
Sceliomorpha	30.683	41.7377	11.0547^{b}
Macroteleia	29.5722	41.3887	11.8165^{b}
Evaniella	21.2445	32.3144	11.0699^{b}

Multivariate Distance Analysis

ANOVA results for the model of Eq **??** presented in Table **??**, show how the overall average change in all comparisons is 5.0535 units with a standard error of 0.2642, which makes the average statistically greater than the 2.3 threshold value. The estimate of η is 26.2081, which means that keeping all the other factors constant, the average ΔE (the distance between the two colors within the CIELAB space) is 26 units greater than the overall mean when the curves differ in color. Also, if the curves differ in both color and genus, the overall mean ΔE is $5.0535 + 4.3540 + 26.2081 + -4.3814 = 31.2342$. The differences due to specimens are statistically not significant and the mean reflectance difference between measurements done in the same specimen but in different spots are

Fig 4. Violin and box plot comparison per genus. Distributions of ΔE calculated for pairs of curves of eight different genera: *Acanthoscelio* (AC), *Baryconus* (BA), *Chromoteleia* (CR), *Macroteleia* (MA), *Opisthacantha* (OP), *Scelio* (SC), *Sceliomorpha* (SM) and *Triteleia* (TR) and two scenarios: when they have the same color (in vermilion) and when their colors differ (bluish green). The dashed vermilion line represents the threshold of 2.3.

on average 0.85, which makes it statistically significant but negligible on average, compared to the threshold.

Table 3. ANOVA Results for model **??**.

	Estimate	Std. Error	t value	p-value
μ	5.0535	0.2642	19.12	0.0000
γ	4.3540	0.2420	17.99	0.0000
η	26.2081	0.3149	83.21	0.0000
ω	0.8524	0.1268	6.72	0.0000
ρ	-0.0425	0.1161	-0.37	0.7142
γ:η	-4.3814	0.3358	-13.05	0.0000

Given that it has been established that there is evidence of a difference between colors and genera in all analyses, there is a need to clarify which genera and which colors are making the difference. For that, a comparison matrix for ΔE was constructed and presented in two ways: comparisons between genera per color difference and comparisons between colors per genus difference. Such results are presented in Fig **??** and Fig **??** respectively. As an example, Fig **??** depicts how the distance ΔE between *Triteleia* and *Acanthoscelio* curves presents two distributions: one when both have the same color (in vermilion) and another when they have different colors (in bluish green). Both distributions present relatively low variability, and the first distribution is closer to zero, although its box - 50% of its data - is not close to zero or the threshold. In contrast, the distance ΔE between *Acanthoscelio* and *Acanthoscelio* curves for the same color and different specimens (in vermilion) clearly include the threshold and can be taken as not significantly different.

The difference between distributions is unclear when comparing colors. Fig **??** presents how in the case in which the colors differ (upper right corner) both distributions for ΔE are both clearly different from zero, while the distributions for ΔE of the curves with the same color are more difficult to distinguish from zero, especially if they are from the same genus (in vermilion).

Fig 5. Violin and box plot comparison per color. Distributions of ΔE calculated for pairs of two different colors, black and orange, and two scenarios: when they are the same genus (in vermilion) and when the genus differs (bluish green). The dashed vermilion line represents the threshold of 2.3.

The differences between curves can be described as an average of all ΔE, for each genus and color. By that metric, the maximum and minimum mean difference can be established, as described in Table **??** for comparisons between curves of different colors and Table **??** for comparisons between curves of the same color, pointing to the top ten comparisons with wider differences. Similarly, top ten comparisons of different genera and the same genus are presented in Table **??** and Table **??**, included in **??** in the supplementary material.

RGB Spectral Components

The comparison of the color component curves red ($R(\lambda)$), green ($G(\lambda)$) and blue ($B(\lambda)$), was done in terms of distance:

Table 4. Top 10 according to $\overline{\Delta E}$ for comparisons of curves of different color. The top 10 was extracted from a sample with overall minimum mean equal to 2.96 and maximum mean to 45.08. *Baryconus* (BA), *Chromoteleia* (CR), *Macroteleia* (MA), *Opisthacantha* (OP) and *Sceliomorpha* (SM).

	Genus 1	Genus 2	Color 1	Color 2	$\overline{\Delta E}$
1	CR	MA	BL	OR	45.08
2	MA	TR	OR	BL	44.79
3	MA	SM	OR	BL	43.59
4	OP	TR	OR	BL	41.51
5	CR	SM	BL	OR	41.25
6	SM	TR	OR	BL	41.22
7	MA	SC	OR	BL	41.02
8	CR	OP	BL	OR	40.84
9	SM	SM	BL	OR	39.71
10	BA	TR	OR	BL	39.69

Table 5. Top 10 according to $\overline{\Delta E}$ for comparisons of curves of the same color. The top 10 was extracted from a sample with overall minimum mean equal to 2.96 and maximum mean to 45.08. *Acanthoscelio* (AC), *Chromoteleia* (CR), *Macroteleia* (MA), *Opisthacantha* (OP) and *Sceliomorpha* (SM).

	Genus 1	Genus 2	Color 1	Color 2	$\overline{\Delta E}$
1	AC	MA	OR	OR	19.64
2	MA	TR	OR	OR	18.26
3	CR	MA	OR	OR	17.02
4	AC	OP	OR	OR	16.95
5	AC	SM	OR	OR	15.77
6	AC	BA	OR	OR	14.35
7	OP	TR	BL	BL	14.16
8	SM	TR	OR	OR	13.69
9	OP	MA	OR	OR	13.61
10	CR	OP	BL	BL	13.32

$$Distance = \sum_{\lambda} |R_c(\lambda) - Rc'(\lambda)|$$

for each pair of comparisons (c, c'). The results in Fig **??** show how the same differences depicted when using ΔE are very clear for the green and red components, but not for the blue component.

Panel (D) of Fig **??** shows the color component contributions for the *Acanthoscelio* and *Triteleia* genera for the black and orange measurements, respectively. The spectra were normalised by their own areas to illustrate the similarity of the contributions that are reflected in all data taken and presented in Fig **??** and **??** in panels A, B and C. Apart from the small changes in intensity for the normalised spectra, the maxima of each curve have a different dominant wavelength between genera, but the shape of the

Fig 6. Examples of violin and box plot comparison per genus and color component. Distributions of *Distance* for (A) green, (B) red and (C) blue components, calculated for pairs of curves of three different genera: *Acanthoscelio* (AC), *Baryconus* (BA) and *Triteleia* (TR), and two scenarios: when they have the same color (in vermilion) and when their colors differ (bluish green). Plot (D) is an example of how the R, G, and B curves look for *Acanthoscelio* (AC) and *Triteleia* (TR) in orange and black spots.

Fig 7. Scatter plot for color components per genus. Logarithm of the area under the curve, per component (red, green or blue) versus the corresponding wavelength in nm where the maximum occurs. Solid color points represent means for each genus and faded points correspond to independent observations. Plot (A) and (B) represent the components for the black and orange colors respectively.

curves was very similar. The main changes are the differences in area when not normalised.

Mapping color variants in a spectral space can yield information about the relationship between genotypic space and evolution [**?**]. In this context, in order to visualize the data provided by the spectral component analysis as a function of the genus, Fig **??** is presented. It shows the scatter plots for the black and orange measurements for all data, representing the logarithm of the area of each color component versus the dominant wavelength at the maxima of each contribution. The mean is plotted as a solid circle of the same color for each set of faded data points.

The range of wavelength values at the maxima for each color component contribution is interesting, being around 5 nm for the blue contribution in both black and orange measurements. For the green (black measurement) and red (orange measurement) components it is about 5 nm, but for the red (black measurement) and green (orange measurement) it is approximately 20 nm.

The genus *Evaniella* appears to separate from the group in the black measurements, but it is not that obvious for the orange measurements. Meanwhile, *Sceliomorpha* and *Macroteleia* group together for the green and red orange observations. The genus *Chromoteleia* is outside the main group in all three color components for the black observations and in the blue component of the orange observations. *Chromoteleia* and *Baryconus* also pair in the green and red components of the black and orange observations. Additionally *Sceliomorpha* and *Acanthoscelio* present a mean that is very close in the black measurements for the blue, green and red contributions. The genera *Opisthacantha* and *Triteleia* group together in the blue and green components for the orange contribution.

Discussion

The following discussion is focused mainly on the contribution of the different statistical approaches to the physical and biological interpretation of the data obtained following the procedures proposed, with the aim of establishing a quantitative analysis method for the study of cuticle color of insects. This section has two main parts: discussion about differences and suitability of the statistical methods and the interpretation of the physical results within a biological context.

Regarding the suitability of the statistical methods applied, the present study highlights the differences in terms of results that could arise using univariate methods, which ignore physical information contained in the reflectance measurement as a function of wavelength, versus analyzing data using FDA techniques.

The difference in intrageneric variances illustrated in Fig **??** of the Supplementary material **??**, confirms that the analysis of reflectance differences should not be done using univariate statistics. This variability is due to the difference in measurements across wavelengths: some sections are equal to zero, others are statistically different from zero, as described in the previous section. This can yield misleading results, finding univariate groups statistically similar, when the functional results are describing something different. In contrast, Functional Data Analysis (FDA) provides methods for analyzing data that are believed to arise from curves evaluated at a finite grid of points allowing the incorporation of spectral information from the reflectance curves [**?**].

In addition, a multivariate Euclidean distance defined in terms of a quantity with physical meaning such as ΔE, provides more evidence for analysis, besides confirming the FDA results. The details about multiple comparisons can be even more specific, if the color components are separated for each curve and the analysis is repeated.

The RGB component analysis showed that the nature of the differences found using the FDA and ΔE methods are related to the green and red color components, but no correlation was found for the blue component. This is consistent with results obtained qualitatively using the difference curves $Y_{ijk}(\lambda)$ shown in Fig **??**. Moreover, ANOVA results for model **??** (Table **??**) confirm the reproducibility of the experimental protocol and, for most of the cases studied, the results suggest that there is a low variability in the sample spectral measurements even though the taxonomic identification to just the genus level means that at least some of the genera could consist of more than one species.

This particular method for statistically testing for color differences differs from those in the literature due to the level of scrutiny. It goes from the general question, "Is there a difference between colors?" to more specific contrasts between genera and color, using both the FDA and the color components methods. In this way, we are statistically comparing each pair of colors and genera, controlling for the other experimental factors.

In general, the results of the spectral decomposition, together with the color differences as analyzed in Fig **??**, suggest that the cuticle segments from different genera, but with the same color (black vs. black, orange vs. orange) might have a similar chemical composition or an identical chemical composition with different concentrations. Tonal variations could be related to different amounts of an individual pigment or a combination of similar polymers distributed in the cuticle.

Recent studies report that the color phenotype is associated with the concentration and ratio of pigment forms [**?**, **?**], but other factors such as nanostrucutres of the integument can affect the expression of color [**?**]. In the case of pigments of different color (black vs. orange), the spectral blue components remain almost identical in terms of shape and characteristic wavelength (where the maximum occurs), suggesting that there is a common compound for the pigments. Notice that the normalization of the spectral components, equations (**??**), (**??**) and (**??**) allows the use of these features for comparison instead of intensity, which is related to the quantity of red, green or blue present in each color. The information given by the spectral components is important because optical properties are related to energetic processes due to electronic transitions when the pigment molecules interact with electromagnetic radiation. The shorter the wavelength, the more energetic the process is and therefore differences can be interpreted in terms of differences of electronic structure, which is related to the chemical composition of the pigments. A chemical characterization using infrared spectroscopy was attempted directly on the cuticle and results showed subtle differences suggesting a similar nature of the functional group attached to the molecule. Such results are not shown here because they are not conclusive due to the fact that the exact chemical composition of the cuticle for each color is unknown. A definitive identification of the pigment requires chemical extraction which at the moment could not be done because of the small amount of cuticle available.

Evaniella belongs to a completely different family (Evaniidae) and might therefore be expected to differ markedly from all the other genera. This indeed appears to be the case for its black color, but not its orange color (Fig **??**). This is an interesting result in that it might suggest that the orange color shows more convergent evolution than does the black color, but it remains to be demonstrated that the pigments are actually convergent. Nonetheless, within Scelionidae there are more differences in the orange color than in the black (Table **??**). Comparing the results shown in Fig **??** with a recent phylogenetic reconstruction of scelionid genera [**?**], a few other relationships between

spectral properties and phylogeny appear to be present. For example, *Baryconus* and *Chromoteleia* are paired in the green and red components for both black and orange, and these two genera are hypothesized to be closely related. *Opisthacantha* and *Triteleia* group together in the blue and green components of the orange coloration, and these two genera are also closely related. On the other hand, the orange color of *Macroteleia* and *Sceliomorpha* group together for the green and red components (Fig **??**B) despite these genera not being closely related. These results are intriguing and merit further investigation to determine whether certain spectral properties parallel the phylogeny.

In some other insects, contrasting black and orange color patterns are known to serve as aposematic (warning) coloration for potential predators [**?**] and it is possible that the same is true of the BOB pattern, although this has not yet been tested. However, the orange mesosoma becomes less common at higher elevations [**?**], suggesting that other factors such as thermoregulation (black absorbs more sunlight) might play a role at these elevations; it is also possible that predators or aposematic models vary with elevation.

There is obviously much more to be learned about the black and orange colors present in numerous parasitic wasps, but it is hoped that the results of the present spectral analyses provide a framework for future research, specially when a direct chemical characterization of the pigments is not possible and the optical and statistical methods proposed in this work can be applied.

Conclusions

The present study provides the first characterization of the common black-orange-black pattern that is found in small (3-10 mm) parasitic wasps. Because of their small size and difficulty in obtaining sufficient numbers of fresh specimens for pigment extraction, microspectrophotometry was used to obtain reflectance spectra for eight genera of scelionids. These reflectance data were analyzed by means of univariate analyses and by Functional Data Analysis, the latter proving to be superior because it gave a better representation of the physical information, given that it takes into account complete curves and not just summaries.

The RGB spectral components method is proposed for studying the origin of the color in the cases when a direct chemical composition is not possible. In the case of the genera studied, the spectral blue components of the orange and black color were found to be almost identical, suggesting that there is a common compound for the pigments. This result holds even when different genera were considered for comparison. Also spectral measures can and should be used for quantifying differences in biological color patterns since such analysis provides information that is not tinted by the illuminant.

A correlation between the mean values of characteristics of the color components was used in an attempt to group genera that show similar values and some, but not all, of these groupings are similar to their positions in a previously published phylogeny of the genera.

Further study, including a chemical characterization of the pigments and classification of specimens into species should be performed in order to obtain validation of these results.

Supporting information

S1 Color coordinates calculation

The tristimulus values for color coordinates, according to the CIE (International Commission on Illumination, or Commission Internationale d'Eclairage) definition, were

calculated as follows:

$$X = k \sum \phi_\lambda \bar{x}(\lambda) \Delta\lambda \quad (8)$$

$$Y = k \sum \phi_\lambda \bar{y}(\lambda) \Delta\lambda \quad (9)$$

$$Z = k \sum \phi_\lambda \bar{z}(\lambda) \Delta\lambda \quad (10)$$

where $\bar{x}(\lambda)$, $\bar{y}(\lambda)$ and $\bar{z}(\lambda)$ are the standardized color-matching functions (CMF) of the CIE 1931 standard observer. The constant k in equations (**??**), (**??**) and (**??**) is defined so that Y=100 for objects with perfect reflectivity. Finally, ϕ_λ is the relative color stimulus function, defined as:

$$\phi_\lambda = R(\lambda) S(\lambda) \quad (11)$$

with $R(\lambda)$being the spectral reflectance factor (the measure spectra) and $S(\lambda)$ is the relative spectral power distribution (SPD) of the illuminat.

These CIE-XYZ tristimulus values were calculated using the measured spectra (Fig **??**). A D- 65 day light standard illuminant for the CIE observer was chosen. Following the CIE recommendations, a spacing of 1 nm for wavelength was used. Since the data measured with the microspectrophotometer have a different wavelength spacing, an interpolation was made using the splinefun function in R [**?**], to generate a list of spectral points with the desired spacing.

After the tristimulus values were obtained, they were used to calculate the CIE 1976 (L* a* b*) (CIELAB) color space coordinates. The coordinates in the CIELAB color space are:

$$L = 116 f(Y/Y_n) - 16 \quad (12)$$

$$a = 500 [f(X/X_n) - f(Y/Y_n)] \quad (13)$$

$$b = 200 [f(X/X_n) - f(Z/Z_n)] \quad (14)$$

where

$$f(r) = r^{1/3} \text{ if } r > (24/116)^3 \quad (15)$$

and

$$f(r) = (841/108) r + 16/116 \text{ if } r \leq (24/116)^3 \quad (16)$$

with X_n, Y_n and Z_n being the tristimulus values of a idealized white reflector.

S2 Univariate comparison for the inter-genera case

For the univariate case, Table **??** shows how *Baryconus*, *Opisthacantha*, and *Scelio* absolute differences are not significantly different from zero. This contradicts the functional data analysis result presented before, where all the difference curves are different between genera.

When describing the difference between genera, Table **??** shows how there are three groups of genera without univariate mean differences: group *a* consists of *Baryconus*, *Opisthacantha*, *Scelio*, *Acanthoscelio* and *Chromoteleia*, while group *b* consist of *Triteleia*, *Sceliomorpha*, *Macroteleia* and *Evaniella* as members. Lastly, *Acanthoscelio* and *Chromoteleia* (group *c*) seem not to differ in terms of univariate means. On the other hand, when examining the curves in Fig **??**, it is clear that they differ, with *Triteleia*'s difference curve being very close to zero from $\lambda = 420$ nm to $\lambda = 590$ nm and then going to a negative difference of 20 around $\lambda = 780$ nm, while the curve for *Macroteleia* deviates from zero around $\lambda = 500$ nm and reaches -20 at $\lambda = 680$ nm. Given that other characteristics from the curve are compared in this case, it is reasonable to have a contradictory result. The pattern of contradictory results between

univariate and functional results is repeated in all pairs of genera belonging to the same univariate group (a,b,c), which could indicate one of the limitations of the univariate analysis: it ignores the fact that the spectrometer measurements are curves and they should be compared as such.

Fig **??** shows the box plots for univariate mean differences for each genus. The black dashed line represents the null hypothesis of no difference between colors within a genus.

Fig 8. Box plots for univariate mean differences per genus. Black dashed line represents the null hypothesis of no difference between colors per genus. *Acanthoscelio* (AC), *Baryconus* (BA), *Chromoteleia* (CR), *Macroteleia* (MA), *Opisthacantha* (OP), *Scelio* (SC), *Sceliomorpha* (SM), *Triteleia* (TR) and *Evaniella* (EV).

S3 Top 10 Tables

Table 6. Top 10 according to $\overline{\Delta E}$ for comparisons of curves of different genera. The top 10 was extracted from a sample with overall minimum mean equal to 2.96 and maximum mean to 45.08 *Acanthoscelio* (AC), *Baryconus* (BA), *Chromoteleia* (CR), *Macroteleia* (MA), *Opisthacantha* (OP) and *Sceliomorpha* (SM).

	Genera 1	Genera 2	Color 1	Color 2	$\overline{\Delta E}$
1	CR	MA	BL	OR	45.08
2	MA	TR	OR	BL	44.79
3	MA	SM	OR	BL	43.59
4	LA	TR	OR	BL	41.51
5	CR	SM	BL	OR	41.25
6	SM	TR	OR	BL	41.22
7	MA	SC	OR	BL	41.02
8	CR	LA	BL	OR	40.84
9	BA	TR	OR	BL	39.69
10	AC	MA	BL	OR	39.58

Table 7. Top 10 according to $\overline{\Delta E}$ for comparisons of curves of the same genera. The top 10 was extracted from a sample with overall minimum mean equal to 2.96 and maximum mean to 45.08. *Acanthoscelio* (AC), *Baryconus* (BA), *Chromoteleia* (CR), *Macroteleia* (MA), *Opisthacantha* (OP), *Scelio* (SC), *Sceliomorpha* (SM) and *Triteleia* (TR).

	Genera 1	Genera 2	Color 1	Color 2	$\overline{\Delta E}$
1	SM	SM	BL	OR	39.71
2	MA	MA	BL	OR	34.59
3	SC	SC	BL	OR	34.36
4	BA	BA	BL	OR	34.11
5	CR	CR	BL	OR	32.02
6	LA	LA	BL	OR	30.26
7	TR	TR	BL	OR	29.57
8	AC	AC	BL	OR	20.37
9	LA	LA	OR	OR	10.40
10	LA	LA	BL	BL	10.28

S4 Eosin hematoxylin technique

The specimens were fixed in 10% formalin, where they were preserved until they were processed. Subsequently, a post fixation in formaldehyde, acetic acid, ethanol solution was carried out for two hours. Dehydration was carried out in alcohols with increasing concentration (70%, 95%,100%). Clarification was implemented with xylol and later the sample was impregnated with paraffin. Once the specimens were processed, they were included in paraffin blocks. Continuous cuts were made at 4 microns in thickness and 3 or 4 cuts were placed per slide, using the entire mesoscutum. Hematoxylin and eosin stains, in a VENTANA HE 600 system, were performed on all sections. The final cuts were observed in an Olympus bx51 microscope and the photographs obtained with an Olympus Dp72 camera.

Fig **??** shows the variation in the distribution of the pigment together with the roughness of the cuticle surface. These factor contribute to the reflection of light in different directions and therefore to the dispersion of the data .

Fig 9. Eosin hematoxylin technique Cross section of the cuticle shows the pigment concentrated in the epicuticle, of a specimen of *Baryconus* with black-orange-black color (a) and black color (b). The variation in the distribution of the pigment together with the roughness of the cuticle surface can contribute to the reflection of light in different directions and therefore to the dispersion of the data

Acknowledgments

This work was inspired by personal conversations with Lubomir Masner. We would like to give special thanks to Mauricio Arce for the macro photography and to Juan Porras Peñaranda for his support in eosin-hematoxilin analysis. We thank three anonymous reviewers whose comments and suggestions helped improve and clarify this manuscript.

References

1. Rutowski RL, Macedonia JM, Morehouse N, Taylor-Taft L. Pterin pigments amplify iridescent ultraviolet signal in males of the orange sulphur butterfly, Colias eurytheme. Proceedings of the Royal Society B 2005, 272: 2329-2335. doi:10.1098/rspb.2005.3216

2. Garcia JA, Polidori C, Nieves-Aldrey JL. Pheomelanin in the secondary sexual characters of male parasitoid wasps (Hymenoptera: Pteromalidae). Arthropod Structure & Development. 2016;45(4):311–319. doi:10.1016/j.asd.2016.05.001.

3. Hines HM, Witkowski P, Wilson JS, Wakamatsu K. Melanic variation underlies aposematic color variation in two hymenopteran mimicry systems. PLOS ONE. 2017;12(7):e0182135. doi:10.1371/journal.pone.0182135.

4. Mallet J, Joron M. Evolution of diversity in warning color and mimicry: polymorphisms, shifting balance, and speciation. Annual Review of Ecology and Systematics. 1999;30(1):201–233. doi:10.1146/annurev.ecolsys.30.1.201.

5. Brakefield PM. Industrial melanism: Do we have the answers? Trends in Ecology & Evolution. 1987;2(5):117–122. doi:10.1016/0169-5347(87)90051-6.

6. Marchini M, Sommaggio D, Minelli A. Playing with black and yellow: the evolvability of a batesian mimicry. Evolutionary Biology. 2017;44(1):100–112. doi:10.1007/s11692-016-9397-0.

7. Vidal-Cordero JM, Moreno-Rueda G, López-Orta A, Marfil-Daza C, Ros-Santaella JL, Ortiz-Sánchez, FJ. Brighter-colored paper wasps (*Polistes dominula*) have larger poison glands. Frontiers in zoology. 2012;9(1):20.

8. Rapti Z, Duennes MA, Cameron SA. Defining the colour pattern phenotype in bumble bees(*Bombus*): a new model for evo devo. Biological Journal of the Linnean Society. 2014;113(2):384–404. doi:10.1111/bij.12356.

9. Plotkin M, Hod I, Zaban A, Boden SA, Bagnall DM, Galushko D. et al. Solar energy harvesting in the epicuticle of the oriental hornet (*Vespa orientalis*). Naturwissenschaften. 2010;97(12):1067–1076. doi:10.1007/s00114-010-0728-1.

10. Austin AD, Johnson, NF, Dowton, M. Systematics, evolution, and biology of scelionid and platygastrid wasps. Annu. Rev. Entomol.2005;50:553–582. doi:10.1146/annurev.ento.50.071803.130500

11. Mora R, Hanson PE. Widespread occurrence of black-orange-black color pattern in Hymenoptera. Journal of Insect Science. 2019;19(2). doi:10.1093/jisesa/iez021.

12. Murphy NP, Carey D, Castro LR, Dowton M, Austin A.D. Phylogeny of the platygastroid wasps (Hymenoptera) based on sequences from the 18S rRNA, 28S rRNA and cytochrome oxidase I genes: implications for the evolution of the ovipositor system and host relationships. Biological Journal of the Linnean Society.2007;91(4):653–669. doi:10.1111/j.1095-8312.2007.00825.x.

13. Aguiar A. An accurate procedure to describe colors in taxonomic works, with an example from Ichneumonidae (Hymenoptera). Zootaxa. 2005;1008(1):30–38. doi:10.11646/zootaxa.1008.1.4.

14. Stavenga DG, Leertouwer HL, Wilts BD. Quantifying the refractive index dispersion of a pigmented biological tissue using Jamin–Lebedeff interference microscopy. Light: Science & Applications. 2013;2(9):e100–e100. doi:10.1038/lsa.2013.56.

15. Skaldina O, Sorvari J. Not simply red: colouration of red wood ant *Formica rufa*(Hymenoptera: Formicidae) is polymorphic, modular and size-dependent. European Journal of Entomology. 2017;114:317–324. doi:10.14411/eje.2017.039.

16. Endler JA, Mielke PW. Comparing entire colour patterns as birds see them. Biological Journal of the Linnean Society. 2005;86(4):405–431. doi:10.1111/j.1095-8312.2005.00540.x

17. Ramsay J, Silverman, BW. Functional data analysis. 2nd ed. Springer Series in Statistics. New York: Springer-Verlag; 2005. Available from: `www.springer.com/gp/book/9780387400808`. doi:10.1007/B98888

18. Rivas T, Matías JM, Taboada J, Ordóñez C. Functional experiment design for the analysis of colour changes in granite using new L*a*b* functional colour coordinates. Journal of Computational and Applied Mathematics. 2011;235(16):4701–4716. doi:10.1016/j.cam.2010.08.005.

19. Masner L. Revisionary notes and keys to world genera of Scelionidae (Hymenoptera: Proctotrupoidea). The Memoirs of the Entomological Society of Canada.1976;108(S97):1-97 doi:10.4039/entm10897fv

20. Peters RS, Krogmann L, Mayer C, Donath A, Gunkel S, Meusemann K, et al. Evolutionary History of the Hymenoptera. Current Biology. 2017;27(7):1013–1018. doi:10.1016/j.cub.2017.01.027.

21. Tkalcic M, Tasic JF. Colour spaces: perceptual, historical and applicational background. vol. 1. IEEE;2003 doi:10.1109/EURCON.2003.1248032

22. Schanda J.(Ed). Colorimetry: understanding the CIE system. New Jersey: John Wiley & Sons; 2007. doi:

23. Melgosa M, Trémeau A, Cui G. Colour difference evaluation. Advanced color image processing and analysis New York: Springer New York; 2013. p. 59–79. doi:10.1007/978-1-4419-6190-7_3.

24. Mahy M, Van Eycken L, Oosterlinck A. Evaluation of uniform color spaces developed after the adoption of CIELAB and CIELUV. Color Research & Application. 1994;19(2):105–121. doi:10.1111/j.1520-6378.1994.tb00070.x

25. Renoult JP, Kelber A, Schaefer HM. Colour spaces in ecology and evolutionary biology Biological Reviews. 2017;92(1):291–315. doi:10.1111/brv.12230

26. Causeur D, Sheu CF, Chu MC, Rufini F. ERP: Significance analysis of event-related potentials data; 2018. Available from: https://CRAN.R-project.org/package=ERP.

27. Gorecki T, Smaga L. fdANOVA: Analysis of variance for univariate and multivariate functional data; 2018. Available from: https://CRAN.R-project.org/package=fdANOVA.

28. Wickham H. tidyverse: Easily Install and Load the 'Tidyverse' R package version 1.2.1. 2017. url:https://CRAN.R-project.org/package=tidyverse

29. Whibley AC, Langlade NB, Andalo C, Hanna AI, Bangham A, Thébaud C, Coen, E. Evolutionary paths underlying flower color variation in *Antirrhinum*. Science. 2006;313(5789):963–966. doi:10.1111/brv.12230

30. Galván I, Wakamatsu K. Color measurement of the animal integument predicts the content of specific melanin forms. RSC Advances.2016;6(82):79135–79142. doi:10.1039/C6RA17463A

31. Shawkey MD, D'Alba L. Interactions between colour-producing mechanisms and their effects on the integumentary colour palette. Philosophical Transactions of the Royal Society B: Biological Sciences. 2017;372(1724):20160536. doi:10.1098/rstb.2016.0536

32. Arenas LM, Walter D, Stevens M. Signal honesty and predation risk among a closely related group of aposematic species. Scientific Reports. 2015;5:11021. doi:10.1038/srep11021.

33. R Core Team RFfSC. R: A Language and Environment for Statistical Computing; 2018. Available from: http://www.R-project.org/.

Fig1

Click here to access/download;Figure;Fig1.tif

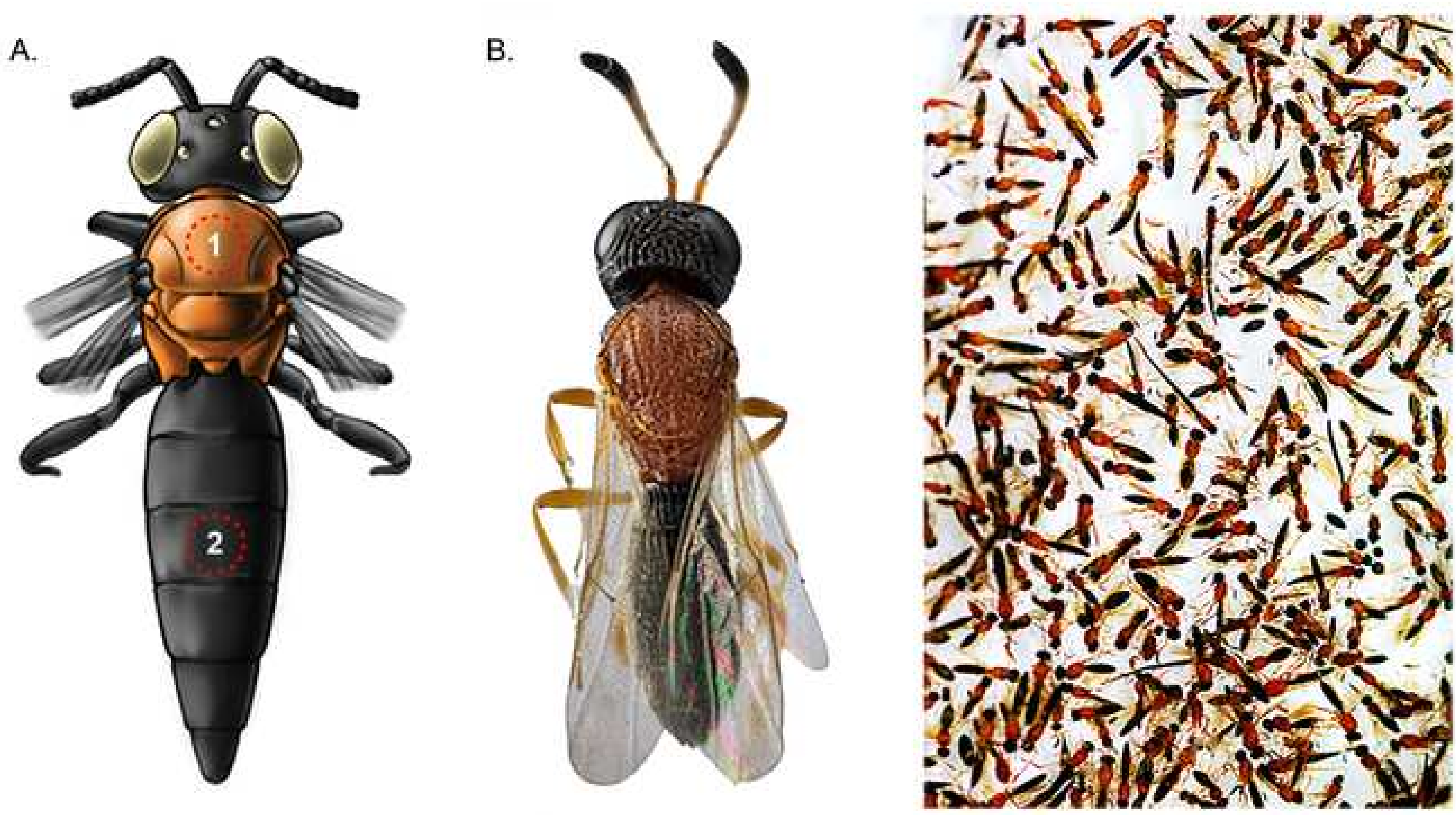

Fig2

Click here to access/download;Figure;Fig2.tif

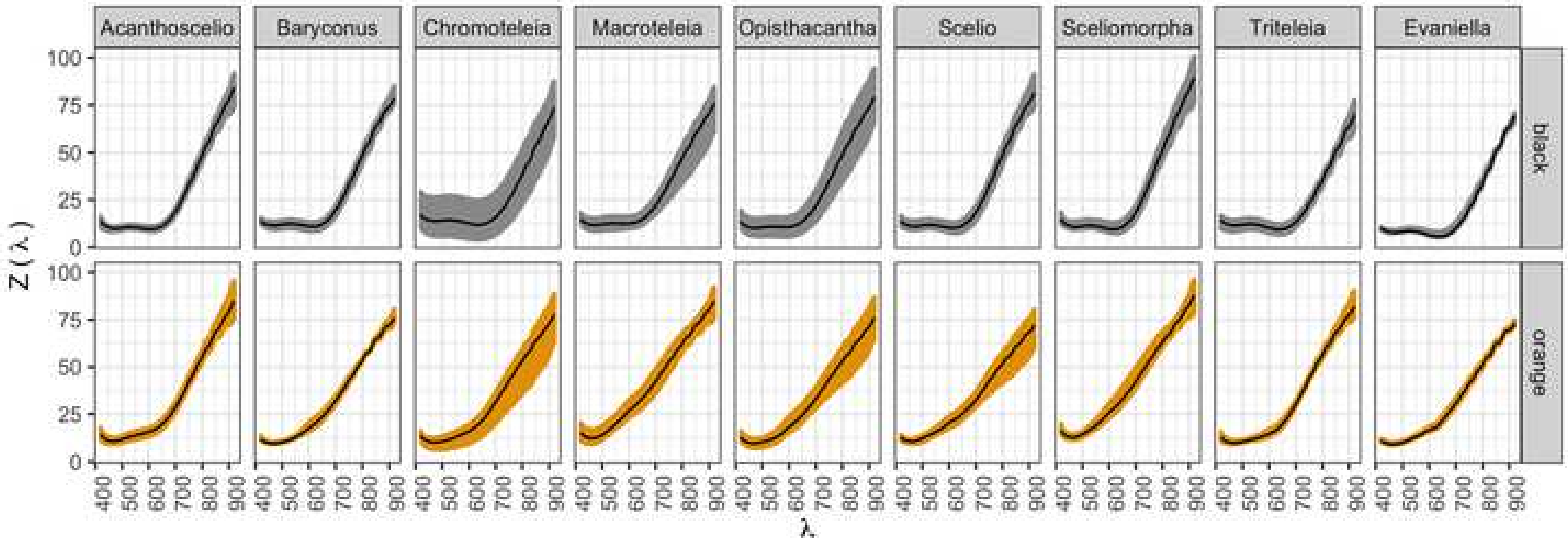

Fig3

Click here to access/download;Figure;Fig3.tif

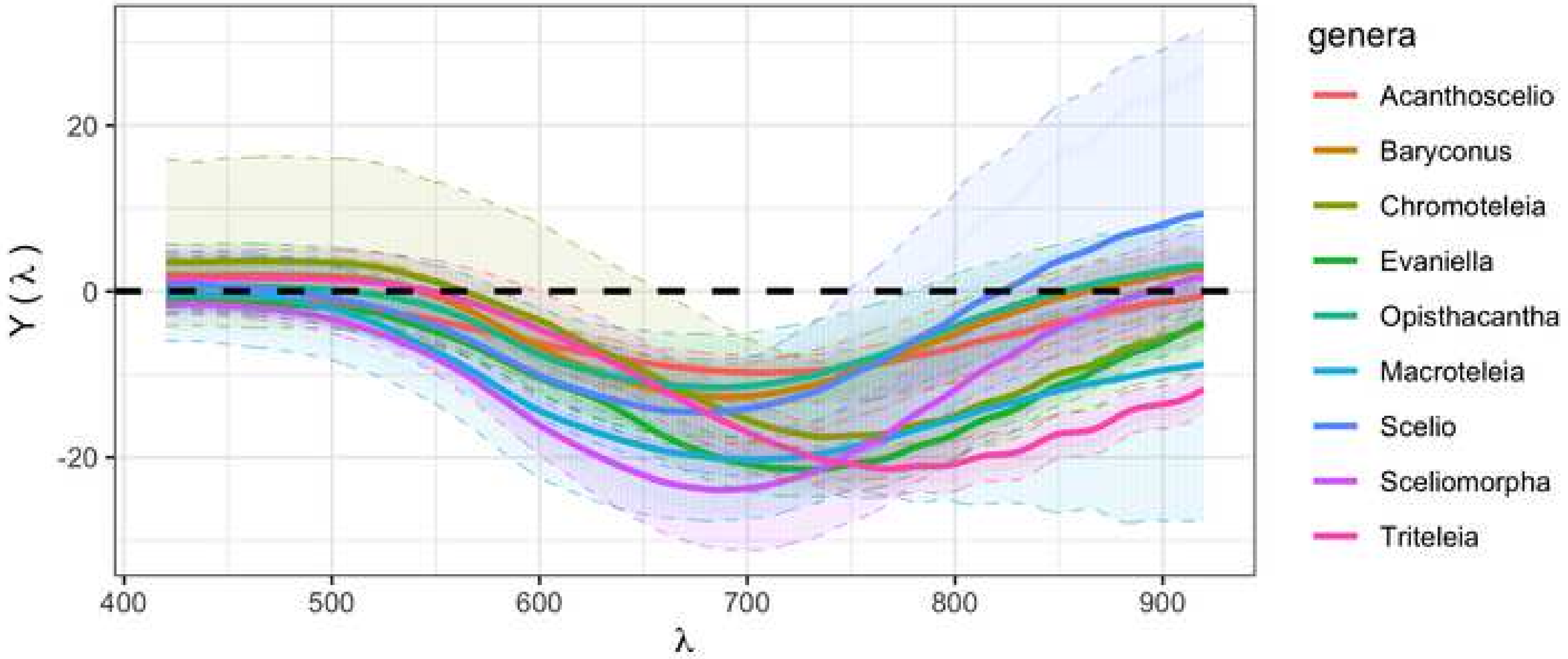

Fig4

Click here to access/download;Figure;Fig4.tif

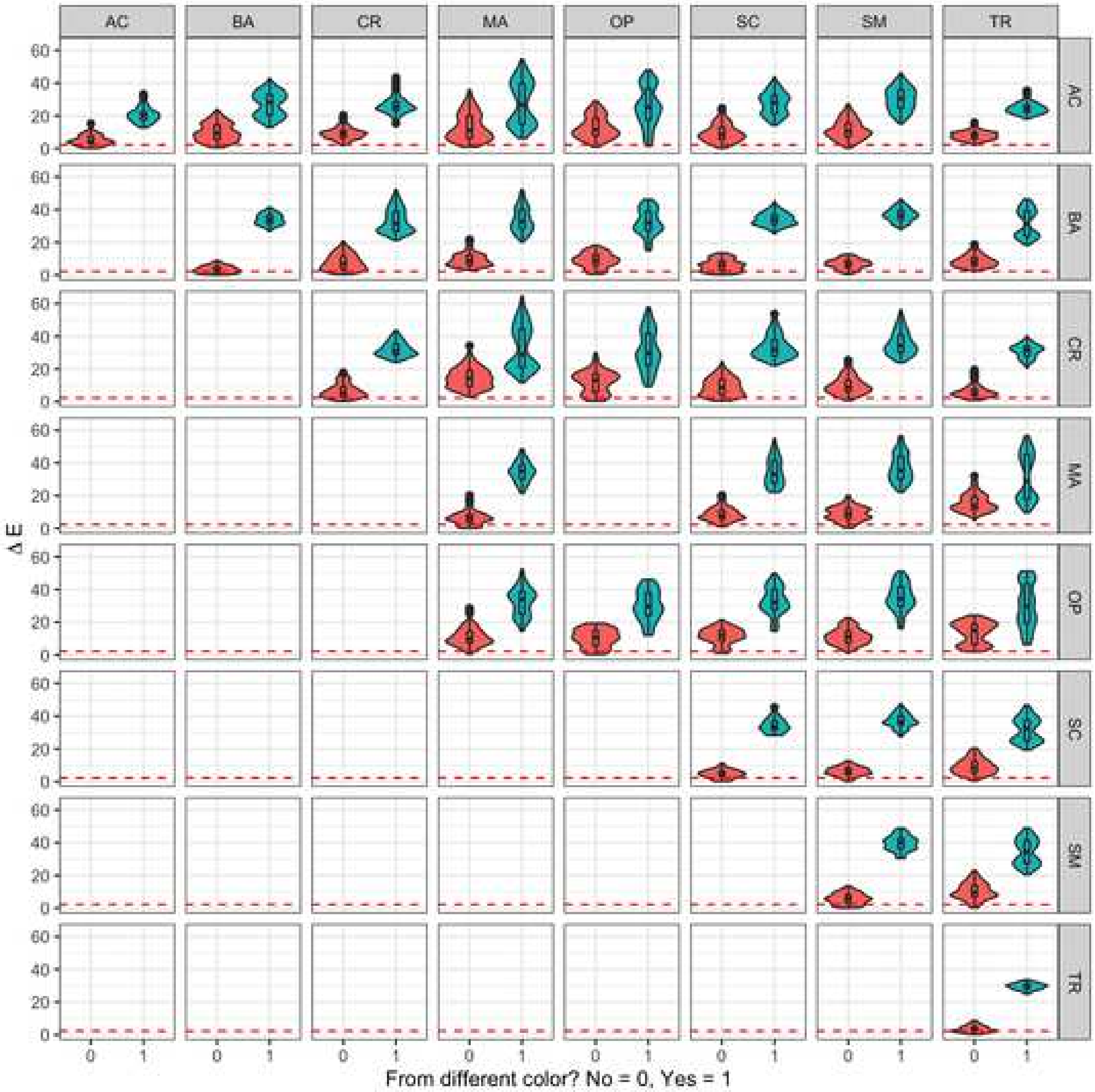

Fig5

Click here to access/download;Figure;Fig5.tif

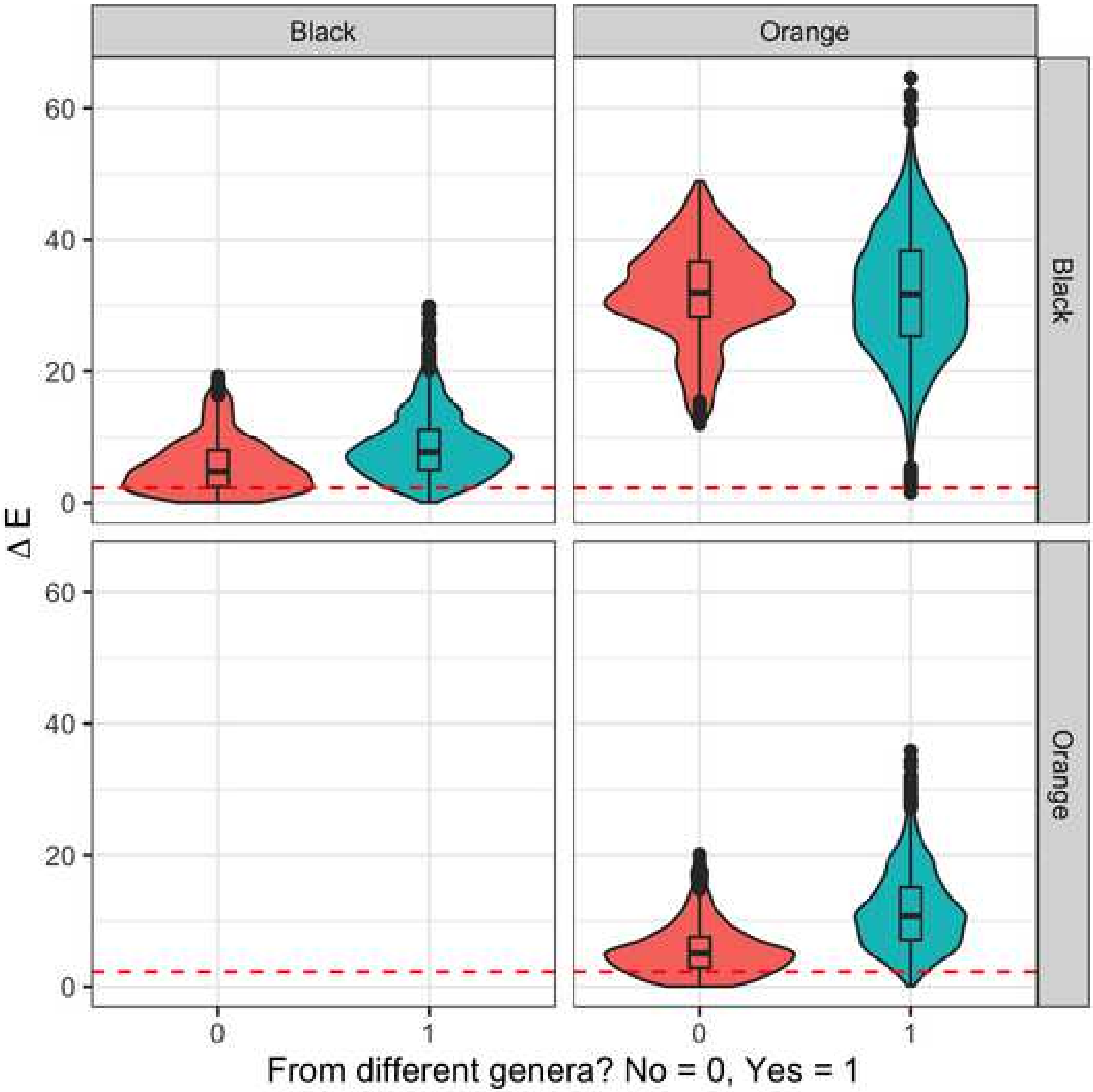

Fig6

Click here to access/download;Figure;Fig6.tif

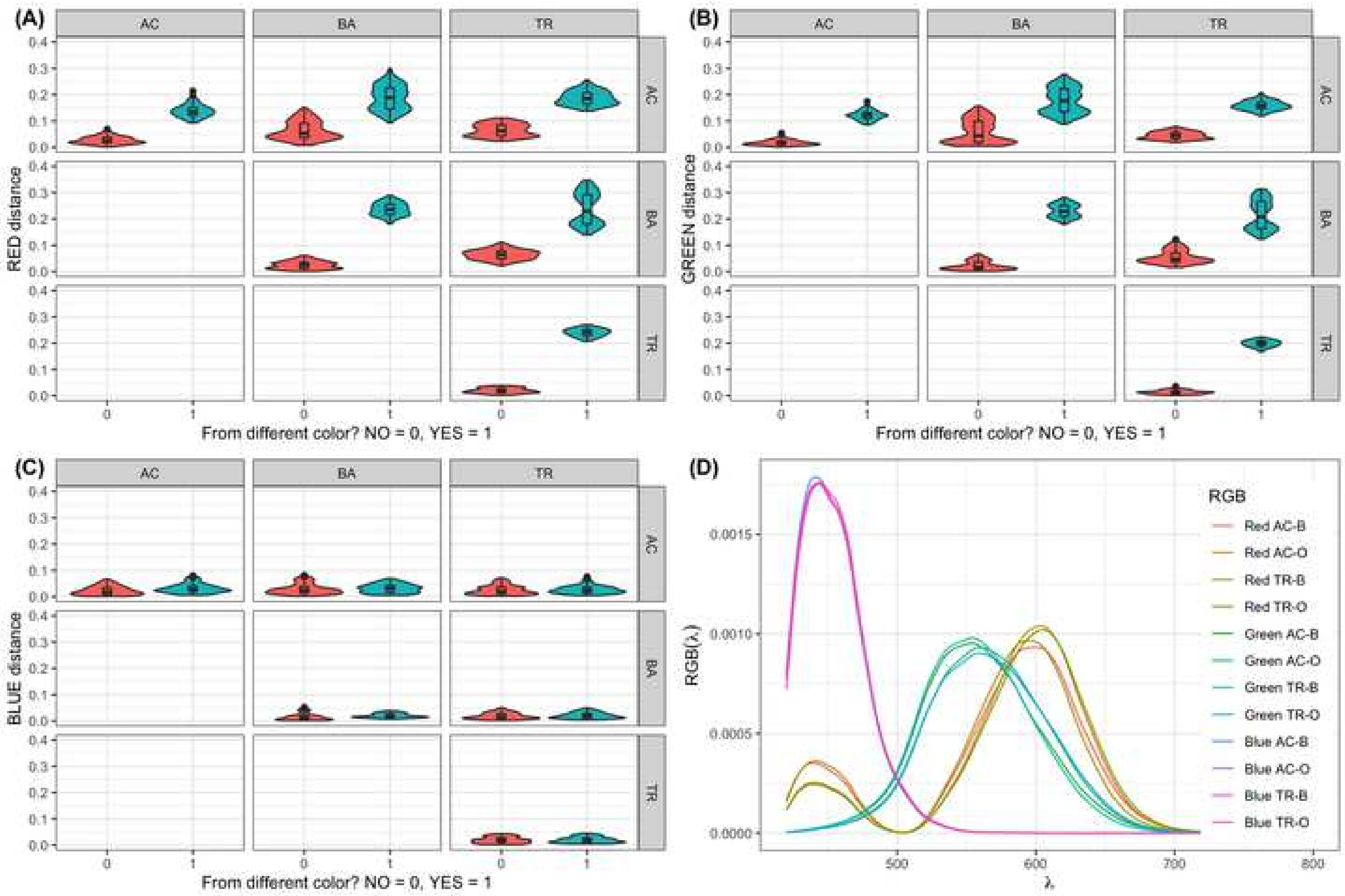

Fig7

Click here to access/download;Figure;Fig7.tif

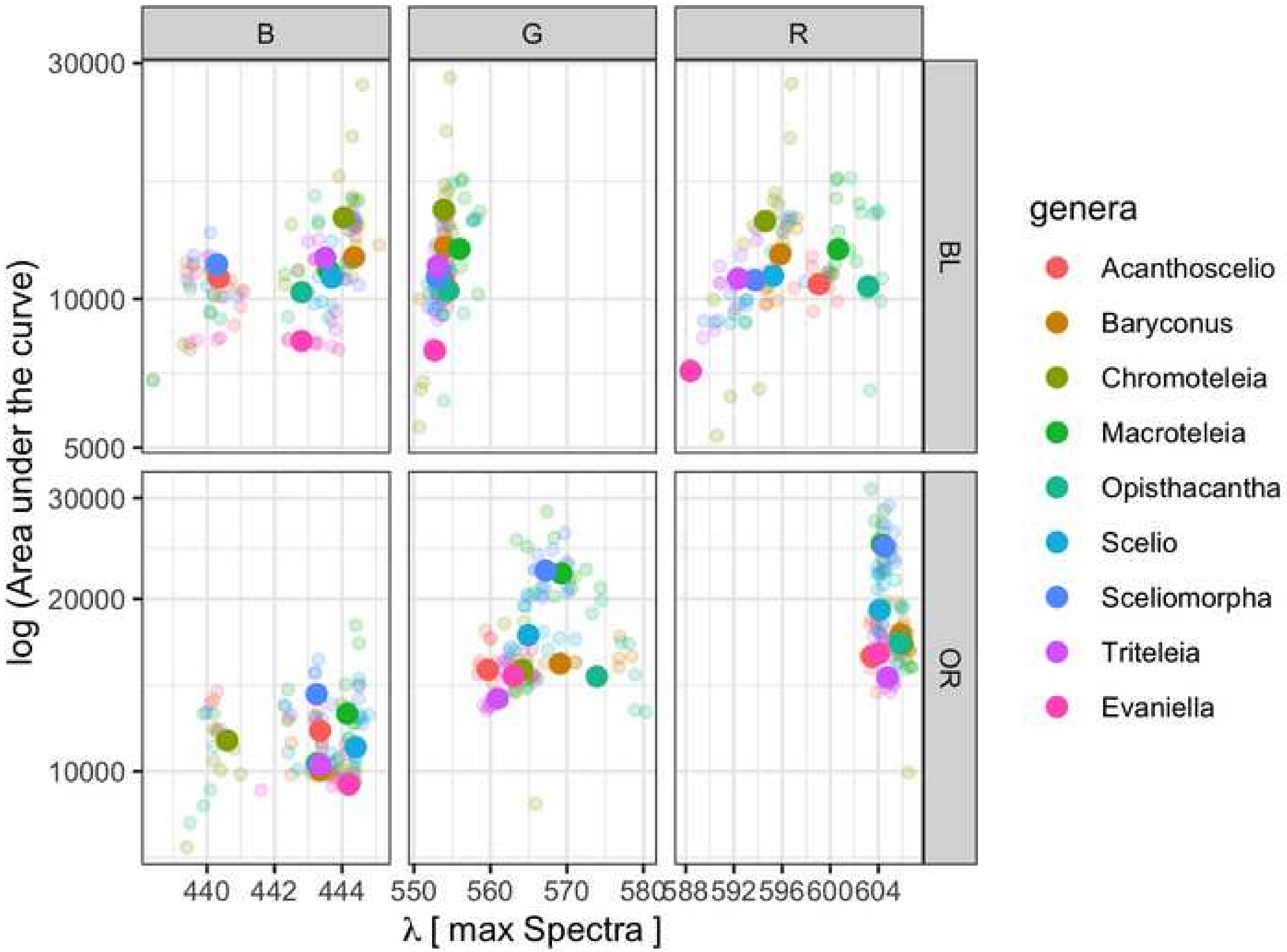

Click here to access/download;Figure;S2_Fig8.tif

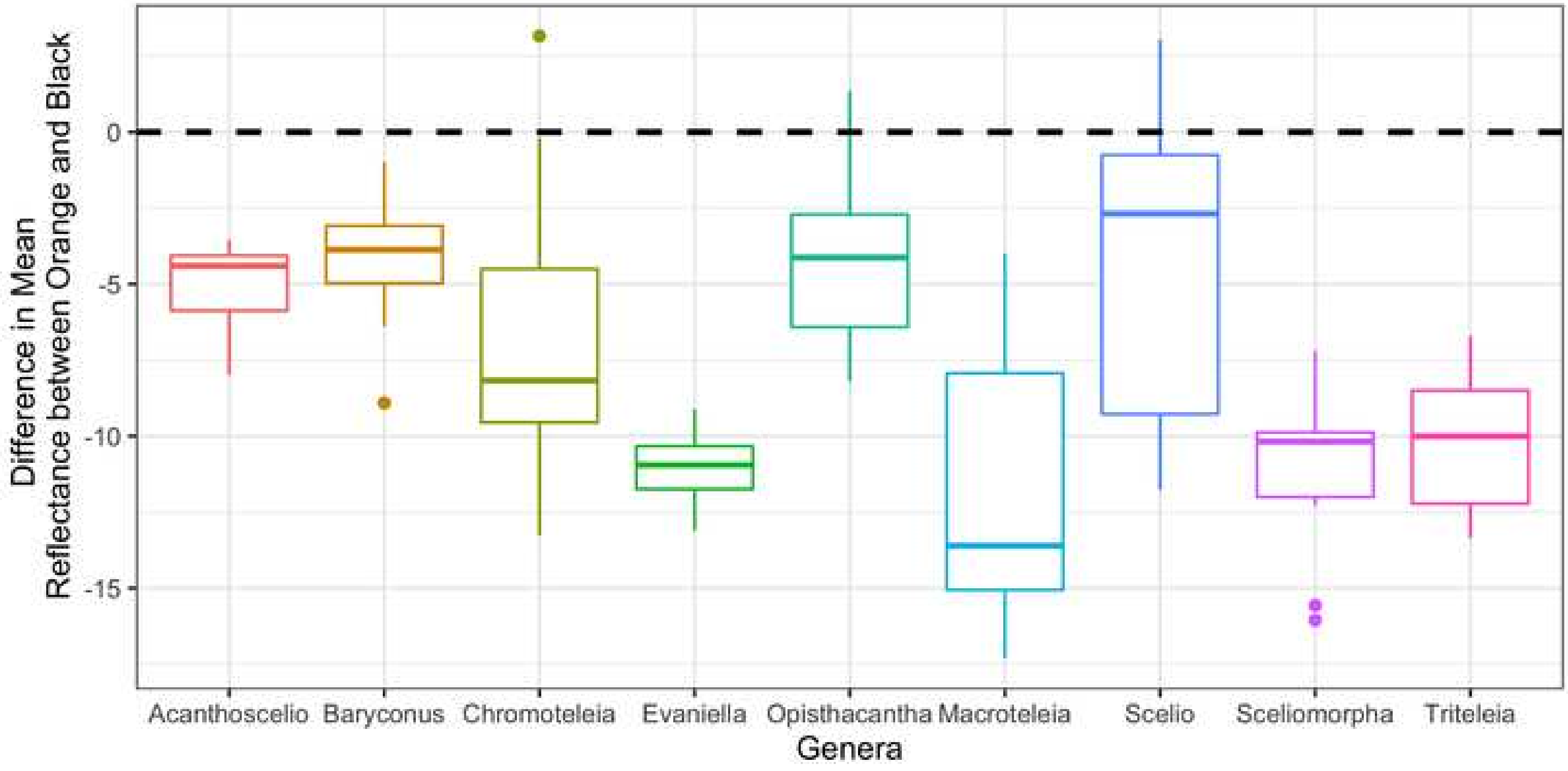

Click here to access/download;Figure;S4_Fig9.tif

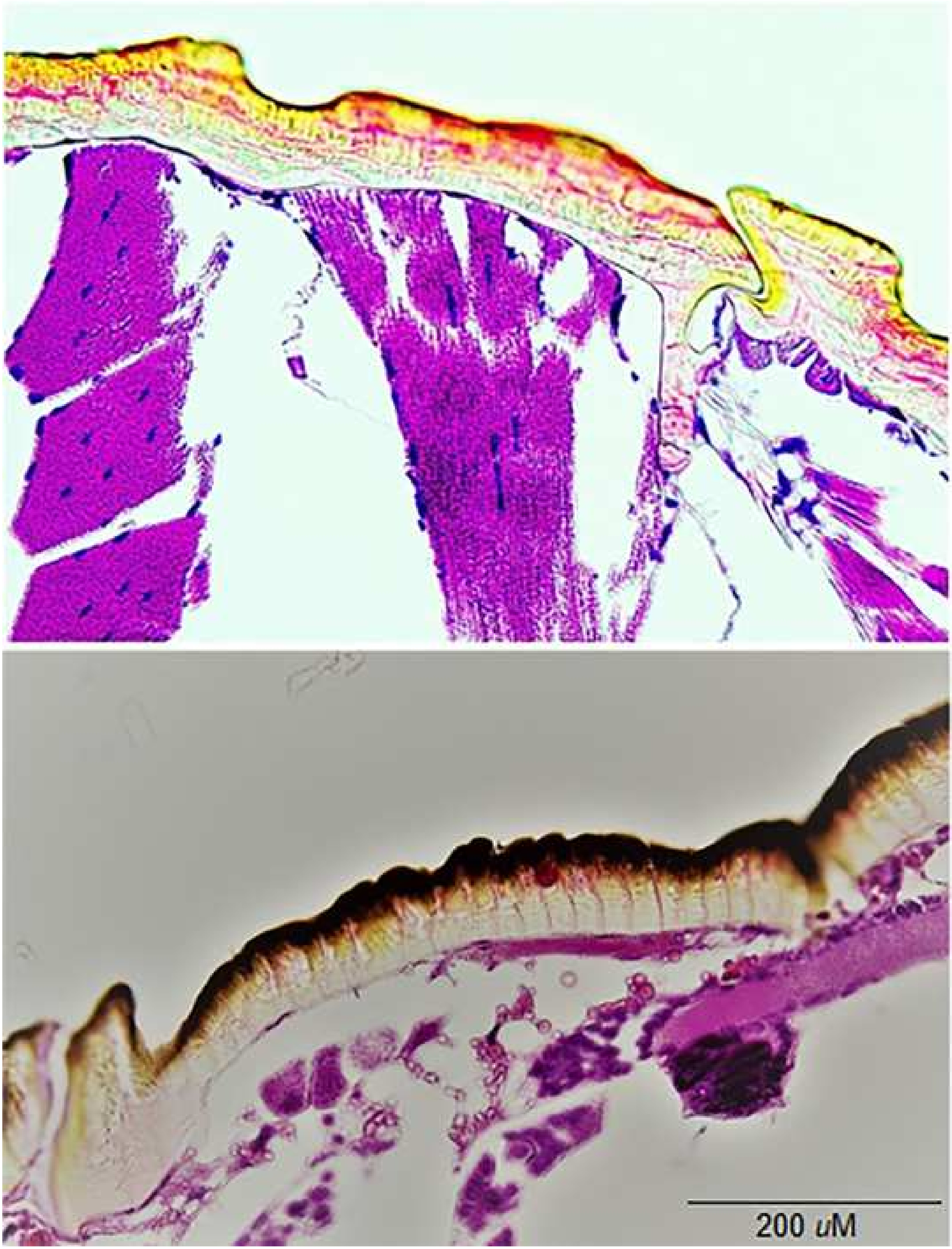

Click here to access/download
Supporting Information
Manuscript-linenumbering.pdf

Spectral measure of color variation of black - orange - black (BOB) pattern in small parasitic wasps (Hymenoptera: Scelionidae), a statistical approach

Rebeca Mora-Castro[1,2,3*],Marcela Hernandez-Jimenez[2,4],Marcela Alfaro-Córdoba[5,6],Esteban Avendaño-Soto[2,4], and Paul Hanson-Snortum[3]

1 Centro de Investigación en Biología Celular y Molecular, Universidad de Costa Rica, San José, Costa Rica.
2 Centro de Investigación en Ciencia e Ingeniería de Materiales, Universidad de Costa Rica, San José, Costa Rica.
3 Escuela de Biología, Universidad de Costa Rica, San José, Costa Rica.
4 Escuela de Física, Universidad de Costa Rica, San José, Costa Rica.
5 Centro de Investigación en Matemática Pura y Aplicada, Universidad de Costa Rica, San José, Costa Rica.
6 Escuela de Estadística, Universidad de Costa Rica, San José, Costa Rica.

* Corresponding author
E-mail: rebeca.mora@ucr.ac.cr (RMC)

Abstract

Small parasitic wasps are abundant and extremely diverse, yet their colors remain mostly uncharacterized. One of the more common color patterns observed in these wasps is a black-orange-black pattern, which is especially common among neotropical species of Scelionidae ranging in size from 2 to 10 mm. Due to the methodological challenges involved in extracting and characterizing pigments from small-sized insects, other methods for examining colors need to be explored. In this work, we propose the use of microspectrophotometry in combination with statistical analysis methods in order to study the spectral properties in such cases. We examined 8 scelionid genera and 1 genus from a distantly related family (Evaniidae), all showing the black-orange-black pattern. Functional Data Analysis and statistical analysis of Euclidean distances for color components were applied to study color differences both inter and intragenera. The Functional Data Analysis proved to be a better method for treating the reflectance data because it gave a better representation of the physical information. Also, the reflectance spectra were separated into spectral color component contributions and each component was labeled according to its own dominant wavelength at the maximum of the spectrum: Red, Green and Blue. When comparing spectral components curves, the spectral blue components of the orange and black colors, independent of the genera being compared, result almost identical, suggesting that there is a common compound for the pigments. The results also suggest that cuticle from different genera, but with the same color might have a similar chemical composition. This is the first time that the black and orange colors in small parasitic wasps has been characterized and our results provide a basis for future research on the color patterns of an abundant but neglected group of insects.

Introduction

The reflection, absorption or scattering of specific wavelengths of light by surface structures, the deposition of different chemical pigments in the outer body layers, or the interaction between various mechanisms such as pigments amplifying iridescence in butterflies [1], produce a remarkable variety of colors with complex and diverse patterns in insects. These color patterns play important roles in the biology of insects, for example as secondary sexual characters [2], in aposematism and mimicry [3, 4], and crypsis [5].

Compared with Lepidoptera and Coleoptera, there are relatively few studies of color patterns in the order Hymenoptera. Studies are mostly related to the yellow and black patterns in Vespidae [6, 7] and the variation in color patterns in *Bombus* [8]. In Hymenoptera there are also few studies regarding the chemical and physical nature of the color patterns exhibited, some of the pigments reported being pheomelanins (black, orange-red, yellow variations in velvet ants and bumble bees) [3], eumelanin (brown-colored cuticle) and xanthopterin (yellow-colored cuticle) in *Vespa orientalis* [9]. Various scelionid wasps [10], principally neotropical species ranging from 3 to 10 mm in length, show a recurring color pattern of black head, orange mesosoma, and black metasoma (BOB pattern; Fig 1), which has also been documented in species of numerous other families of Hymenoptera [11]. Several lines of evidence suggest that this color pattern has evolved many times independently within scelionids: the phylogenetically most basal scelionids [12] do not show this pattern; though quite common in the Neotropics only a minority of species show this pattern; species showing the BOB pattern occur in genera that are widely scattered in the phylogeny and not all species in these genera have this pattern [11]. This in turn strongly suggests that the BOB color pattern has some biological function but there are currently no studies addressing this question. Nor have the orange and black colors present in these wasps been characterized, which is the primary objective of the present investigation.

Fig 1. BOB Pattern Illustration. (A) Schematic detail of the area (dotted circles) used for measurements of both colors, zone of 1 mm2 of mesoscutum (1) and third tergite of metasoma (2). (B) Focus-stacked macro photography of a scelionid wasp with blak-orange-black color pattern obtained with a Reflex 850 camera coupled to a 20x microscope lens. The photograph at the far right, shows the striking black-orange-black color pattern of a group of scelionid specimens recently collected from the field; it was obtained with a Canon SX10 IS camera and a 105mm macro lens.

Color characterization of specimens, especially in taxonomy, has been done mostly subjectively, although more quantitative methods have been proposed [13]. There are few reports treating the measurement of color rigorously or analyzing spectral data and its variability as a functional measurement, which potentially can show differences detected by insects, and shed a light on their biology.

Among many studies that measure and compare colors in insects, the methods vary widely [7, 14, 15]. Endler et al. [16] took into account the continuous nature of the wavelengths as measures of color and used Principal Component Analysis (PCA) on the radiance spectra to analyze patterns of color, while recognizing its limitations, specifically, those related to the violation of assumptions for statistical tests to discriminate between colors. Functional Data Analysis (FDA) is a powerful method to study spectral data since it assumes that the underlying process generating the data is smooth, and hence provides tools to analyse a spectral measurement as a function and not as a series of data points [17]. For example, Rivas et al. [18] proposed the use of FDA techniques to test color changes in stone, and present a comparison with a multivariate method.

Materials and Methods

All experimental protocols were carried out in accordance with the involved institution's guidelines and regulations, thus, a scientific-academic license was obtained by the Ministry of Environment and Energy in relation to the National System of Conservation Areas, document N° ACTo-050-18, for the sampling and collection of the material under study.

A statistical approach based upon reflectance measurements of the dorsal surface of eight scelionid genera was used in order to study the BOB color pattern. The reflectance spectra were measured by means of a microspectrophotometer. A statistical description of the BOB color pattern was obtained by means of different statistical approaches, applied to the spectral information as a function of genus, color, spots of measurement and specimens. This approach was used due to the small size of these wasps and the difficulty involved in obtaining sufficient fresh material for chemical analysis of the pigments.

Microspectrophotometry

The reflectance spectra of 8 genera belonging to the Hymenopteran family Scelionidae were measured with a 508 PV UV-Visible-NIR Microscope Spectrophotometer (CRAIC, Los Angeles, USA). The spectrophotometer is coupled to an Eclipse LV100 ND Microscope using episcopic illumination (Nikon, Tokyo, Japan). A spot size area of 200μm x 200μm was measured. A spectralon standard was used as a white reference because of the diffuse reflection produced by the roughness of the surface of the cuticle. Table 1 includes further information regarding the measurement settings.

Table 1. Information about the analysis of color data.

Microspectrophotometry technical details	Information
Light source	12V-100W halogen lamp
Magnification	5X, CFI60 2 TU Plan Fluor BD, N.A 0.15, WD 18.0mm, (Nikon,Tokyo,Japan)
White reference	Spectralon Diffuse Reflectance Standard USRS-00-010(Labsphere, North Sutton,NH, USA)
Dark reference	Internal attenuators
Software for spectral capture	Lambda Fire (CRAIC,Los Angeles,USA)
Integration time	150 ms
Number of spectra averaged	100

Specimens and sampling for optical measurements

Recently collected specimens are crucial to ensure the good condition of the external cuticle structures. Since Scelionidae are small in size and their hosts (insect eggs) are difficult to find, collecting specimens requires considerable effort.

Specimens of 8 genera were collected: *Acanthoscelio*Ashmead, *Baryconus* Foerster, *Chromoteleia* Ashmead, *Macroteleia* Westwood,*Opisthacantha* Ashmead (*Lapitha* Ashmead is currently considered to be a junior synonym [19]), *Scelio* Latreille, *Sceliomorpha* Ashmead, *Triteleia* Kieffer. They were collected with entomological nets over a period of 12 months. Additionally some specimens of the genus *Evaniella* Bradley (Evaniidae) were included, because they exhibit a similar BOB pattern, but are quite far removed from scelionids in the phylogeny of Hymenoptera [20].

The collection site was a patch within the protected zone of El Rodeo, which is located in the Colon district of the San José province, between the geographical

coordinates 9º 52´ - 9º 56´ N and 84º 14´-84º 20´ W (Abra 3345 I and Río Grande 3345 IV cartographic sheets, National Geographic Institute 1989), at a distance of 18 km in a straight line from the capital city. The natural boundaries of the area are the Virilla River to the north, the Jaris river to the southeast and the Quebrada Honda river to the northeast. This natural reserve is composed of a secondary forest and a remnant of primary forest (approximately 200 ha). Specimens were identified by R.M. and P.H. using the keys to the scelionid genera [19] and voucher specimens were deposited in the Museum of Zoology of the University of Costa Rica. Due to the lack of taxonomic studies for six of the eight genera, species level identifications were not carried out. A previous study demonstrated that the vast majority hymenopterans with the BOB pattern, and those that are all black, do not show sexual dimorphism in color, at least in specimens that have been identified to species level in museum collections [11].

The specimens were killed by freezing and then, by using a needle cut and polished on both ends, one square millimeter or a small notch of area was dissected in the mesoscutum (orange) and in the third tergite of the metasoma (black) (Fig 2). The orange and black samples of 4 specimens of each of the 8 genera were analyzed. For each specimen three samples were taken from the mesosoma (orange) and three from the metasoma (black).

Fig 2. Reflection functions. Functions $Z(\lambda)$ in y axis, for each color within each genus. Black lines show the functional mean per case.

Colorimetric differences

Fig 2 shows the spectral reflectance of the eight scelionid genera for the black and orange sections together with the distantly related genus, *Evaniella*. The data are presented with the average reflectance in black and the rough data showing the dispersion in gray and orange.

Regarding geometrical representation of color stimuli, the CIE color spaces are recognized to have important characteristics such as being device-independent and having a perceptual linearity [21]. Nevertheless, not all color spaces are suitable for comparing colors because distances between coordinates are not easily quantifiable due to the lack of uniformity of the space. For these effects, the CIE recommends using CIELAB color space due to its uniformity [22] . The corresponding coordinates are calculated using the procedure described in supplementary material S1.

The CIELAB color space is Euclidean, therefore distances between points can be used to represent approximately the perceived magnitude of color differences between object color stimuli of the same size and shape, viewed under similar conditions. The CIE 1976 L, a, b (CIELAB) color difference definition was adopted [22]:

$$\Delta E = \sqrt{\Delta L^2 + \Delta a^2 + \Delta b^2} \quad (1)$$

Here, the quantities ΔL, Δa, Δb, represent the subtraction of the corresponding coordinates of the two colors being compared and ΔE represents the distance between the two colors within the CIELAB space. There are other CIE definitions for color differences, see for example the ones explained in reference [22]. Nevertheless, as discussed by Melgosa [23], the definition in Eq 1 yields results that are not very different from more recent and complex definitions. Since the purpose of this work is not to evaluate the color space metrics, but to consolidate a quantitative framework to compare colors using the information available from microspectrophotometry, the CIE 1976 definition (Eq 1) was chosen for the sake of simplicity. Regarding the definition of a color difference threshold, it can be argued that CIE color spaces are based on human

vision sensibility, and the color difference threshold will depend not only on the spectral region but also on the observer. Nevertheless, in order to establish a mean threshold value to define whether two colors are different, we used the value 2.3 as reported by [24] specifically for CIELAB. In this sense, when the color difference ΔE is less than this value, colors are considered as equal. Otherwise, colors are said to be different.

RGB spectral components

When calculating ΔE, all the spectral information is condensed into a single number and it is not clear whether the difference comes from shorter or longer wavelengths. In this sense, the use of a spectral space instead of a color space would provide a more objective description of the physical variation among stimuli and also allows the identification of different physical mechanisms producing the same perception of color but having different spectral behavior [25]. Therefore, in order to visualize the characteristics of the the reflectance curves in different regions of the electromagnetic spectrum, in this work, we propose a trichromatic spectral space defined as follows using the CIE color matching functions (CMFs):

$$R(\lambda) = \frac{\bar{x}(\lambda) Z(\lambda)}{\int \bar{x}(\lambda) Z(\lambda) d\lambda}, \tag{2}$$

$$G(\lambda) = \frac{\bar{y}(\lambda) Z(\lambda)}{\int \bar{y}(\lambda) Z(\lambda) d\lambda} \tag{3}$$

and

$$B(\lambda) = \frac{\bar{z}(\lambda) Z(\lambda)}{\int \bar{z}(\lambda) Z(\lambda) d\lambda}, \tag{4}$$

where $Z(\lambda)$ is the measured reflectance, $\bar{x}(\lambda)$, $\bar{y}(\lambda)$ and $\bar{z}(\lambda)$ are the CIE 1976 CMF's [22].

We label these components as Red, Green and Blue (RGB) because their maximum amplitudes occur at 600 nm, 555 nm and 446 nm respectively. .

It is recognized that spectral spaces have the advantage of providing information about the different processes generating phenotypic color diversity [25], and therefore we propose this RGB spectral component method as an alternative for the case when a direct chemical characterization can not be performed.

Statistical Analysis

Data sets

Each of the observations has 1185 recorded points: the reflectance (Y) as a function of wavelength value (λ), where λ is a sequence of 1185 equidistant points from 420.2 nm to 919.7 nm. The use of (λ) instead of the usual (s) in FDA, is done to match the representation of a wavelength in the physics literature. For the remainder of this paper, the 1185 points will be referred as reflectance curves, which were recorded in the following way: each curve was constructed with the reflectance of color in mesoscutum spots per colored area, and was measured three times in each specimen, in different spots. The observations correspond to the reflectance measured in the orange mesoscutum areas versus the black metasoma areas for 36 specimens: 4 specimens for each of the 8 genera, and 4 specimens for *Evaniella*, which was observed as a control (the latter genus belongs to a different family, which is not closely related to scelionids). The response variable is either a difference of reflectance or a measure of reflectance, and it can be measured in three different levels:

- The univariate mean measure: m_{ijk} where m is the mean difference between black and orange measures for all possible wavelengths in curve ijk, where k represents repetitions, j specimens, and i genera. This is is used as a naive base comparison.

- The functional measure: $Y_{ijk}(\lambda)$ where the difference in reflectance between black and orange curves $Z(\lambda)$ is represented by Y, and was measured for each wavelength λ, and repeated k times in each specimen j, and genera i. In this case, $Y_{ijk}(\lambda)$ describes a reflectance difference curve.

- The multivariate distance measure: a Euclidean distance ΔE as defined in 1 for each pair of curves being compared.

A table was created relating the different factors (spots, specimen, and genera) with the response in the univariate and functional case. In total, 96 combinations were recorded. In the multivariate distance case, a total of $N = C_2^{192} = 18336$ comparisons were used as observations, and then it was recorded whether or not each pair had a difference: genus, color, spot and specimen.

Data Analysis

Prior to the analysis, descriptive statistics were generated for each genus and color section (black and orange). The objective of the data analysis is to contrast the null hypothesis of no difference between color reflectance means of each combination of genus and color area, and for that, univariate and functional ANOVA were performed. The multivariate distance case was also analyzed to match the physics literature on color differences. The following methods were used in each case:

- Univariate method: A one-way ANOVA was used to compare the mean difference between black and orange for all possible bandwidths m_{ijk} with genus as the factor and controlling for specimen and spot.

- Functional method: A FANOVA [17] was used to compare the difference curves $Y_{ijk}(\lambda)$ with genus as the factor, and controlling for specimen and spot. The statistical F tests were included to interpret the contrast results for the mean difference curves. R packages erpFtest [26], and fdANOVA [27] were used.

- Multivariate distance method: A two-way ANOVA was used to compare the Euclidean distance ΔE between each combination of color and genus, using dichotomous variables as factors: color as 0 if the pair is from the same color, 1 if it differs; genus as 0 if the pair is from the same genus and 1 if it differs. Here also, specimen and spot were included as factors in the linear equation.

The objective is to illustrate the functional method compared to a univariate analysis approach, and with a multivariate distance alternative, to compare the results when taking into account all the information from the difference curves. More specifically, for the univariate and functional responses, the ANOVA model can be written as the following:

$$Y_{ijk} = \mu + \alpha_i + \beta_j + \epsilon_{ijk}, \tag{5}$$

for $i = 1, 2, ..., 8$ species, $j = 1, 2, ..., 4$ specimens, and $k = 1, 2, 3$ sampled spots in each colored area from each of the 4 specimens. Usual restrictions and assumptions for the ANOVA model apply:

$$\sum_{i=1}^{8} \alpha_i = 0, \qquad \sum_{j=1}^{4} \beta_j = 0, \qquad \epsilon_{ijk} \sim N(0, \sigma^2). \tag{6}$$

In the model, the global mean $\mu = 0$ given that we are working with difference curves, and α_i is the genera effect of level i and β_j is a block effect to explain the intra-genus variance . In order to account for the dependence between spots from the same specimens (in the two colored areas), a random effect η_k was tested, and different covariance structures for Σ_η were fitted (assuming dependence $\eta_k \sim N(0, \Sigma_\eta)$ or independence between spots). Lastly, ϵ_{ijk} is the residual accounting for the unexplained variation specific to the kth observation, ith genus and jth specimen. Depending on the response Y_{ijk}, the residuals ϵ_{ijk} are a number (univariate) or a curve (functional). Assumptions were tested for all the model options.

For the multivariate distances, the ANOVA model differed in the factors:

$$\Delta E_i = \mu + \gamma_i + \eta_i + \omega_i + \rho_i + \gamma_i * \eta_i + \epsilon_i, \quad (7)$$

where the global mean μ represents the average change for the N comparisons, and then each of the factors add the change given that the pair has different genera (γ_i), color (η_i), spots (ω_i) or specimens (ρ_i) for each pair i. Finally, ϵ_i is the error modeled as white noise. Assumptions were tested for this model as well.

Statistical analysis was performed using the computing environment R ([33]). The code and data to perform each of the tests mentioned in this paper are available for download in `https://github.com/malfaro2/Mora_et_al`. Data formatting and figures were prepared using the collection of R packages Tidyverse [28].

Results

The data provide support for reflectance difference variations between and within genera of the scelionids observed, when testing both the univariate data and the functional data (FDA method) (p-value < 0.00001 in all cases). The result is supported by the multivariate distance test, where there is evidence to say that overall change μ is greater than zero (p-value < 0.00001). Details about the significant factors that explain that change are summarized in the following sections.

Overall differences

Fig 2 presents descriptive statistics for measured spectra for each genus and each of the spectral colors. There are some genera with more variability, and there is a tendency to have low reflectance at lower wavelengths for both black and orange spots. It is important to point out that the measurements were performed grouping genera and not species, a factor that may account for the variability in some cases such as *Chromoteleia*. Because the spot size of 200μm x 200μm used in the reflectance measurements is more than 20 times larger that the features that comprise the mesosoma, the effect of topography of the cuticle and homogeneity of the pigment distribution can partly explain the data dispersion in some of the genera. See supplementary material S4.

The difference curves for reflectance spectra, defined as $Y_{ijk}(\lambda)$ are plotted in Fig 3. The patterns from Fig 2 appear more evident. For each genus, the behavior of the difference curves between black and orange can be described according to the wavelength in the following way. Before 550nm, differences are around zero, meaning that the spectra are similar in this region. Between 550nm and 750nm the differences are below zero and are similar between genera. And finally, for wavelengths greater than 750nm differences are both above and below zero and are dispersed with respect to genus. This analysis cannot be done if only the univariate measures are studied, given the information reduction as presented in the supplementary material S2.

In order to test the null hypothesis of $H_0 : \alpha_i = 0$ for all genera, models were used to fit the data for each response. The best fit in all cases was from the models that assume

Fig 3. Difference (black-orange) of mean reflectance functions in each genus. Solid lines represent functional means, colored areas represent the area where difference functions in each genus are observed. A black dashed line represents the null hypothesis of no difference between colors in each genus. Curves above the black line mean that the black curves have higher reflectance values than the orange ones.

independence between spots on the same specimen, so linear models were used subsequently. The univariate (p-value < 0.0001) and functional (p-value < 0.0001) results coincide in that in at least one genus examined, the mean reflectance of the orange spot is significantly different from the mean reflectance of the black spot, controlling for specimen and spot.

The statistical tests confirm what the descriptive plots showed. For each genus, the difference between black and orange are significantly different from zero for both the univariate and functional (FDA) case. Results from the functional ANOVA and a permutation test based on a Fourier basis function representation, to test whether there is a mean difference between colors and genera, show that the calculated statistic is $T = 18.66961$ [26] with an associated p-value < 0.0001, which means that the difference between the black and orange spots in at least one genus is different from the difference between the black and orange spots in the other genera, as shown in Fig 3. The univariate differences between genera shown in Table 2 only take into account the aggregated data collected at all wavelengths considered. The results for univariate comparison between genera can be found in Supplementary material S2.

Table 2. Mean reflectance m_i for all possible bandwidths, by genus. Genera are ordered according to the difference magnitude. ** The univariate difference is not significantly different from zero. ^{a,b,c} Represent a group different from the rest, when doing univariate multiple comparisons. All analyses use $\alpha = 0.05$. Color representation corresponds to the CIE 1976 (L* a* b*) color space coordinates calculated from the mean reflectance curves from Fig 1.

Genera	Black	Orange	Absolute Difference
Baryconus	29.5307	33.692	$4.1613^{**,a}$
Opisthacantha	29.5492	33.7317	$4.1825^{**,a}$
Scelio	29.3678	33.8015	$4.4337^{**,a}$
Acanthoscelio	29.2331	34.3834	5.1503^{a}
Chromoteleia	26.5133	33.3825	$6.8692^{a,c}$
Triteleia	24.0089	34.1785	$10.1696^{b,c}$
Sceliomorpha	30.683	41.7377	11.0547^{b}
Macroteleia	29.5722	41.3887	11.8165^{b}
Evaniella	21.2445	32.3144	11.0699^{b}

Multivariate Distance Analysis

ANOVA results for the model of Eq 7 presented in Table 3, show how the overall average change in all comparisons is 5.0535 units with a standard error of 0.2642, which makes the average statistically greater than the 2.3 threshold value. The estimate of η is 26.2081, which means that keeping all the other factors constant, the average ΔE (the distance between the two colors within the CIELAB space) is 26 units greater than the overall mean when the curves differ in color. Also, if the curves differ in both color and genus, the overall mean ΔE is $5.0535 + 4.3540 + 26.2081 + -4.3814 = 31.2342$. The differences due to specimens are statistically not significant and the mean reflectance difference between measurements done in the same specimen but in different spots are

Fig 4. Violin and box plot comparison per genus. Distributions of ΔE calculated for pairs of curves of eight different genera: *Acanthoscelio* (AC), *Baryconus* (BA), *Chromoteleia* (CR), *Macroteleia* (MA), *Opisthacantha* (OP), *Scelio* (SC), *Sceliomorpha* (SM) and *Triteleia* (TR) and two scenarios: when they have the same color (in vermilion) and when their colors differ (bluish green). The dashed vermilion line represents the threshold of 2.3.

on average 0.85, which makes it statistically significant but negligible on average, compared to the threshold.

Table 3. ANOVA Results for model 7.

	Estimate	Std. Error	t value	p-value
μ	5.0535	0.2642	19.12	0.0000
γ	4.3540	0.2420	17.99	0.0000
η	26.2081	0.3149	83.21	0.0000
ω	0.8524	0.1268	6.72	0.0000
ρ	-0.0425	0.1161	-0.37	0.7142
γ:η	-4.3814	0.3358	-13.05	0.0000

Given that it has been established that there is evidence of a difference between colors and genera in all analyses, there is a need to clarify which genera and which colors are making the difference. For that, a comparison matrix for ΔE was constructed and presented in two ways: comparisons between genera per color difference and comparisons between colors per genus difference. Such results are presented in Fig 4 and Fig 5 respectively. As an example, Fig 4 depicts how the distance ΔE between *Triteleia* and *Acanthoscelio* curves presents two distributions: one when both have the same color (in vermilion) and another when they have different colors (in bluish green). Both distributions present relatively low variability, and the first distribution is closer to zero, although its box - 50% of its data - is not close to zero or the threshold. In contrast, the distance ΔE between *Acanthoscelio* and *Acanthoscelio* curves for the same color and different specimens (in vermilion) clearly include the threshold and can be taken as not significantly different.

The difference between distributions is unclear when comparing colors. Fig 5 presents how in the case in which the colors differ (upper right corner) both distributions for ΔE are both clearly different from zero, while the distributions for ΔE of the curves with the same color are more difficult to distinguish from zero, especially if they are from the same genus (in vermilion).

Fig 5. Violin and box plot comparison per color. Distributions of ΔE calculated for pairs of two different colors, black and orange, and two scenarios: when they are the same genus (in vermilion) and when the genus differs (bluish green). The dashed vermilion line represents the threshold of 2.3.

The differences between curves can be described as an average of all ΔE, for each genus and color. By that metric, the maximum and minimum mean difference can be established, as described in Table 4 for comparisons between curves of different colors and Table 5 for comparisons between curves of the same color, pointing to the top ten comparisons with wider differences. Similarly, top ten comparisons of different genera and the same genus are presented in Table 6 and Table 7, included in S3 in the supplementary material.

RGB Spectral Components

The comparison of the color component curves red ($R(\lambda)$), green ($G(\lambda)$) and blue ($B(\lambda)$), was done in terms of distance:

Table 4. Top 10 according to $\overline{\Delta E}$ for comparisons of curves of different color. The top 10 was extracted from a sample with overall minimum mean equal to 2.96 and maximum mean to 45.08. *Baryconus* (BA), *Chromoteleia* (CR), *Macroteleia* (MA), *Opisthacantha* (OP) and *Sceliomorpha* (SM).

	Genus 1	Genus 2	Color 1	Color 2	$\overline{\Delta E}$
1	CR	MA	BL	OR	45.08
2	MA	TR	OR	BL	44.79
3	MA	SM	OR	BL	43.59
4	OP	TR	OR	BL	41.51
5	CR	SM	BL	OR	41.25
6	SM	TR	OR	BL	41.22
7	MA	SC	OR	BL	41.02
8	CR	OP	BL	OR	40.84
9	SM	SM	BL	OR	39.71
10	BA	TR	OR	BL	39.69

Table 5. Top 10 according to $\overline{\Delta E}$ for comparisons of curves of the same color. The top 10 was extracted from a sample with overall minimum mean equal to 2.96 and maximum mean to 45.08. *Acanthoscelio* (AC), *Chromoteleia* (CR), *Macroteleia* (MA), *Opisthacantha* (OP) and *Sceliomorpha* (SM).

	Genus 1	Genus 2	Color 1	Color 2	$\overline{\Delta E}$
1	AC	MA	OR	OR	19.64
2	MA	TR	OR	OR	18.26
3	CR	MA	OR	OR	17.02
4	AC	OP	OR	OR	16.95
5	AC	SM	OR	OR	15.77
6	AC	BA	OR	OR	14.35
7	OP	TR	BL	BL	14.16
8	SM	TR	OR	OR	13.69
9	OP	MA	OR	OR	13.61
10	CR	OP	BL	BL	13.32

$$Distance = \sum_{\lambda} |R_c(\lambda) - Rc'(\lambda)|$$

for each pair of comparisons (c, c'). The results in Fig 6 show how the same differences depicted when using ΔE are very clear for the green and red components, but not for the blue component.

Panel (D) of Fig 6 shows the color component contributions for the *Acanthoscelio* and *Triteleia* genera for the black and orange measurements, respectively. The spectra were normalised by their own areas to illustrate the similarity of the contributions that are reflected in all data taken and presented in Fig 4 and 6 in panels A, B and C. Apart from the small changes in intensity for the normalised spectra, the maxima of each curve have a different dominant wavelength between genera, but the shape of the curves

Fig 6. Examples of violin and box plot comparison per genus and color component. Distributions of *Distance* for (A) green, (B) red and (C) blue components, calculated for pairs of curves of three different genera: *Acanthoscelio* (AC), *Baryconus* (BA) and *Triteleia* (TR), and two scenarios: when they have the same color (in vermilion) and when their colors differ (bluish green). Plot (D) is an example of how the R, G, and B curves look for *Acanthoscelio* (AC) and *Triteleia* (TR) in orange and black spots.

Fig 7. Scatter plot for color components per genus. Logarithm of the area under the curve, per component (red, green or blue) versus the corresponding wavelength in nm where the maximum occurs. Solid color points represent means for each genus and faded points correspond to independent observations. Plot (A) and (B) represent the components for the black and orange colors respectively.

was very similar. The main changes are the differences in area when not normalised.

Mapping color variants in a spectral space can yield information about the relationship between genotypic space and evolution [29]. In this context, in order to visualize the data provided by the spectral component analysis as a function of the genus, Fig 7 is presented. It shows the scatter plots for the black and orange measurements for all data, representing the logarithm of the area of each color component versus the dominant wavelength at the maxima of each contribution. The mean is plotted as a solid circle of the same color for each set of faded data points.

The range of wavelength values at the maxima for each color component contribution is interesting, being around 5 nm for the blue contribution in both black and orange measurements. For the green (black measurement) and red (orange measurement) components it is about 5 nm, but for the red (black measurement) and green (orange measurement) it is approximately 20 nm.

The genus *Evaniella* appears to separate from the group in the black measurements, but it is not that obvious for the orange measurements. Meanwhile, *Sceliomorpha* and *Macroteleia* group together for the green and red orange observations. The genus *Chromoteleia* is outside the main group in all three color components for the black observations and in the blue component of the orange observations. *Chromoteleia* and *Baryconus* also pair in the green and red components of the black and orange observations. Additionally *Sceliomorpha* and *Acanthoscelio* present a mean that is very close in the black measurements for the blue, green and red contributions. The genera *Opisthacantha* and *Triteleia* group together in the blue and green components for the orange contribution.

Discussion

The following discussion is focused mainly on the contribution of the different statistical approaches to the physical and biological interpretation of the data obtained following the procedures proposed, with the aim of establishing a quantitative analysis method for the study of cuticle color of insects. This section has two main parts: discussion about differences and suitability of the statistical methods and the interpretation of the physical results within a biological context.

Regarding the suitability of the statistical methods applied, the present study highlights the differences in terms of results that could arise using univariate methods, which ignore physical information contained in the reflectance measurement as a function of wavelength, versus analyzing data using FDA techniques.

The difference in intrageneric variances illustrated in Fig 8 of the Supplementary material S2, confirms that the analysis of reflectance differences should not be done using univariate statistics. This variability is due to the difference in measurements across wavelengths: some sections are equal to zero, others are statistically different from zero, as described in the previous section. This can yield misleading results, finding univariate groups statistically similar, when the functional results are describing something different. In contrast, Functional Data Analysis (FDA) provides methods for analyzing data that are believed to arise from curves evaluated at a finite grid of points allowing the incorporation of spectral information from the reflectance curves [17].

In addition, a multivariate Euclidean distance defined in terms of a quantity with

physical meaning such as ΔE, provides more evidence for analysis, besides confirming the FDA results. The details about multiple comparisons can be even more specific, if the color components are separated for each curve and the analysis is repeated.

The RGB component analysis showed that the nature of the differences found using the FDA and ΔE methods are related to the green and red color components, but no correlation was found for the blue component. This is consistent with results obtained qualitatively using the difference curves $Y_{ijk}(\lambda)$ shown in Fig 3. Moreover, ANOVA results for model 7 (Table 3) confirm the reproducibility of the experimental protocol and, for most of the cases studied, the results suggest that there is a low variability in the sample spectral measurements even though the taxonomic identification to just the genus level means that at least some of the genera could consist of more than one species.

This particular method for statistically testing for color differences differs from those in the literature due to the level of scrutiny. It goes from the general question, "Is there a difference between colors?" to more specific contrasts between genera and color, using both the FDA and the color components methods. In this way, we are statistically comparing each pair of colors and genera, controlling for the other experimental factors.

In general, the results of the spectral decomposition, together with the color differences as analyzed in Fig 5, suggest that the cuticle segments from different genera, but with the same color (black vs. black, orange vs. orange) might have a similar chemical composition or an identical chemical composition with different concentrations. Tonal variations could be related to different amounts of an individual pigment or a combination of similar polymers distributed in the cuticle.

Recent studies report that the color phenotype is associated with the concentration and ratio of pigment forms [3, 30], but other factors such as nanostrucutres of the integument can affect the expression of color [31].In the case of pigments of different color (black vs. orange), the spectral blue components remain almost identical in terms of shape and characteristic wavelength (where the maximum occurs), suggesting that there is a common compound for the pigments. Notice that the normalization of the spectral components, equations (2), (3) and (4) allows the use of these features for comparison instead of intensity, which is related to the quantity of red, green or blue present in each color. The information given by the spectral components is important because optical properties are related to energetic processes due to electronic transitions when the pigment molecules interact with electromagnetic radiation. The shorter the wavelength, the more energetic the process is and therefore differences can be interpreted in terms of differences of electronic structure, which is related to the chemical composition of the pigments. A chemical characterization using infrared spectroscopy was attempted directly on the cuticle and results showed subtle differences suggesting a similar nature of the functional group attached to the molecule. Such results are not shown here because they are not conclusive due to the fact that the exact chemical composition of the cuticle for each color is unknown. A definitive identification of the pigment requires chemical extraction which at the moment could not be done because of the small amount of cuticle available.

Evaniella belongs to a completely different family (Evaniidae) and might therefore be expected to differ markedly from all the other genera. This indeed appears to be the case for its black color, but not its orange color (Fig 7). This is an interesting result in that it might suggest that the orange color shows more convergent evolution than does the black color, but it remains to be demonstrated that the pigments are actually convergent. Nonetheless, within Scelionidae there are more differences in the orange color than in the black (Table 5). Comparing the results shown in Fig 7 with a recent phylogenetic reconstruction of scelionid genera [12], a few other relationships between spectral properties and phylogeny appear to be present. For example, *Baryconus* and

Chromoteleia are paired in the green and red components for both black and orange, and these two genera are hypothesized to be closely related. *Opisthacantha* and *Triteleia* group together in the blue and green components of the orange coloration, and these two genera are also closely related. On the other hand, the orange color of *Macroteleia* and *Sceliomorpha* group together for the green and red components (Fig 7B) despite these genera not being closely related. These results are intriguing and merit further investigation to determine whether certain spectral properties parallel the phylogeny.

In some other insects, contrasting black and orange color patterns are known to serve as aposematic (warning) coloration for potential predators [32] and it is possible that the same is true of the BOB pattern, although this has not yet been tested. However, the orange mesosoma becomes less common at higher elevations [11], suggesting that other factors such as thermoregulation (black absorbs more sunlight) might play a role at these elevations; it is also possible that predators or aposematic models vary with elevation.

There is obviously much more to be learned about the black and orange colors present in numerous parasitic wasps, but it is hoped that the results of the present spectral analyses provide a framework for future research, specially when a direct chemical characterization of the pigments is not possible and the optical and statistical methods proposed in this work can be applied.

Conclusions

The present study provides the first characterization of the common black-orange-black pattern that is found in small (3-10 mm) parasitic wasps. Because of their small size and difficulty in obtaining sufficient numbers of fresh specimens for pigment extraction, microspectrophotometry was used to obtain reflectance spectra for eight genera of scelionids. These reflectance data were analyzed by means of univariate analyses and by Functional Data Analysis, the latter proving to be superior because it gave a better representation of the physical information, given that it takes into account complete curves and not just summaries.

The RGB spectral components method is proposed for studying the origin of the color in the cases when a direct chemical composition is not possible. In the case of the genera studied, the spectral blue components of the orange and black color were found to be almost identical, suggesting that there is a common compound for the pigments. This result holds even when different genera were considered for comparison. Also spectral measures can and should be used for quantifying differences in biological color patterns since such analysis provides information that is not tinted by the illuminant.

A correlation between the mean values of characteristics of the color components was used in an attempt to group genera that show similar values and some, but not all, of these groupings are similar to their positions in a previously published phylogeny of the genera.

Further study, including a chemical characterization of the pigments and classification of specimens into species should be performed in order to obtain validation of these results.

Supporting information

S1 Color coordinates calculation

The tristimulus values for color coordinates, according to the CIE (International Commission on Illumination, or Commission Internationale d'Eclairage) definition, were calculated as follows:

$$X = k \sum \phi_\lambda \bar{x}(\lambda) \Delta\lambda \tag{8}$$

$$Y = k \sum \phi_\lambda \bar{y}(\lambda) \Delta\lambda \tag{9}$$

$$Z = k \sum \phi_\lambda \bar{z}(\lambda) \Delta\lambda \tag{10}$$

where $\bar{x}(\lambda)$, $\bar{y}(\lambda)$ and $\bar{z}(\lambda)$ are the standardized color-matching functions (CMF) of the CIE 1931 standard observer. The constant k in equations (8), (9) and (10) is defined so that Y=100 for objects with perfect reflectivity. Finally, ϕ_λ is the relative color stimulus function, defined as:

$$\phi_\lambda = R(\lambda) S(\lambda) \tag{11}$$

with $R(\lambda)$being the spectral reflectance factor (the measure spectra) and $S(\lambda)$ is the relative spectral power distribution (SPD) of the illuminat.

These CIE-XYZ tristimulus values were calculated using the measured spectra (Fig 2). A D-65 day light standard illuminant for the CIE observer was chosen. Following the CIE recommendations, a spacing of 1 nm for wavelength was used. Since the data measured with the microspectrophotometer have a different wavelength spacing, an interpolation was made using the splinefun function in R [33], to generate a list of spectral points with the desired spacing.

After the tristimulus values were obtained, they were used to calculate the CIE 1976 (L* a* b*) (CIELAB) color space coordinates. The coordinates in the CIELAB color space are:

$$L = 116 f(Y/Y_n) - 16 \tag{12}$$

$$a = 500 [f(X/X_n) - f(Y/Y_n)] \tag{13}$$

$$b = 200 [f(X/X_n) - f(Z/Z_n)] \tag{14}$$

where

$$f(r) = r^{1/3} \text{ if } r > (24/116)^3 \tag{15}$$

and

$$f(r) = (841/108) r + 16/116 \text{ if } r \leq (24/116)^3 \tag{16}$$

with X_n, Y_n and Z_n being the tristimulus values of a idealized white reflector.

S2 Univariate comparison for the inter-genera case

For the univariate case, Table 2 shows how *Baryconus*, *Opisthacantha*, and *Scelio* absolute differences are not significantly different from zero. This contradicts the functional data analysis result presented before, where all the difference curves are different between genera.

When describing the difference between genera, Table 2 shows how there are three groups of genera without univariate mean differences: group a consists of *Baryconus*, *Opisthacantha*, *Scelio*, *Acanthoscelio* and *Chromoteleia*, while group b consist of *Triteleia*, *Sceliomorpha*, *Macroteleia* and *Evaniella* as members. Lastly, *Acanthoscelio* and *Chromoteleia* (group c) seem not to differ in terms of univariate means. On the other hand, when examining the curves in Fig 3, it is clear that they differ, with *Triteleia*'s difference curve being very close to zero from $\lambda = 420$ nm to $\lambda = 590$ nm and then going to a negative difference of 20 around $\lambda = 780$ nm, while the curve for *Macroteleia* deviates from zero around $\lambda = 500$ nm and reaches -20 at $\lambda = 680$ nm. Given that other characteristics from the curve are compared in this case, it is reasonable to have a contradictory result. The pattern of contradictory results between univariate and functional results is repeated in all pairs of genera belonging to the same

univariate group (a,b,c), which could indicate one of the limitations of the univariate analysis: it ignores the fact that the spectrometer measurements are curves and they should be compared as such.

Fig 8 shows the box plots for univariate mean differences for each genus. The black dashed line represents the null hypothesis of no difference between colors within a genus.

Fig 8. Box plots for univariate mean differences per genus. Black dashed line represents the null hypothesis of no difference between colors per genus. *Acanthoscelio* (AC), *Baryconus* (BA), *Chromoteleia* (CR), *Macroteleia* (MA), *Opisthacantha* (OP), *Scelio* (SC), *Sceliomorpha* (SM), *Triteleia* (TR) and *Evaniella* (EV).

S3 Top 10 Tables

Table 6. Top 10 according to $\overline{\Delta E}$ for comparisons of curves of different genera. The top 10 was extracted from a sample with overall minimum mean equal to 2.96 and maximum mean to 45.08 *Acanthoscelio* (AC), *Baryconus* (BA), *Chromoteleia* (CR), *Macroteleia* (MA), *Opisthacantha* (OP) and *Sceliomorpha* (SM).

	Genera 1	Genera 2	Color 1	Color 2	$\overline{\Delta E}$
1	CR	MA	BL	OR	45.08
2	MA	TR	OR	BL	44.79
3	MA	SM	OR	BL	43.59
4	LA	TR	OR	BL	41.51
5	CR	SM	BL	OR	41.25
6	SM	TR	OR	BL	41.22
7	MA	SC	OR	BL	41.02
8	CR	LA	BL	OR	40.84
9	BA	TR	OR	BL	39.69
10	AC	MA	BL	OR	39.58

Table 7. Top 10 according to $\overline{\Delta E}$ for comparisons of curves of the same genera. The top 10 was extracted from a sample with overall minimum mean equal to 2.96 and maximum mean to 45.08. *Acanthoscelio* (AC), *Baryconus* (BA), *Chromoteleia* (CR), *Macroteleia* (MA), *Opisthacantha* (OP), *Scelio* (SC), *Sceliomorpha* (SM) and *Triteleia* (TR).

	Genera 1	Genera 2	Color 1	Color 2	$\overline{\Delta E}$
1	SM	SM	BL	OR	39.71
2	MA	MA	BL	OR	34.59
3	SC	SC	BL	OR	34.36
4	BA	BA	BL	OR	34.11
5	CR	CR	BL	OR	32.02
6	LA	LA	BL	OR	30.26
7	TR	TR	BL	OR	29.57
8	AC	AC	BL	OR	20.37
9	LA	LA	OR	OR	10.40
10	LA	LA	BL	BL	10.28

S4 Eosin hematoxylin technique

The specimens were fixed in 10% formalin, where they were preserved until they were processed. Subsequently, a post fixation in formaldehyde, acetic acid, ethanol

solution was carried out for two hours. Dehydration was carried out in alcohols with increasing concentration (70%, 95%,100%). Clarification was implemented with xylol and later the sample was impregnated with paraffin. Once the specimens were processed, they were included in paraffin blocks. Continuous cuts were made at 4 microns in thickness and 3 or 4 cuts were placed per slide, using the entire mesoscutum. Hematoxylin and eosin stains, in a VENTANA HE 600 system, were performed on all sections. The final cuts were observed in an Olympus bx51 microscope and the photographs obtained with an Olympus Dp72 camera.

Fig 9 shows the variation in the distribution of the pigment together with the roughness of the cuticle surface. These factor contribute to the reflection of light in different directions and therefore to the dispersion of the data .

Fig 9. Eosin hematoxylin technique Cross section of the cuticle shows the pigment concentrated in the epicuticle, of a specimen of *Baryconus* with black-orange-black color (a) and black color (b). The variation in the distribution of the pigment together with the roughness of the cuticle surface can contribute to the reflection of light in different directions and therefore to the dispersion of the data

Acknowledgments

This work was inspired by personal conversations with Lubomir Masner. We would like to give special thanks to Mauricio Arce for the macro photography and to Juan Porras Peñaranda for his support in eosin-hematoxilin analysis. We thank three anonymous reviewers whose comments and suggestions helped improve and clarify this manuscript.

References

1. Rutowski RL, Macedonia JM, Morehouse N, Taylor-Taft L. Pterin pigments amplify iridescent ultraviolet signal in males of the orange sulphur butterfly, Colias eurytheme. Proceedings of the Royal Society B 2005, 272: 2329-2335. doi:10.1098/rspb.2005.3216

2. Garcia JA, Polidori C, Nieves-Aldrey JL. Pheomelanin in the secondary sexual characters of male parasitoid wasps (Hymenoptera: Pteromalidae). Arthropod Structure & Development. 2016;45(4):311–319. doi:10.1016/j.asd.2016.05.001.

3. Hines HM, Witkowski P, Wilson JS, Wakamatsu K. Melanic variation underlies aposematic color variation in two hymenopteran mimicry systems. PLOS ONE. 2017;12(7):e0182135. doi:10.1371/journal.pone.0182135.

4. Mallet J, Joron M. Evolution of diversity in warning color and mimicry: polymorphisms, shifting balance, and speciation. Annual Review of Ecology and Systematics. 1999;30(1):201–233. doi:10.1146/annurev.ecolsys.30.1.201.

5. Brakefield PM. Industrial melanism: Do we have the answers? Trends in Ecology & Evolution. 1987;2(5):117–122. doi:10.1016/0169-5347(87)90051-6.

6. Marchini M, Sommaggio D, Minelli A. Playing with black and yellow: the evolvability of a batesian mimicry. Evolutionary Biology. 2017;44(1):100–112. doi:10.1007/s11692-016-9397-0.

7. Vidal-Cordero JM, Moreno-Rueda G, López-Orta A, Marfil-Daza C, Ros-Santaella JL, Ortiz-Sánchez, FJ. Brighter-colored paper wasps (*Polistes dominula*) have larger poison glands. Frontiers in zoology. 2012;9(1):20.

8. Rapti Z, Duennes MA, Cameron SA. Defining the colour pattern phenotype in bumble bees(*Bombus*): a new model for evo devo. Biological Journal of the Linnean Society. 2014;113(2):384–404. doi:10.1111/bij.12356.

9. Plotkin M, Hod I, Zaban A, Boden SA, Bagnall DM, Galushko D. et al. Solar energy harvesting in the epicuticle of the oriental hornet (*Vespa orientalis*). Naturwissenschaften. 2010;97(12):1067–1076. doi:10.1007/s00114-010-0728-1.

10. Austin AD, Johnson, NF, Dowton, M. Systematics, evolution, and biology of scelionid and platygastrid wasps. Annu. Rev. Entomol.2005;50:553–582. doi:10.1146/annurev.ento.50.071803.130500

11. Mora R, Hanson PE. Widespread occurrence of black-orange-black color pattern in Hymenoptera. Journal of Insect Science. 2019;19(2). doi:10.1093/jisesa/iez021.

12. Murphy NP, Carey D, Castro LR, Dowton M, Austin A.D. Phylogeny of the platygastroid wasps (Hymenoptera) based on sequences from the 18S rRNA, 28S rRNA and cytochrome oxidase I genes: implications for the evolution of the ovipositor system and host relationships. Biological Journal of the Linnean Society.2007;91(4):653–669. doi:10.1111/j.1095-8312.2007.00825.x.

13. Aguiar A. An accurate procedure to describe colors in taxonomic works, with an example from Ichneumonidae (Hymenoptera). Zootaxa. 2005;1008(1):30–38. doi:10.11646/zootaxa.1008.1.4.

14. Stavenga DG, Leertouwer HL, Wilts BD. Quantifying the refractive index dispersion of a pigmented biological tissue using Jamin–Lebedeff interference microscopy. Light: Science & Applications. 2013;2(9):e100–e100. doi:10.1038/lsa.2013.56.

15. Skaldina O, Sorvari J. Not simply red: colouration of red wood ant *Formica rufa*(Hymenoptera: Formicidae) is polymorphic, modular and size-dependent. European Journal of Entomology. 2017;114:317–324. doi:10.14411/eje.2017.039.

16. Endler JA, Mielke PW. Comparing entire colour patterns as birds see them. Biological Journal of the Linnean Society. 2005;86(4):405–431. doi:10.1111/j.1095-8312.2005.00540.x

17. Ramsay J, Silverman, BW. Functional data analysis. 2nd ed. Springer Series in Statistics. New York: Springer-Verlag; 2005. Available from: `www.springer.com/gp/book/9780387400808`. doi:10.1007/B98888

18. Rivas T, Matías JM, Taboada J, Ordóñez C. Functional experiment design for the analysis of colour changes in granite using new L*a*b* functional colour coordinates. Journal of Computational and Applied Mathematics. 2011;235(16):4701–4716. doi:10.1016/j.cam.2010.08.005.

19. Masner L. Revisionary notes and keys to world genera of Scelionidae (Hymenoptera: Proctotrupoidea). The Memoirs of the Entomological Society of Canada.1976;108(S97):1-97 doi:10.4039/entm10897fv

20. Peters RS, Krogmann L, Mayer C, Donath A, Gunkel S, Meusemann K, et al. Evolutionary History of the Hymenoptera. Current Biology. 2017;27(7):1013–1018. doi:10.1016/j.cub.2017.01.027.

21. Tkalcic M, Tasic JF. Colour spaces: perceptual, historical and applicational background. vol. 1. IEEE;2003 doi:10.1109/EURCON.2003.1248032

22. Schanda J.(Ed). Colorimetry: understanding the CIE system. New Jersey: John Wiley & Sons; 2007. doi:

23. Melgosa M, Trémeau A, Cui G. Colour difference evaluation. Advanced color image processing and analysis New York: Springer New York; 2013. p. 59–79. doi:10.1007/978-1-4419-6190-7_3.

24. Mahy M, Van Eycken L, Oosterlinck A. Evaluation of uniform color spaces developed after the adoption of CIELAB and CIELUV. Color Research & Application. 1994;19(2):105–121. doi:10.1111/j.1520-6378.1994.tb00070.x

25. Renoult JP, Kelber A, Schaefer HM. Colour spaces in ecology and evolutionary biology Biological Reviews. 2017;92(1):291–315. doi:10.1111/brv.12230

26. Causeur D, Sheu CF, Chu MC, Rufini F. ERP: Significance analysis of event-related potentials data; 2018. Available from: `https://CRAN.R-project.org/package=ERP`.

27. Gorecki T, Smaga L. fdANOVA: Analysis of variance for univariate and multivariate functional data; 2018. Available from: `https://CRAN.R-project.org/package=fdANOVA`.

28. Wickham H. tidyverse: Easily Install and Load the 'Tidyverse' R package version 1.2.1. 2017. url:https://CRAN.R-project.org/package=tidyverse

29. Whibley AC, Langlade NB, Andalo C, Hanna AI, Bangham A, Thébaud C, Coen, E. Evolutionary paths underlying flower color variation in *Antirrhinum*. Science. 2006;313(5789):963–966. doi:10.1111/brv.12230

30. Galván I, Wakamatsu K. Color measurement of the animal integument predicts the content of specific melanin forms. RSC Advances.2016;6(82):79135–79142. doi:10.1039/C6RA17463A

31. Shawkey MD, D'Alba L. Interactions between colour-producing mechanisms and their effects on the integumentary colour palette. Philosophical Transactions of the Royal Society B: Biological Sciences. 2017;372(1724):20160536. doi:10.1098/rstb.2016.0536

32. Arenas LM, Walter D, Stevens M. Signal honesty and predation risk among a closely related group of aposematic species. Scientific Reports. 2015;5:11021. doi:10.1038/srep11021.

33. R Core Team RFfSC. R: A Language and Environment for Statistical Computing; 2018. Available from: `http://www.R-project.org/`.

Academic Editor
PLOS ONE

Dear Phillip Barden,

We would like to thank the reviewers for their critical assessment of our work. In the following rebuttal letter, we address each point raised by the academic editor and reviewer(s).

Comments from Editor and Reviewers	Our response (including specific changes made to the document)
**Chosen color to highlight PLOS ONE comments in the manuscript**<br><br>1. In your Methods section, please provide additional location information, including geographic coordinates for the data set if available.	Done ✔
2. To comply with PLOS ONE submissions requirements for field studies, please provide the following information in the Methods section of the manuscript and in the "Ethics Statement" field of the submission form (via "Edit Submission"):<br>a) Provide the name of the authority who issued the permission for each location (for example, the authority responsible for a national park or other protected area of land or sea, the relevant regulatory body concerned with protection of wildlife, etc.). If the study was carried out on private land, please confirm that the owner of the land gave permission to conduct a study on this site.<br>b) For any locations/activities for which specific permission was not required, please	Information provided. Done ✔

- i. state clearly that no specific permissions were required for these locations/activities, and provide details on why this is the case<br>- ii. confirm that the field studies did not involve endangered or protected species<br>c) For vertebrate studies only, please provide the following additional information:<br>- i. Full details of collection and sampling methods, including method of sacrifice if applicable<br>- ii. State whether the vertebrate work was approved by an Institutional Animal Care and Use Committee (IACUC) or equivalent animal ethics committee. If no approval was obtained, please explain why it was not required.<br>- iii. State clearly whether all sampling procedures and/or experimental manipulations were reviewed or specifically approved as part of obtaining the field permit.<br>For more information about PLOS ONE submissions requirements for field studies, please refer to http://journals.plos.org/plosone/s/submission-guidelines#loc-animal-research.	
3. We note you have included a table to which you do not refer in the text of your manuscript. Please ensure that you refer to Tables 6 & 7 in your text; if accepted, production will need this reference to link the reader to the Table.	The reference was pointing to the supplementary section, but it has been corrected to reference the tables in the following way: "Similarly, top ten comparisons of different genera and the same genus are presented in Table~\ref{tab:T8app} and Table~\ref{tab:T9app}, included in \nameref{S3_Table} in the supplementary material.".<br>Done ✔

<table>
<tr><td>From Reviewer's Responses to Questions
1. Is the manuscript technically sound, and do the data support the conclusions?
Reviewer #1: Partly</td><td>It is not clear to us why reviewer 1 said partly in this case.
Done ✔</td></tr>
<tr><td>From Reviewer's Responses to Questions
3. Have the authors made all data underlying the findings in their manuscript fully available?
Reviewer #2: No</td><td>Data and code is fully available in a Github repo, plus, summary stats were presented in the manuscript. The complete repository will be added as Supplementary Material in a .zip file.

Done ✔</td></tr>
<tr><td colspan="2">The deletions of text suggested by the reviewers were done, having been deleted, they are not observed in color.</td></tr>
<tr><td>Reviewer 1 (chosen color to highlight the suggestions of this reviewer in the manuscript)</td><td></td></tr>
<tr><td>Reviewer 1: The point and motivation of the article

a. Is this a case study, meant to show that FDA is better than univariate analysis for quantifying differences in biological color pattern?
b. Is this a case study, meant to show that more rigorous measurements can and should be used when quantifying differences in biological color pattern?
c.Is the point of this study that colors that appear very similar from the point of view of a human observer can in fact be significantly different from each other?
d. Why did the authors choose this family of wasps?</td><td>A-B This is a case study, meant to show that more rigorous measurements can and should be used when quantifying differences in biological color patterns.

C. No. This phrase was eliminated.

D-E. The Introduction was modified in order to incorporate reviewers suggestions.

F. The BOB pattern includes both colors.

G. See Discussion

Done ✔</td></tr>
</table>

e. Why didn't they make these measurements with other parasitic wasps? Or with flies? Or with flowers?<br>f. What is the additional benefit of analyzing the orange colors, instead of only analyzing the black colors?<br>g. How do the authors hope that this article will influence their colleagues' questions/methods in future studies?	
Reviewer 1: rewrite the abstract following this guide: http://www.easterbrook.ca/steve/2010/01/how-to-write-a-scientific-abstract-in-six-easy-steps/	Done ✔
Reviewer 1: clarity in Abstract. 'describe and test the color differences between genera' What are these 'color differences'? If a color is found to be 'different' between two genera, what does that mean?	Done ✔
Reviewer 1: add a conclusion section with individual sentences - containing each of their main conclusions (significant differences in color, the superiority of FDA over univariate analyses, etc.), arrange those sentences from most to least important starting with the main point of the paper, and then turn their list of sentences into a cohesive paragraph by adding transition phrases.	Done ✔
Reviewer 1: clarity. The Introduction, Methods, and Discussion center on how wonderful FDA is, but neither the phrase 'Functional Data Analysis' nor the abbreviation 'FDA' appear in the Results. If you want to convince people to use a certain method, you	Results using FDA methods were presented in the Results, as well as were commented in the Discussion. The abbreviation FDA was added in those sections in order to make it more clear.<br>Done ✔

must make the results of that method clear.	
Reviewer 1: clarity / Line 157. Which 'means' are the authors talking about? (This question is answered a few lines down, in the bullet points, but the reader shouldn't have to wonder as they finish this paragraph.)	Added "color reflectance". Done ✔
Reviewer 1: Line 223. 'independence between spots' Independence in what sense—independence between the black and orange spots on the same specimen? Between different specimens belonging to the same genus?	Added "on the same specimen". Done ✔
Reviewer 1: Line 224-6. 'there is statistical evidence of a difference between mean reflectance of color for at least one genus and zero, controlling by specimen and spot.' This is not entirely clear. Do the authors mean that, in at least one genus examined, the mean reflectance of the orange spot is significantly different from the mean reflectance of the black spot?	Deleted "there is statistical evidence of a difference between mean reflectance of color for at least one genus and zero" and added "in at least one genus examined, the mean reflectance of the orange spot is significantly different from the mean reflectance of the black spot". Done ✔
Reviewer 1: Line 247. 'the differences due to spots' What does this mean?	Deleted "the differences due to spots" and added "the mean reflectance difference between measurements done in the same specimen but in different spots".<br>Done ✔
Reviewer 1: Line 259-61. 'In contrast, the distance between Acanthoscelio (AC) and Acanthoscelio (AC) curves for the same color (in vermilion) clearly include the threshold and can be taken as not significantly different.' The curves being compared are	Correct, individual curves from different specimens. Added the phrase "and different specimens" for clarity.<br>Done ✔

individual curves from different specimens, right?	
Reviewer 1: Even if it is not possible to implement existing phylogenetic comparative methods in this manuscript, the authors should discuss whether the genera included in the Murray phylogeny that are most closely related to each other are most similar in terms of their color. So far the authors do this for only one pair of genera (‘the orange color of Macroteleia and Sceliomorpha group together for the green and red components (Fig. 7B) despite these genera not being closely related’, lines 360-2). This needs to happen in a comprehensive, deliberate manner.	We have done this in a rewritten paragraph. Done ✔
Reviewer 1: When a genus or species is mentioned for the first time, its name must be followed by its taxonomic authority: the author(s) who described the genus/species. For example, Acanthoscelio is Acanthoscelio Ashmead. Also, the two-letter abbreviation for the generic names just add a layer of unnecessary information to the manuscript. These abbreviations should be removed, and replaced with the full names of the genera.	Done ✔
Reviewer 1: accessibility. Line 91. Explain what the CIE color space is and why it was used here. •	The following lines were added:<br>“Regarding geometrical representation of color stimuli, the CIE color spaces are recognized to have important characteristics such as being device-independent and having a perceptual linearity \cite{tkalcic2003colour}. Nevertheless, not all color spaces are suitable for comparing colors because distances between

	coordinates are not easily quantifiable due to the lack of uniformity of the space. For these effects, the CIE recommends using CIELAB color space due to its uniformity\cite{schanda_colorimetry:_2007}."<br>Done ✔
Reviewer 1: accessibility.Reviewer 1: accessibility. Line 102-3. Explain the biological meaning of delta-E and delta-P. • Line 138. Explain the biological meaning of the 'response variable'. • Line 239-40. Restate the biological meaning of 'the overall average change in all comparisons'. • Line 243. Restate the biological meaning of delta-E when it is first used in the Results.	interpretation<br>delta-E: change or distance between reflectance curves.<br>delta-P: change or distance between L, a or b components. In order to avoid confusion, it was written explicitly delta-L, delta-a, delta-b and the term delta-P was eliminated. The new line reads as follows:<br>"Here, the quantity Delta L, Delta a, Delta b, represent the subtraction of the corresponding coordinates of the two colors being compared and Delta E represents the distance between the two colors within the CIELAB space.<br><br>Response variable (biological meaning): difference of reflectance or a measure of reflectance.<br>Overall average change in all comparisons (biological meaning): average difference between all measurements.<br>Done ✔
Reviewer 1: Line 2-11. The first two paragraphs of the Introduction say the same thing; one of them should be deleted. Also, the biological statements here ('These markings contribute to important behavioral, neurological, physiological, visual, morphological and developmental traits') are too vague/general.	First paragraph deleted. The vague statement has been rewritten to be more specific.<br>Done ✔
Reviewer 1: Line 22-5. 'The wide distribution of the BOB pattern in Hymenoptera suggests that the	These are important observations regarding the original manuscript and we have

coloration arose due to changes in shared metabolic or genetic routes as reported in other taxa, and strongly suggests that it has some biological function [10].' Not necessarily, at least according to the information the authors have provided here. This color pattern could be ancestral (and not serve any biological function) in the hymenopteran clade in question, with widespread, convergent losses.	therefore totally rewritten this section (third paragraph in the Introduction).<br>Done ✔
Reviewer 1: Line 26. 'It has been reported that most animals perceive color differently than humans' Differently in what way?	This phrase has been deleted, as it is not necessary.<br>Done ✔
Reviewer 1: Line 27. 'which has created a new surge of interest' Citations needed to demonstrate that there is a 'surge'.	This phrase has been deleted, as it is not necessary.<br>Done ✔
Reviewer 1: Line 39-40. What data were fed into the PCA?	Added "on the radiance spectra".<br>Done ✔
Reviewer 1: Line 40. 'while recognizing its limitations' What are these 'limitations'?	Added "specifically, those related to the violation of assumptions for statistical tests to discriminate between colors".<br>Done ✔
Reviewer 1: Line 41-42. 'Functional data analysis (FDA) is a powerful method to study spectrally measured data' Powerful how? How does it work, how is it different/better than PCA?	Added "since it assumes that the underlying process generating the data is smooth, and hence provides tools to analyse a spectral measurement as a function and not as a series of data points".<br>Done ✔
Reviewer 1: • Line 67-8. ''They were found and caught with entomological nets over a period of 12 months.' This raises the question of whether there are seasonal changes in color pattern.	There are only two seasons in Costa Rica, dry and wet season, and there is no evidence of change in color pattern during the year. Indeed there appears to be very little intra-specific variation in color (Mora & Hanson 2019).<br>Done ✔

Reviewer 1: Line 68. Evaniidae is a family, not a genus.	We added the genus of Evaniidae<br>Done ✔
Reviewer 1: Line 78-9. 'A previous study demonstrated that males and females do not differ in color' This finding holds across a large clade that includes Scelionidae and also Evaniidae?	Yes. Changed to read: "A previous study demonstrated that the vast majority of hymenopterans with the BOB pattern, and those that are all black, do not show sexual dimorphism in color, at least in specimens that have been identified to species level in museum collections [15]."<br>Done ✔
Reviewer 1: Line 85-86. 'For each specimen three samples were taken from the mesosoma (orange) and from the metasoma (black).' Three samples were taken from the mesosoma and three were taken from the metasoma? This sentence is unclear.	We have clarified this sentence.<br>Done ✔
Reviewer 1: Line 88-89. 'Fig 2 shows the spectral reflectance of the eight genera for the black and orange segments together with the control genus.' This sentence belongs in the Results, not Methods. Also in what sense is the evaniid a 'control'? This seems like the wrong word to use.	Instead of "control" we now use "distantly related", as explained in a previous paragraph.<br>Done✔
Reviewer 1: Line 109. Spell out the whole term instead of using the abbreviation 'MSP'—this abbreviation is not used frequently enough to justify making the reader memorize it.	Done✔
Reviewer 1: Line 164. Define 'FANOVA'. I suspect the authors should cite its origination.	Added reference.<br>Done ✔
Reviewer 1: Line 165-7. 'A statistical test was performed to interpret the contrast results for the mean	Rephrased.<br>Done ✔

difference curves.' Which statistical test?	
Reviewer 1: Line 171-2. 'Here also, specimen and spot were controlled.' This is unclear.	Deleted "controlled", and added "included as factors in the linear equation".<br>Done ✔
Reviewer 1: Line 173-4. ``the usual univariate practice' The authors imply that the univariate method is commonly used, but I don't recall seeing any citations that show this to be the case.	Deleted "the usual univariate practice" and added "a univariate analysis approach".<br>Done ✔
Reviewer 1: Line 174-5. 'to highlight the importance of taking into account all the information from the difference curves.' It's disconcerting to see the authors state, in the Methods, that they designed their study based on the assumption that one analysis will perform significantly better than another.	Deleted "highlight the importance" and added "compare the results when".<br>Done ✔
Reviewer 1: Line 176. It's odd to see the same equation used for the univariate and functional responses, since each response was analyzed with a different kind of ANOVA and the functional response was analyzed with a metric computed with a special R package.	In order to simplify the section, we didn't include separate equations. The detail is that for FDA each observation is a curve, so the only thing that changes is the response: from Y to Y(lambda), the rest of the factors are the same, since they continue being measured in the same way. This has very important implications mathematically, hence the different packages, but it can be noisy to include a separate equation.<br>Done ✔
Reviewer 1: Line 178. What is the 'p =' doing in there? Why not just write 'i = 1, 2, ... , 8'?	Deleted p.<br>Done ✔
Reviewer 1: Line 178. Change 'j = 1, 2, .., r = 4 ' to 'j = 1, 2, 3, 4'.	Deleted r.<br>Done ✔

Reviewer 1: Line 197. I recommend that the authors cite the R package used to make their figures	Excellent suggestion. We added the citation. Added "Main data format and figures were done using the collection of R packages Tidyverse \cite{tidy}".<br>Done ✔
Reviewer 1: Line 205. The caption for Figure 2 is not provided anywhere.	Perhaps you missed it, its at the top of page 4.<br>Done ✔
Reviewer 1: Line 211-2. 'Moreover, the effect of topography of the cuticle and homogeneity of the pigment distribution can partly explain the data dispersion in some of the genera.' The authors need to substantiate this statement with a figure, data, something.	"Because the spot size of 200µm x 200µm used the reflectance measurements is more than 20 times larger than the features that comprise the mesosoma, the effect of topography of the cuticle and homogeneity of the pigment distribution can partly explain the data dispersion in some of the genera. See supplementary material S4"<br>Done ✔
Reviewer 1: Line 213. 'The difference reflectance of the curves' I think this is grammatically incorrect.	We are referring to the curve calculated as the difference, wavelength by wavelength, between two reflectance curves.<br>Changed the beginning of the sentence to "The difference curves for reflectance spectra".<br>Done ✔
Reviewer 1: Line 216-7. 'in the range between 550 nm and 750 nm, and the region between 750 nm and 900 nm' Why not change this to 'in the range between 550 nm and 900 nm'? Also the measurements go above 900 nm.	We meant to draw attention to the fact that the behavior of the curves can be described according to the wavelength. We substituted the whole sentence by:<br><br>" For each genus, the behavior of the difference curves between black and orange can be described according to the wavelength in the following way. Before 550nm, differences are around zero, meaning that the spectra are similar in this region. Between 550nm and 750nm the differences are below zero and are similar between genera. And finally, for wavelengths greater than 750nm differences are both above and below zero and are

	dispersed with respect to genus.<br>Also, eliminated the sentence: “Thus the differences between colors in each genus can be explained depending on the wavelength range” because that idea was already mentioned with the new sentence.<br>Done ✔
Reviewer 1: Figure 3. There is so much overlap between the different ribbons that it is often impossible to distinguish them. I suggest that the authors use a thick line for the mean value and thinner lines to mark the upper and lower extremes of each ribbon, leaving the interior blank (without any fill) so that it’s easier to distinguish the ribbons	Corrected the plot (See Fig3.tiff)<br>Done ✔
Reviewer 1: Line 220-1 (Figure 3 caption). What does it mean if the curve is below the black line versus above it?	Changed to caption to: “Difference (black-orange) of mean reflectance functions in each genus}}. Solid lines represent functional means, colored areas represent the area where difference functions in each genus are observed. A black dashed line represents the null hypothesis of no difference between colors in each genus. Curves above the black line mean that the black curves have higher reflectance values than the orange ones.”<br>Done ✔
Reviewer 1: Line 222-3. ‘The best fit in all cases was from the models that assume independence between spots, so linear models were used subsequently.’ I’m not sure exactly what mean by this (as outlined in the above section), but the genera examined here are not statistically independent from each other because some are more closely related to each other than others.	When fitting statistical models one can assume dependence and then compare those models taking into account dependence with others assuming independence. This phrase intends to clarify that the models assuming independence had a better fit than those that did not assume it. Therefore, we continued using the later ones.<br>Done ✔

Reviewer 1: Line 233-4. 'there is evidence of at least one genera difference being different from the rest' This is unclear phrasing. I think the authors meant to say, 'the difference between the black and orange spots in at least one genus is different from the difference between the black and orange spots in the other genera'?	Deleted "there is evidence of at least one genera difference being different from the rest" and added "the difference between the black and orange spots in at least one genus is different from the difference between the black and orange spots in the other genera".<br>Done ✔
Reviewer 1: Line 236-7. 'From such an analysis it can be concluded that the univariate approach is not suitable for treating this type of data.' Why can it be concluded that the univariate approach is not suitable'? Also, this is Discussion, not Results.	Deleted "From such an analysis it can be concluded that the univariate approach is not suitable for treating this type of data."<br>Done ✔
Reviewer 1: Line 248. 'statistically significant but negligible' The authors need to justify their contention that 'statistically significant' differences can be considered 'negligible'. Also, if statistically significant differences can be negligible, then what is the point of this analysis?	The significance tests should have a context in terms of the application. In this case, we found a bibliographical reference for a threshold. The point of this analysis is to compare differences statistically, taking into account application-based thresholds. See: https://amstat.tandfonline.com/doi/full/10.1080/00031305.2016.1154108#.XUmT3JMzbOR<br>Done ✔
Reviewer 1: Line 270-1. 'pointing to the specific contrast with wider differences' I do not understand what this is supposed to mean.	In statistics, a contrast is a comparison test among two groups or a linear combination of groups. Here, we refer to specific comparisons between pairs of genera and/or specific colors. We replaced the word contrast with comparisons.<br>Done ✔
Reviewer 1: Line 275-7. 'The hypothesis in this case is that the differences that were found in terms of can be explained in terms of color components.' If the differences cannot be explained in terms of color	Here we meant to address the possibility of using spectral color components as an analysis method for accessing information about the optical properties related to pigments without using color coordinates, that is, avoiding the question of whether the color is orange, red, brown or black to

components, what could explain the differences?	human vision. When calculating Delta E, all the information regarding the different wavelengths is integrated into a number. When using spectral components analysis, it becomes clear whether this difference comes from shorter or longer wavelengths. This information is important because optical properties are related to energetic process due to electronic transitions in the pigment molecules when interacting with electromagnetic radiation. The shorter the wavelength, the more energetic the process is and therefore it becomes more relevant in the identification of the pigment. In order to clarify the importance of this information, we replaced the first paragraph of the section named "RGB spectral components" of the **Materials and Methods Section** with the following paragraph:<br><br>"When calculating Delta E, all the spectral information is condensed into a single number and it is not clear whether the difference comes from shorter or longer wavelengths. In this sense, the use of a spectral space instead of a color space would provide a more objective description of the physical variation among stimuli and also allows the identification of different physical mechanisms producing the same perception of color but having different spectral behavior [Renault2017]Therefore, in order to visualize the characteristics of the reflectance curves in different regions of the electromagnetic spectrum, in this work, we propose a trichromatic spectral space defined using the IE color matching functions (CMFs) as follows:"<br><br>Also, the line mentioned by Referee 1 was deleted because the paragraph was changed.<br>Done ✔
Reviewer 1: Figure 6. Why on earth aren't the same genera used for panels A, B, and C (AC, BA, and TR)	Very good point. Redo the plot. We included only AC and TR on panel D for clarity, since

and also D (AC and SM)? At best, this is sloppy.	including a third set of curves could make it difficult to contrast the curves. See Fig6.tiff.<br>Done ✔
Reviewer 1: Tables 3 and 4. The captions for both tables say, 'Overall minimum mean is 2.96 and maximum mean is 45.08'. However, this is not true—the overall maximum mean in Table 4 is 19.64, not 45.08.	In this case, we are talking about the whole sample, and each table represents the top ten, thus, the caption is meant to clarify the descriptive statistics of the whole sample and not only from the top ten. Added to the captions: "The top 10 was extracted from a sample with overall minimum mean equal to 2.96 and maximum mean to 45.08"<br>Done ✔
Reviewer 1: Line 289-91. 'Our group has found evidence of a correlation between the color and/or reflectance spectra of beetles of the genus Chrysina (Scarabaeidae) with respect to its phylogenetic tree.' Is there a publication that the authors can cite? If not, this statement must be removed. You can't sneak definitive statements such as these into the refereed literature without subjecting the data behind those statements to peer review. And if this statement can be backed with a citation and does remain in the manuscript, it still doesn't belong in the Results.	This is a recent finding of our group. We are still working on this, therefore it hasn't been published yet. Following the recommendation, the statement was deleted from the manuscript.<br><br>We added instead :<br><br>"Mapping color variants in a spectral space can yield to information about the relationship between genotypic space and evolution [Whibley et al.2016]. In this context, in order to visualize the data provided by the spectral component analysis as a function of the genus, Fig 7 is presented."<br>Done ✔
Reviewer 1: Figure 7. The y-axis should be standardized by row and the x-axis should be standardized by column so that it is easier for the viewer to compare different panels that occur within the same column.	Redo the plot<br>See Fig7.tiff<br>Done ✔
Reviewer 1: Line 294-5. 'The mean is plotted as a solid circle of the same color for each set of faded data points.' It would be far more effective to plot the actual data as	Convex hulls add more noise to the plot, so we left it with the semitransparent points.<br>Done ✔

solid/opaque points, and replace the large dots with semi-transparent convex hulls that encompass the data for each genus.	
Reviewer 1: Line 301-10. These pairwise comparisons need to be discussed in terms of phylogeny/relatedness.	We have expanded this part to read: "Comparing the results shown in Fig. 7 with a recent phylogenetic reconstruction of scelionid genera [35], a few other relationships between spectral properties and phylogeny appear to be present. For example, *Baryconus* and *Chromoteleia* are paired in the green and red components for both black and orange, and these two genera are hypothesized to be closely related. *Opisthacantha* and *Triteleia* group together in the blue and green components of the orange coloration, and these two genera are also closely related. On the other hand, the orange color of *Macroteleia* and *Sceliomorpha* group together for the green and red components (Fig. 7B) despite these genera not being closely related. These results are intriguing and merit further investigation to determine whether certain spectral properties parallel the phylogeny."<br><br>Done ✔
Reviewer 1: Line 312. Table 5 contains Results, so it needs to be cited before the Discussion.	Done ✔
Reviewer 1: Line 334-5. 'there is a homogeneity of the sampling' What does this mean?	Deleted "a homogeneity of the sampling" and added "low variability in the sample spectral measurements".<br>Done ✔
Reviewer 1: Line 339. Specify what these 'more specific contrasts' are.	Added "between genera and color".<br>Done ✔
Reviewer 1: Line 342-7. The authors need to cite publications that support their use of spectral comparisons for speculation about whether these taxa	We agree, but it seems to us that this acclaration belongs more to the **"RGB spectral components**" section of the **Materials and Methods Section instead of Discussion. In this section,** we added the line:

have the same pigments. At what threshold of spectral dissimilarity would the authors no longer speculate that these taxa have the same pigments?	"It is recognized that spectral spaces have the advantage of providing information about the different processes generating phenotypic color diversity[Renuolt2017], and therefore we propose this RGB spectral component method as an alternative for the case when a direct chemical characterization can not be performed"<br><br>The question about the threshold is very important. We hoped to address this issue by the use of comparisons between color component curves in terms of the distance between them, and using experimental design techniques to measure the uncertainty associated with the difference between curves, and hence reporting the statistical differences along with the practical differences (see comment for Reviewer 1: Line 248).<br><br>Results are summarized in Fig 6 for three different genera, and also the Top 10 most different pairs of curves are summarized in Tables 4,5,6,7.<br>Done ✔
Reviewer 1: Line 348-9. 'Recent studies report that the color phenotype is clearly associated with the concentration of certain pigment forms' This statement requires multiple citations (one per study, at least two studies).	Done ✔
Reviewer 1: Line 349-50. 'there could be other relevant factors besides concentration related to the expression of color' Name those factors.	Changed to "...but other factors such as nanostructures of the integument can affect the expression of color [Shawkey & D'Alba 2017]<br><br>Shawkey MD & D'Alba L. 2017 Interactions between colour-producing mechanisms and their effects on the integumentary colour palette. Philosophical Transactions of the

	Royal Society B 372: 20160536.<br>Done ✔
Reviewer 1: Line 350-2. 'In the case of pigments of different color (black vs. orange), the spectral blue components remain almost identical, suggesting that there is a common compound for the pigments.' Why do the authors believe this? Are there any known black and orange pigments that share a common compound that is characterized by its spectral blue component?	Since wavelengths involved in the blue component are shorter than the ones involved in the red and even in the green component, electronic transitions associated with the characteristics of the spectrum in this region are considered of importance. The fact that even for different colors (black vs. orange) the components are identical is an important piece of information regarding the nature of the pigments involved in the black and orange coloration observed. See also comment about line 275-7.<br><br>The following line was added:<br><br>"This information is important because optical properties are related to energetic process due to electronic transitions when the pigment molecules interact with electromagnetic radiation. The shorter the wavelength, the more energetic the process is and therefore differences can be interpreted in terms of differences of electronic structure, which is related to the chemical composition of the pigments."<br>Done ✔
Reviewer 1: Line 352-5. 'Preliminary chemical characterization by means of infrared spectroscopy showed subtle differences between spectra, suggesting that the pigments differ only by the nature of the functional group attached to the molecule.' Which data are the authors referring to here? They need to reference one of their figures or tables so the reader can see this for themselves.	We realize the ideal method for determining whether the pigments are the same or no, is a chemical characterization by other means such as liquid chromatography coupled to a mass spectrometer or infrared spectroscopies, just to mention two widely used techniques. Nevertheless, such techniques require to perform a chemical extraction of the pigment, which was not attempted due to the small amount of material available. The use of optical properties is then proposed as an alternative method when the chemical extraction can not be performed. We attempted infrared spectroscopy directly on the cuticle of the insects. But since in this case there is a matrix in which the pigments are embedded, results

	showed subtle differences, but were not conclusive. This data is not included in the manuscript but it can be included if the referee considers it is necessary. Otherwise we have substituted this statement by the following:<br><br>"A chemical characterization using infrared spectroscopy was attempted directly on the cuticle and results showed subtle differences suggesting a similar nature of the functional group attached to the molecule. Such results are not shown here because they are not conclusive due to the fact that the exact chemical composition of the cuticle for each color is unknown. A definitive identification of the pigment requires to perform a chemical extraction which at the moment could not be done due to the small amount of cuticle available."<br>Done ✔
Reviewer 1: Line 358-9. 'A correlation between spectral color and phylogenetic relationship in beetles of the genus Chrysina (Scarabaeidae) has been observed in ongoing research by our group.' See my above comment about lines 289-91.	Recommendation taken. See below.<br>Done ✔
Reviewer 1: Line 363-4. 'Among the few phylogenetic correlations detected was for Evaniella' This does not make sense. What is Evaniella correlated with, and in what sense is this correlation phylogenetic?	The paragraph now reads:<br>*Evaniella* belongs to a completely different family (Evaniidae) and might therefore be expected to differ markedly from all the other genera. This indeed appears to be the case for its black color, but not its orange color (Fig. 7). This is an interesting result in that it might suggest that the orange color shows more convergent evolution than does the black color, but it remains to be demonstrated that the pigments are actually convergent. Nonetheless, within Scelionidae there are more differences in the orange color than in the black (Table 4). Comparing the results shown in Fig. 7 with a recent

	phylogenetic reconstruction of scelionid genera [35], a few other relationships between spectral properties and phylogeny appear to be present. For example, *Baryconus* and *Chromoteleia* are paired in the green and red components for both black and orange, and these two genera are hypothesized to be closely related. *Opisthacantha* and *Triteleia* group together in the blue and green components of the orange coloration, and these two genera are also closely related. On the other hand, the orange color of *Macroteleia* and *Sceliomorpha* group together for the green and red components (Fig. 7B) despite these genera not being closely related. These results are intriguing and merit further investigation to determine whether certain spectral properties parallel the phylogeny."
Reviewer 1: Line 366-8. 'This is an interesting result in that it suggests that the orange color shows more convergent evolution than does the black color.' This is a dangerously bold claim to make, because this claim is based off of just one single (poorly explained) comparison.	See above.
Reviewer 1: Line 370-1. 'In some other insects, contrasting black and orange color patterns are known to serve as aposematic (warning) coloration for potential predators [32].' How big are those insects—are they anywhere near the size of the wasps examined here? One wonders whether the predators that might be deterred by aposematic coloration on these wasps would have the visual acuity to notice the orangeon-black coloration in the first place.	Ladybird beetles are slightly larger than scelionid wasps, but not by much. The largest scelionid overlaps in size with the smallest ladybird.<br><br>We are currently carrying out experiments with jumping spiders, which appear to have the necessary visual acuity to detect the scelionid orange-on-black

Reviewer 1: Line 370-2. 'In some other insects, contrasting black and orange color patterns are known to serve as aposematic (warning) coloration for potential predators [32] and it is likely that the same is true of the BOB pattern, although this has not yet been tested.' In order for this color pattern to be aposematic in these wasps, they must be poisonous/venomous, or they must be mimicking a poisonous/venomous taxon. If the authors wish to argue that these color patterns are 'likely' aposematic, they must explain which poisonous/venomous taxa are involved.	We agree and have therefore changed "likely" to "possible". The indirect evidence provided in Mora & Hanson 2019 suggests aposematism and/or mimicry, but future research is needed to address this question.
Reviewer 1: Line 376-7. 'it is hoped that the results of the present spectral analyses provide a framework for future research.' The authors should say how this would be the case—according to this study, how should future research change?	Added:<br>"...especially when a direct chemical characterization of the pigments is not possible and the optical and statistical methods proposed in this work can be applied." at the end of this line.<br><br>Done ✔
Reviewer 1: At the end of the manuscript, the figures are in a random order. If I review this manuscript again I would like to see the figures presented in the correct order, and I would like to see the figure captions included directly below each figure so that I do not need to scroll back and forth.	We followed the instructions on the submission system and we are not entirely sure on why the figures are in a random order (we submitted them in order). Also, the figure captions are included directly below the first reference to the figure in the text as indicated in the PLOS one system instructions: "**Place** figure captions in the manuscript text in read order, immediately following the paragraph where the figure is first cited. Do not include captions as part of the figure files or submit them in a separate document." https://journals.plos.org/plosone/s/figures#loc-how-to-submit-figures-and-captions<br>Done ✔

Reviewer 1: Various figures contain unnecessary legends that should be removed: figures 4, 5, 6, and especially 2 and 8.	Removed legends in figures 2 (Fig2.tiff) 4 (Fig4.tiff), 5 (Fig5.tiff), 6 *Fig6.tiff) and 8 (Fig8.tiff).<br>Done ✔
Reviewer 2 (chosen color to highlight the suggestions of this reviewer in the manuscript)<br>I would recommend that the authors make the introduction and methods sections more concise; the methods section is on the long side and some information could perhaps be moved to the supplementary material.	Same suggestion as Reviewer 1. See above for comments.
Reviewer 2: the discussion section is lacking somewhat with regard to the potential usefulness and impact of the study and would appreciate some more substantive explanation in this regard. Nevertheless, if this approach will form the basis of future studies from this group then I am intrigued to see results of the correlation between spectral colour and phylogenetic relationships mentioned in the case of the *Chrysina* beetle genus.	The following paragraph was added at the beginning of the Discussion for the guidance of the reader:<br><br>"The following discussion is focused mainly on the contribution of the different statistical approaches to the physical and biological interpretation of the data obtained following the procedures proposed, with the aim of establishing a quantitative analysis method for the study of cuticle color of insects. This section has two main parts: discussion about differences and suitability of the statistical methods and the interpretation of the physical results within a biological context. "<br><br>The following line was rewritten and moved right after the new first paragraph of Discussion:<br><br>"Regarding the suitability of the statistical methods applied, the present study highlights the differences in terms of results that could arise using univariate methods, which ignore physical information contained in the reflectance measurement as a function of wavelength, versus analyzing data using FDA techniques."<br><br>Also, we think that some of the changes that have been made in the manuscript as a

	whole contribute to stating more clearly the potential impact of our study:<br><br>● In response to the comments of the referees, the aim of our work is now stated more clearly in the introduction, and we hope this clarifies the impact of the study.<br>● As suggested by a referee, we have added a conclusion section, which clarifies the contribution of our research.<br>● As a result of comments made by another referee, the mention of Chrysina studies was eliminated from the manuscript since this research is not yet ready for publication. Nevertheless, we want to clarify that it was the work on Chrysina that inspired us to try to find a phylogenetic correlation in this study, not the other way around.<br>Done ✔
Reviewer 2: Line 18 – pheomelanin is a type of melanin so with regard to browncoloured cuticle, eumelanin should be specified rather than saying melanin	Done ✔
Reviewer 2: Line 23 – change ‘arose’ to ‘arises’ to keep tense consistent	Done ✔
Reviewer 2: Line 24 – can the authors mention some examples of other taxa with respect to shared metabolic and genetic routes	The part about metabolic and genetic routes is confusing and so we have deleted it.<br>Done ✔
Reviewer 2: Figure 1 caption – what information is the photograph (Fig. 1C?) providing? I couldn’t see any highlighted square? The two highlighted areas both look circular to me.	Done ✔

Reviewer 2: Line 44 – 'A statistical approach of reflectance measurements…' does not read well. I suggest changing 'of' to something like 'based upon'	Replaced "of" for "based upon.<br>Done ✔
Reviewer 2: Line 44-51 – This final paragraph of the introduction feels more suited to the methods section<br>Reviewer 2: Line 137 – As someone who's not an expert on parasitic wasps I think a short explanation of why Evianella is a suitable control specimen would be helpful.	Done ✔<br><br>Done ✔
Reviewer 2: Line 147 – presumably should read 'as defined in Eq. 1'	Done ✔
Reviewer 2: Line 213 – 'The difference reflectance…' does not make sense. Change to something like 'The difference in reflectance measured for the curves…'	Reviewer 1 also commented on this line. Changed the beginning of the sentence to "The difference curves for reflectance spectra"<br>Done ✔
Reviewer 2: Line 282 – 'Fig 6 in panel (D)' should be changed to 'Fig 6D shows' or 'Panel (D) of Fig 6 shows'	Changed 'Fig 6 in panel (D)' for 'Panel (D) of Fig 6 shows'.<br><br>Done ✔
Reviewer 2: Line 291 – typo in figure reference link	Done ✔
Reviewer 3: **(chosen color to highlight the suggestions of this reviewer in the manuscript)**<br><br>For the first time , abstract	Done ✔
Reviewer 3: word corrector eliminations line 7, 8 abstract	Done ✔
Reviewer 3: word corrector eliminations line 13,15 and 16, introduction	Done ✔

Reviewer 3: line 20 comment<br>This is not correct. Scelionidae is a large and cosmopolitan family and the vast majority of them do not have the BOB pattern. Furthermore, this pattern is limited to the Neotropics. The same genera in N. America, Africa, etc. do not have this pattern.	We have incorporated this important clarification: "Various scelionid wasps, principally neotropical species ranging from 3 to 10 mm in length, show a recurring color pattern of …" However, the color pattern does occur in other parts of the world, especially in non-scelionid hymenopterans (see Mora & Hanson, 2019).<br>Done ✔
Reviewer 3: line 23 comment<br>I strongly disagree. The shared metabolic and genetic routes of the genera treated here are also shared by these genera outside of the Neotropics, where these color patterns do not occur. So, it cannot be a function of shared ancestry.	Done ✔<br>We have deleted this part about "shared metabolic and genetic routes".
Reviewer 3: Figure 1 legend, one word corrector elimination and meaning of 0,6 mm measurement	Done ✔
Reviewer 3: line 38, 39 and 42 word corrector eliminations and verb past tense	Done ✔
Reviewer 3:line 41<br>Functional data analysis (FDA) is a powerfu<br>These words are all capitalized earlier in the manuscript. Please be consistent about capitalization.	Done ✔
Reviewer 3: line 45 and 46 taxonomy/typos	Done ✔

Reviewer 3: line 65,66,67 author of genus	Done ✔
Reviewer 3: line 66<br>*Lapitha* (LA), *Macroteleia* (MA), *Scelio* (SC), [illegible]<br>Lapitha is not a valid genus. It was treated as a junior synonym of Opisthacantha by Masner 1976 and has not been resurrected. The author of Opisthacantha is Ashmead	Done ✔
Reviewer 3: line 67 change words for collected	Done ✔
Reviewer 3: line 68 author of genus	Done ✔
Reviewer 3: line 69 extra ,	Done ✔
Reviewer 3: line 73 word corrector elimination	Done ✔
Reviewer 3: line 74<br>Specimens were identified by R.M. and P.H.<br>Identified using what key? Masner 1976 should be referenced if that was how the specimens were identified.	We added this reference, which was indeed used for identification.<br>Done ✔
Reviewer 3: line 76<br>Due to the lack of taxonomic studies, species<br>Acanthoscelio was revised in 2001 (Dotseth & Johnson); Chromoteleia was revised in 2018 (Chen et al.: https://zookeys.pensoft.net/article/25775/)	This detail has been incorporated into the text<br>Done ✔
Reviewer 3: line 76 and 79 word corrector elimination and word incorporation	Done ✔

Reviewer 3: line 79<br>If the taxonomy has not been studied, how do you know that males and females are correctly associated?	Now reads: "...the vast majority hymenopterans with the BOB pattern, and those that are all black, do not show sexual dimorphism in color, at least in specimens that have been identified to species level in museum collections [15]."<br>Done ✔
Reviewer 3: line 83 and 84, 3 word corrector suggestions	Done ✔
Reviewer 3: Line 89<br>I suggest using a term other than "segment", which has a specific meaning in insect morphology. How about " section"?	Done ✔
Reviewer 3: line 110 word corrector suggestion	Done ✔
Reviewer 3: line 150, word corrector elimination	Done ✔
Reviewer 3, line 196<br>download in https://github.com/malfaro2<br>This should also be included as supplemental data with the publication.	We will try to include it in the submission system as Supplemental material in a .zip file, but it is worth noticing that the zip file is 86.3MB, and we do not know if it exceeds the limit.
Reviewer 3, Line 210, 213, 215 word corrector incorporations	Done ✔
Reviewer 3, Line 234 word corrector change of word	Done ✔
Reviewer 3, Line 241 simplify the sentence (The most important factor in this case is when there is a change of color, since the estimate of η is 242 26.2081, which means that keeping all the other factors constant, the	Deleted "The most important factor in this case is when there is a change of color, since"<br>Done ✔.

average ΔE is 26 243 units greater than the overall mean when the curves differ in color).	
Reviewer 3, Line 262 word correction	Done ✔
Reviewer 3, Line 274<br>Are green, red, and blue used because they correspond to human photoreceptors? If so, that should be clarified, and what are the wavelengths attributed to each of these colors?	Yes. This calculation is based on the human photoreceptors. We are referring to spectral components, not to a single wavelength. Nevertheless, it is possible to define a characteristic wavelength for each curve, which is the wavelength at which the maximum of the color matching function occurs. Since a comment in this sense corresponds more to the description of the calculation of the color components, we added the information about the corresponding wavelengths but in the section **"RGB components"** of the **Methods section** instead of the Results section.<br><br>"We label these components as Green, Red and Blue (RGB) because their maximum amplitude occur at 700 nm, 546 nm and 436 nm respectively."<br>Done ✔
Reviewer 3, Line 283 word corrections	Done ✔
Reviewer 3, Line<br>spectra of beetles of the genus *Chrysina* (Sca<br>tree. Following the same line of thought. Fig<br>"with respect to its phylogenetric tree" can mean many things. Please clarify what you mean. Also, please follow Chrysina with the author of this genus name.	This line was eliminated, due to the fact that it refers to results that are still in study and have not been published yet<br>Done ✔
Reviewer 3, Line 313 and 315 word corrections	Done ✔
Reviewer 3, Line 350	No. The spectral components are curves that were normalized using the area under the curve. This procedure make the curves

Could it also be the case the spectral blue components are unrelated to the pigments that create the orange-black colors?	comparable between them. So, even if orange is expected to have less blue than black, for example, the information related to chemical composition of the pigment is contained in features such as the shape of the curves and the wavelength at which the maximum occurs. For the sake of clarity, the line was modified as follows:<br><br>"In the case of pigments of different colors (black vs. orange), the spectral blue components remain almost identical in terms of shape and characteristic wavelength (where the maximum occurs), suggesting that there is a common compound for the pigments. Notice that the normalization of the spectral components, equations (2), (3) and (4), allows the use of these features for comparison instead instead of intensity, which is related to the quantity of red, green or blue present in each color.<br>Done ✔
Reviewer 3, Line 367<br>Having a similar spectral profile is not the same as convergent evolution. You would first need to provide evidence that the pigments are actually convergent in some way. It could be the case that very different pigments simply have a similar spectral profile.	We have made this clarification in the text: "This is an interesting result in that it might suggest that the orange color shows more convergent evolution than does the black color, but it remains to be demonstrated that the pigments are actually convergent."<br>Done ✔
Reviewer 3, Line 371 word corrector elimination	Done ✔
Reviewer 3, Line 374	We have made this clarification in the text: "...thermoregulation (black absorbs more sunlight) might play a role at these elevations; it is also possible that predators or aposematic models vary with elevation."<br>Done ✔

Is this asserting that they would be black to absorb more sunlight? It could also be that there are different predators at high elevation, or the aposematic model of the mimicry complex is not present at higher elevation. It should be noted that BOB occurs in large scelionids.	
Please ensure that your manuscript meets PLOS ONE's style requirements, including those for file naming. The PLOS ONE style templates can be found at http://www.journals.plos.org/plosone/s/file?id=wjVg/PLOSOne_formatting_sample_main_body.pdf and http://www.journals.plos.org/plosone/s/file?id=ba62/PLOSOne_formatting_sample_title_authors_affiliations.pdf	Done ✔
While revising your submission, please upload your figure files to the Preflight Analysis and Conversion Engine (PACE) digital diagnostic tool, https://pacev2.apexcovantage.com/. PACE helps ensure that figures meet PLOS requirements. To use PACE, you must first register as a user. Registration is free. Then, login and navigate to the UPLOAD tab, where you will find detailed instructions on how to use the tool. If you encounter any issues or have any questions when using PACE, please email us at figures@plos.org. Please note that Supporting Information files do not need this step.	Done ✔

Data Review URL

Click here to download Data Review URL
http://github.com/malfaro2/Mora_et_al
