# Supplementary material for: Spectral measure of color variation of black-orange-black (BOB) pattern in small parasitoid wasps (Hymenoptera: Scelionidae), a statistical approach
Source: PLoS One. 2019 Oct 24;14(10):e0218061. doi: 10.1371/journal.pone.0218061 (PMC6812806; doi:10.1371/journal.pone.0218061)
Supplement: Attachment — Submitted filename: Response_to_reviewers.pdf [file pone.0218061.s016.pdf]

RESPONSE TO REVIEWERS

Academic Editor
PLOS ONE

Dear Phillip Barden,

We would like to thank the reviewers for their critical assessment of our work. In the following rebuttal letter, we address each point raised by the two reviewers.

Reviewer #1 **highlighted in red color in manuscript.**

Scelionidae are parasitoids, not parasites. **DONE**

l 2, 455, etc. “(un)characterized” how? Some people might think that calling a color “orange” is a form of characterization. **DONE (changed to “have not been analyzed”, “analyzing”, etc.)**

l 5 says "2 to 10 mm" but l 40 & 456 say "3 to 10 mm", which is it? **DONE**

l 12. "both inter and intragenera" -> "both between and within genera". **DONE**

l 35, 39, etc. Need taxonomic authorities for genera and species. **DONE**

Fig. 1 caption. Make the 2 superscripts in "mm2". **DONE**

l 74-5. What about the evaniid? **DONE**

l 83. "Hymenopteran" -> "hymenopteran" **DONE**

l 94. Need a space between "Acanthoscelio" and "Ashmead". **DONE**

l 95. Need a space between "Westwood," and "Opisthacantha". **DONE**

l 110, 111, 230. Remove the spaces before the periods. **DONE**

l 115. Remove "by". **DONE**

L 122 It's odd that Fig. 2 is cited in the Methods, since this figure shows the Results of this study. This line was replaced by “Spectral reflectance curves can be studied in terms of colors using a geometrical representation or color space.” Figure 2 reference was moved to the line after the first paragraph of Results, where is mentioned for the first time. **DONE**

l 153. Italicize the E in Delta-E. **DONE**

l 160. Remove "in this work, ". **DONE**

l 167. Remove the second period. **DONE**

l 178. "referred as" -> "called" **DONE**

l 179. What about the metasoma? **DONE**

l 214. Many journals prefer a monospace font (\texttt{} in LaTeX) for the names of software packages, such as erpFtest and fdANOVA. **DONE**

l 225. Shouldn't "species" be "genera"? **DONE**

l 229. Should there be a comma between "i" and "and"? **DONE**

l 230. Remove "In order". **DONE**

l 233. "accounting" -> "that accounts" **DONE**

l 242. Remove the parentheses (). **DONE**

l 245. See my comment about l 214. **DONE**

l 247. "provide support for". This paragraph changed, see next comment. **DONE**

l 247. State the meaning of "reflectance difference variations between and within genera". This statement was replaced by “Reflectance curves in Fig~\ref{fig:F2} show differences both when comparing curves measured for the same genera and curves representing the reflectance of different genera. At the same time, such differences present variability within the group of measurements performed. For both between and within genera cases, this reflectance differences are statistically compared to zero in both the univariate data and the functional data (FDA method) (p-value < 0.00001 in all cases).” **DONE**

l 258. "in some cases" -> "for some genera" **DONE**

l 260. "that" -> "than" **DONE**

l 263. Put a comma after the closed paren. **DONE**

l 267-8. Explain to the reader what it means when "differences are below zero". **DONE**

l 269. Add a comma after "750nm". **DONE**

l 272. Remind the reader of what this hypothesis means. **DONE**

l 280. "are" -> "is" **DONE**

l 282. Why isn't the "Fourier basis function representation" mentioned in the Methods? It is the method performed by \texttt{fdANOVA}, we added the clarification. **DONE**

l 285-6. Do the authors mean to say "from the MEAN difference between the black and orange spots in the other genera"? Correct, thank you for the correction. **DONE**

l 311. Replace the hyphens with em dashes (--- in LaTeX). **DONE**

l 312. Remove "and Acanthoscelio". **DONE**

l 321. “maximum and minimum mean” is somewhat tortured/tortuous language. I’d rephrase this. **DONE**

l 368. I really like this opening to the Discussion. I’d suggest using subheadings for the two subsections. **DONE**

l 370. Remove “in terms of results”. **DONE**

l 379. “Functional Data Analysis (FDA)” -> “FDA” you already defined this acronym. **DONE**

l 395. “This particular method” make it clear which method you’re talking about, you mentioned various methods in the previous paragraph.**DONE**

l 430-1. What do the authors mean by “the pigments are actually convergent” - did wasps in both families converge on use of the same pigment, or do they use two different pigments that

produce colors with convergent reflectance properties? **DONE.** This sentence was eliminated as suggested by reviewer 2.

l 563. Capitalize "B" in "batesian" **DONE**

l 569, 592. Need a space before the open paren. **DONE**

Reviewer #2:

I think that the speculation about convergent evolution is unfounded and should be removed entirely. **DONE**

There are some grammatical corrections in the attached pdf. **DONE**

Thank you very much for the comment we did a detailed and careful reading and found 8 grammatical errors that were corrected.
