# Supplementary material for: Spectral measure of color variation of black-orange-black (BOB) pattern in small parasitoid wasps (Hymenoptera: Scelionidae), a statistical approach
Source: PLoS One. 2019 Oct 24;14(10):e0218061. doi: 10.1371/journal.pone.0218061 (PMC6812806; doi:10.1371/journal.pone.0218061)
Supplement: Attachment — Submitted filename: PONE-D-19-14642_reviewer_1_comments.docx [file pone.0218061.s013.docx]

PONE-D-19-14642 reviewer comments

General comments

The authors have presented a rigorous analysis of the reflectance spectra from orange and black regions of 8 genera of scelionid wasps, highlighting subtle differences in reflectance spectra between different genera – these differences may identify similarities and/or differences in chemical composition i.e. melanin type and concentration within the cuticle of the wasps. On the whole the paper is well written and the results discussed in a logical order. As a minor point, I would recommend that the authors make the introduction and methods sections more concise; the methods section is on the long side and some information could perhaps be moved to the supplementary material. In addition, I feel that the discussion section is lacking somewhat with regard to the potential usefulness and impact of the study and would appreciate some more substantive explanation in this regard. Nevertheless, if this approach will form the basis of future studies from this group then I am intrigued to see results of the correlation between spectral colour and phylogenetic relationships mentioned in the case of the *Chrysina* beetle genus.

Specific comments

Line 18 – pheomelanin is a type of melanin so with regard to brown‑coloured cuticle, eumelanin should be specified rather than saying melanin

Line 23 – change ‘arose’ to ‘arises’ to keep tense consistent

Line 24 – can the authors mention some examples of other taxa with respect to shared metabolic and genetic routes

Figure 1 caption – what information is the photograph (Fig. 1C?) providing? I couldn’t see any highlighted square? The two highlighted areas both look circular to me.

Line 44 – ‘A statistical approach of reflectance measurements…’ does not read well. I suggest changing ‘of’ to something like ‘based upon’

Line 44-51 – This final paragraph of the introduction feels more suited to the methods section

Line 137 – As someone who’s not an expert on parasitic wasps I think a short explanation of why Evianella is a suitable control specimen would be helpful

Line 147 – presumably should read ‘as defined in Eq. 1’

Line 213 – ‘The difference reflectance…’ does not make sense. Change to something like ‘The difference in reflectance measured for the curves…’

Line 282 – ‘Fig 6 in panel (D)’ should be changed to ‘Fig 6D shows’ or ‘Panel (D) of Fig 6 shows’

Line 291 – typo in figure reference link
